# Non-Catalyzed Click Reactions of ADIBO Derivatives with 5-Methyluridine Azides and Conformational Study of the Resulting Triazoles

**DOI:** 10.1371/journal.pone.0144613

**Published:** 2015-12-16

**Authors:** Petra Smyslova, Igor Popa, Antonín Lyčka, Gracian Tejral, Jan Hlavac

**Affiliations:** 1 Institute of Molecular and Translation Medicine, Olomouc, Czech Republic; 2 Department of Organic Chemistry, Faculty of Science, Palacký University, Olomouc, Czech Republic; 3 University of Hradec Kralove, Faculty of Science, Hradec Kralove, Czech Republic; 4 Institute of Biophysics, Second Faculty of Medicine, Charles University, Praha 5, Czech Republic; 5 Laboratory of Tissue Engineering, Institute of Experimental Medicine, Academy of Sciences of the Czech Republic, Praha 4, Czech Republic; National Research Council of Italy, ITALY

## Abstract

Copper-free click reactions between a dibenzoazocine derivative and azides derived from 5-methyluridine were investigated. The non-catalyzed reaction yielded both regioisomers in an approximately equivalent ratio. The NMR spectra of each regioisomer revealed conformational isomery. The ratio of isomers was dependent on the type of regioisomer and the type of solvent. The synthesis of various analogs, a detailed NMR study and computational modeling provided evidence that the isomery was dependent on the interaction of the azocine and pyrimidine parts.

## Introduction

**Fig 1:**
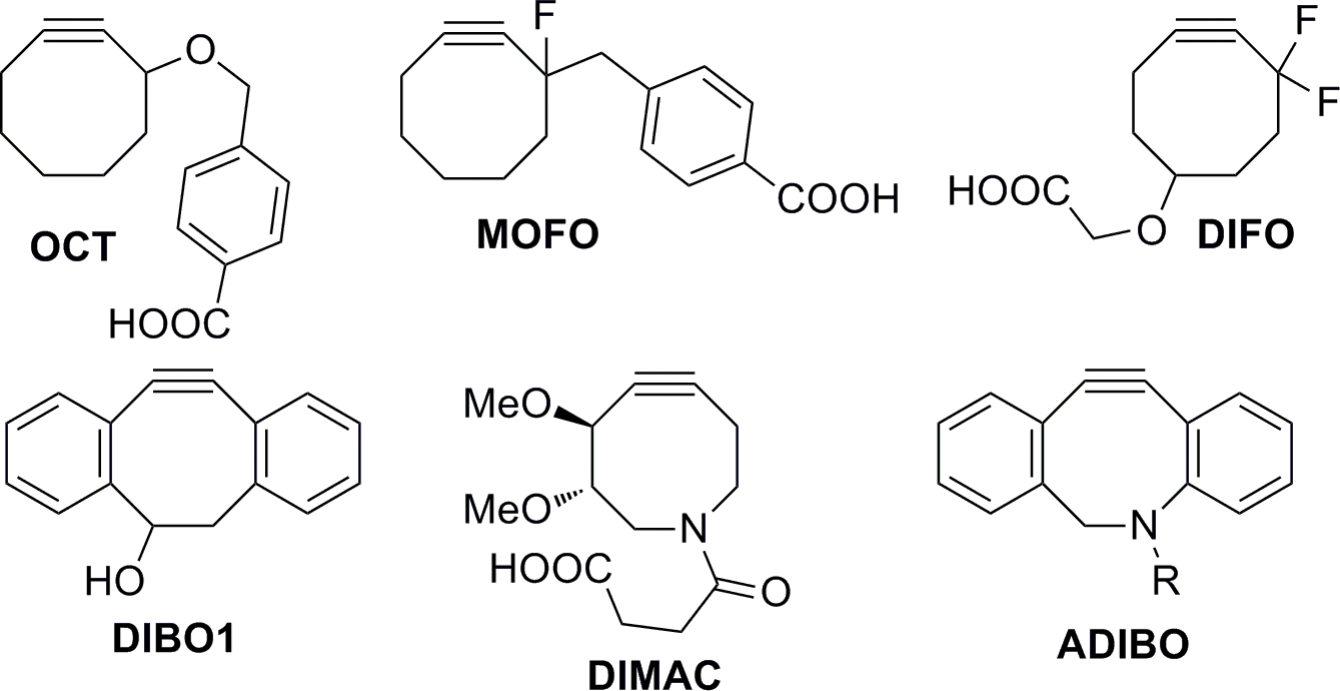
Cyclooctynes for copper-free click reactions.

Copper-free click reactions based on the strain-promoted alkyne-azide cycloaddition reaction (SPAAC) were discovered by Wittig and Krebs in 1961 [1]. During examination of the properties of cyclooctyne, they observed its rapid reaction with phenyl azide to yield a single triazole product [1,2]. In 2004, Bertozzi and co-workers first used the SPAAC with biotinylated cyclooctyne as a bioorthogonal reaction to modify biomolecules and living cells [3]. Various derivatives of cyclooctyne have been developed to improve the kinetics of cycloaddition (Fig 1) [4,5].

Incorporation of an electron-withdrawing fluorine in cyclooctyne leads to a significant increase in the reaction rate [4,5]. The use of dibenzocyclooctyne results in further acceleration due to the additional ring strain caused by the phenyl rings [4,5]. The introduction of nitrogen into cyclooctyne further improves the reaction rate [4,5] and facilitates binding of the necessary appendix for labeling or reaction with other substrates. In 2010, an aza-dibenzocyclooctyne motif (Fig 1), a combination of DIBO and DIMAC, was developed and used for the PEGylation of enzymes [6]. The use of ADIBO derivatives for a wide range of biological applications is exemplified by the work of Kjems and co-workers, who used an ADIBO moiety to ligate DNA to macromolecules to produce DNA conjugates with polymers, proteins and other large biomolecules [7] Pfeifer and co-workers then prepared ADIBO-activated glass slides for the immobilization of diagnostic peptides [8]. ADIBO derivatives have also been used to label membrane bilayers [9], 5´-capped RNA [10], antibodies [11] and proteins [12] The modification of nanoparticles with ADIBO for biological purposes is another research area [13–15]. ADIBO derivatives often serve as F-18 probes [16–20] or ^64^Cu radiolabeled probes [21–23] for PET imaging. Surprisingly, although ADIBO derivatives are widely used for copper-free click reactions, the structures of the final triazoles have been fully described for only three simple compounds in a single article [24] In that study, the reaction was performed between polymethoxy azocine and a few simple azides: 5-azidopentanoic acid, benzyl azide and 4-azidophenyl isothiocyanate. The reaction proceeded with slight regioselectivity, and no unexpected behavior was observed in the NMR spectra.

In our study, we investigated copper-free click reactions of ADIBO derivative **7** with azides derived from 5-methyluridine prepared using our newly developed procedures. The use of simple nucleosides led to the formation of triazoles, and the structures of the triazoles were characterized. Full characterization of the final products is crucial for describing bioorthogonal reactions. Copper-free click reactions with nucleobases using dibenzoazocine derivatives have been described in only two articles [25,26]. In the first, the structures of the final triazoles synthesized on azides derived from purine-based acyclovir and ganciclovir were not determined by standard analytical technique [26]. Wnuk and co-workers then described the reaction of 5-azidouridines and 8-azidopurines with dibenzoazocine to afford “a mixture of several inseparable regioisomers” identified by HPLC, although the reaction could afford in principle only two regioisomers [25].

The structure of triazoles derived from ADIBO and oligonucleotides or nucleosides has not been studied to date, although the vicinity of the bulky dibenzoazocine group to the nucleic base can play a significant role in the conformation of the DNA duplex, RNA strain assembly or protein tertiary structure when used for biomolecule labeling.

Here, we report the first results of a conformational study of triazoles formed directly on nucleosides at the 5´ position. This triazole formation could be used for the non-catalyzed 5´-end chemical labeling of oligonucleotides to bind DNA/RNA probes to other molecules or surfaces to enable target delivery or immobilization. The results were also verified for a derivative of 5-azidomethylene uridine to reveal potential difficulties with the labeling of oligonucleotides via base derivatization. To avoid negative or false-positive effects of the immobilized/labeled nucleic acid in a biological assay, an effect of biomolecule modifications to its structure should be elucidated.

## Result and Discussion

### Preparation of 5´- azides

**Fig 2:**
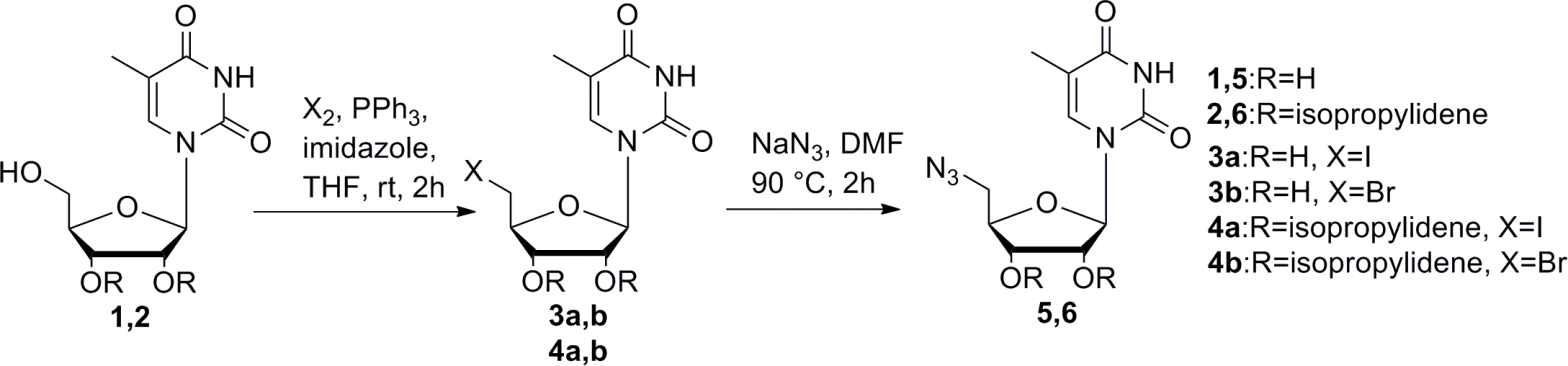
Preparation of azides 5 and 6.

Our synthesis of 5´-azidoderivative **5** was based on a two-step approach starting from 5-methyluridine (Scheme 1). The total yield of this reaction reached approximately 60%, which is more efficient than the previously described synthesis [27] beginning with protection of 5-methyluridine by acetone. The newly developed synthetic protocol was also successfully tested in the synthesis of protected 5´-azidoderivative **6** starting from protected 5-methyluridine **2**, prepared in a very good yield using the described procedure (Fig 2) [28].

### Copper-free click reactions

**Fig 3:**
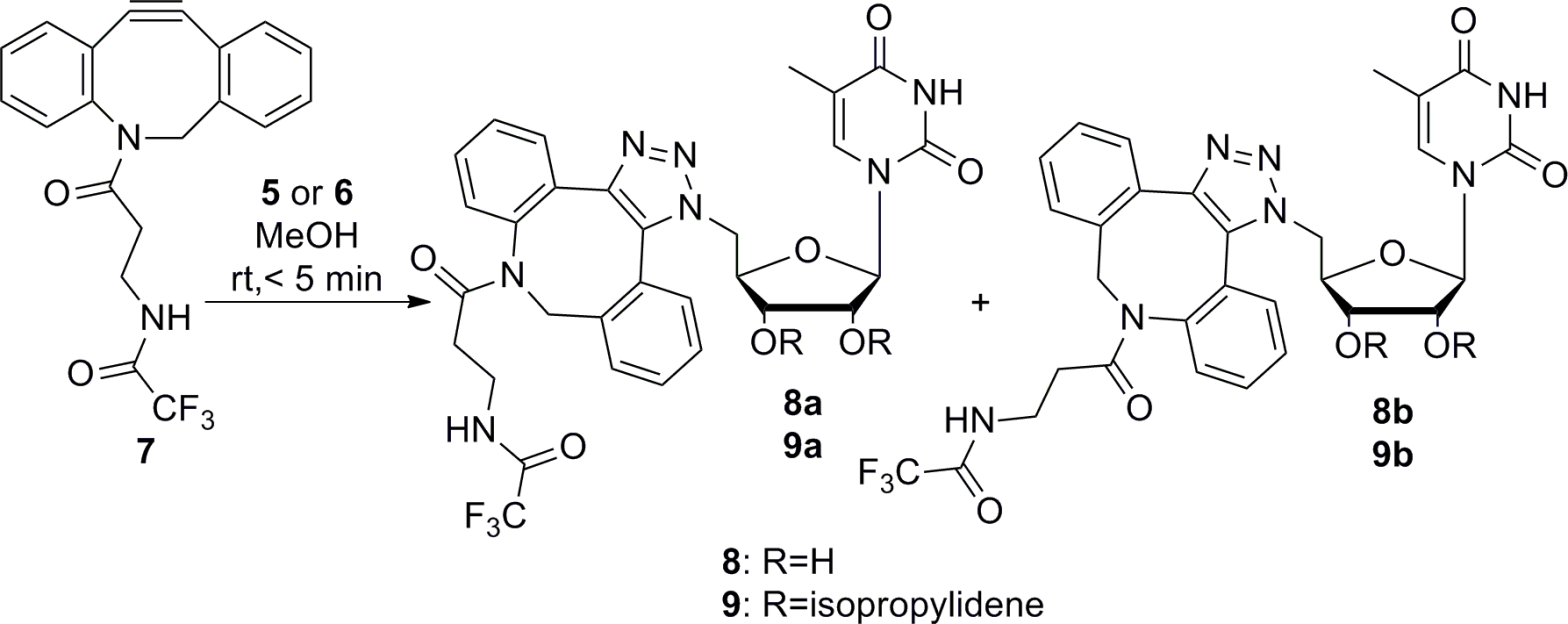
Copper-free click reaction with dibenzoazocine 7 and azides 5 and 6.

First, we studied the copper-free click reactions of azides **5** and **6** by treatment of dibenzoazocine derivative **7** (Fig 3), which was prepared as described previously [29]. This compound was highly reactive, and all reactions in methanol were nearly instantaneous. Both products were produced as a mixture of two regioisomers in a 1:1 ratio. The conversion of this reaction depended on the amount of azocine **7**. Complete conversion of the azides required at least 1.4 equivalents of **7**.

Isomers **8a,b** and **9a,b** were successfully separated by semi-preparative HPLC, yielding products in >99% purity. All isomers were subjected to detailed NMR study.

### NMR study

**Fig 4:**
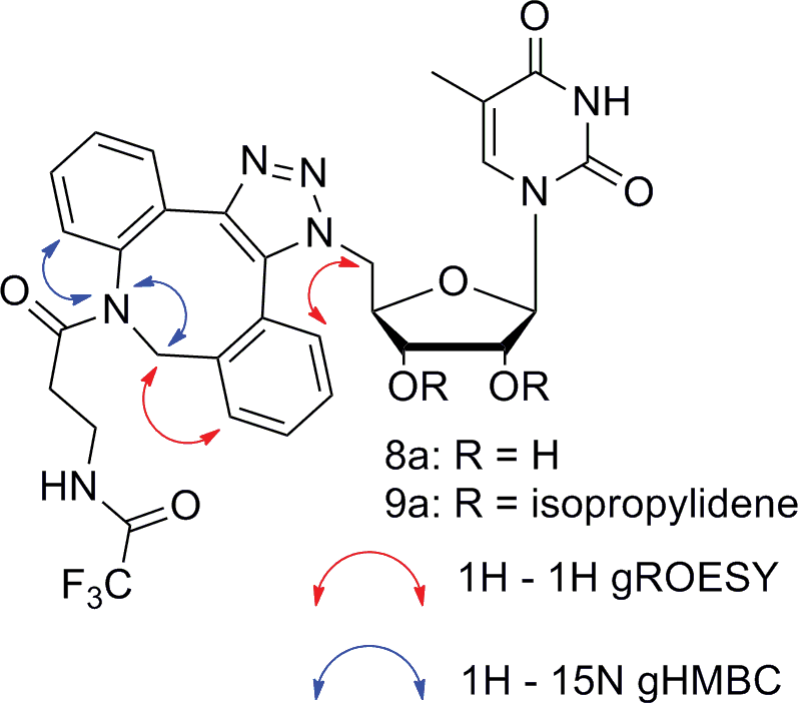
Homo and heteronuclear interactions used to determine the structures of derivatives 8a and 9a.

The structures of compounds **8a** and **9a** were clearly confirmed by the ^1^H, ^13^C and ^15^N signals assigned via 2D experiments, particularly ^1^H – ^1^H gROESY and ^1^H – ^15^N gHMBC. The correlation was based on the interaction of the azocine nitrogen with azocine methylene protons and with the aromatic proton next to the azocine nitrogen in ^1^H – ^15^N gHMBC. An interaction of the aromatic protons with the ribose methylene group and the azocine methylene group was observed in ^1^H – ^1^H gROESY as well (Fig 4).

Assigning the ^1^H, ^13^C and ^15^N signals (measured in CDCl_3_) of regioisomers **8b** and **9b** was problematic due to the presence of more forms of each pure compound (see below). Because the reaction course predetermines the formation of two regioisomers and the HRMS of compounds **8b** and **9b** afforded the same elemental composition as for **8a** and **9a**, respectively, we conclude that the structures correspond to the opposite regioisomers. The NMR spectra for **8b** and **9b** are described as the set of signals of all their present forms.

**Fig 5:**
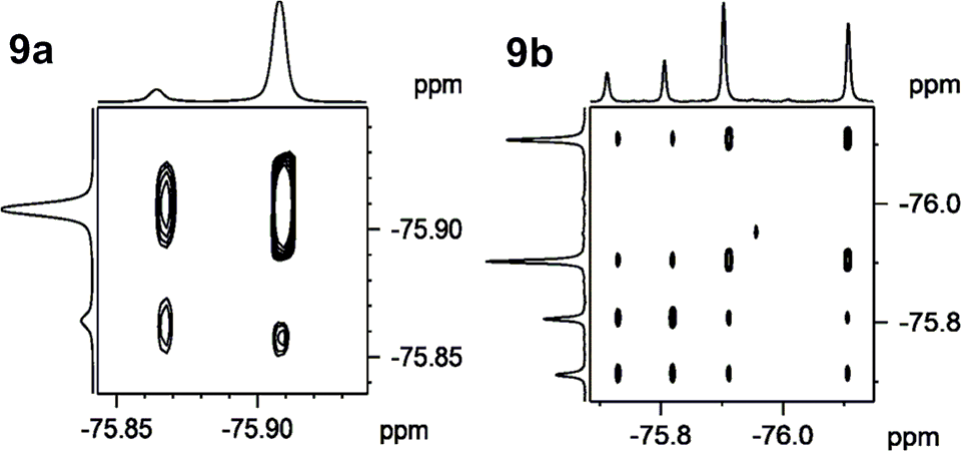
^19^F-^19^F EXSY of triazoles 9a and 9b (mixing time 1 s).

Although the purity of triazoles **8a,b** and **9a,b** was confirmed by HPLC before and after the NMR experiments and verified under several HPLC conditions, the ^1^H NMR spectra revealed the presence of two isomers of the **8a** and **9a** derivatives and even more for the **8b** as well as **9b** derivatives. The ^13^C NMR spectra of CDCl_3_ solutions also revealed more than the expected number of signals. For couple **9a**/**9b**, we performed standard ^19^F NMR and ^19^F-^19^F EXSY observed two resonances for **9a** and four resonances for **9b** (CDCl_3_). ^19^F-^19^F EXSY of both compounds revealed a mutual slow exchange between all existing forms, as evidenced by positive cross-peaks in the spectra (Fig 5). These positive cross-peaks confirmed exchange between all four existing forms of compound **9b** for a relatively wide range of mixing times from 0.05 to 2 s, indicating that the relative rates of exchange must be very similar.

**Fig 6:**
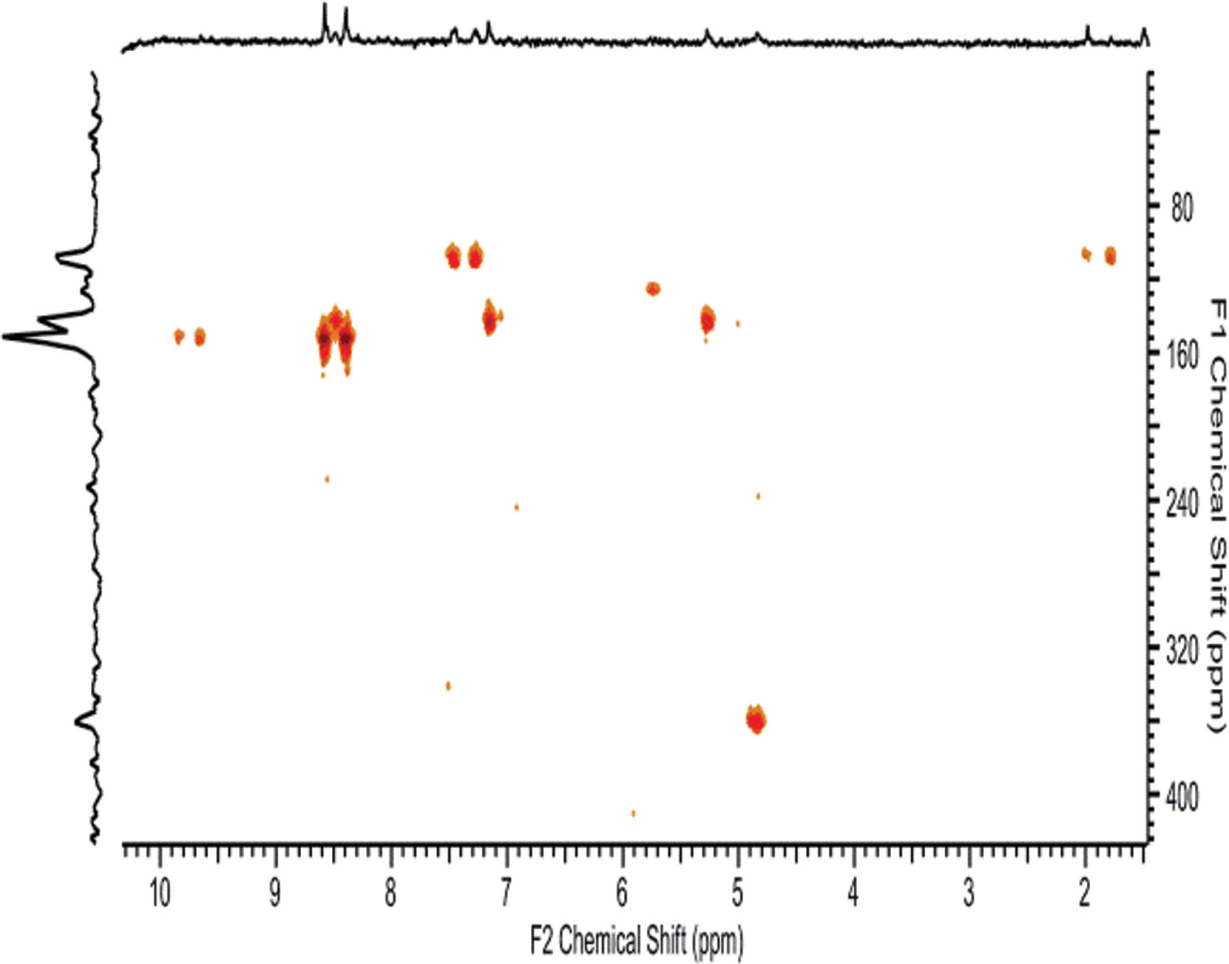
^1^H-^15^N HMBC of 9a in CDCl_3_.

^1^H-^15^N gHMQC and ^1^H-^15^N gHMBC experiments in CDCl_3_ suggested that the isomery was of conformational origin. Doubled signals of the pyrimidine NH group for compound **9a** at 150.75 ppm (Fig 6) likely hindered the rotation of the pyrimidine ring relative to the other parts of the molecule. Identical results were obtained in *d*_*6*_-DMSO.

**Fig 7:**
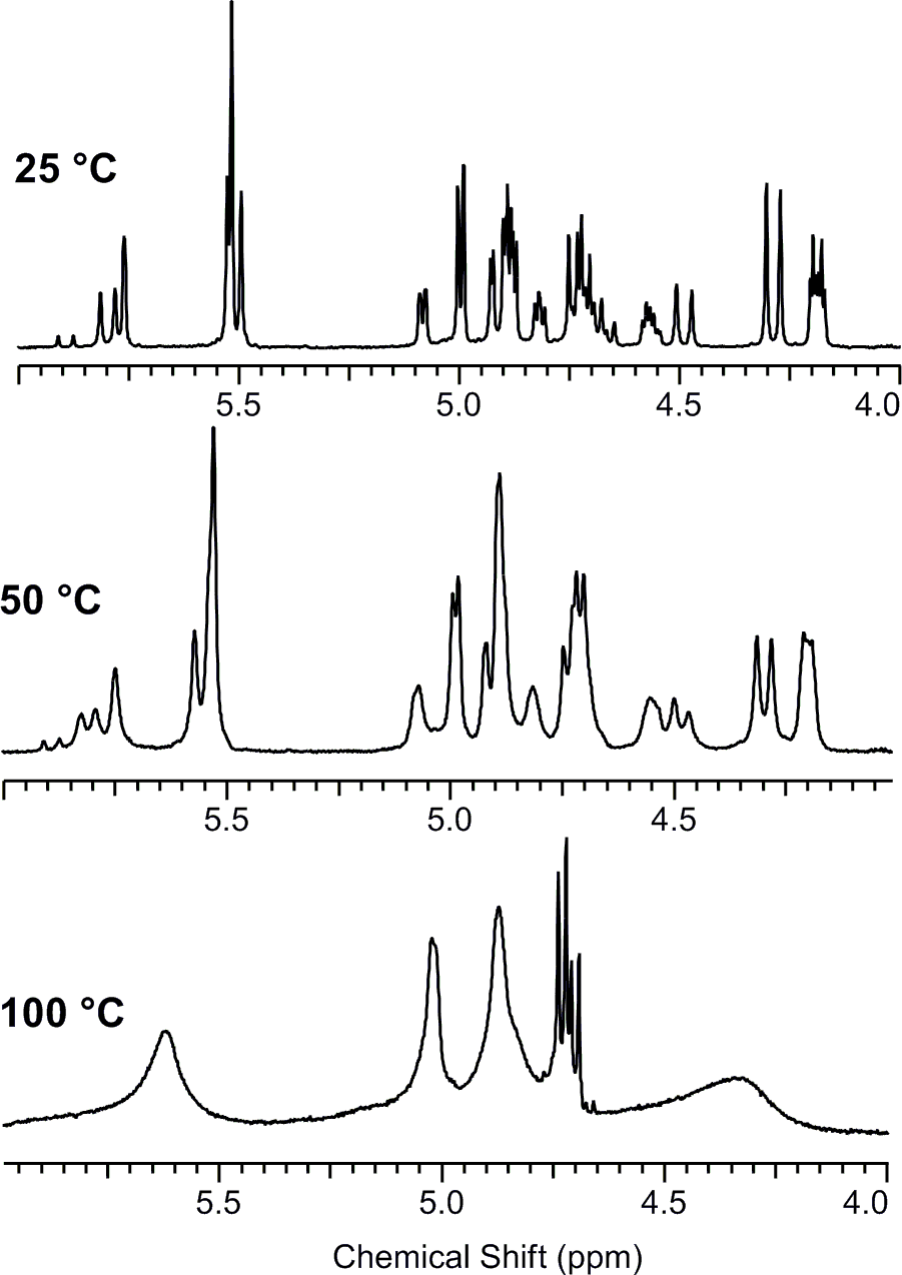
^1^H NMR spectra of 9a at different temperatures.

The ^1^H spectra of compounds **9a** and **9b** in *d*_*6*_-DMSO were measured at various temperatures (25°C, 50°C, 100°C and cooling back) to determine whether the number of isomers was affected by temperature. The spectral pattern was essentially unaffected until +50°C. Signal coalescence was finally observed at +100°C (Fig 7).

**Fig 8:**
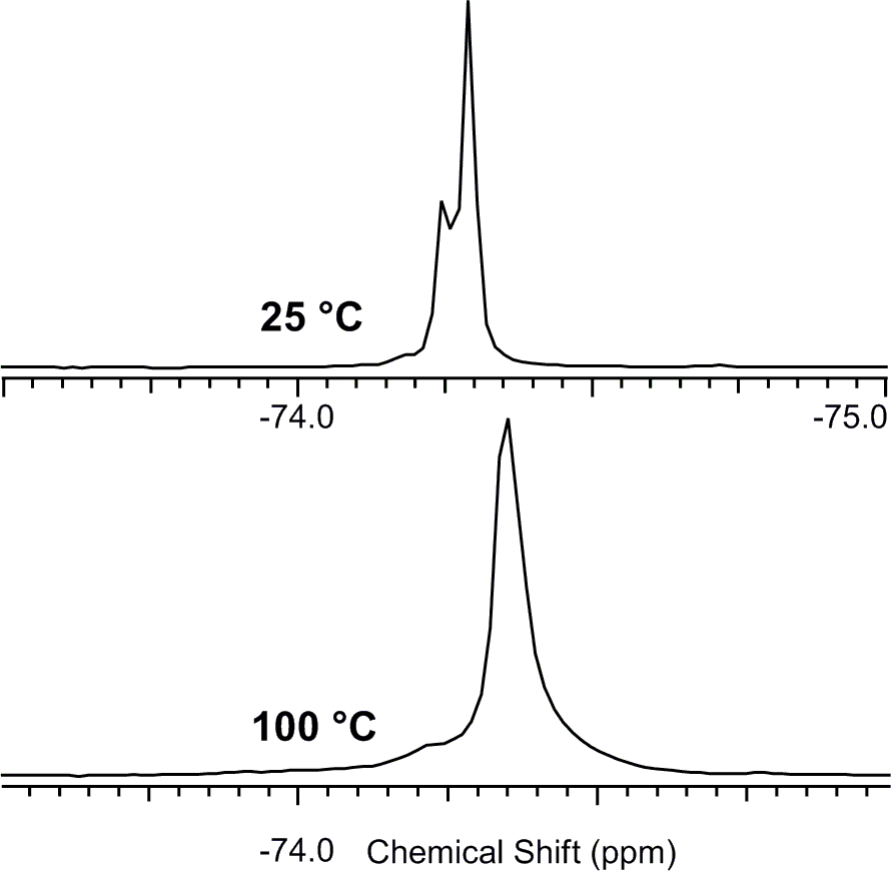
^19^F spectra of 9a at different temperatures.

Identical results were obtained for standard ^19^F spectra of **9a** and **9b** measured in DMSO, with signal coalescence at 100°C (Fig 8).

**Fig 9:**
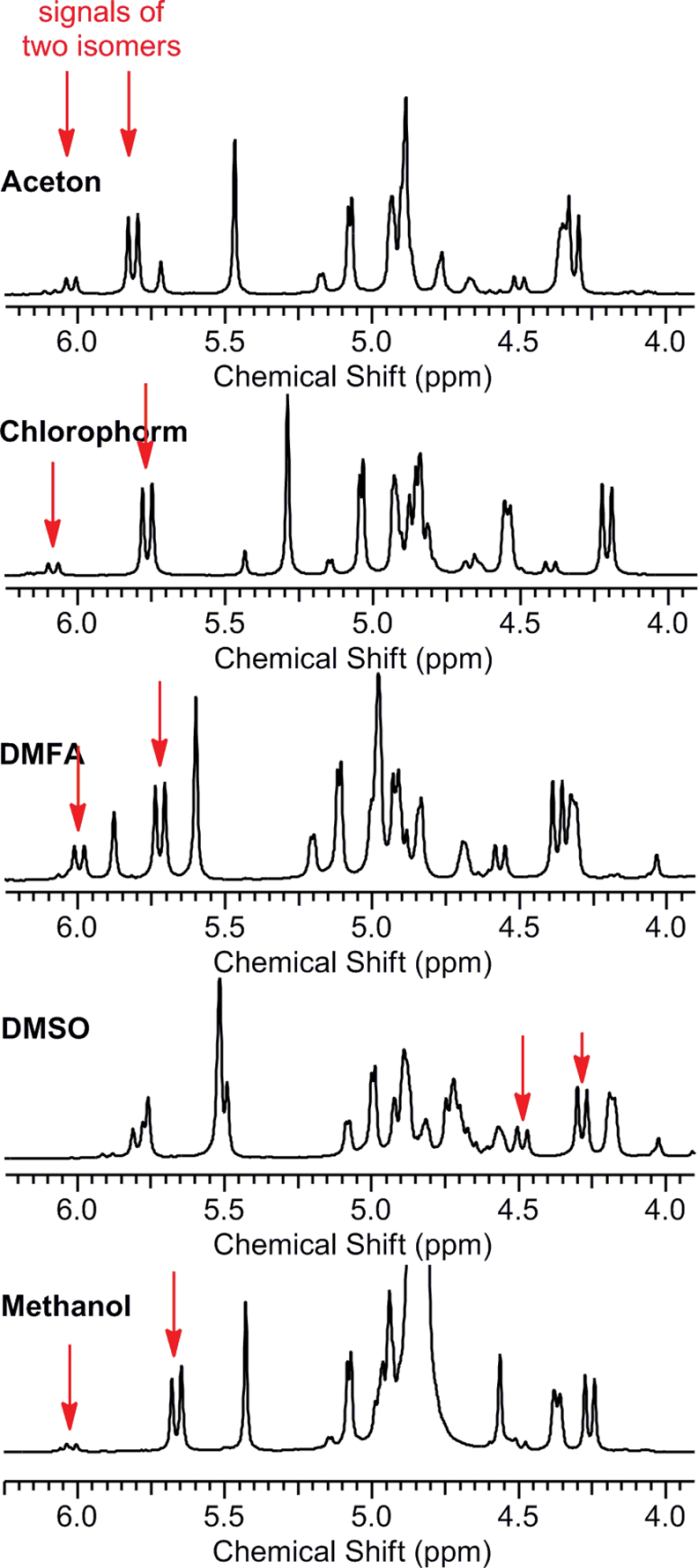
^1^H NMR spectra of 9a in various solvents.

To characterize the relationship between the number of isomers and the type of solvent, we extended the number of tested solvents to include acetone, D_2_O, MeOD, *d*_*6*_-DMSO and *d*_*7*_-DMFA for derivatives **8a** and **9a**, which were selected as representative model compounds. Changing the solvent not only shifted the signals but also affected their ratio (Fig 9).

The number of isomers of compounds **8a** and **9a** remained constant; only the ratio was affected. The dependence of the isomeric ratio on solvent is presented in Table 1.

**Table 1:** Ratio of isomers of compounds 8a and 9a.

8a		9a	
Solvent	Ratio*	Solvent	Ratio*
CD_3_OD	3.2:1	CD_3_OD	9:1
CDCl_3_	2.6:1	CDCl_3_	6.6:1
d_7_-DMFA	1.7:1	d_7_-DMFA	2.6:1
D_2_O	7.9:1	d_6_-Acetone	4:1
d_6_-DMSO	1.4:1	d_6_-DMSO	2.2:1

*The ratio was determined from the peak integrals of the azocine methylene group protons at approximately 5.75 ppm and 6.00 ppm or at 4.25 ppm and 4.50 ppm (see S13–S17 and S28–S32 Figs).

**Fig 10:**
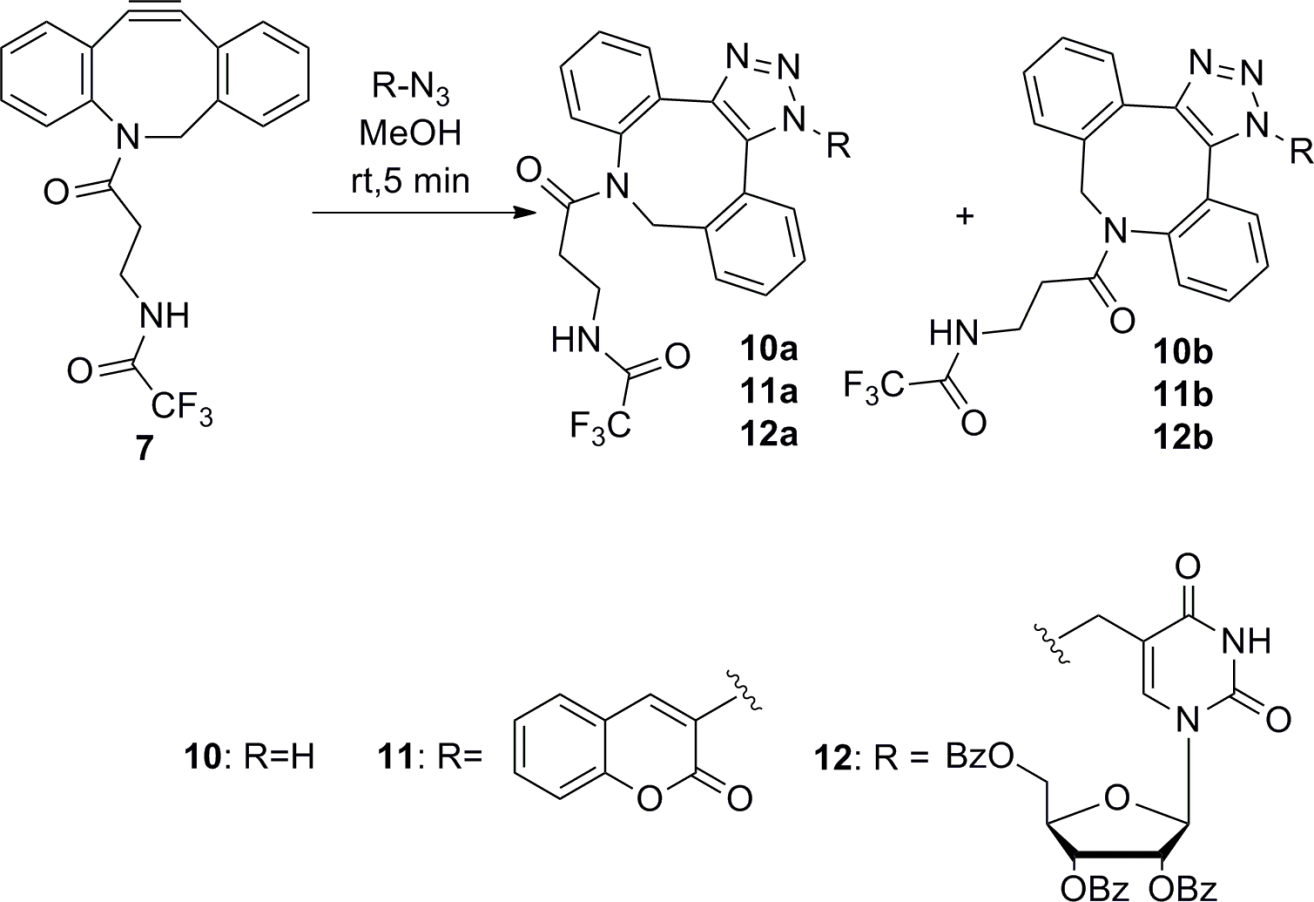
Preparation of triazoles 10, 11 and 12.

To determine whether the presence of isomers was caused by the nucleoside part of molecule or by s-cis, s-trans isomery of the amide groups on the azocine moiety, we prepared triazoles **10–12** substituted on nitrogen by only hydrogen, the sterically bulkier coumarin, and by 2´,3´,5´tribenzoyl-5-methyluridine, which mimics the bulky surrounding in a nucleotide (Fig 10).

The azidocoumarin as the starting material was synthesized as described previously [30]. 2´,3´,5´Tribenzoyl-5-azidomethyluridine was synthesized using a simple procedure starting from 5-hydroxymethyleneuridine[31].

Using a simple triazole with hydrogen, we obtained only a single regioisomer **10a**, whereas 3-azidocoumarine and protected 5-methyluridine formed two regioisomers, **11a/11b** and **12a/12b**, respectively.

**Fig 11:**
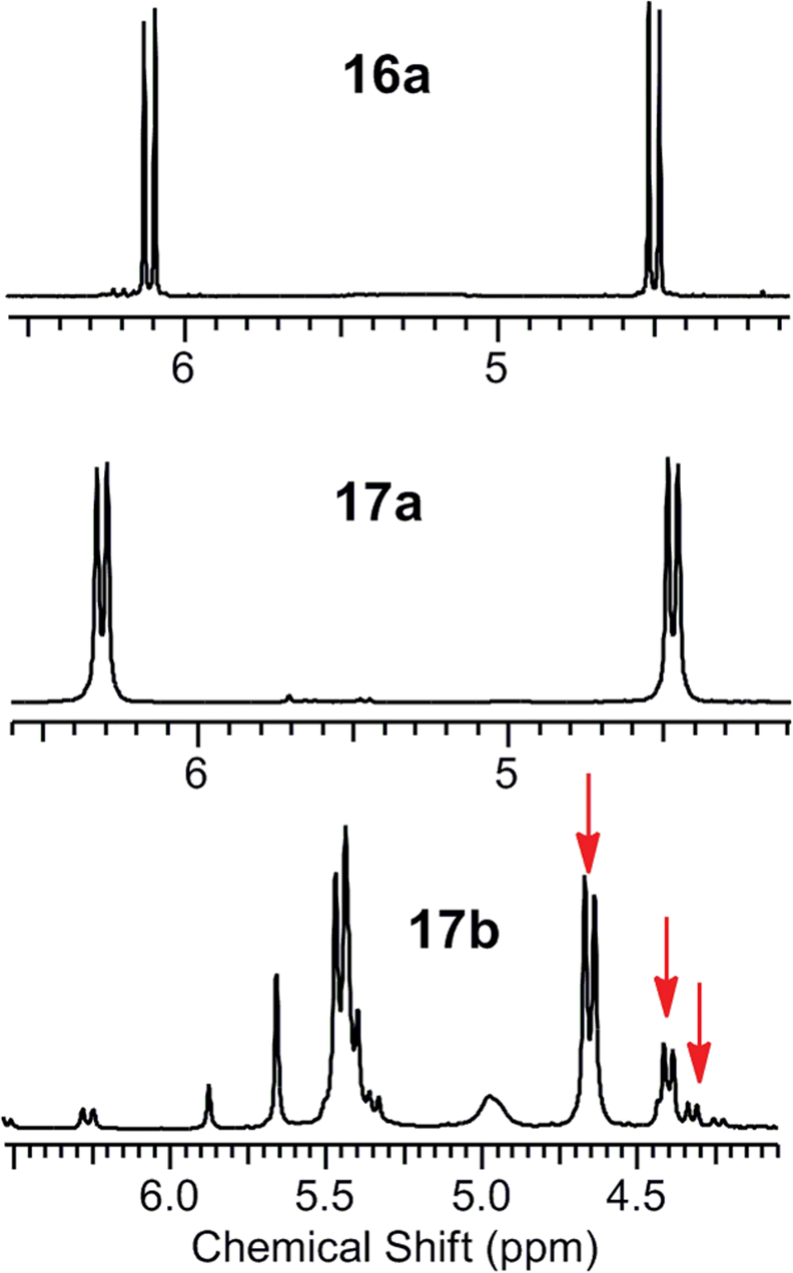
Detail of the ^1^H spectra of compounds 10a, 11a and 11b.

For triazoles **10a** and **11a**, we observed only one set of signals, with no isomery. Surprisingly, the ^1^H spectra of triazole **11b** contained at least three sets of signals (Fig 11), similar to the ^13^C NMR spectra in which more than one set of signals was detected. These results confirm that the type of substituent on the triazole strongly influences the number of isomers in NMR spectra. Moreover, the presence of isomery in **11b** and the lack of isomery in derivative **11a** indicate that the position of the aliphatic part of azocine relative to the triazole substituent is crucial for the number of isomers formed. These results also demonstrate that the presence of amide bonds in the azocine part of the molecule does not affect the isomery observed in the NMR spectra.

Regioisomers **12a** and **12b** were inseparable under several HPLC conditions; the retention times of the isomers were nearly identical on semi-preparative C_18_ columns. The ^1^H NMR spectrum of the mixture of **12a,b** in CDCl_3_ revealed the presence of additional isomers, similar to compounds **8a,b** and **9a,b**. Thus, all possible isomers can also be expected when labeling oligonucleotides *via* nucleobase derivatization.

To clearly identify the origin of the isomery, triazoles **8a,b**, **9a,b** and **11a,b** were subjected to computational study.

### Computational study

**Fig 12:**
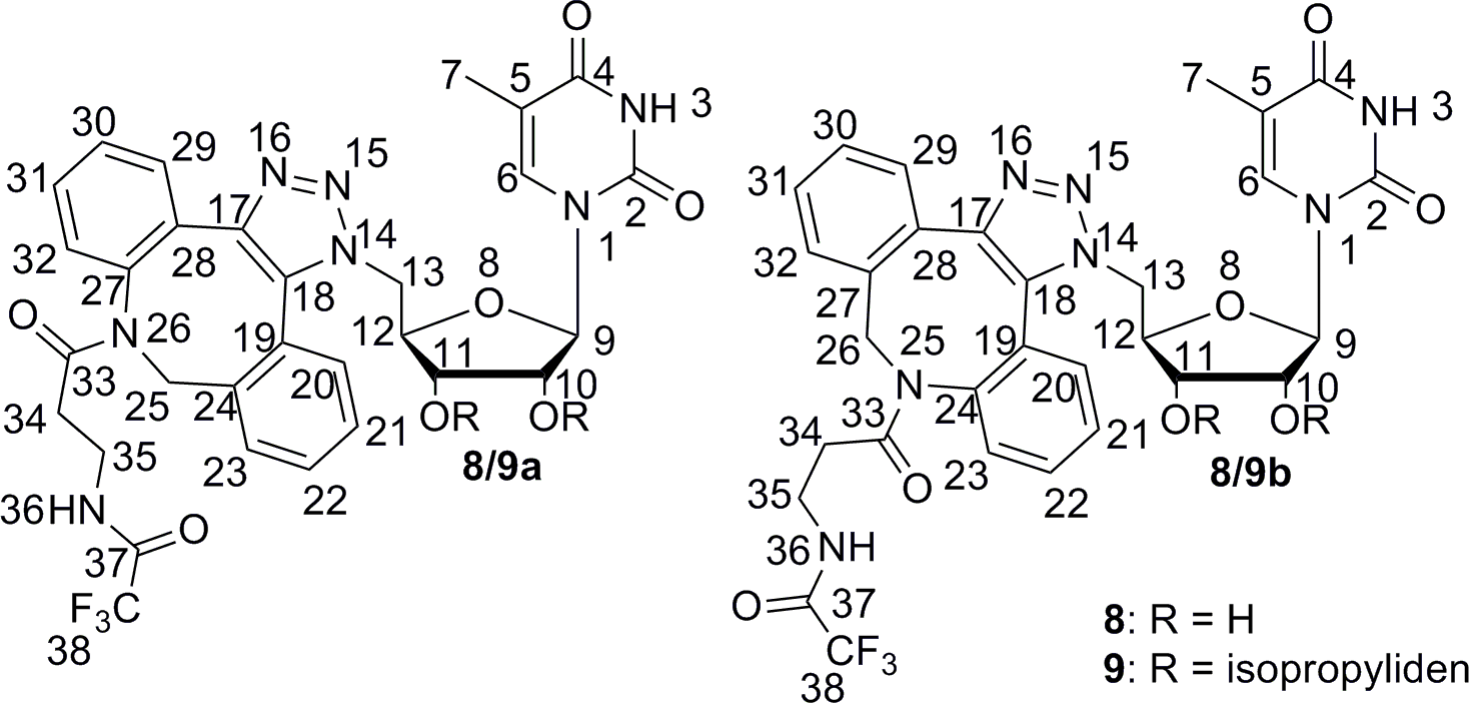
Numbering of atoms in 8a,b and 9a,b in the conformational study.

According to the NMR study described above, we assumed that the observed conformations of compounds **8a,b** and **9a,b** were the result of a combination of rotation about two bonds between the triazole and ribose rings. The rotations of these bonds were studied as the changes of two dihedral angles involving backbone atoms N^15^N^14^C^13^C^12^ and N^14^C^13^C^12^C^11^ (for numbering see Fig 12).

Although the conformational changes are dependent on the solvent, we simplified the quantum calculation to a vacuum to assess the ability of the compounds to form stable conformers whose distributions further depend on the solvents. The theoretical model used in this study was the B3LYP method with a 6-31G(d,p) basis set in Gaussian 09 [32]. The optimized geometry was determined for all individual structures. Using the optimized structures, the potential energy surface (PES) was scanned with 10-degree increments of rotation, up to a total of 360 degrees for every dihedral angle.

To determine the configurations with energies at the local minima, the dependencies of the energies on both dihedral angles were examined. The conformation with the lowest energy was selected as the zero point on the energy scale for each structure. The potential local energy minima were subsequently determined, and the fractional populations from the Maxwell-Boltzmann distribution were estimated according to the following standard equation at 300 K:

$$\frac{N_{i}}{N}=\frac{\text{exp}\left(-E_{i}/kT\right)}{\sum \text{exp}\left(-E_{j}/kT\right)}.$$

where *N*_*i*_ is the number of molecules in the configuration with the energy *E*_*i*_ of a total number *N* of all molecules at temperature *T* and κ is the Boltzmann constant. The sum in the denominator is over all our configurations with particular energies *E*_*j*_.

**Fig 13:**
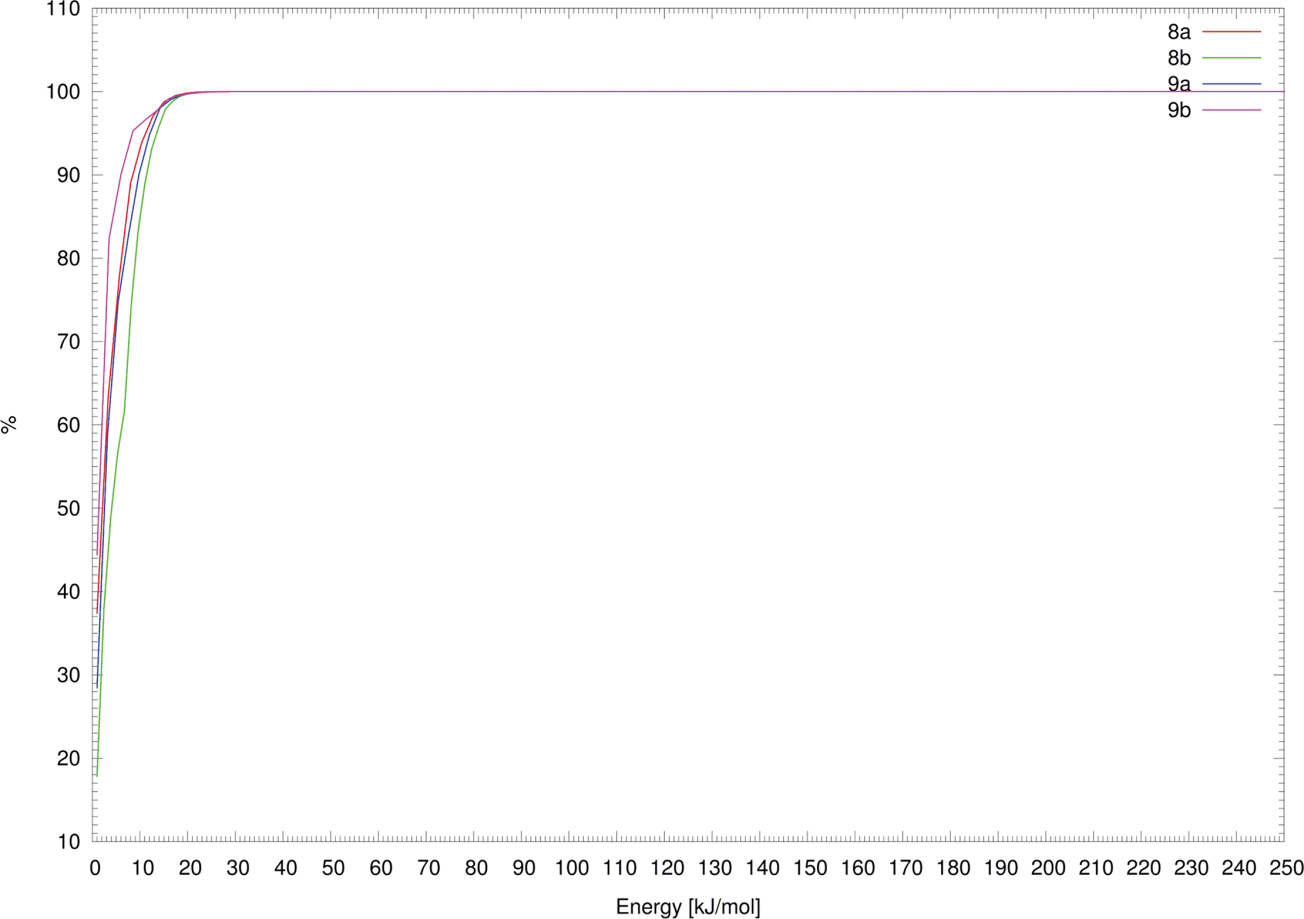
Dependency of the total population on the energy for the appropriate derivatives.

The resulting total populations (the sum of the populations of the conformers with energy lower than or equal to the corresponding energy) in the energy are presented in Fig 13. These dependencies revealed that the most frequent conformers have potential energies lower than 30 kJ/mol.

Compound **8a** formed 20 local minima with energy lower than 30 kJ/mol, and one conformer significantly predominated with a population close to 14% (see Table 2). Compound **8b** formed 16 local minima with energy < 30 kJ/mol, and 12 and 15 local minima satisfied these criteria for derivatives **9a** and **9b**, respectively. The local minima representing conformers with populations greater than 0.5% are summarized in Table 2 (a list of all minima with energy lower than 30 kJ/mol is presented in the S1–S4 Tables).

**Table 2:** Local minima of derivatives 8a, 8b, 9a and 9b with conformer populations greater than 0.5%.

Derivative	Local minima	Energy (kj/mol)	Population (%)
**8a**	1	0.00	13.93
	2	3.64	3.24
	3	5.71	1.41
	4	6.78	0.92
	5	7.84	0.60
**8b**	1	0.00	17.84
	2	5.93	1.66
	3	6.86	1.14
	4	7.08	1.04
	5	7.45	0.90
	6	8.03	0.71
**9a**	1	0.00	13.93
	2	5.47	3.24
	3	6.00	1.41
**9b**	1	0.00	16.40
	2	0.71	12.34

**Fig 14:**
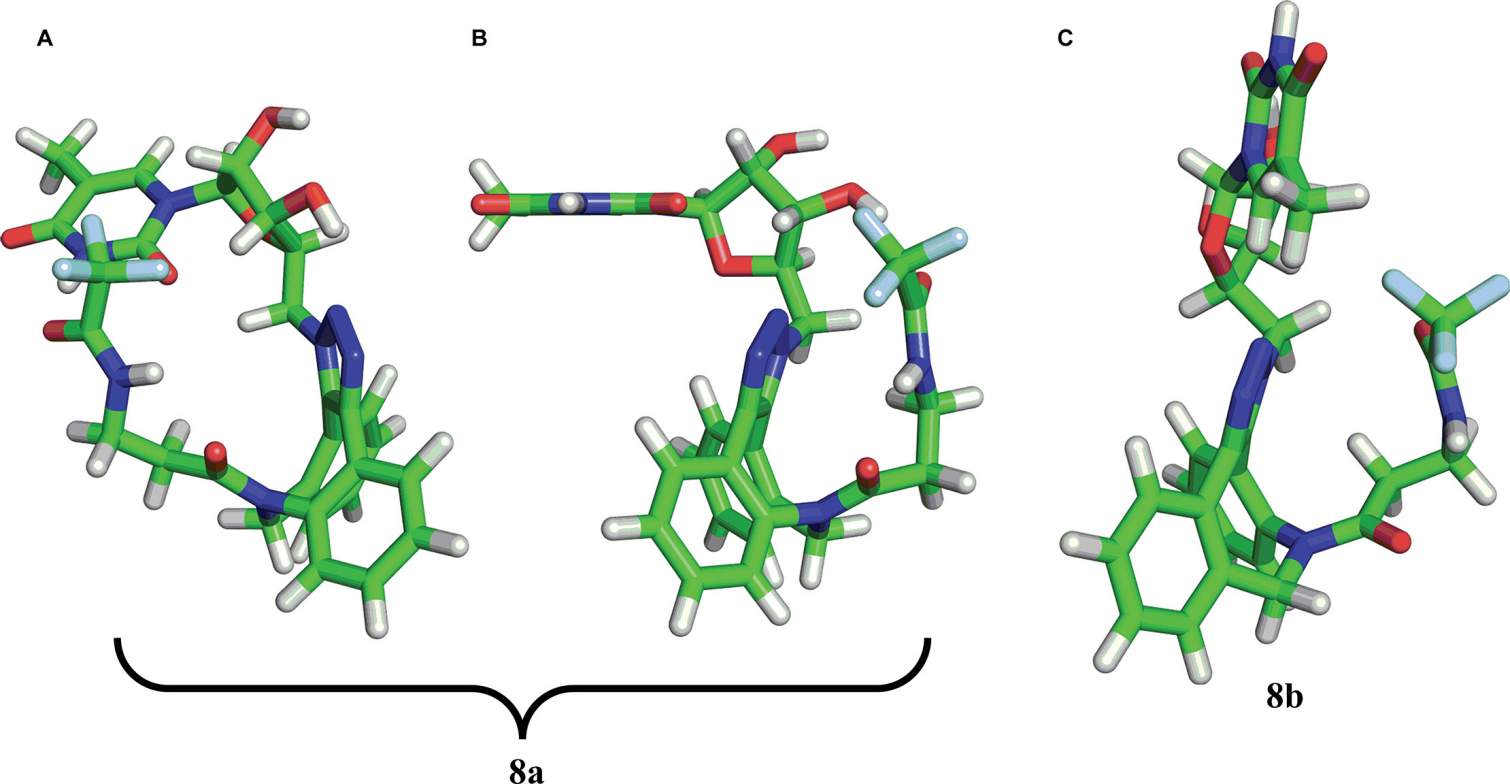
Possible orientation of the aliphatic chain exemplified by structures 8a and 8b. The orientation of the triazole ring plane perpendicular to the plane of the page with the orientation of the bond to 5-methyluridine behind the plane of the page was established as the reference plane in the description of the position of the aliphatic chain in structures **8a** (“left” for A and “right” for B) and **8b** (“right” for C).

Analyses of compounds **8a,b** and **9a,b** with respect to changes in both dihedral angles revealed that the position of the aliphatic chain in structures (a) (intended **8a/9a**) and (b) (intended **8b/9b**) differed. For the (a) structures, both the left and right positions of the aliphatic chain (with respect to the triazole ring–see Fig 14) were observed (Fig 14A and 14B), but the positions on the right site were characteristic for only three local minima, 18, 19 and 20, with higher energies (26.03–26.79 kJ/mol) and a low conformers population (below 0.01%) for derivative **8a**; two local minima—8 (19.94 kJ/mol) and 11 (23.59 kJ/mol)—with populations less than 0.01% were observed for derivative **9a**. However, the (b) structures were characterized by the right position only (Fig 14C).

**Fig 15:**
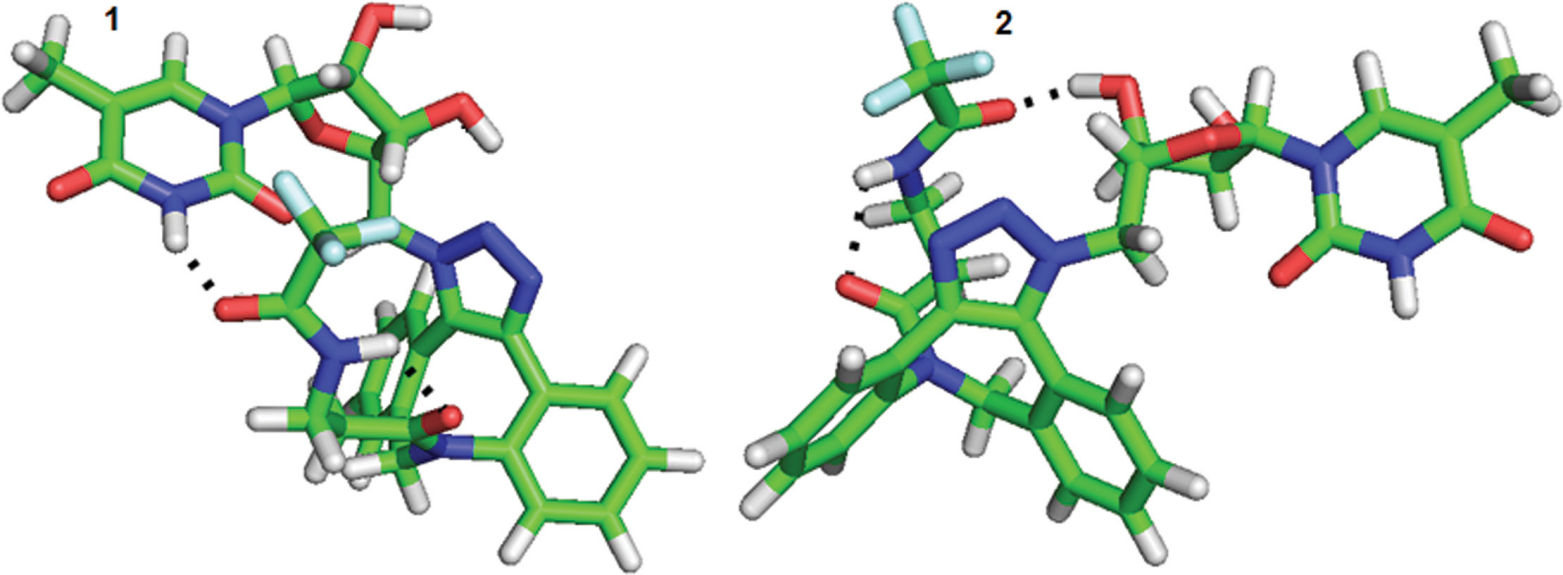
Two conformers 1 and 2 of derivative 8a with different positions of the aliphatic chain.

In addition, the interactions between the 5-methyluridine and the aliphatic chain as well as the distinct intermolecular hydrogen bonds were observed. The most frequent conformer at the first local minima of derivative **8a** (see Table 2) maintained the aliphatic chain in direct interaction with the pyrimidine ring. This interaction was enabled by a hydrogen bond between the carbonyl of trifluoroacetyl group 37 and imide hydrogen 3 of the pyrimidine ring (see Fig 15-1). In the conformer representing the second most populated local minimum, the trifluoroacetyl carbonyl group of the aliphatic chain interacted with the ribose OH hydrogen (see Fig 15-2).

Similar to conformation 2, the other local minima of derivative **8a** summarized in Table 2 exhibited interactions of the trifluoroacetyl group with the ribose OH hydrogens (see S79 Fig).

**Fig 16:**
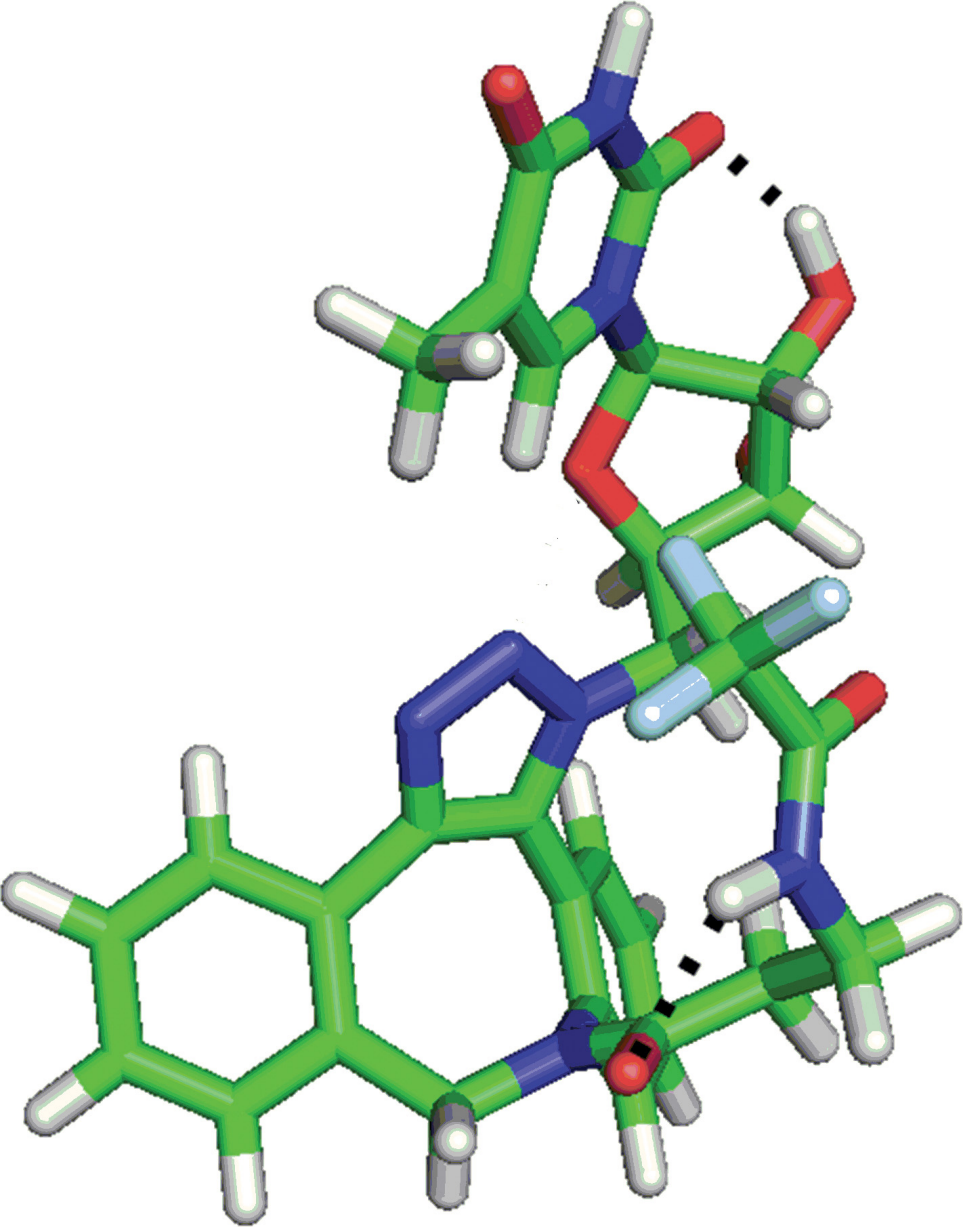
The most abundant conformation of derivative 8b.

Local minimum 1, in which the uracil carbonyl group directly interacts with the ribose hydroxyl group, predominated for derivative **8b** (Fig 16). In local minimum 4, interaction of both ribose OH hydrogens with the trifluoroacetyl carbonyl group was observed (see S80 Fig). Structures at other local minima (2, 3, 5 and 6) were again fixed by hydrogen bonds between the ribose OH hydrogen and uracil carbonyl group (see S80 Fig).

**Fig 17:**
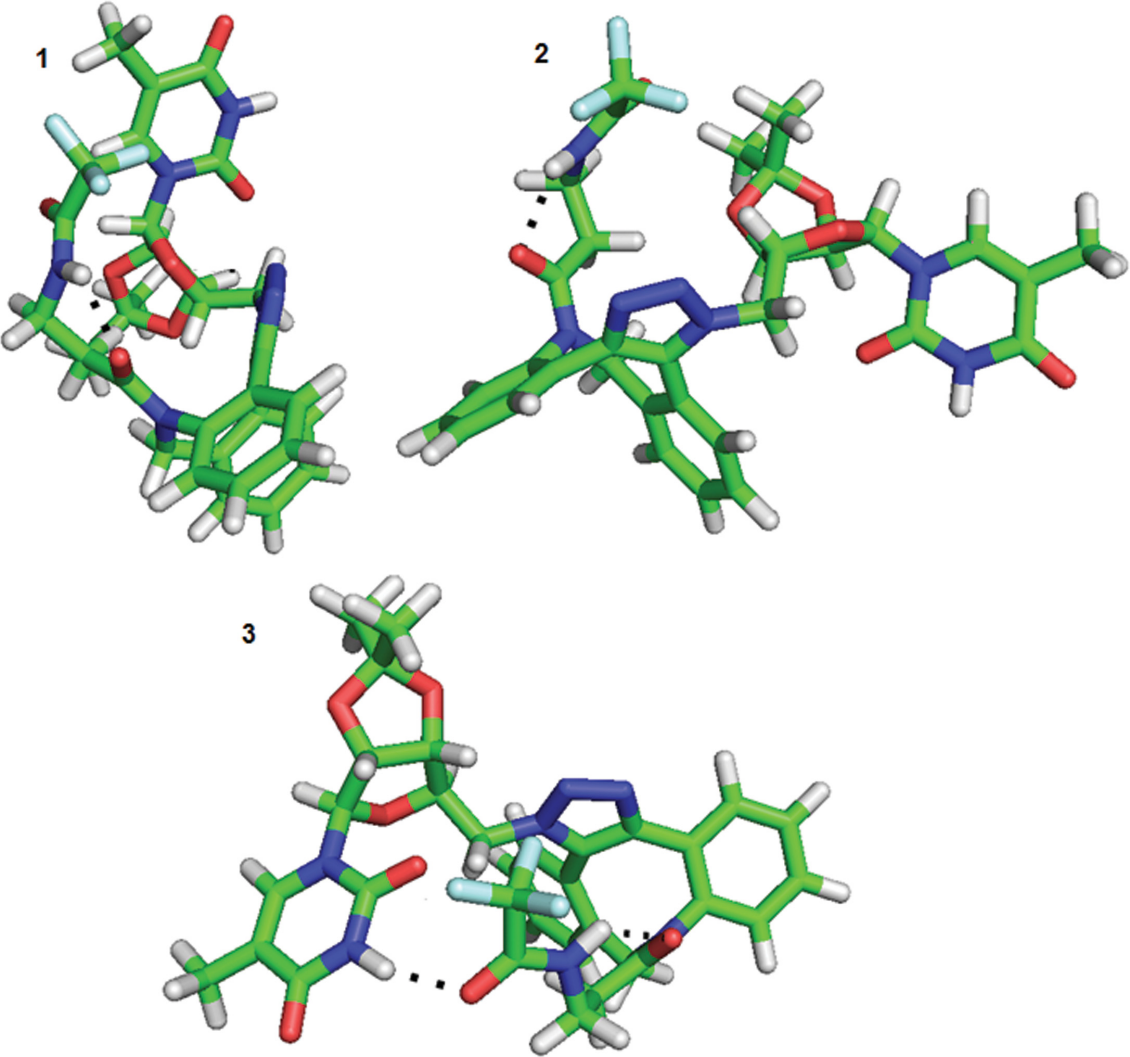
The most abundant conformers of derivative 9a.

In derivative **9a**, in the conformer of the first local minimum, the aliphatic chain was located close to the pyrimidine ring; however, no hydrogen bond was observed between them (Fig 17-1). The conformer with local minimum 2 (Table 2) was characterized by the location of the pyrimidine part of the molecule distant from the aliphatic chain (Fig 17–2). Moreover, in the conformer with local minimum 3 (Table 2), an interaction between the pyrimidine imide group 3 and trifluoroacetyl carbonyl group 37 was detected (Fig 17–3).

**Fig 18:**
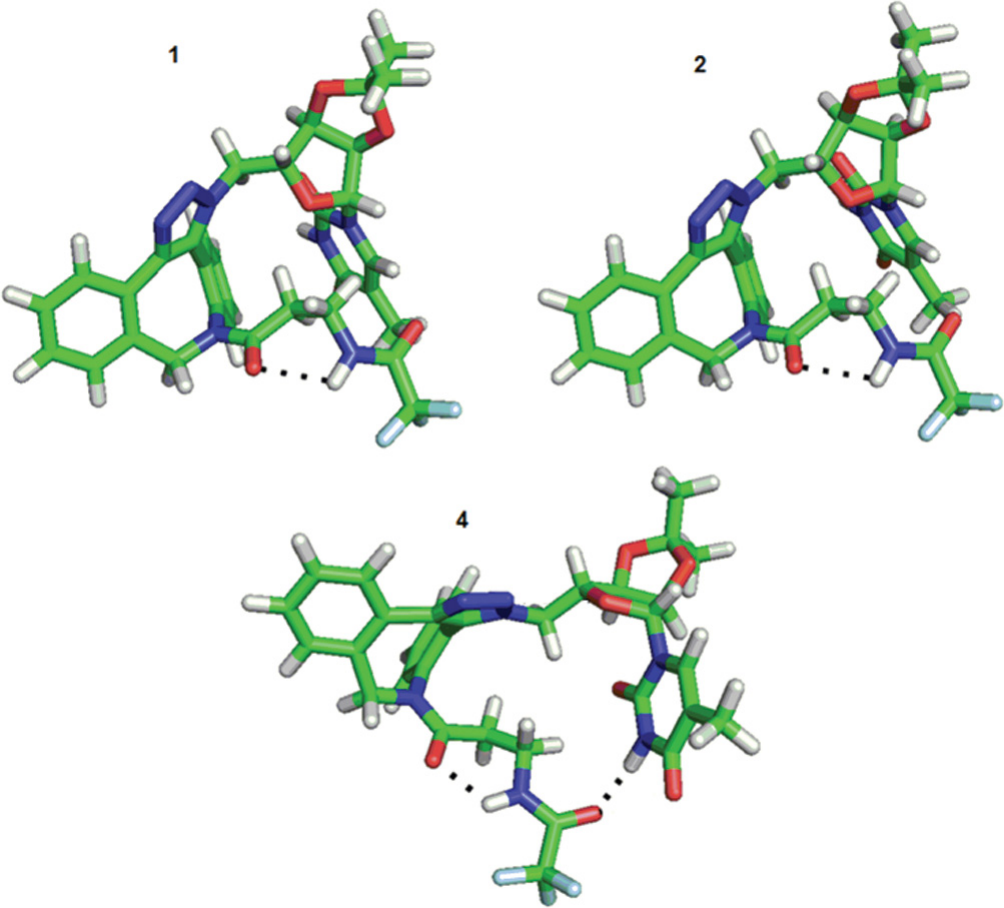
Conformation 1 and 2 of derivative 9b.

The derivative **9b** was characterized by two predominant local minima with a total population of approximately 28% (Table 2). These two local minima, which differed only slightly in their combination of dihedral angles, exhibited a close position of the aliphatic chain and pyrimidine ring, although no hydrogen bond was observed between them (Fig 18–1 and 18–2).

To elucidate the conformational changes and relationship among individual local minima, we determined the pathways of the conformational changes with appropriate energy. The pathways included all local minima of the appropriate derivatives with intrinsic energy lower than or equivalent to 30 kJ/mol (and a few intermediate states with higher energies).

**Fig 19:**
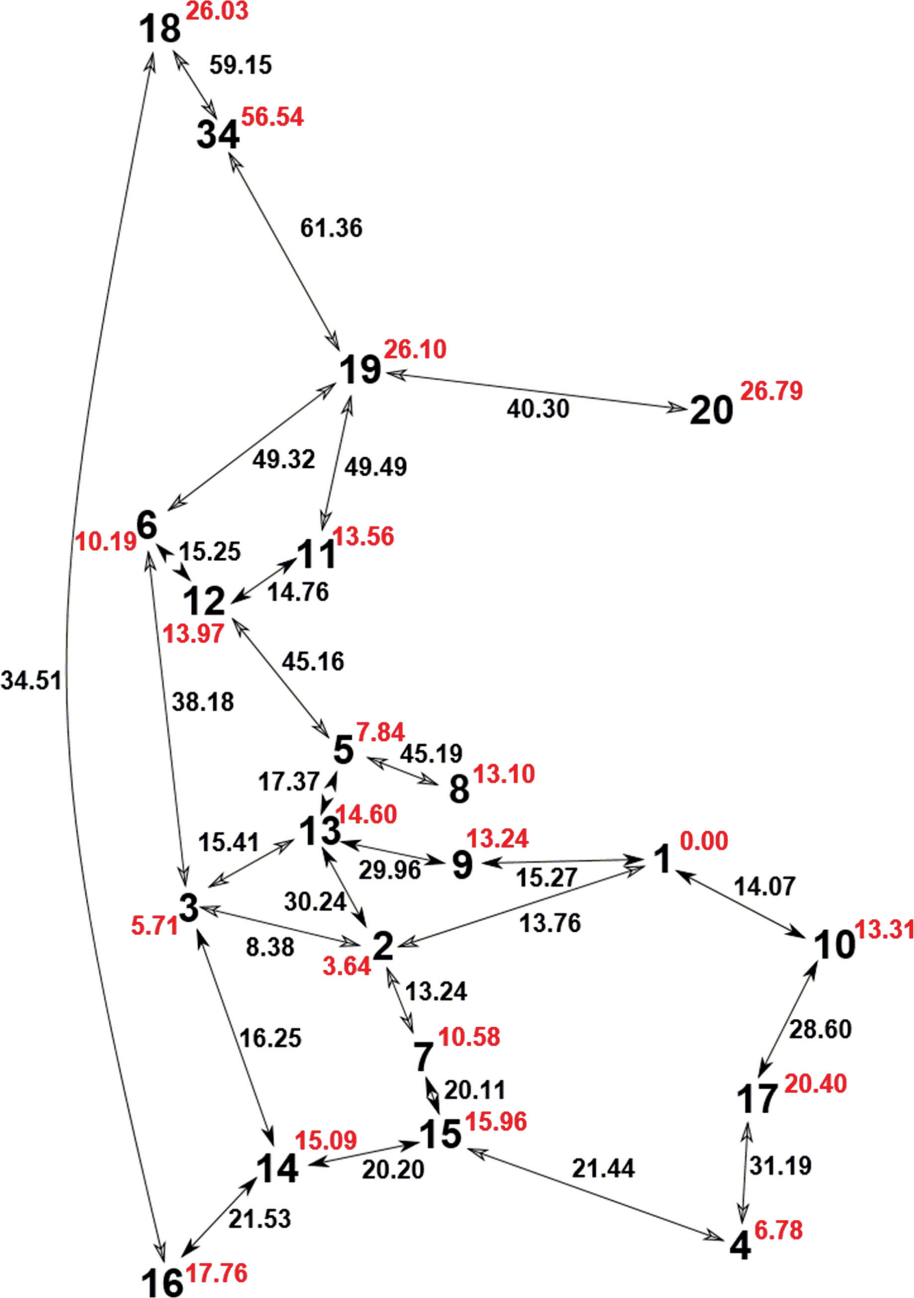
The transitions among the individual local minima of derivative 8a characterized by adequate energies. The red numbers depict the intrinsic energies of individual local minima; black numbers describe the energy barriers for the conversion of local minima.

In derivative **8a,** the transition from local minimum 1 to 2 was connected with energy of 13.76 kJ/mol, whereas other changes required relatively high energy. Although the transition between minima 2 and 3 required low energy, any transition to another local minimum involved overcoming a relatively high energy barrier. Thus, the transition to local minimum 4 and 5 was relatively complicated (see Fig 19).

Identical diagrams were determined for structures **8b**, **9a** and **9b** (see S80, S82 and S83 Figs). Similar to **8a**, the transitions among individual local minima were energetically demanding.

This conformational analysis using quantum mechanical investigations revealed that derivatives **8a**, **8b**, **9a** and **9b** can form more local minima on the PES in which different interactions between the aliphatic chain (bearing a fluorine atom), uracil and ribose were observed, consistent with the differentiation of signals in the ^1^H and ^19^F NMR spectra.

## Conclusions

In summary, an alternative approach to the synthesis of 5- and 5´-azido derivatives of thymidine riboside was developed. These derivatives were successfully converted to 5- and 5´-1,2,3-triazol-1-yl derivatives *via* copper-free click reactions using dibenzoazocine derivative **7**. The NMR spectra of all formed triazoles revealed the presence of conformational isomers., The isomery does not originate from the potentially expected s-cis/s-trans isomery of the amide groups in the aliphatic chain bound to the azocine moiety, what was confirmed by NMR analysis. ^1^H-^15^N HMQC and ^1^H-^15^N HMBC NMR spectra predicted the formation of conformational isomers due to hindered rotation of the pyrimidine and azocine parts of the molecule and ^19^F-^19^F EXSY experiments proved exchange between individual conformers. The computational study of the studied compounds revealed that their isomery is caused either by different positions of the aliphatic chain relative to the nucleoside part of the molecule or by the formation of intramolecular hydrogen bonds. The similar isomery was studied subsequently also for derivatives, in which the nucleoside was replaced by hydrogen (**10a**) or coumarine scaffold (**11a/11b)**. For the substrates **10a** and **11a** no isomery was observed, while for derivative **11b** at least three conformers were detected.

The formation of triazoles on a nucleic acid sequence can distort the nucleobase, resulting in deformation of the nucleic acid structure, which can have a negative effect on imaging processes. However, this distortion could potentially be exploited in various biological applications that are based on the deformation of nucleic acids.

## Experimental Section

LC/MS analyses were performed using UHPLC/MS on a UHPLC chromatograph with a PDA detector and a single quadrupole mass spectrometer; a C18 column was used at 30°C and a flow rate of 600 μl/min. The mobile phase consisted of (A) 0.01 M ammonium acetate in water and (B) acetonitrile, with B linearly programmed to change from 10% to 80% over the course of 2.5 min and then to maintain this concentration for 1.5 min. The column was re-equilibrated at 10% B for 1 min. APCI ionization was operated with a discharge current of 5 μA, vaporizer temperature of 350°C and a capillary temperature of 200°C.

Purification was performed by semi-preparative HPLC on a reverse-phase C18 column, 20 x 100 mm, with 5-μm particles. The mobile phase consisted of acetonitrile and a 10 mM aqueous ammonium acetate gradient over 6 min.

NMR spectra were measured in DMSO-*d6* or CDCl_3_ on 500 MHz and 400 MHz spectrometers. Chemical shifts (δ are reported in parts per million (ppm), and coupling constants (*J*) are reported in Hertz (Hz). Acetate salts exhibited a singlet at 1.7–1.9 ppm in ^1^H NMR spectra and two resonances at 173 and 23 ppm in ^13^C spectra.

HRMS analysis was performed using a high-resolution mass spectrometer operating in positive full-scan mode (120 000 FWMH) in the range of 200–900 m/z. The settings for electrospray ionization were as follows: oven temperature of 300°C, sheath gas of 8 arb. units and source voltage of 1.5 kV. The acquired data were internally calibrated with diisooctyl phthalate as a contaminant in methanol (m/z 391.2843). Samples were diluted to a final concentration of 20 μmol/l with 0.1% formic acid in water and methanol (50:50, v/v). The samples were injected by direct infusion into the mass spectrometer.

1-(-6-(Hydroxymethyl)-2,2-dimethyltetrahydrofuro[3,4-d][1,3]dioxol-4-yl)-5-methylpyrimidine-2,4(1H,3H)-dione **2** [28] and 5-hydroxymethylene-uracil [33] were prepared as described in the literature.

### Compound synthesis

#### 1-(3,4-Dihydroxy-5-iodomethyl-tetrahydrofuran-2-yl)-5-methyl-1*H*-pyrimidin-2,4-dione (3a)

5-Methyluridine **1** (1.18 g, 4.57 mmol), PPh_3_ (1.85 g, 7.05 mmol) and imidazole (479 mg, 7.04 mmol) were dissolved in anhydrous THF (30 ml). Then, a solution of iodine (1.28 g, 5.04 mmol) in anhydrous THF (15 ml) was added. The reaction mixture was stirred at room temperature for 2 hours. The white precipitate was removed by filtration, and the THF was evaporated under reduced pressure. The resulting brown solid was purified on a silica gel column with a mobile phase of CHCl_3_/MeOH (6:1 v/v). Yellow solid, 1.19 g (70%).

^1^H NMR (400 MHz, DMSO-*d*_6_) δ ppm 1.80 (s, 3 H), 3.40 (dd, *J* = 10.52, 6.58 Hz, 1 H), 3.57 (dd, *J* = 10.52, 6.14 Hz, 1 H), 3.83 (td, *J* = 6.25, 3.73 Hz, 1 H), 3.90 (dd, *J* = 5.48, 3.73 Hz, 1 H), 4.20 (t, *J* = 5.92 Hz, 1 H), 5.81 (d, *J* = 6.14 Hz, 1 H), 7.52 (d, *J* = 0.88 Hz, 1 H), 11.37 (s, 1 H); ^13^C NMR (101 MHz, DMSO-*d*_6_) δ ppm 7.98, 12.29, 72.17, 73.01, 83.21, 88.09, 110.14, 136.75, 151.01, 163.86. HRMS m/z calculated for C_10_H_14_IN_2_O_5_ [M+H]^+^ 368.9942, found 368.9941.

#### 1-(3,4-Dihydroxy-5-bromomethyl-tetrahydrofuran-2-yl)-5-methyl-1*H*-pyrimidin-2,4-dione (3b)

5-Methyluridine **1** (0.5 g, 1.94 mmol), PPh_3_ (0.78 g, 2.98 mmol) and imidazole (200 mg, 2.98 mmol) were dissolved in anhydrous THF (10 ml), and bromine was added (0.11 ml, 2.13 mmol). The reaction mixture was stirred at room temperature for 2 hours. The white precipitate was removed by filtration, and the THF was evaporated under reduced pressure. The resulting brown solid was purified on a silica gel column with a mobile phase of CHCl_3_/MeOH (6:1 v/v). White solid 0.42 g (68%).

^1^H NMR (400 MHz, DMSO-*d*_6_) δ ppm 1.76–1.83 (m, 3 H), 3.67 (dd, *J* = 10.74, 5.92 Hz, 1 H), 3.76–3.84 (m, 1 H), 3.96 (dq, *J* = 8.50, 4.48 Hz, 2 H), 4.18 (t, *J* = 5.70 Hz, 1 H), 5.81 (d, *J* = 6.14 Hz, 1 H), 7.51 (d, *J* = 0.88 Hz, 1 H), 11.37 (s, 1 H); ^13^C NMR (101 MHz, DMSO-*d*_6_) δ ppm 12.11, 33.82, 71.63, 71.92, 82.75, 87.78, 109.94, 136.39, 150.81, 163.66. HRMS m/z calculated for C_10_H_14_BrN_2_O_5_ [M+H]^+^ 321.0081, found 321.0080.

#### 5´-Deoxy-5´-iodo-5-methyl-2´,3´-*O*-isopropyliden-uridine (4a)

Compound **2** [28] (1.27 g, 4.26 mmol), PPh_3_ (1.71 g, 6.52 mmol) and imidazole (448 mg, 6.58 mmol) were dissolved in anhydrous THF (25 ml). Then, a solution of iodine (1.19 g, 4.69 mmol) in anhydrous THF (15 ml) was added. The reaction mixture was stirred at room temperature for 2 hours. The white precipitate was removed by filtration, and the THF was evaporated under reduced pressure. The resulting brown solid was purified on a silica gel column with a mobile phase of Tol/AcN (5:2 v/v). Yellow solid 1.31 g (75%).

^1^H NMR (400 MHz, DMSO-*d*_6_) δ ppm 1.29 (s, 3 H), 1.48 (s, 3 H), 1.77 (s, 3 H), 3.36–3.40 (m, 1 H), 3.48 (dd, *J* = 10.10, 8.00 Hz, 1 H), 4.12 (ddd, *J* = 7.56, 6.03, 3.95 Hz, 1 H), 4.73 (dd, *J* = 6.58, 3.95 Hz, 1 H), 5.08 (dd, *J* = 6.58, 2.19 Hz, 1 H), 5.80 (d, *J* = 2.19 Hz, 1 H), 7.60 (d, *J* = 1.32 Hz, 1 H), 11.45 (s, 1 H); ^13^C NMR (101 MHz, DMSO-*d*_6_) δ ppm 6.68, 12.17, 25.28, 27.06, 83.66, 83.90, 86.29, 92.61, 109.91, 113.61, 138.90, 150.49, 164.04. HRMS m/z calculated for C_13_H_18_IN_2_O_5_ [M+H]^+^ 409.0255, found 409.0253.

#### 5´-Bromo-5´-deoxy-5-methyl-2´,3´-*O*-isopropyliden-uridine (4b)

Compound **2** [28] (1.23 g, 4.12 mmol), PPh_3_ (1.66 g, 6.33 mmol) and imidazole (432 mg, 6.35 mmol) were dissolved in anhydrous THF (40 ml), and bromine (0.24 ml, 4.56 mmol) was added. The reaction mixture was stirred at room temperature for 2 hours. The white precipitate was removed by filtration, and the THF was evaporated under reduced pressure. The resulting solid was purified on a silica gel column with a mobile phase of Tol/AcN (5:2 v/v). White solid 0.94 g (63%).

^1^H NMR (400 MHz, DMSO-*d*_6_) δ ppm 1.29 (s, 3 H), 1.48 (s, 3 H), 1.77 (d, *J* = 1.32 Hz, 3 H), 3.63 (dd, *J* = 10.50, 6.10 Hz, 1 H), 3.73 (dd, *J* = 10.00, 7.00 Hz, 1 H), 4.19 (td, *J* = 6.47, 3.73 Hz, 1 H), 4.79 (dd, *J* = 6.58, 3.95 Hz, 1 H), 5.08 (dd, *J* = 6.58, 2.19 Hz, 1 H), 5.79 (d, *J* = 2.63 Hz, 1 H), 7.59 (s, 1 H), 11.45 (s, 1 H); ^13^C NMR (101 MHz, DMSO-*d*_6_) δ ppm 11.98, 25.06, 26.86, 32.87, 82.56, 83.51, 85.74, 92.47, 109.71, 113.44, 138.67, 150.32, 163.85. HRMS m/z calculated for C_13_H_18_BrN_2_O_5_ [M+H]^+^ 361.0394, found 361.0394.

#### 1-(5-(Azidomethyl)-3,4-dihydroxytetrahydrofuran-2-yl)-5-methylpyrimidine-2,4(1*H*,3*H*)-dione (5)

Compound **3a** (1 g, 2.72 mmol) or **3b** (850 mg, 2.72 mmol) was dissolved in anhydrous DMF (15 ml), and sodium azide (555 mg, 8.5 mmol) was added. The suspension was stirred at 90°C for 2 hours. The remaining sodium azide was removed by filtration. Water (15 ml) was added, and the mixture was extracted with ethyl acetate (4x25 ml). The organic layer was dried over sodium sulfate, and the solvent was evaporated. The product was dried with use of freeze dryer. Yield 620 mg (81%).

^1^H NMR (400 MHz, DMSO-*d*_6_) δ ppm 1.79 (s, 3 H), 3.58 (d, *J* = 4.82 Hz, 1 H), 3.88–3.97 (m, 2 H), 4.11–4.19 (m, 1 H), 5.28 (br. s., 1 H), 5.45 (d, *J* = 4.39 Hz, 1 H), 5.78 (d, *J* = 5.70 Hz, 1 H), 7.51 (s, 1 H), 11.37 (s, 1 H); ^13^C NMR (101 MHz, DMSO-*d*_6_) δ ppm 12.05, 51.68, 70.39, 72.03, 82.17, 88.30, 109.85, 136.53, 150.78, 163.70. HRMS m/z calculated for C_10_H_14_N_5_O_5_ [M+H]^+^ 284.0989, found 284.0987.

#### 5´-Azido-5´-deoxy-5-methyl-2´,3´-*O*-isopropyliden-uridine (6)

Compound **4a** (1.3 g, 3.18 mmol) or **4b** (1.14 g, 3.18 mmol) was dissolved in anhydrous DMF (20 ml), and sodium azide (0.65 g, 10 mmol) was added. The suspension was stirred at 90°C for 2 hours. The remaining sodium azide was removed by filtration, and DMF was removed by lyophilization. The solid was then suspended in water (20 ml), and the product was obtained as a white precipitate by filtration. Yield 0.83 g (82%).

^1^H NMR (400 MHz, DMSO-*d*_6_) δ ppm 1.29 (s, 3 H), 1.48 (s, 3 H), 1.77 (s, 3 H), 3.58 (d, *J* = 5.70 Hz, 2 H), 4.12 (q, *J* = 4.80 Hz, 1 H), 4.76 (dd, *J* = 6.58, 4.39 Hz, 1 H), 5.06 (dd, *J* = 6.58, 2.19 Hz, 1 H), 5.80 (d, *J* = 2.63 Hz, 1 H), 7.57 (s, 1 H), 11.43 (br. s., 1 H); ^13^C NMR (101 MHz, DMSO-*d*_6_) δ ppm 11.96, 25.14, 26.92, 51.53, 80.97, 83.13, 84.49, 91.79, 109.71, 113.55, 138.41, 150.31, 163.82. HRMS m/z calculated for C_13_H_18_N_5_O_5_ [M+H]^+^ 324.1302, found 324.1303.

#### N-(3-(1-((-3,4-Dihydroxy-5-(5-methyl-2,4-dioxo-3,4-dihydropyrimidin-1(2H)-yl)tetrahydrofuran-2-yl)methyl)-1H-dibenzo[b,f][1,2,3]triazolo[4,5-d]azocin-8(9H)-yl)-3-oxopropyl)-2,2,2-trifluoroacetamide (8a) N-(3-(3-((-3,4-Dihydroxy-5-(5-methyl-2,4-dioxo-3,4-dihydropyrimidin-1(2H)-yl)tetrahydrofuran-2-yl)methyl)-3H-dibenzo[b,f][1,2,3]triazolo[4,5-d]azocin-8(9H)-yl)-3-oxopropyl)-2,2,2-trifluoroacetamide (8b)

Azido derivative **5** (50 mg, 0.18 mmol) was dissolved in MeOH (2 ml), and dibenzoazocine **7** (93 mg, 0.25 mmol) was added. The solution was stirred at room temperature for 5 minutes, diluted with MeOH to 8 ml and then purified by semi-preparative HPLC. The mobile phase consisted of (A) 0.01 M ammonium acetate in water and (B) acetonitrile, with B linearly programmed to change from 20% to 50% over the course of 6 min. The derivatives **8a** and **8b** were collected separately. Derivative **8a** was the first compound and was isolated as a white solid, 44 mg (35%). Derivative **8b** was the second compound and was isolated as a white solid, 38 mg (30%).

#### 8a

^1^H NMR (500 MHz, CDCl_3_-*d*) δ ppm 1.77 (dt, *J* = 17.0, 5.60 Hz 1 H), 1.83 (s, 3 H), 2.08 (dt, *J* = 17.0, 5.60 Hz 1 H), 3.24 (m, 2 H), 4.02 (t, *J* = 5.50 Hz 1 H), 4.08 (q, *J* = 5.50 1 H), 4.18 (t, *J* = 4.60 Hz 1 H), 4.32 (d, *J* = 16.60 Hz 1 H), 4.71 (dd, *J* = 13.80, 5.20 Hz 1 H), 4.95 (dd, *J* = 13.80, 4.50 Hz 1 H), 5.61 (d, *J* = 3.70 Hz 1 H), 6.03 (d, *J* = 16.60 Hz 1 H), 7.05 (d, *J* = 7.0 Hz 1 H), 7.14 (s, 1 H), 7.18 (m, 1 H), 7.26 (m, 1 H), 7.28 (m, 1 H), 7.35 (tt, *J* = 6.70, 2.0 Hz 1 H), 7.46 (m, 1 H), 7.47 (m, 1 H), 7.65 (dd, *J* = 6.70, 2.0 Hz 1 H), 7.82 (t, *J* = 5.50 Hz 1 H); ^13^C NMR (126 MHz, CDCl_3_) δ ppm 12.31, 33.31, 35.39, 50.22, 51.32, 70.72, 73.37, 82.12, 92.05, 111.05, 115.79 (q, J = 287.9), 124.32, 127.33, 127.44, 127.77, 129.82, 129.86, 129.92, 131.05, 131.10, 131.13, 131.83, 135.11, 135.77, 137.36, 139.27, 142.97, 150.78, 157.17 (q, *J* = 36.5), 164.32, 171.86. HRMS m/z calculated for C_30_H_29_F_3_N_7_O_7_ [M+H]^+^ 656.2075, found 656.2072.

#### 8b

Because it was not possible to identify the major ^1^H and ^13^C NMR signals, characterization was carried out for mixture of all conformers.

^1^H NMR (500 MHz, DMSO-*d*_6_) δ ppm ^1^H NMR (500 MHz, DMSO-*d*_6_) 1.60–1.63 (m, 2 H) 1.65–1.69 (m, 1 H) 1.81 (d, *J* = 0.86 Hz, 3 H) 1.84 (s, 1 H) 1.87 (s, 1 H) 2.03–2.11 (m, 2 H) 2.15–2.25 (m, 1 H) 2.35–2.37 (m, 1 H) 2.47 (quin, *J* = 1.86 Hz, 1 H) 2.51–2.53 (m, 1 H) 2.62–2.65 (m, 1 H) 2.70–2.80 (m, 1 H) 2.87–2.94 (m, 1 H) 2.98–3.05 (m, 1 H) 3.08 (dt, *J* = 13.53, 6.55 Hz, 1 H) 3.13–3.18 (m, 1 H) 3.19–3.27 (m, 2 H) 4.03 (t, *J* = 4.44 Hz, 1 H) 4.26 (br. s., 2 H) 4.28–4.32 (m, 1 H) 4.35–4.40 (m, 2 H) 4.41–4.45 (m, 1 H) 4.46–4.51 (m, 2 H) 4.52–4.57 (m, 1 H) 4.65 (d, *J* = 4.87 Hz, 1 H) 4.78–4.83 (m, 1 H) 4.94–5.00 (m, 1 H) 5.36–5.49 (m, 2 H) 5.51–5.59 (m, 2 H) 5.74 (d, *J* = 3.44 Hz, 2 H) 5.78 (d, *J* = 4.30 Hz, 1 H) 5.81–5.86 (m, 1 H) 5.88 (s, 1 H) 7.07–7.10 (m, 1 H) 7.25–7.32 (m, 5 H) 7.37 (ddd, *J* = 7.73, 6.73, 2.15 Hz, 1 H) 7.40–7.43 (m, 2 H) 7.52–7.54 (m, 2 H) 7.55–7.59 (m, 1 H) 7.59–7.63 (m, 1 H) 7.63–7.68 (m, 3 H) 7.73–7.76 (m, 1 H) 9.21–9.27 (m, 1 H) 9.28–9.37 (m, 1 H) 10.96–11.21 (m, 1 H); ^13^C NMR (101 MHz, DMSO-*d*_6_) δ ppm 11.95, 12.05, 31.35, 31.85, 32.08, 35.46, 35.51, 49.44, 49.78, 51.74, 51.87, 54.29, 70.77, 70.86, 71.87, 72.16, 80.85, 81.87, 89.07, 90.20, 109.86, 110.05, 114.59, 116.88, 126.46, 126.52, 126.83, 126.95, 127.61, 127.99, 128.05, 128.08, 128.22, 128.41, 128.66, 128.81, 129.00, 129.07, 129.13, 129.36, 129.43, 130.15, 130.42, 131.52, 131.63, 132.01, 132.23, 132.30, 132.73, 133.72, 133.80, 136.09, 137.05, 140.81, 140.86, 143.85, 143.91, 150.50, 150.58, 150.65, 155.94, 156.02, 156.21, 156.31, 163.49, 163.73, 163.77, 168.70, 168.84. HRMS m/z calculated for C_30_H_29_F_3_N_7_O_7_ [M+H]^+^ 656.2075, found 656.2072.

#### N-(3-(1-((2,2-Dimethyl-6-(5-methyl-2,4-dioxo-3,4-dihydropyrimidin-1(2H)-yl)tetrahydrofuro[3,4-d][1,3]dioxol-4-yl)methyl)-1H-dibenzo[b,f][1,2,3]triazolo[4,5-d]azocin-8(9H)-yl)-3-oxopropyl)-2,2,2-trifluoroacetamide (9a) N-(3-(3-((2,2-Dimethyl-6-(5-methyl-2,4-dioxo-3,4-dihydropyrimidin-1(2H)-yl)tetrahydrofuro[3,4-d][1,3]dioxol-4-yl)methyl)-3H-dibenzo[b,f][1,2,3]triazolo[4,5-d]azocin-8(9H)-yl)-3-oxopropyl)-2,2,2-trifluoroacetamide (9b)

Azido derivative **6** (50 mg, 0.15 mmol) was dissolved in MeOH (2 ml), and dibenzoazocine **7** (78 mg, 0.21 mmol) was added. The solution was stirred at room temperature for 5 minutes, diluted with MeOH to 8 ml and then purified by semi-preparative HPLC. The mobile phase consisted of (A) 0.01 M ammonium acetate in water and (B) acetonitrile, with B linearly programmed to change from 30% to 60% over the course of 6 min. Derivatives **9a** and **9b** were collected separately. Derivative **9a** was the first compound and was isolated as a white solid, 31 mg (30%). Derivative **9b** was the second compound and was isolated as a white solid, 24 mg (23%).

#### 9a

^1^H NMR (500 MHz, CDCl_3_-*d*) δ ppm 1.30 (s, 3 H), 1.48 (s, 3 H), 1.80 (m, 1 H), 1.96 (d, *J* = 1.40 Hz 3 H), 2.00 (m, 1 H), 3.22 (m, 1 H), 3.32 (m, 1 H), 4.19 (d, *J* = 16.5 Hz 1 H), 4.53 (dt, *J* = 9.90, 3.30 Hz 1 H), 4.79 (dd, *J* = 13.80, 2.60 Hz 1 H), 4.85 (dd, *J =* 13.80, 2.60 Hz 1 H), 4.91 (dd, *J* = 6.50, 3.40 Hz 1 H), 5.02 (d, *J* = 6.30 1 H), 5.27 (m, 1 H), 5.75 (d, *J* = 16.5 Hz 1 H), 6.95 (m, 1 H), 7.03 (m, 1 H), 7.16 (s, 1 H), 7.25 (m, 1 H), 7.26 (m, 2 H), 7.38 (t, *J* = 5.70 Hz 1 H), 7.44 (m, 2 H), 7.65 (dd, *J* = 5.90, 3.40 Hz 1 H), 8.34 (br. s., 1 H); ^13^C NMR (126 MHz, CDCl_3_) δ ppm 12.31, 33.31, 35.39, 50.22, 51.32, 70.72, 73.37, 82.12, 92.05, 111.05, 115.79 (q, *J* = 287.9), 124.32, 127.33, 127.44, 127.77, 129.82, 129.86, 129.92, 131.05, 131.10, 131.13, 131.83, 135.11, 135.77, 137.36, 139.27, 142.97, 150.78, 157.17 (q, *J* = 36.5), 164.32, 171.86. HRMS m/z calculated for C_33_H_33_F_3_N_7_O_7_ 696.2388, found 696.2385.

#### 9b

Because it was not possible to identify the major ^1^H and ^13^C NMR signals, characterization was carried out for mixture of all conformers.

^1^H NMR (500 MHz, CDCl_3_-*d*) δ ppm 1.33–1.39 (m, 9 H), 1.51 (s, 1 H), 1.53–1.57 (m, 7 H), 1.71 (ddd, *J* = 17.18, 6.87, 4.01 Hz, 1 H), 1.81–1.86 (m, 8 H), 1.87 (dd, *J* = 4.58, 2.86 Hz, 1 H), 1.91 (s, 1 H), 1.98 (dd, *J* = 7.45, 3.44 Hz, 1 H), 2.00 (s, 1 H), 2.01–2.03 (m, 1 H), 2.09 (s, 1 H), 2.16 (ddd, *J* = 17.18, 7.45, 4.58 Hz, 2 H), 2.28–2.36 (m, 1 H), 2.65–2.75 (m, 1 H), 3.18–3.25 (m, 1 H), 3.26–3.49 (m, 6 H), 4.40 (dd, *J* = 16.90, 4.30 Hz, 2 H), 4.57–4.67 (m, 2 H), 4.68–4.73 (m, 4 H), 4.79–4.83 (m, 1 H), 4.85–4.95 (m, 2 H), 4.99–5.05 (m, 1 H), 5.09 (dd, *J* = 6.59, 2.00 Hz, 1 H), 5.11–5.15 (m, 1 H), 5.16 (dd, *J* = 6.30, 3.44 Hz, 1 H), 5.23 (dd, *J* = 6.30, 3.44 Hz, 1 H), 5.35 (br. s., 1 H), 5.37–5.43 (m, 1 H), 5.53 (d, *J* = 1.15 Hz, 1 H), 5.57 (d, *J* = 1.72 Hz, 1 H), 5.65 (d, *J* = 1.72 Hz, 1 H), 5.81 (d, *J* = 16.61 Hz, 1 H), 6.03 (d, *J* = 17.18 Hz, 1 H), 6.88–6.92 (m, 2 H), 7.06–7.08 (m, 1 H), 7.09–7.13 (m, 1 H), 7.13–7.18 (m, 2 H), 7.22–7.27 (m, 4 H), 7.29–7.34 (m, 3 H), 7.35 (s, 1 H), 7.36–7.41 (m, 5 H), 7.41–7.46 (m, 3 H), 7.46–7.51 (m, 3 H), 7.51–7.56 (m, 2 H), 7.58–7.68 (m, 3 H), 9.12 (s, 1 H), 9.32 (s, 1 H), 9.41 (s, 1 H), 9.66 (s, 1 H); ^13^C NMR (126 MHz, CDCl_3_-*d*) δ ppm 11.85, 12.02, 12.18, 25.14, 25.25, 25.45, 26.86, 26.91, 27.01, 27.09, 31.95, 32.06, 33.19, 34.82, 35.03, 35.19, 35.34, 49.57, 49.93, 50.40, 50.69, 52.81, 53.34, 55.08, 55.13, 82.27, 82.71, 82.84, 84.00, 84.10, 84.38, 85.43, 85.72, 85.90, 87.30, 95.05, 95.94, 96.32, 96.50, 111.02, 111.18, 111.88, 112.28, 114.36, 114.58, 114.67, 114.92, 116.87, 126.81, 127.09, 127.22, 127.35, 127.39, 127.43, 127.50, 127.59, 128.03, 128.09, 128.19, 128.34, 128.45, 128.61, 128.71, 128.86, 129.02, 129.07, 129.20, 129.33, 129.53, 130.02, 130.33, 131.09, 131.79, 131.84, 131.92, 132.26, 132.42, 132.82, 132.91, 133.54, 138.51, 139.01, 139.41, 139.52, 140.67, 140.94, 141.87, 142.00, 143.12, 145.02, 145.37, 150.04, 150.18, 150.58, 157.08, 157.20, 157.42, 157.48, 163.82, 163.90, 163.96, 164.00, 170.30, 170.49, 171.16, 171.64. HRMS m/z calculated for C_33_H_33_F_3_N_7_O_7_ 696.2388, found 696.2385.

#### N-(3-(1H-dibenzo[b,f][1,2,3]triazolo[4,5-d]azocin-8(9H)-yl)-3-oxopropyl)-2,2,2-trifluoroacetamide (10a)

Dibenzoazocine **7** (50 mg, 0.13 mmol) was dissolved in MeOH (5 ml), and sodium azide (13 mg, 0.19 mmol) was added. The solution was stirred at room temperature for 5 minutes, diluted with MeOH to 8 ml and then purified by semi-preparative HPLC. The mobile phase consisted of (A) 0.01 M ammonium acetate in water and (B) acetonitrile, with B linearly programmed to change from 30% to 60% over the course of 6 min. White solid, 38 mg (70%).

^1^H NMR (500 MHz, CDCl_3_-*d*) δ ppm 1.91 (dt, *J* = 17.18, 6.00 Hz, 1 H), 2.14 (dt, *J* = 17.26, 6.00 Hz, 1 H), 3.28 (q, *J* = 6.00 Hz, 2 H), 4.50 (d, *J* = 17.30 Hz, 1 H), 6.11 (d, *J* = 17.30 Hz, 1 H), 7.21 (d, *J* = 7.50 Hz, 1 H), 7.23–7.27 (m, 1 H), 7.29–7.33 (m, 1 H), 7.42 (dd, *J* = 8.02, 0.86 Hz, 1 H), 7.46–7.50 (m, 1 H), 7.52 (dd, *J* = 7.73, 1.15 Hz, 1 H), 7.55–7.59 (m, 1 H), 7.62–7.67 (m, 1 H), 7.72 (br. s, 1 H), 7.73 (d, *J* = 1.15 Hz, 1 H); ^13^C NMR (126 MHz, CDCl_3_-*d*) δ ppm 32.95, 35.36, 52.60, 115.94 (q, *J* = 287.9 Hz), 124.46, 127.49, 128.09, 128.72, 128.93, 129.65, 129.97, 130.18, 131.95, 132.78, 134.14, 139.14, 139.97, 140.94, 157.19 (q, *J* = 36.5 Hz), 171.46. HRMS m/z calculated for C_20_H_17_F_3_N_5_O_2_ 416.1329, found 416.1328.

#### 2,2,2-trifluoro-N-(3-oxo-3-(1-(2-oxo-2H-chromen-3-yl)-1H-dibenzo[b,f][1,2,3]triazolo[4,5-d]azocin-8(9H)-yl)propyl)acetamide (11a) 2,2,2-trifluoro-N-(3-oxo-3-(3-(2-oxo-2H-chromen-3-yl)-3H-dibenzo[b,f][1,2,3]triazolo[4,5-d]azocin-8(9H)-yl)propyl)acetamide (11b)

Dibenzoazocine **7** (149 mg, 0.4 mmol) was dissolved in MeOH (4 ml) and 3-azidocoumarine (50 mg, 0.26 mmol) was added. The solution was stirred at room temperature for 15 minutes, diluted with MeOH to 8 ml and then purified by semi-preparative HPLC. The mobile phase consisted of (A) 0.01 M ammonium acetate in water and (B) acetonitrile, with B linearly programmed to change from 50% to 80% over the course of 6 min. Derivatives **11a** and **11b** were collected separately. Derivative **11a** was the first compound and was isolated as a white solid, 27 mg (19%). Derivative **11b** was the second compound and was isolated as a white solid, 31 mg (21%).

#### 11a

^1^H NMR (500 MHz, CDCl_3_-*d*) δ ppm 1.83–1.90 (m, 1 H), 2.07 (ddd, *J* = 17.47, 8.59, 4.01 Hz, 1 H), 3.25 (ddt, *J* = 13.50, 9.06, 4.47, 4.47 Hz, 1 H), 3.39–3.47 (m, 1 H), 4.45 (d, *J* = 16.61 Hz, 1 H), 6.29 (d, *J* = 16.60 Hz, 1 H), 6.91 (d, *J* = 7.73 Hz, 1 H), 7.06–7.10 (m, 1 H), 7.21–7.24 (m, 1 H), 7.26–7.28 (m, 1 H), 7.30–7.34 (m, 1 H), 7.36 (d, *J* = 0.86 Hz, 1 H), 7.36–7.40 (m, 2 H), 7.50–7.53 (m, 2 H), 7.61–7.66 (m, 2 H), 7.74–7.78 (m, 1 H), 8.20 (s, 1 H); ^13^C NMR (126 MHz, CDCl_3_-*d*) δ ppm 33.31, 35.16, 51.67, 115.86 (q, *J* = 287.9 Hz), 117.01, 117.73, 123.60, 124.72, 125.35, 127.33, 127.73, 129.13, 129.69, 129.82, 130.04, 130.08, 130.33, 130.90, 131.10, 133.46, 135.09, 136.24, 139.75, 140.12, 142.46, 153.68, 155.93, 156.61 (q, *J* = 36.5 Hz), 171.43. HRMS m/z calculated for C_29_H_21_F_3_N_5_O_4_ 560.1540, found 560.1540.

#### 11b

^1^H NMR (500 MHz, CDCl_3_-*d*) δ ppm 2.34 (dd, *J* = 7.16, 4.58 Hz, 1 H), 2.37 (dd, *J* = 6.44, 4.44 Hz, 1 H), 3.42–3.49 (m, 1 H), 3.60–3.68 (m, 1 H), 4.68 (d, *J* = 15.50 Hz, 1 H), 5.49 (d, *J* = 15.47 Hz, 1 H), 7.10–7.12 (m, 2 H), 7.21–7.24 (m, 1 H), 7.29–7.31 (m, 2 H), 7.31–7.35 (m, 2 H), 7.40 (d, *J* = 8.31 Hz, 2 H), 7.60–7.63 (m, 2 H), 7.67–7.71 (m, 1 H), 7.74 (dd, *J* = 8.02, 1.43 Hz, 1 H), 8.39 (s, 1 H); ^13^C NMR (126 MHz, CDCl_3_-*d*) δ ppm 33.36, 35.76, 53.76, 115.85 (q, *J* = 289.0 Hz), 117.03, 117.84, 123.04, 125.23, 125.71, 127.43, 127.99, 128.80, 128.92, 129.29, 129.75, 130.14, 131.04, 131.30, 131.97, 133.20, 133.89, 137.41, 138.82, 140.05, 141.38, 153.67, 156.22, 157.19 (q, *J* = 36.6 Hz), 171.85. HRMS m/z calculated for C_29_H_21_F_3_N_5_O_4_ 560.1540, found 560.1539.

#### 2-((benzoyloxy)methyl)-5-(2,4-dioxo-5-((8-(3-(2,2,2-trifluoroacetamido)propanoyl)-8,9-dihydro-1H-dibenzo[b,f][1,2,3]triazolo[4,5-d]azocin-1-yl)methyl)-3,4-dihydropyrimidin-1(2H)-yl)tetrahydrofuran-3,4-diyl dibenzoate 12a 2-((benzoyloxy)methyl)-5-(2,4-dioxo-5-((8-(3-(2,2,2-trifluoroacetamido)propanoyl)-8,9-dihydro-3H-dibenzo[b,f][1,2,3]triazolo[4,5-d]azocin-3-yl)methyl)-3,4-dihydropyrimidin-1(2H)-yl)tetrahydrofuran-3,4-diyl dibenzoate 12b

Dibenzoazocine **7** (46 mg, 0.12 mmol) was dissolved in MeOH (4 ml) and 2´,3´,5´tribenzoyl-5-azidomethyluridine (50 mg, 0.08 mmol) was added. The solution was stirred at room temperature for 15 minutes, diluted with MeOH to 8 ml and then purified using semipreparative HPLC. The mobile phase consisted of (A) 0.01 M ammonium acetate in water and (B) acetonitrile, with B linearly programmed to change from 50% to 80% over the course of 6 min. The compound **12** was separated as a mixture of two inseparable regioisomers. Because it was not possible to identify the ^1^H and ^13^C NMR signals for individual regisomers, characterization was performed for both regioisomers together. White solid 35 mg (44%).

^1^H NMR (500 MHz, CDCl_3_-*d*) δ ppm 1.69–1.80 (m, 6 H), 1.81–1.90 (m, 1 H), 1.92–2.01 (m, 1 H), 2.07–2.17 (m, 1 H), 2.22–2.28 (m, 1 H), 2.62–2.72 (m, 1 H), 3.09–3.18 (m, 1 H), 3.19–3.34 (m, 2 H), 3.36–3.47 (m, 1 H), 4.23–4.31 (m, 1 H), 4.39–4.45 (m, 1 H), 4.59–4.73 (m, 4 H), 4.74–4.82 (m, 2 H), 4.84–4.90 (m, 1 H), 4.98–5.02 (m, 1 H), 5.25–5.31 (m, 1 H), 5.62–5.67 (m, 1 H), 5.82 (dt, *J* = 15.90, 5.80 Hz, 1 H), 5.88–5.93 (m, 1 H), 5.94–5.99 (m, 1 H), 6.01–6.11 (m, 1 H), 6.21 (dd, *J* = 12.60, 4.87 Hz, 1 H), 6.26–6.29 (m, 1 H), 6.34 (d, *J* = 5.44 Hz, 1 H), 6.92–6.96 (m, 1 H), 7.04–7.08 (m, 1 H), 7.23–7.29 (m, 7 H), 7.31–7.42 (m, 12 H), 7.43–7.48 (m, 4 H), 7.51–7.60 (m, 8 H), 7.90–7.94 (m, 4 H), 7.95–8.01 (m, 3 H), 8.07–8.12 (m, 3 H), 8.76–9.07 (m, 2 H); ^13^C NMR (126 MHz, CDCl_3_-*d*) δ ppm 33.06, 33.10, 33.36, 33.39, 35.16, 35.27, 35.37, 44.13, 44.99, 45.60, 51.31, 52.86, 63.57, 64.00, 70.82, 71.26, 71.35, 74.01, 74.19, 80.32, 80.49, 80.91, 89.15, 89.29, 89.68, 89.78, 107.94, 108.62, 109.13, 127.09, 127.29, 127.45, 127.49, 128.37, 128.53, 128.63, 128.89, 129.61, 129.78, 129.83, 129.93, 130.87, 131.21, 131.41, 131.89, 132.68, 133.50, 133.73, 133.79, 133.83, 134.57, 135.09, 135.39, 139.60, 139.66, 139.96, 140.37, 140.42, 142.24, 142.62, 142.71, 144.60, 145.40, 149.31, 149.38, 149.40, 149.48, 156.84, 157.13, 157.42, 161.34, 161.45, 161.85, 161.91, 165.23, 165.28, 165.31, 165.35, 166.01, 170.12, 170.27, 171.45, 171.50.

#### 2-(5-(Azidomethyl)-2,4-dioxo-3,4-dihydropyrimidin-1(2*H*)-yl)-5-((benzoyloxy)methyl)tetrahydrofuran-3,4-diyl dibenzoate

5-hydroxymethylenuracil (3.25 g, 22.8 mmol) was suspended in HMDS (120 ml), and a catalytic amount of ammonium sulfate was added. The mixture was stirred under reflux for 2 hours and evaporated. The resulting solid was dissolved in 1,2-dichloroethane (85 ml). 1-*O*-Acetyl-2,3,5-tri-*O*-benzoyl-β-D-ribofuranose (11.2 g, 22.2 mmol) and trimethylsilyl trifluormethansulfonate (4.69 ml, 25.9 mmol) were added. The reaction was stirred at room temperature overnight. Then, extraction with saturated sodium hydrogen carbonate solution was performed. The organic layer was dried over anhydrous sodium sulfate, and the solvent was evaporated. The crude product 5-hydroxymethylenuridin (15 g) was dissolved without purification in acetonitrile (300 ml), and thionyl chloride (2.3 ml) was added. The reaction mixture was stirred at room temperature for 30 minutes, and triethylamine (4.5 ml) was added to neutralize the reaction. The solvent was evaporated, the resulting solid was dissolved in anhydrous DMF (187.5 ml), and sodium azide (9.5 g, 146.7 mmol) was added. The reaction was stirred at 90°C overnight and then diluted with water (1.5 l). The white precipitate was obtained by filtration and washed with water. The crude product was purified first on a silica gel column with a mobile phase of Tol:EtAc:HCOOH (7:2:0.2 v/v/v). White solid 2.75 g (33%). For characterization the product was purified again using semipreparative HPLC. The mobile phase consisted of (A) 0.01 M ammonium acetate in water and (B) acetonitrile, with B linearly programmed to shift from 50% to 80% over the course of 6 min.

^1^H NMR (400 MHz, DMSO-*d*_6_) δ ppm 4.01 (dd, *J* = 13.60, 7.00 Hz, 1 H), 4.64 (dd, *J* = 11.90, 5.20 Hz, 1 H), 4.71 (d, *J* = 3.73 Hz, 1 H), 4.73–4.79 (m, 1 H), 5.91–5.97 (m, 1 H), 6.19–6.23 (m, 1 H), 7.41–7.48 (m, 1 H), 7.48–7.53 (m, 1 H), 7.61–7.68 (m, 1 H), 7.89 (ddd, *J* = 8.22, 4.17, 1.21 Hz, 1 H), 7.99–8.01 (m, 1 H), 8.02 (d, *J* = 0.88 Hz, 1 H), 11.76 (br. s., 1 H); ^13^C NMR (101 MHz, DMSO-*d*_6_) δ ppm 46.66, 63.62, 70.42, 73.26, 78.79, 89.08, 109.09, 128.42, 128.72–128.75 (3C), 129.19–129.32 (3C), 129.36, 133.57, 133.85, 133.96, 141.02, 150.07, 162.84, 164.59, 164.61, 165.48. HRMS m/z calculated for C_31_H_26_N_5_O_9_ [M+H]^+^ 612.1725, found 612.1723.

## Supporting Information

S1 Fig ^1^H NMR spectrum of 1-(3,4-Dihydroxy-5-iodomethyl-tetrahydrofuran-2-yl)-5-methyl-1*H*-pyrimidin-2,4-dione 3a (DMSO). (TIF)

S2 Fig ^13^C NMR spectrum of 1-(3,4-Dihydroxy-5-iodomethyl-tetrahydrofuran-2-yl)-5-methyl-1*H*-pyrimidin-2,4-dione 3a (DMSO). (TIF)

S3 Fig ^1^H NMR spectrum of 1-(3,4-Dihydroxy-5-bromomethyl-tetrahydrofuran-2-yl)-5-methyl-1*H*-pyrimidin-2,4-dione 3b (DMSO). (TIF)

S4 Fig ^13^C NMR spectrum of 1-(3,4-Dihydroxy-5-bromomethyl-tetrahydrofuran-2-yl)-5-methyl-1*H*-pyrimidin-2,4-dione 3b (DMSO). (TIF)

S5 Fig ^1^H NMR spectrum of 5´-Deoxy-5´-iodo-5-methyl-2´,3´-O-isopropyliden-uridine 4a (DMSO). (TIF)

S6 Fig ^13^C NMR spectrum of 5´-Deoxy-5´-iodo-5-methyl-2´,3´-O-isopropyliden-uridine 4a (DMSO). (TIF)

S7 Fig ^1^H NMR spectrum of 5´-Bromo-5´-deoxy-5-methyl-2´,3´-O-isopropyliden-uridine 4b (DMSO). (TIF)

S8 Fig ^13^C NMR spectrum of 5´-Bromo-5´-deoxy-5-methyl-2´,3´-O-isopropyliden-uridine 4b (DMSO). (TIF)

S9 Fig ^1^H NMR spectrum of 1-(5-(azidomethyl)-3,4-dihydroxytetrahydrofuran-2-yl)-5-methylpyrimidine-2,4(1*H*,3*H*)-dione 5 (DMSO). (TIF)

S10 Fig ^13^C NMR spectrum of 1-(5-(azidomethyl)-3,4-dihydroxytetrahydrofuran-2-yl)-5-methylpyrimidine-2,4(1*H*,3*H*)-dione 5 (DMSO). (TIF)

S11 Fig ^1^H NMR spectrum of 5´-Azido-5´-deoxy-5-methyl-2´,3´-O-isopropyliden-uridine 6 (DMSO). (TIF)

S12 Fig ^13^C NMR spectrum of 5´-Azido-5´-deoxy-5-methyl-2´,3´-O-isopropyliden-uridine 6 (DMSO). (TIF)

S13 Fig ^1^H NMR spectrum of *N*-(3-(1-((-3,4-dihydroxy-5-(5-methyl-2,4-dioxo-3,4-dihydropyrimidin-1(2*H*)-yl)tetrahydrofuran-2-yl)methyl)-1*H*-dibenzo[b,f][1,2,3]triazolo[4,5-d]azocin-8(9*H*)-yl)-3-oxopropyl)-2,2,2-trifluoroacetamide 8a (CDCl_3_). (TIF)

S14 Fig ^1^H NMR spectrum of *N*-(3-(1-((-3,4-dihydroxy-5-(5-methyl-2,4-dioxo-3,4-dihydropyrimidin-1(2*H*)-yl)tetrahydrofuran-2-yl)methyl)-1*H*-dibenzo[b,f][1,2,3]triazolo[4,5-d]azocin-8(9*H*)-yl)-3-oxopropyl)-2,2,2-trifluoroacetamide 8a (D_2_O). (TIF)

S15 Fig ^1^H NMR spectrum of *N*-(3-(1-((-3,4-dihydroxy-5-(5-methyl-2,4-dioxo-3,4-dihydropyrimidin-1(2*H*)-yl)tetrahydrofuran-2-yl)methyl)-1*H*-dibenzo[b,f][1,2,3]triazolo[4,5-d]azocin-8(9*H*)-yl)-3-oxopropyl)-2,2,2-trifluoroacetamide 8a (DMF). (TIF)

S16 Fig ^1^H NMR spectrum of *N*-(3-(1-((-3,4-dihydroxy-5-(5-methyl-2,4-dioxo-3,4-dihydropyrimidin-1(2*H*)-yl)tetrahydrofuran-2-yl)methyl)-1*H*-dibenzo[b,f][1,2,3]triazolo[4,5-d]azocin-8(9*H*)-yl)-3-oxopropyl)-2,2,2-trifluoroacetamide 8a (DMSO). (TIF)

S17 Fig ^1^H NMR spectrum of *N*-(3-(1-((-3,4-dihydroxy-5-(5-methyl-2,4-dioxo-3,4-dihydropyrimidin-1(2*H*)-yl)tetrahydrofuran-2-yl)methyl)-1*H*-dibenzo[b,f][1,2,3]triazolo[4,5-d]azocin-8(9*H*)-yl)-3-oxopropyl)-2,2,2-trifluoroacetamide 8a (MeOD). (TIF)

S18 Fig ^13^C NMR spectrum of *N*-(3-(1-((-3,4-dihydroxy-5-(5-methyl-2,4-dioxo-3,4-dihydropyrimidin-1(2*H*)-yl)tetrahydrofuran-2-yl)methyl)-1*H*-dibenzo[b,f][1,2,3]triazolo[4,5-d]azocin-8(9*H*)-yl)-3-oxopropyl)-2,2,2-trifluoroacetamide 8a (CDCl_3_). (TIF)

S19 Fig ^19^F NMR spectrum of *N*-(3-(1-((-3,4-dihydroxy-5-(5-methyl-2,4-dioxo-3,4-dihydropyrimidin-1(2*H*)-yl)tetrahydrofuran-2-yl)methyl)-1*H*-dibenzo[b,f][1,2,3]triazolo[4,5-d]azocin-8(9*H*)-yl)-3-oxopropyl)-2,2,2-trifluoroacetamide 8a (CDCl_3_). (TIF)

S20 Fig ^1^H –^1^H COSY NMR spectrum of *N*-(3-(1-((-3,4-dihydroxy-5-(5-methyl-2,4-dioxo-3,4-dihydropyrimidin-1(2*H*)-yl)tetrahydrofuran-2-yl)methyl)-1*H*-dibenzo[b,f][1,2,3]triazolo[4,5-d]azocin-8(9*H*)-yl)-3-oxopropyl)-2,2,2-trifluoroacetamide 8a (CDCl_3_). (TIF)

S21 Fig ^1^H –^13^C HMQC NMR spectrum of *N*-(3-(1-((-3,4-dihydroxy-5-(5-methyl-2,4-dioxo-3,4-dihydropyrimidin-1(2*H*)-yl)tetrahydrofuran-2-yl)methyl)-1*H*-dibenzo[b,f][1,2,3]triazolo[4,5-d]azocin-8(9*H*)-yl)-3-oxopropyl)-2,2,2-trifluoroacetamide 8a (CDCl_3_). (TIF)

S22 Fig ^1^H—^13^C HMBC NMR spectrum of *N*-(3-(1-((-3,4-dihydroxy-5-(5-methyl-2,4-dioxo-3,4-dihydropyrimidin-1(2*H*)-yl)tetrahydrofuran-2-yl)methyl)-1*H*-dibenzo[b,f][1,2,3]triazolo[4,5-d]azocin-8(9*H*)-yl)-3-oxopropyl)-2,2,2-trifluoroacetamide 8a (CDCl_3_). (TIF)

S23 Fig ^1^H—^15^N HMQC NMR spectrum of *N*-(3-(1-((-3,4-dihydroxy-5-(5-methyl-2,4-dioxo-3,4-dihydropyrimidin-1(2*H*)-yl)tetrahydrofuran-2-yl)methyl)-1*H*-dibenzo[b,f][1,2,3]triazolo[4,5-d]azocin-8(9*H*)-yl)-3-oxopropyl)-2,2,2-trifluoroacetamide 8a (CDCl_3_). (TIF)

S24 Fig ^1^H—^15^N HMBC NMR spectrum of *N*-(3-(1-((-3,4-dihydroxy-5-(5-methyl-2,4-dioxo-3,4-dihydropyrimidin-1(2*H*)-yl)tetrahydrofuran-2-yl)methyl)-1*H*-dibenzo[b,f][1,2,3]triazolo[4,5-d]azocin-8(9*H*)-yl)-3-oxopropyl)-2,2,2-trifluoroacetamide 8a (CDCl_3_). (TIF)

S25 Fig ^1^H – ^1^H ROESY NMR spectrum of *N*-(3-(1-((-3,4-dihydroxy-5-(5-methyl-2,4-dioxo-3,4-dihydropyrimidin-1(2*H*)-yl)tetrahydrofuran-2-yl)methyl)-1*H*-dibenzo[b,f][1,2,3]triazolo[4,5-d]azocin-8(9*H*)-yl)-3-oxopropyl)-2,2,2-trifluoroacetamide 8a (CDCl_3_). (TIF)

S26 Fig ^1^H NMR spectrum of *N*-(3-(3-((-3,4-dihydroxy-5-(5-methyl-2,4-dioxo-3,4-dihydropyrimidin-1(2*H*)-yl)tetrahydrofuran-2-yl)methyl)-3*H*-dibenzo[b,f][1,2,3]triazolo[4,5-d]azocin-8(9*H*)-yl)-3-oxopropyl)-2,2,2-trifluoroacetamide 8b (MeOD) at 0°C. (TIF)

S27 Fig ^13^C NMR spectrum of *N*-(3-(3-((-3,4-dihydroxy-5-(5-methyl-2,4-dioxo-3,4-dihydropyrimidin-1(2*H*)-yl)tetrahydrofuran-2-yl)methyl)-3*H*-dibenzo[b,f][1,2,3]triazolo[4,5-d]azocin-8(9*H*)-yl)-3-oxopropyl)-2,2,2-trifluoroacetamide 8b (DMSO). (TIF)

S28 Fig ^1^H NMR spectrum of *N*-(3-(1-((2,2-dimethyl-6-(5-methyl-2,4-dioxo-3,4-dihydropyrimidin-1(2*H*)-yl)tetrahydrofuro[3,4-d][1,3]dioxol-4-yl)methyl)-1*H*-dibenzo[b,f][1,2,3]triazolo[4,5-d]azocin-8(9*H*)-yl)-3-oxopropyl)-2,2,2-trifluoroacetamide 9a (CDCl_3_). (TIF)

S29 Fig ^1^H NMR spectrum of *N*-(3-(1-((2,2-dimethyl-6-(5-methyl-2,4-dioxo-3,4-dihydropyrimidin-1(2*H*)-yl)tetrahydrofuro[3,4-d][1,3]dioxol-4-yl)methyl)-1*H*-dibenzo[b,f][1,2,3]triazolo[4,5-d]azocin-8(9*H*)-yl)-3-oxopropyl)-2,2,2-trifluoroacetamide 9a (Acetone). (TIF)

S30 Fig ^1^H NMR spectrum of *N*-(3-(1-((2,2-dimethyl-6-(5-methyl-2,4-dioxo-3,4-dihydropyrimidin-1(2*H*)-yl)tetrahydrofuro[3,4-d][1,3]dioxol-4-yl)methyl)-1*H*-dibenzo[b,f][1,2,3]triazolo[4,5-d]azocin-8(9*H*)-yl)-3-oxopropyl)-2,2,2-trifluoroacetamide 9a (DMF). (TIF)

S31 Fig ^1^H NMR spectrum of *N*-(3-(1-((2,2-dimethyl-6-(5-methyl-2,4-dioxo-3,4-dihydropyrimidin-1(2*H*)-yl)tetrahydrofuro[3,4-d][1,3]dioxol-4-yl)methyl)-1*H*-dibenzo[b,f][1,2,3]triazolo[4,5-d]azocin-8(9*H*)-yl)-3-oxopropyl)-2,2,2-trifluoroacetamide 9a (DMSO). (TIF)

S32 Fig ^1^H NMR spectrum of *N*-(3-(1-((2,2-dimethyl-6-(5-methyl-2,4-dioxo-3,4-dihydropyrimidin-1(2*H*)-yl)tetrahydrofuro[3,4-d][1,3]dioxol-4-yl)methyl)-1*H*-dibenzo[b,f][1,2,3]triazolo[4,5-d]azocin-8(9*H*)-yl)-3-oxopropyl)-2,2,2-trifluoroacetamide 9a (MeOD). (TIF)

S33 Fig ^13^C NMR spectrum of *N*-(3-(1-((2,2-dimethyl-6-(5-methyl-2,4-dioxo-3,4-dihydropyrimidin-1(2*H*)-yl)tetrahydrofuro[3,4-d][1,3]dioxol-4-yl)methyl)-1*H*-dibenzo[b,f][1,2,3]triazolo[4,5-d]azocin-8(9*H*)-yl)-3-oxopropyl)-2,2,2-trifluoroacetamide 9a (CDCl_3_). (TIF)

S34 Fig Details of ^1^H NMR spectra of *N*-(3-(1-((2,2-dimethyl-6-(5-methyl-2,4-dioxo-3,4-dihydropyrimidin-1(2*H*)-yl)tetrahydrofuro[3,4-d][1,3]dioxol-4-yl)methyl)-1*H*-dibenzo[b,f][1,2,3]triazolo[4,5-d]azocin-8(9*H*)-yl)-3-oxopropyl)-2,2,2-trifluoroacetamide at different temperatures 9a (DMSO). The bottom spectrum was measured after cooling the sample back.
(TIF)

S35 Fig ^19^F NMR spectrum of *N*-(3-(1-((2,2-dimethyl-6-(5-methyl-2,4-dioxo-3,4-dihydropyrimidin-1(2*H*)-yl)tetrahydrofuro[3,4-d][1,3]dioxol-4-yl)methyl)-1*H*-dibenzo[b,f][1,2,3]triazolo[4,5-d]azocin-8(9*H*)-yl)-3-oxopropyl)-2,2,2-trifluoroacetamide 9a (DMSO). (TIF)

S36 Fig ^19^F NMR spectra of *N*-(3-(1-((2,2-dimethyl-6-(5-methyl-2,4-dioxo-3,4-dihydropyrimidin-1(2*H*)-yl)tetrahydrofuro[3,4-d][1,3]dioxol-4-yl)methyl)-1*H*-dibenzo[b,f][1,2,3]triazolo[4,5-d]azocin-8(9*H*)-yl)-3-oxopropyl)-2,2,2-trifluoroacetamide 9a in DMSO at different temperatures (heating and cooling back). (TIF)

S37 Fig ^1^H – ^1^H COSY NMR spectrum *N*-(3-(1-((2,2-dimethyl-6-(5-methyl-2,4-dioxo-3,4-dihydropyrimidin-1(2*H*)-yl)tetrahydrofuro[3,4-d][1,3]dioxol-4-yl)methyl)-1*H*-dibenzo[b,f][1,2,3]triazolo[4,5-d]azocin-8(9*H*)-yl)-3-oxopropyl)-2,2,2-trifluoroacetamide 9a (CDCl_3_). (TIF)

S38 Fig ^1^H – ^13^C HMQC NMR spectrum of *N*-(3-(1-((2,2-dimethyl-6-(5-methyl-2,4-dioxo-3,4-dihydropyrimidin-1(2*H*)-yl)tetrahydrofuro[3,4-d][1,3]dioxol-4-yl)methyl)-1*H*-dibenzo[b,f][1,2,3]triazolo[4,5-d]azocin-8(9*H*)-yl)-3-oxopropyl)-2,2,2-trifluoroacetamide 9a (CDCl_3_). (TIF)

S39 Fig ^1^H – ^13^C HMBC NMR spectrum of *N*-(3-(1-((2,2-dimethyl-6-(5-methyl-2,4-dioxo-3,4-dihydropyrimidin-1(2*H*)-yl)tetrahydrofuro[3,4-d][1,3]dioxol-4-yl)methyl)-1*H*-dibenzo[b,f][1,2,3]triazolo[4,5-d]azocin-8(9*H*)-yl)-3-oxopropyl)-2,2,2-trifluoroacetamide 9a (CDCl_3_). (TIF)

S40 Fig ^1^H – ^15^N HMQC NMR spectrum of *N*-(3-(1-((2,2-dimethyl-6-(5-methyl-2,4-dioxo-3,4-dihydropyrimidin-1(2*H*)-yl)tetrahydrofuro[3,4-d][1,3]dioxol-4-yl)methyl)-1*H*-dibenzo[b,f][1,2,3]triazolo[4,5-d]azocin-8(9*H*)-yl)-3-oxopropyl)-2,2,2-trifluoroacetamide 9a (CDCl_3_). (TIF)

S41 Fig ^1^H – ^15^N HMBC NMR spectrum of *N*-(3-(1-((2,2-dimethyl-6-(5-methyl-2,4-dioxo-3,4-dihydropyrimidin-1(2*H*)-yl)tetrahydrofuro[3,4-d][1,3]dioxol-4-yl)methyl)-1*H*-dibenzo[b,f][1,2,3]triazolo[4,5-d]azocin-8(9*H*)-yl)-3-oxopropyl)-2,2,2-trifluoroacetamide 9a (CDCl_3_). (TIF)

S42 Fig ^1^H – ^1^H ROESY NMR spectrum of *N*-(3-(1-((2,2-dimethyl-6-(5-methyl-2,4-dioxo-3,4-dihydropyrimidin-1(2*H*)-yl)tetrahydrofuro[3,4-d][1,3]dioxol-4-yl)methyl)-1*H*-dibenzo[b,f][1,2,3]triazolo[4,5-d]azocin-8(9*H*)-yl)-3-oxopropyl)-2,2,2-trifluoroacetamide 9a (CDCl_3_). (TIF)

S43 Fig ^1^H NMR spectrum of *N*-(3-(3-((2,2-Dimethyl-6-(5-methyl-2,4-dioxo-3,4-dihydropyrimidin-1(2*H*)-yl)tetrahydrofuro[3,4-d][1,3]dioxol-4-yl)methyl)-3*H*-dibenzo[b,f][1,2,3]triazolo[4,5-d]azocin-8(9*H*)-yl)-3-oxopropyl)-2,2,2-trifluoroacetamide 9b (CDCl_3_). Formation of four conformers is evident especially from NH signals at 9.11–9.66.
(TIF)

S44 Fig ^13^C NMR spectrum of *N*-(3-(3-((2,2-Dimethyl-6-(5-methyl-2,4-dioxo-3,4-dihydropyrimidin-1(2*H*)-yl)tetrahydrofuro[3,4-d][1,3]dioxol-4-yl)methyl)-3*H*-dibenzo[b,f][1,2,3]triazolo[4,5-d]azocin-8(9*H*)-yl)-3-oxopropyl)-2,2,2-trifluoroacetamide 9b (CDCl_3_) (TIF)

S45 Fig Details of ^1^H NMR spectra of *N*-(3-(3-((2,2-Dimethyl-6-(5-methyl-2,4-dioxo-3,4-dihydropyrimidin-1(2*H*)-yl)tetrahydrofuro[3,4-d][1,3]dioxol-4-yl)methyl)-3*H*-dibenzo[b,f][1,2,3]triazolo[4,5-d]azocin-8(9*H*)-yl)-3-oxopropyl)-2,2,2-trifluoroacetamide 9b in DMSO at different temperatures (heating and cooling back). (TIF)

S46 Fig ^19^F NMR spectrum of *N*-(3-(3-((2,2-Dimethyl-6-(5-methyl-2,4-dioxo-3,4-dihydropyrimidin-1(2*H*)-yl)tetrahydrofuro[3,4-d][1,3]dioxol-4-yl)methyl)-3*H*-dibenzo[b,f][1,2,3]triazolo[4,5-d]azocin-8(9*H*)-yl)-3-oxopropyl)-2,2,2-trifluoroacetamide 9b (DMSO). (TIF)

S47 Fig Details of ^19^F NMR spectra of *N*-(3-(3-((2,2-Dimethyl-6-(5-methyl-2,4-dioxo-3,4-dihydropyrimidin-1(2*H*)-yl)tetrahydrofuro[3,4-d][1,3]dioxol-4-yl)methyl)-3*H*-dibenzo[b,f][1,2,3]triazolo[4,5-d]azocin-8(9*H*)-yl)-3-oxopropyl)-2,2,2-trifluoroacetamide 9b in DMSO at different temperatures (heating and cooling back). (TIF)

S48 Fig ^1^H – ^1^H COSY NMR spectrum of *N*-(3-(3-((2,2-Dimethyl-6-(5-methyl-2,4-dioxo-3,4-dihydropyrimidin-1(2*H*)-yl)tetrahydrofuro[3,4-d][1,3]dioxol-4-yl)methyl)-3*H*-dibenzo[b,f][1,2,3]triazolo[4,5-d]azocin-8(9*H*)-yl)-3-oxopropyl)-2,2,2-trifluoroacetamide 9b (DMSO). (TIF)

S49 Fig ^1^H – ^15^N HMQC NMR spectrum of *N*-(3-(3-((2,2-Dimethyl-6-(5-methyl-2,4-dioxo-3,4-dihydropyrimidin-1(2*H*)-yl)tetrahydrofuro[3,4-d][1,3]dioxol-4-yl)methyl)-3*H*-dibenzo[b,f][1,2,3]triazolo[4,5-d]azocin-8(9*H*)-yl)-3-oxopropyl)-2,2,2-trifluoroacetamide 9b (DMSO). (TIF)

S50 Fig ^1^H – ^15^N HMBC NMR spectrum of *N*-(3-(3-((2,2-Dimethyl-6-(5-methyl-2,4-dioxo-3,4-dihydropyrimidin-1(2*H*)-yl)tetrahydrofuro[3,4-d][1,3]dioxol-4-yl)methyl)-3*H*-dibenzo[b,f][1,2,3]triazolo[4,5-d]azocin-8(9*H*)-yl)-3-oxopropyl)-2,2,2-trifluoroacetamide 9b (DMSO). (TIF)

S51 Fig ^1^H – ^1^H ROESY NMR spectrum of *N*-(3-(3-((2,2-Dimethyl-6-(5-methyl-2,4-dioxo-3,4-dihydropyrimidin-1(2*H*)-yl)tetrahydrofuro[3,4-d][1,3]dioxol-4-yl)methyl)-3*H*-dibenzo[b,f][1,2,3]triazolo[4,5-d]azocin-8(9*H*)-yl)-3-oxopropyl)-2,2,2-trifluoroacetamide 9b (DMSO). (TIF)

S52 Fig ^1^H NMR spectrum of *N*-(3-(1H-dibenzo[b,f][1,2,3]triazolo[4,5-d]azocin-8(9*H*)-yl)-3-oxopropyl)-2,2,2-trifluoroacetamide 10 (CDCl_3_). (TIF)

S53 Fig ^13^C NMR spectrum of *N*-(3-(1H-dibenzo[b,f][1,2,3]triazolo[4,5-d]azocin-8(9*H*)-yl)-3-oxopropyl)-2,2,2-trifluoroacetamide 10 (CDCl_3_). (TIF)

S54 Fig ^19^F NMR spectrum of *N*-(3-(1H-dibenzo[b,f][1,2,3]triazolo[4,5-d]azocin-8(9*H*)-yl)-3-oxopropyl)-2,2,2-trifluoroacetamide 10 (CDCl_3_). (TIF)

S55 Fig ^1^H – ^1^H COSY NMR spectrum of *N*-(3-(1H-dibenzo[b,f][1,2,3]triazolo[4,5-d]azocin-8(9*H*)-yl)-3-oxopropyl)-2,2,2-trifluoroacetamide 10 (CDCl_3_). (TIF)

S56 Fig ^1^H – ^13^C HMQC NMR spectrum of *N*-(3-(1H-dibenzo[b,f][1,2,3]triazolo[4,5-d]azocin-8(9*H*)-yl)-3-oxopropyl)-2,2,2-trifluoroacetamide 10 (CDCl_3_). (TIF)

S57 Fig ^1^H – ^13^C HMBC NMR spectrum of *N*-(3-(1H-dibenzo[b,f][1,2,3]triazolo[4,5-d]azocin-8(9*H*)-yl)-3-oxopropyl)-2,2,2-trifluoroacetamide 10 (CDCl_3_). (TIF)

S58 Fig Detail of ^1^H – ^15^N HMQC NMR spectrum of *N*-(3-(1H-dibenzo[b,f][1,2,3]triazolo[4,5-d]azocin-8(9*H*)-yl)-3-oxopropyl)-2,2,2-trifluoroacetamide 10 (CDCl_3_). (TIF)

S59 Fig ^1^H – ^15^N HMBC NMR spectrum of *N*-(3-(1H-dibenzo[b,f][1,2,3]triazolo[4,5-d]azocin-8(9*H*)-yl)-3-oxopropyl)-2,2,2-trifluoroacetamide 10 (CDCl_3_). (TIF)

S60 Fig ^1^H – ^1^H ROESY NMR spectrum of *N*-(3-(1H-dibenzo[b,f][1,2,3]triazolo[4,5-d]azocin-8(9*H*)-yl)-3-oxopropyl)-2,2,2-trifluoroacetamide 10 (CDCl_3_). (TIF)

S61 Fig ^1^H NMR spectrum of 2,2,2-trifluoro-*N*-(3-oxo-3-(1-(2-oxo-2*H*-chromen-3-yl)-1*H*-dibenzo[b,f][1,2,3]triazolo[4,5-d]azocin-8(9*H*)-yl)propyl)acetamide 11a (CDCl_3_). (TIF)

S62 Fig ^13^C NMR spectrum of 2,2,2-trifluoro-*N*-(3-oxo-3-(1-(2-oxo-2*H*-chromen-3-yl)-1*H*-dibenzo[b,f][1,2,3]triazolo[4,5-d]azocin-8(9*H*)-yl)propyl)acetamide 11a (CDCl_3_). (TIF)

S63 Fig ^19^F NMR spectrum of 2,2,2-trifluoro-*N*-(3-oxo-3-(1-(2-oxo-2*H*-chromen-3-yl)-1*H*-dibenzo[b,f][1,2,3]triazolo[4,5-d]azocin-8(9*H*)-yl)propyl)acetamide 11a (CDCl_3_). (TIF)

S64 Fig ^1^H – ^1^H COSY NMR spectrum of 2,2,2-trifluoro-*N*-(3-oxo-3-(1-(2-oxo-2*H*-chromen-3-yl)-1*H*-dibenzo[b,f][1,2,3]triazolo[4,5-d]azocin-8(9*H*)-yl)propyl)acetamide 11a (CDCl_3_). (TIF)

S65 Fig ^1^H – ^13^C HMQC NMR spectrum of 2,2,2-trifluoro-*N*-(3-oxo-3-(1-(2-oxo-2*H*-chromen-3-yl)-1*H*-dibenzo[b,f][1,2,3]triazolo[4,5-d]azocin-8(9*H*)-yl)propyl)acetamide 11a (CDCl_3_). (TIF)

S66 Fig ^1^H – ^13^C HMBC NMR spectrum of 2,2,2-trifluoro-*N*-(3-oxo-3-(1-(2-oxo-2*H*-chromen-3-yl)-1*H*-dibenzo[b,f][1,2,3]triazolo[4,5-d]azocin-8(9*H*)-yl)propyl)acetamide 11a (CDCl_3_). (TIF)

S67 Fig ^1^H – ^15^N HMBC NMR spectrum of 2,2,2-trifluoro-*N*-(3-oxo-3-(1-(2-oxo-2*H*-chromen-3-yl)-1*H*-dibenzo[b,f][1,2,3]triazolo[4,5-d]azocin-8(9*H*)-yl)propyl)acetamide 11a (CDCl_3_). (TIF)

S68 Fig ^1^H NMR spectrum of 2,2,2-trifluoro-*N*-(3-oxo-3-(3-(2-oxo-2*H*-chromen-3-yl)-3*H*-dibenzo[b,f][1,2,3]triazolo[4,5-d]azocin-8(9H)-yl)propyl)acetamide 11b (CDCl_3_). (TIF)

S69 Fig ^13^C NMR spectrum of 2,2,2-trifluoro-*N*-(3-oxo-3-(3-(2-oxo-2*H*-chromen-3-yl)-3*H*-dibenzo[b,f][1,2,3]triazolo[4,5-d]azocin-8(9H)-yl)propyl)acetamide 11b (CDCl_3_). (TIF)

S70 Fig ^1^H – ^1^H COSY NMR spectrum of 2,2,2-trifluoro-*N*-(3-oxo-3-(3-(2-oxo-2*H*-chromen-3-yl)-3*H*-dibenzo[b,f][1,2,3]triazolo[4,5-d]azocin-8(9H)-yl)propyl)acetamide 11b (CDCl_3_). (TIF)

S71 Fig ^1^H – ^13^C HMQC NMR spectrum of 2,2,2-trifluoro-*N*-(3-oxo-3-(3-(2-oxo-2*H*-chromen-3-yl)-3*H*-dibenzo[b,f][1,2,3]triazolo[4,5-d]azocin-8(9H)-yl)propyl)acetamide 11b (CDCl_3_). (TIF)

S72 Fig ^1^H – ^13^C HMBC NMR spectrum of 2,2,2-trifluoro-*N*-(3-oxo-3-(3-(2-oxo-2*H*-chromen-3-yl)-3*H*-dibenzo[b,f][1,2,3]triazolo[4,5-d]azocin-8(9H)-yl)propyl)acetamide 11b (CDCl_3_). (TIF)

S73 Fig HPLC chromatogram of mixture of regioisomers 12a and 12b. (TIF)

S74 Fig ^1^H NMR spectrum of 2-((benzoyloxy)methyl)-5-(2,4-dioxo-5-((8-(3-(2,2,2-trifluoroacetamido)propanoyl)-8,9-dihydro-1*H*-dibenzo[b,f][1,2,3]triazolo[4,5-d]azocin-1-yl)methyl)-3,4-dihydropyrimidin-1(2*H*)-yl)tetrahydrofuran-3,4-diyl dibenzoate 12a and 2-((benzoyloxy)methyl)-5-(2,4-dioxo-5-((8-(3-(2,2,2-trifluoroacetamido)propanoyl)-8,9-dihydro-3*H*-dibenzo[b,f][1,2,3]triazolo[4,5-d]azocin-3-yl)methyl)-3,4-dihydropyrimidin-1(2*H*)-yl)tetrahydrofuran-3,4-diyl dibenzoate 12b (CDCl_3_). (TIF)

S75 Fig Detail of 1H NMR: Example of conformers presented in the mixture of regioisomers 12a and 12b. (TIF)

S76 Fig ^13^C NMR spectrum 2-((benzoyloxy)methyl)-5-(2,4-dioxo-5-((8-(3-(2,2,2-trifluoroacetamido)propanoyl)-8,9-dihydro-1*H*-dibenzo[b,f][1,2,3]triazolo[4,5-d]azocin-1-yl)methyl)-3,4-dihydropyrimidin-1(2*H*)-yl)tetrahydrofuran-3,4-diyl dibenzoate 12a and 2-((benzoyloxy)methyl)-5-(2,4-dioxo-5-((8-(3-(2,2,2-trifluoroacetamido)propanoyl)-8,9-dihydro-3*H*-dibenzo[b,f][1,2,3]triazolo[4,5-d]azocin-3-yl)methyl)-3,4-dihydropyrimidin-1(2*H*)-yl)tetrahydrofuran-3,4-diyl dibenzoate 12b (CDCl_3_). (TIF)

S77 Fig ^1^H NMR spectrum of 2-(5-(Azidomethyl)-2,4-dioxo-3,4-dihydropyrimidin-1(2*H*)-yl)-5-((benzoyloxy)methyl) etrahydrofuran-3,4-diyl dibenzoate. (TIF)

S78 Fig ^13^C NMR spectrum of 2-(5-(Azidomethyl)-2,4-dioxo-3,4-dihydropyrimidin-1(2*H*)-yl)-5-((benzoyloxy)methyl) etrahydrofuran-3,4-diyl dibenzoate (TIF)

S79 Fig The conformers 3, 4 and 5 for the structure 8a. (TIF)

S80 Fig The scheme of the conformation changes for the structure 8b. The float numbers express the energies of the conformers (red number) and the energy barriers of the conformation changes (black number).
(TIF)

S81 Fig The conformers 2–6 for the structure 8b. (TIF)

S82 Fig The scheme of the conformation changes for the structure 9a. The float numbers express the energies of the conformers (red number) and the energy barriers of the conformation changes (black number).
(TIF)

S83 Fig The scheme of the conformation changes for the structure 9b. The float numbers express the energies of the conformers (red number) and the energy barriers of the conformation changes (black number).
(TIF)

S1 Table The fractional and the percentage of population for the local minima of the structure 8a. (TIF)

S2 Table The fractional and the percentage of population for the local minima of the structure 8b. (TIF)

S3 Table The fractional and the percentage of population for the local minima of the structure 9a. (TIF)

S4 Table The fractional and the percentage population for the local minima of the structure 9b. (TIF)
