# Supplementary figures and images for: Non-Catalyzed Click Reactions of ADIBO Derivatives with 5-Methyluridine Azides and Conformational Study of the Resulting Triazoles
Source: PLoS One. 2015 Dec 16;10(12):e0144613. doi: 10.1371/journal.pone.0144613 (PMC4690608; doi:10.1371/journal.pone.0144613)

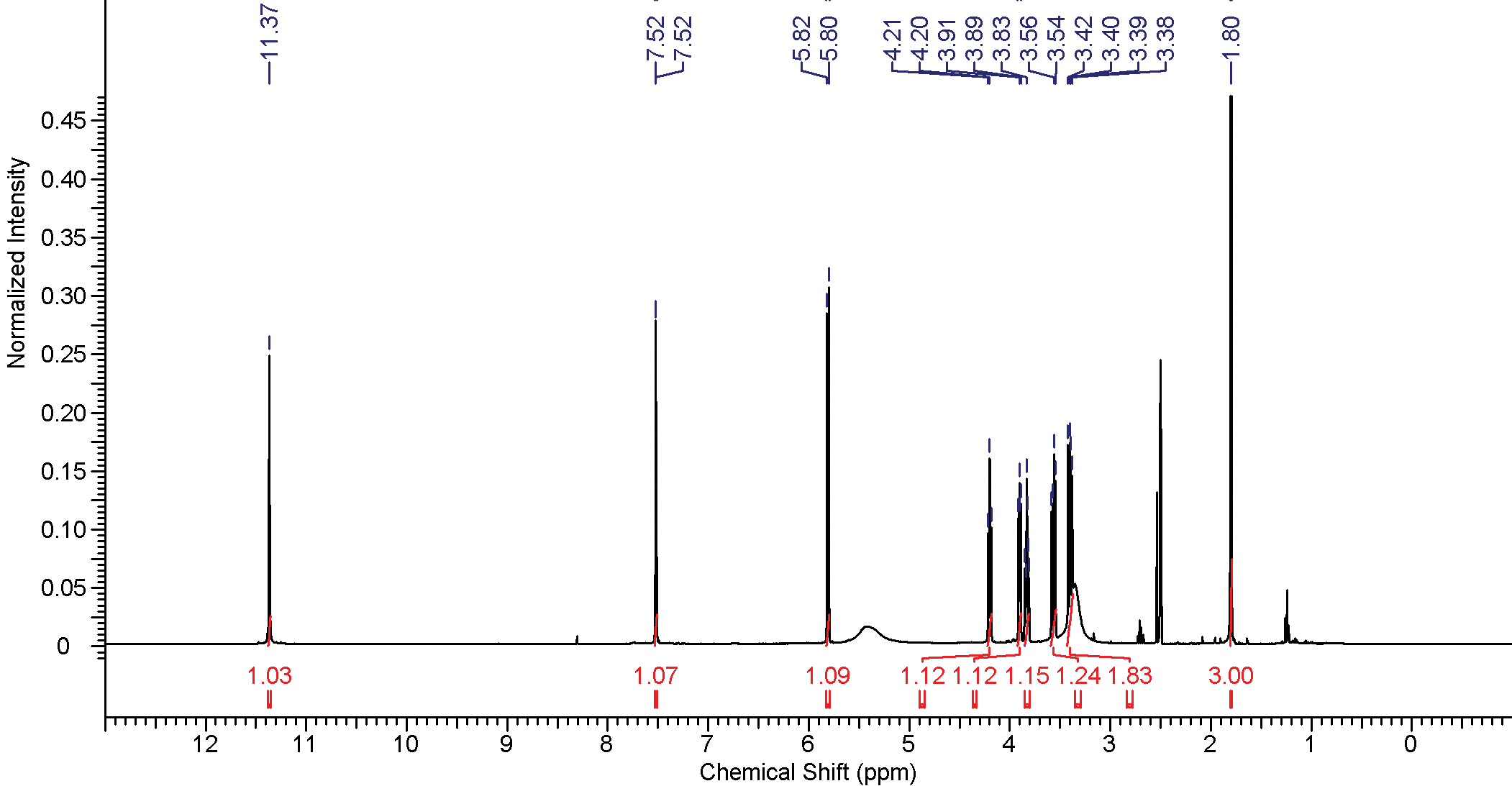

Supplement: S1 Fig — (TIF) [file pone.0144613.s001.tif]

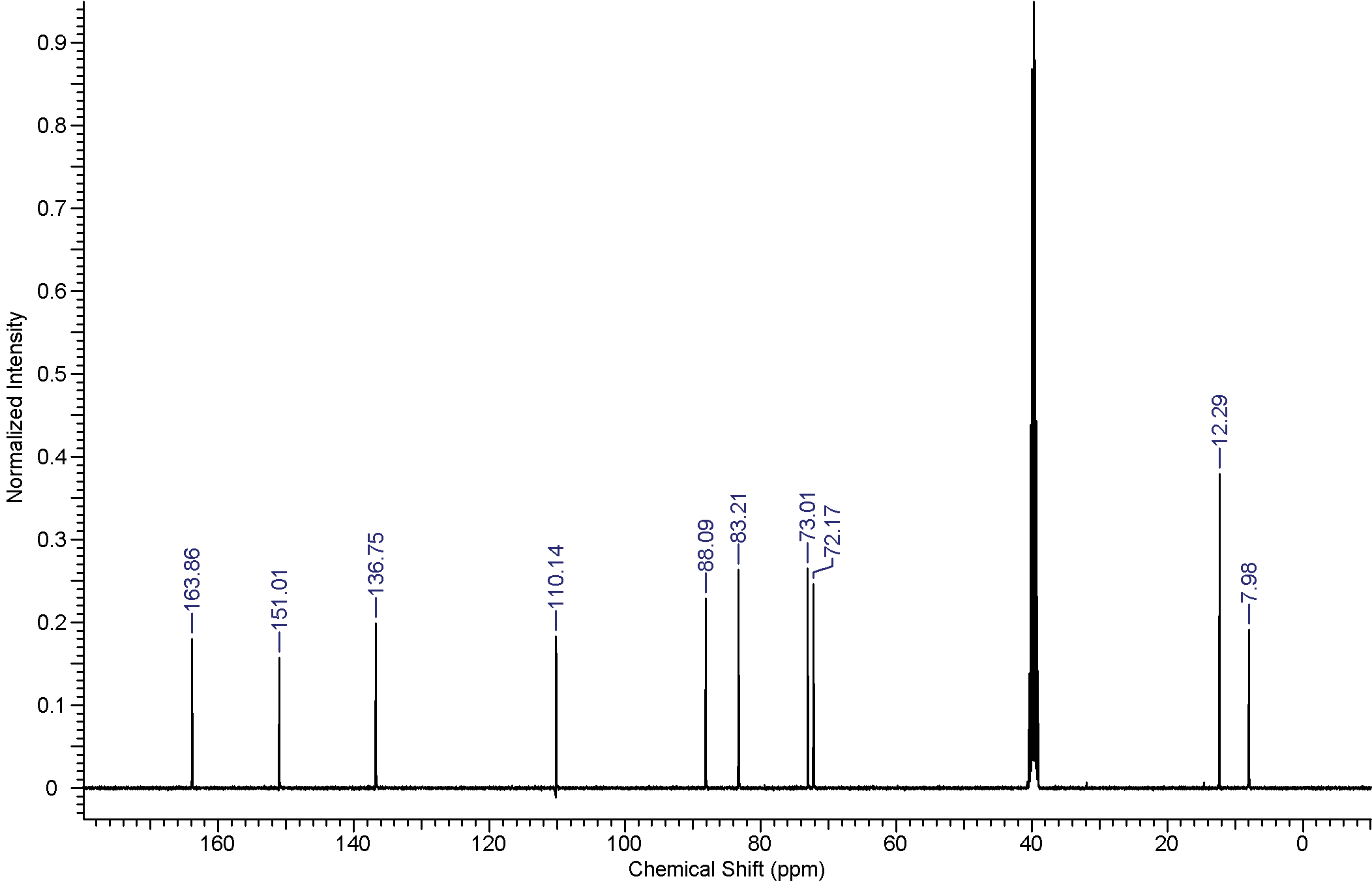

Supplement: S2 Fig — (TIF) [file pone.0144613.s002.tif]

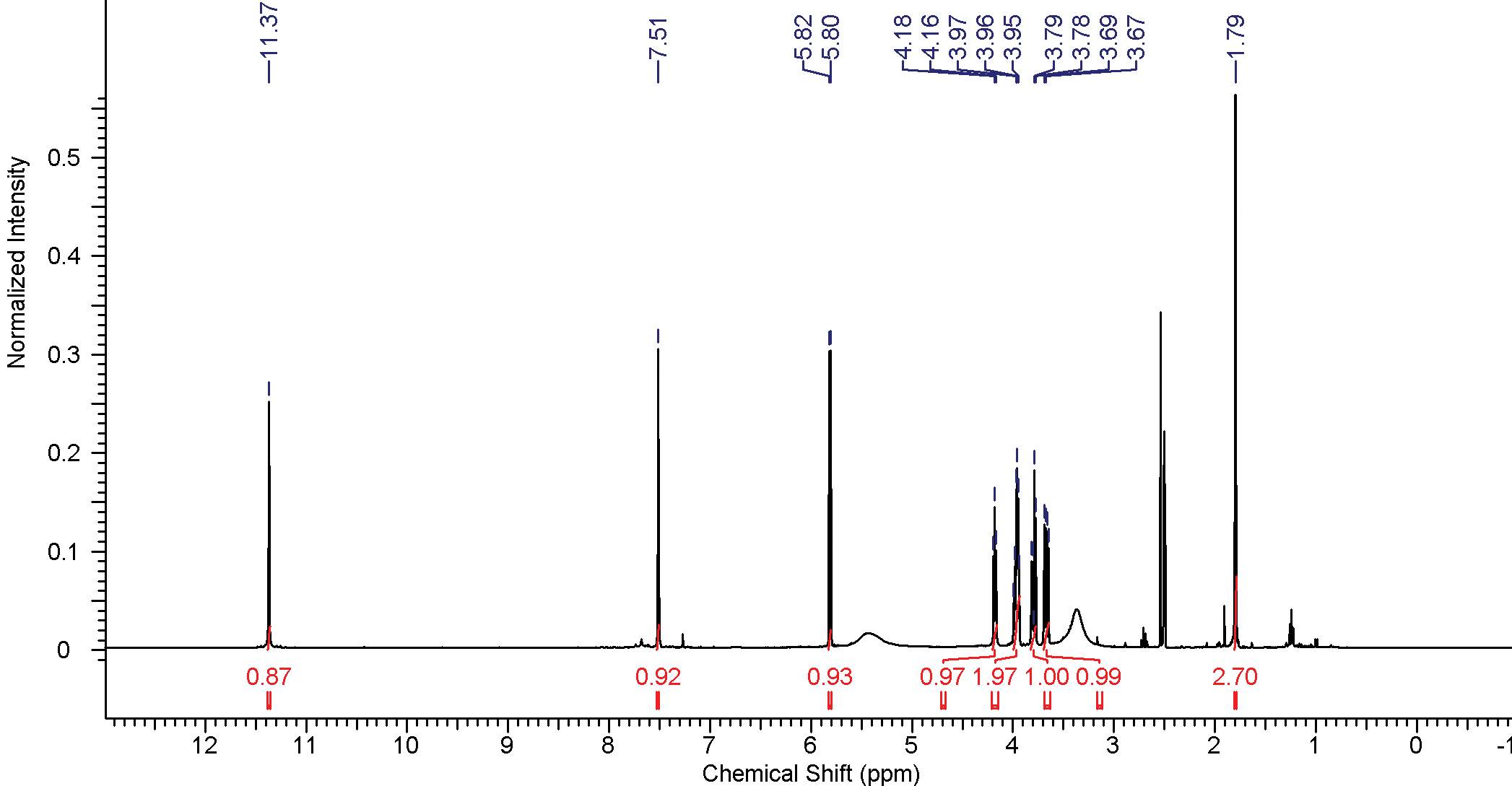

Supplement: S3 Fig — (TIF) [file pone.0144613.s003.tif]

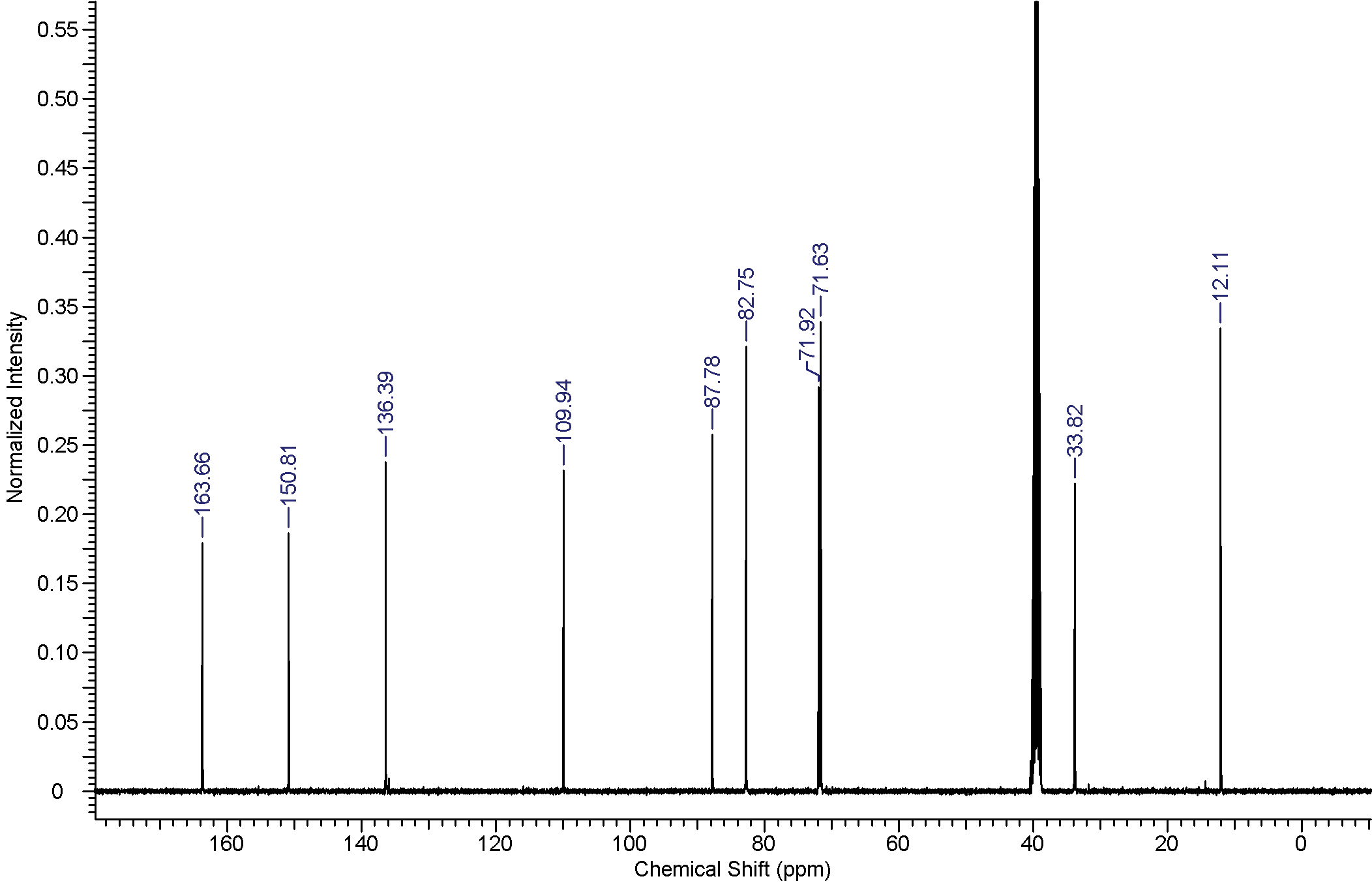

Supplement: S4 Fig — (TIF) [file pone.0144613.s004.tif]

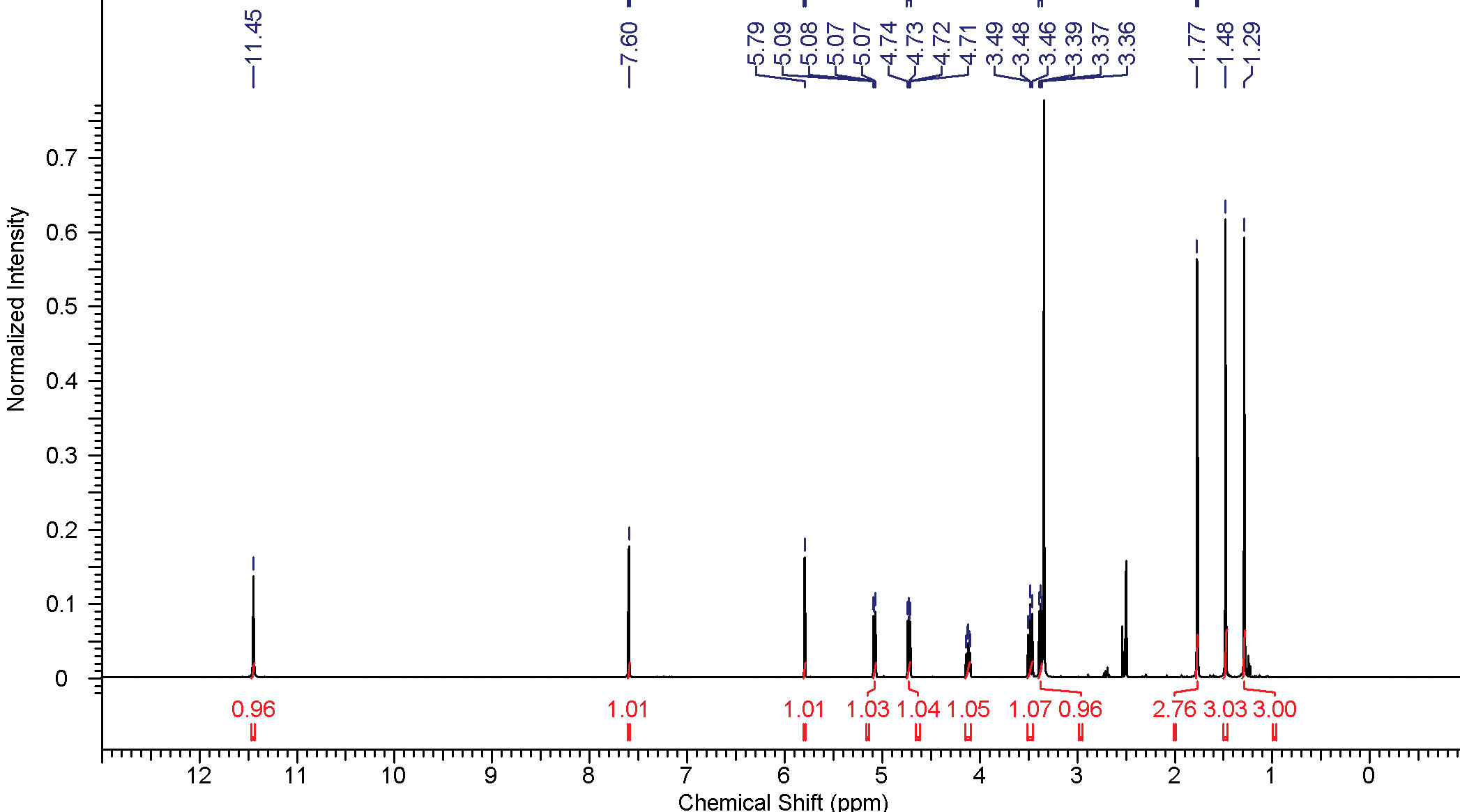

Supplement: S5 Fig — (TIF) [file pone.0144613.s005.tif]

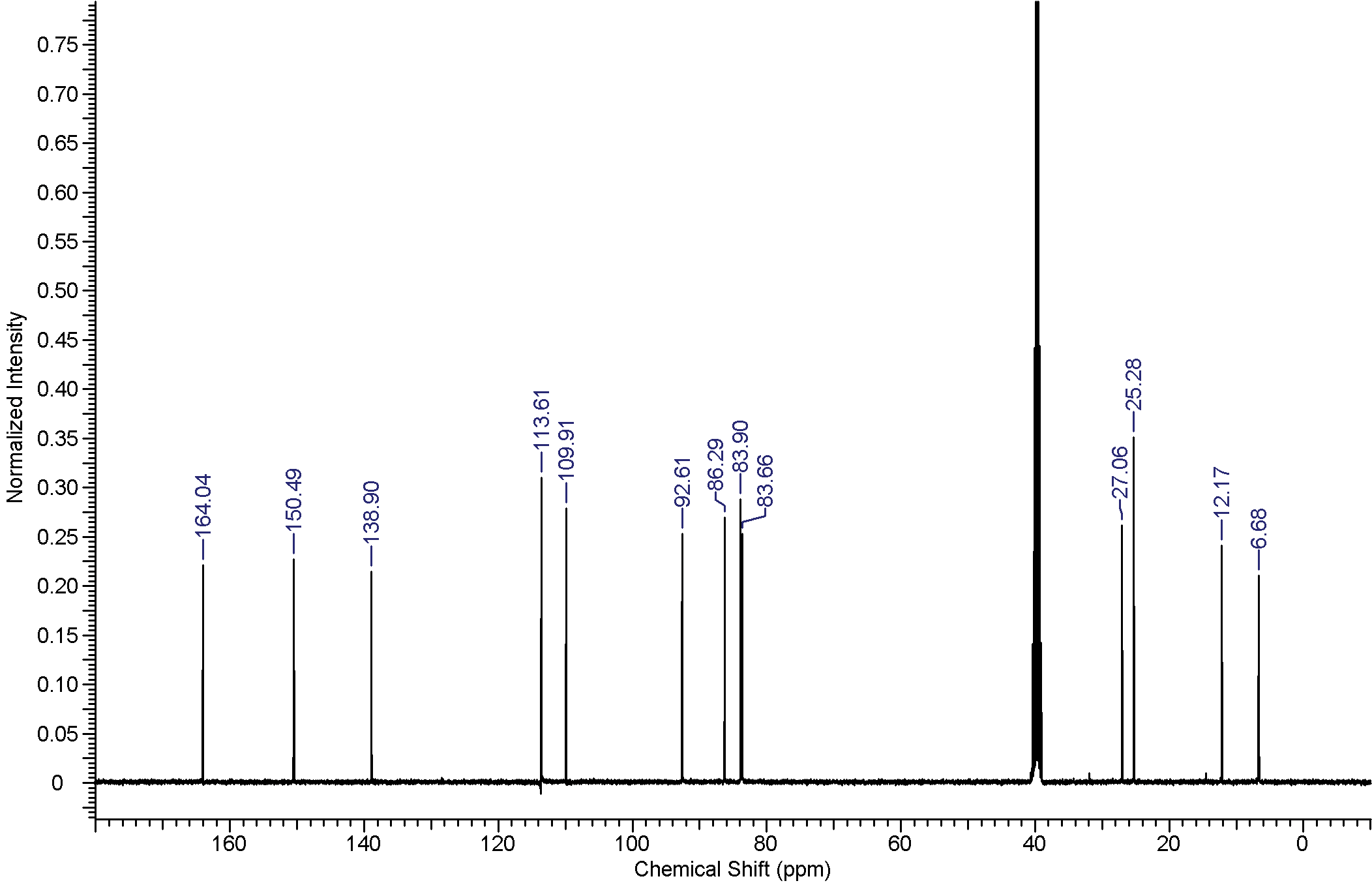

Supplement: S6 Fig — (TIF) [file pone.0144613.s006.tif]

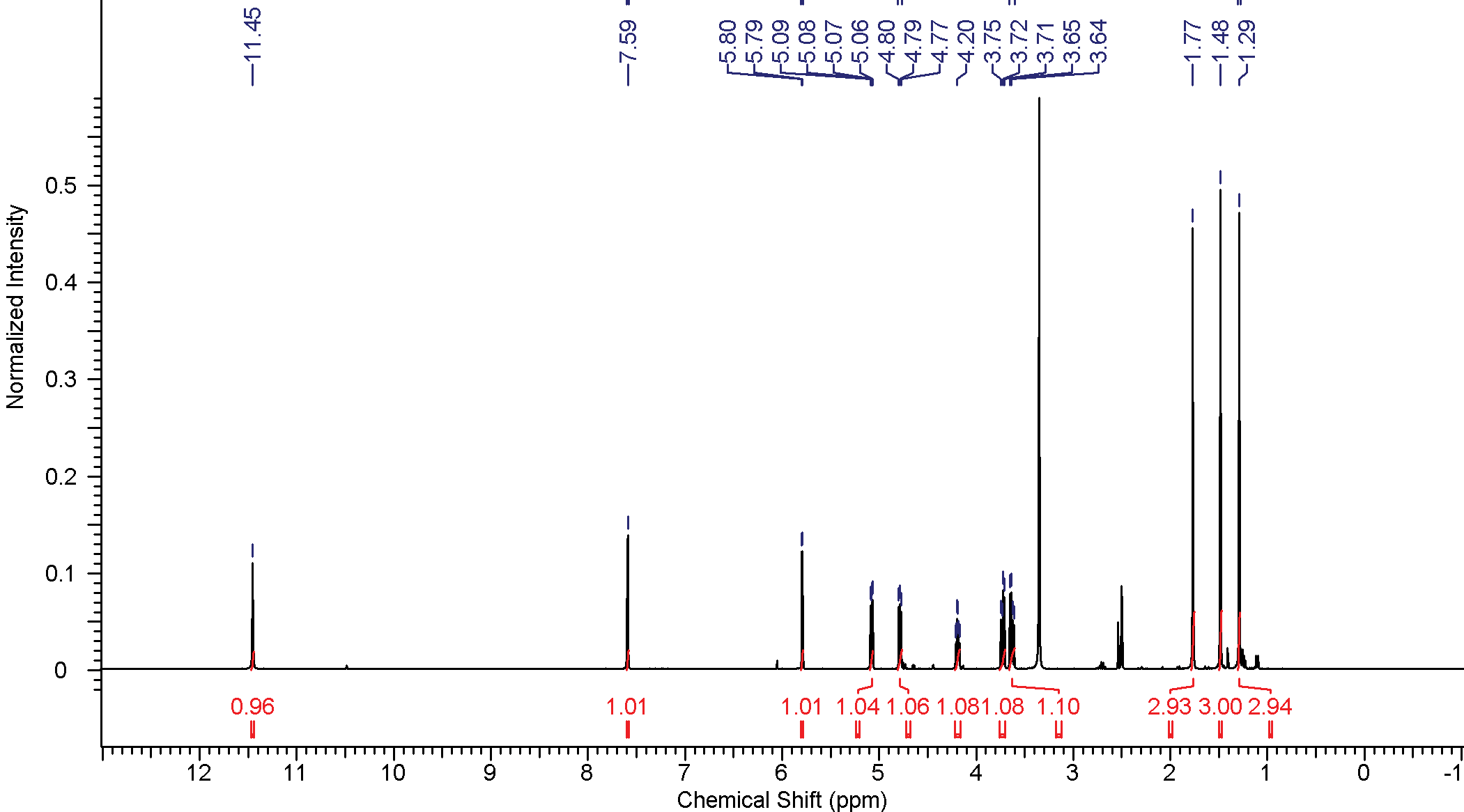

Supplement: S7 Fig — (TIF) [file pone.0144613.s007.tif]

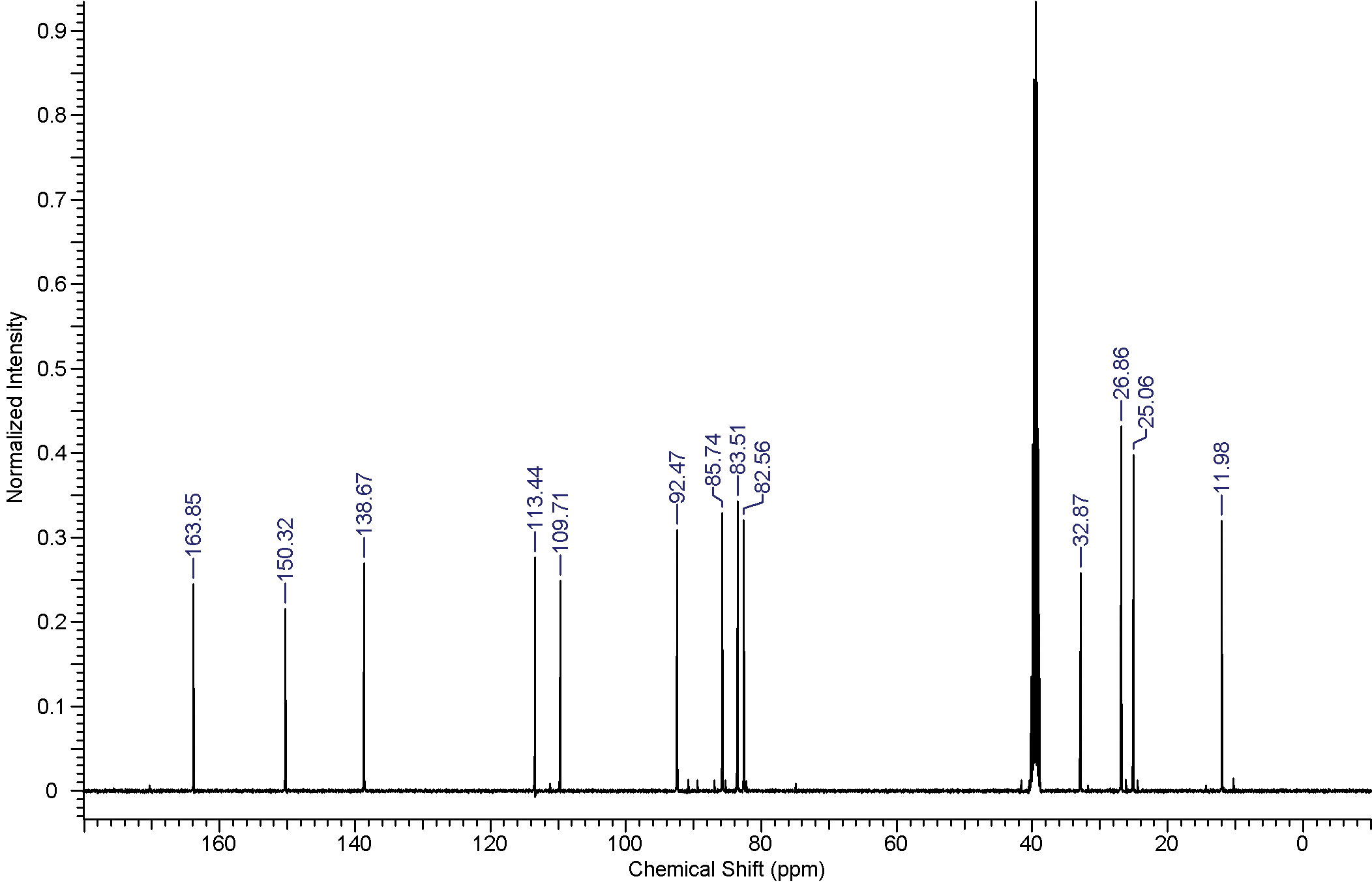

Supplement: S8 Fig — (TIF) [file pone.0144613.s008.tif]

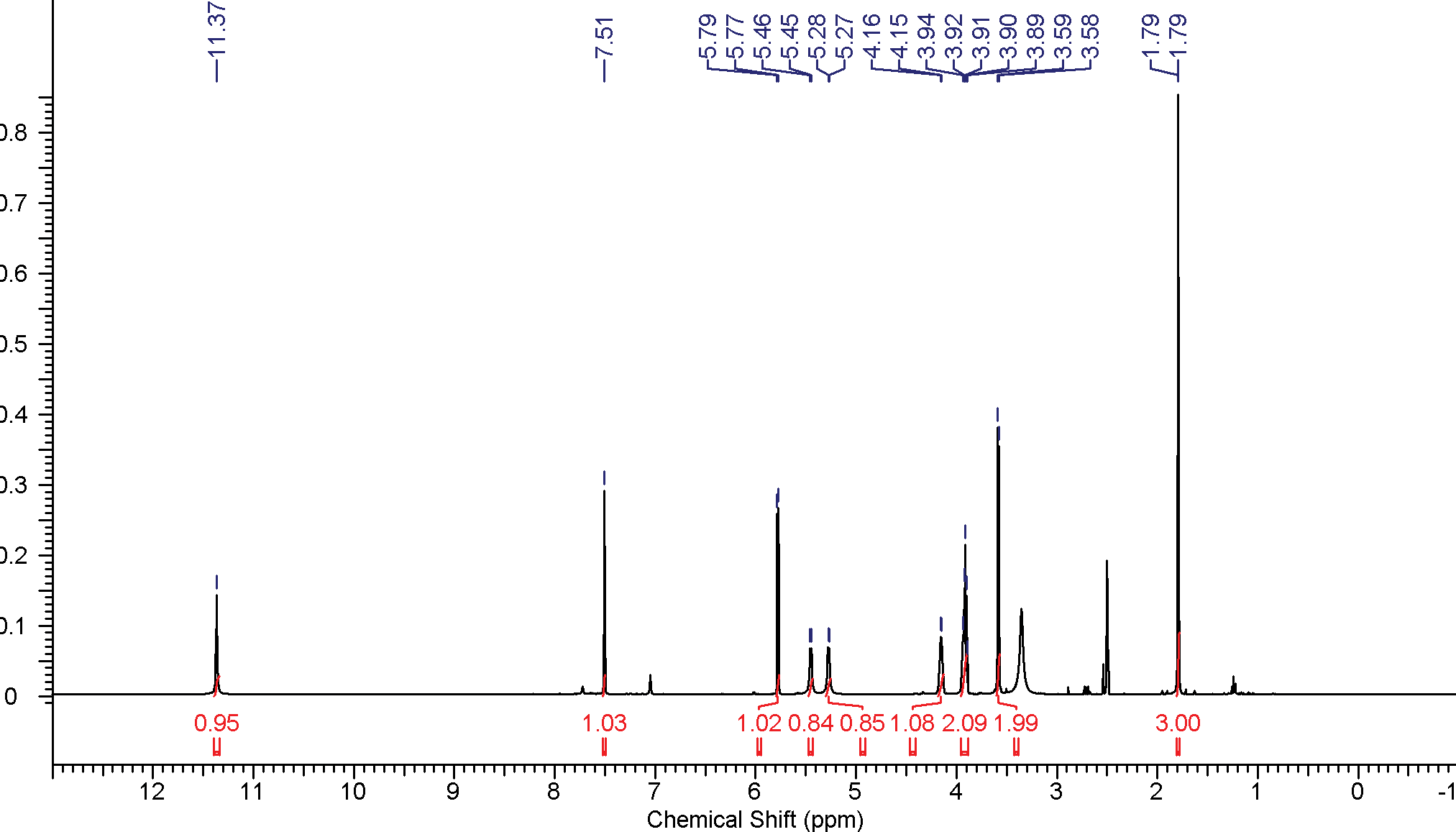

Supplement: S9 Fig — (TIF) [file pone.0144613.s009.tif]

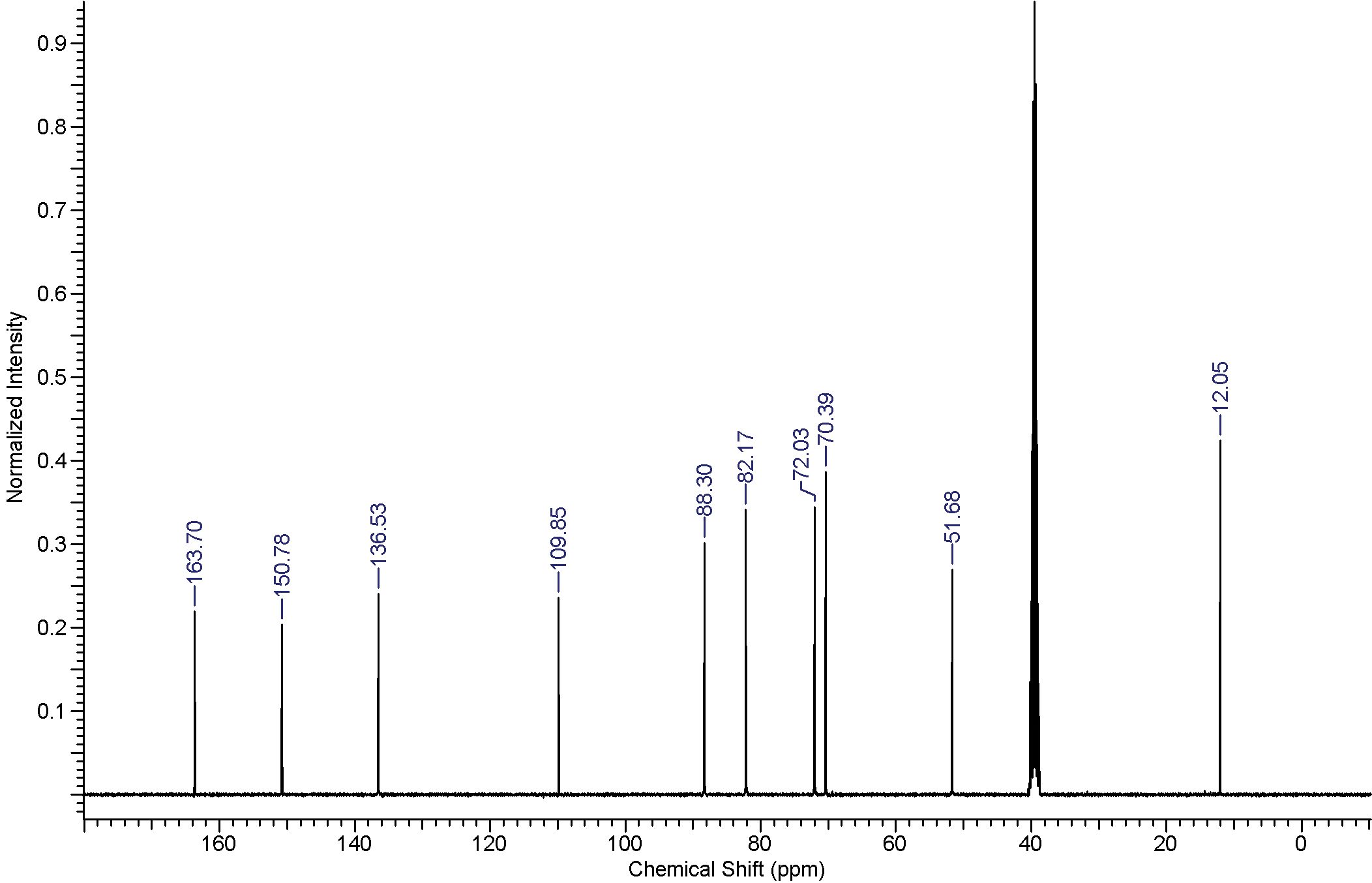

Supplement: S10 Fig — (TIF) [file pone.0144613.s010.tif]

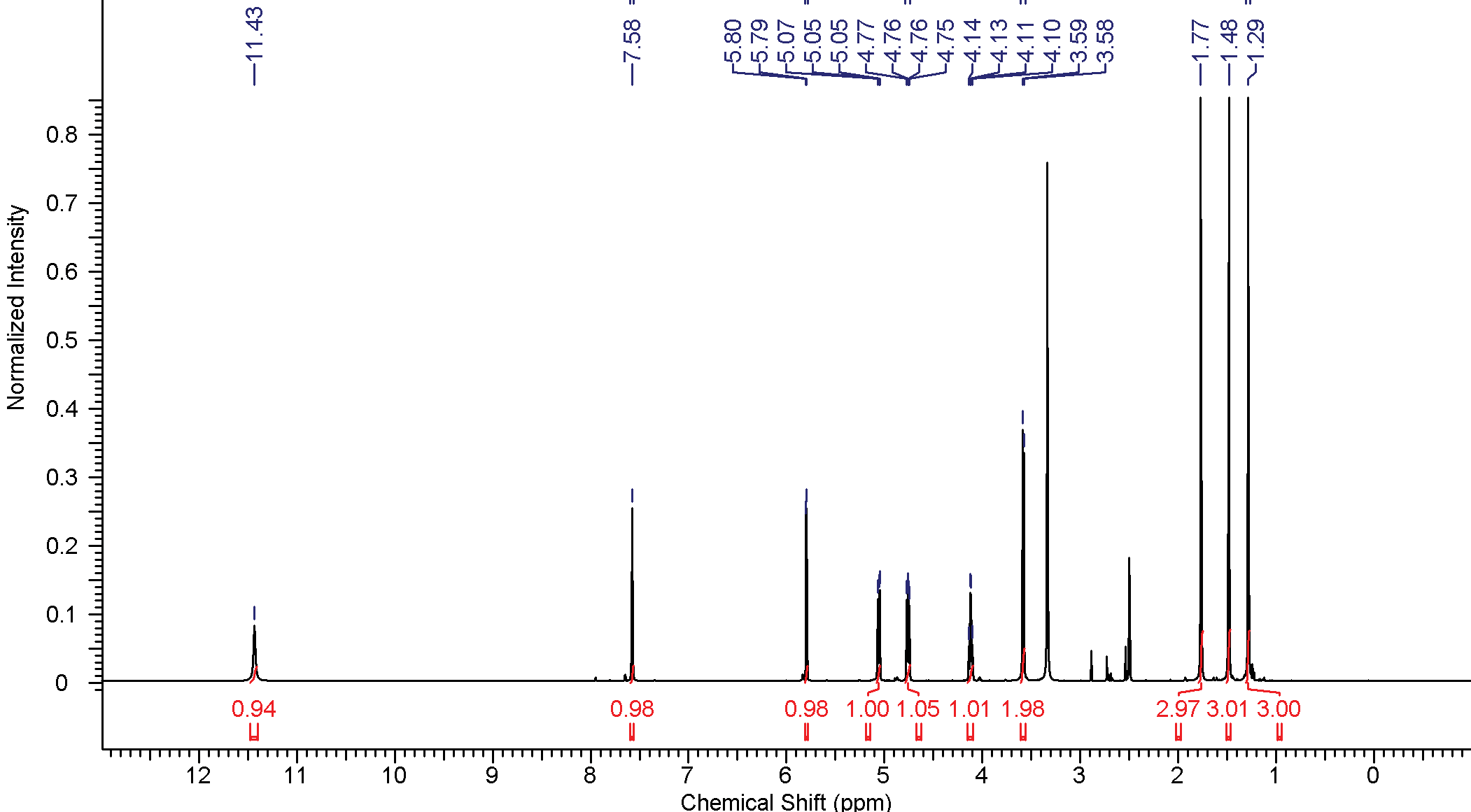

Supplement: S11 Fig — (TIF) [file pone.0144613.s011.tif]

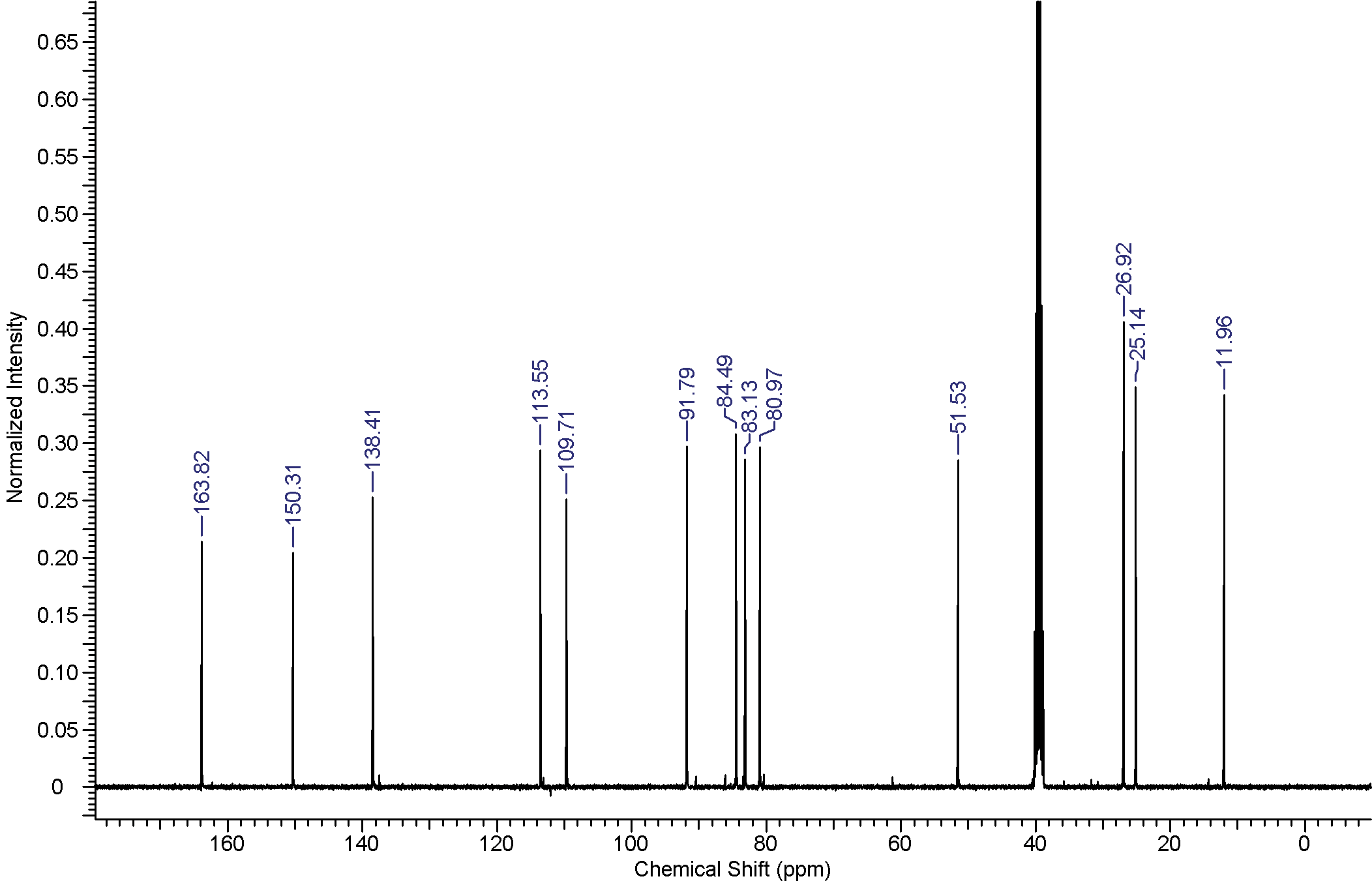

Supplement: S12 Fig — (TIF) [file pone.0144613.s012.tif]

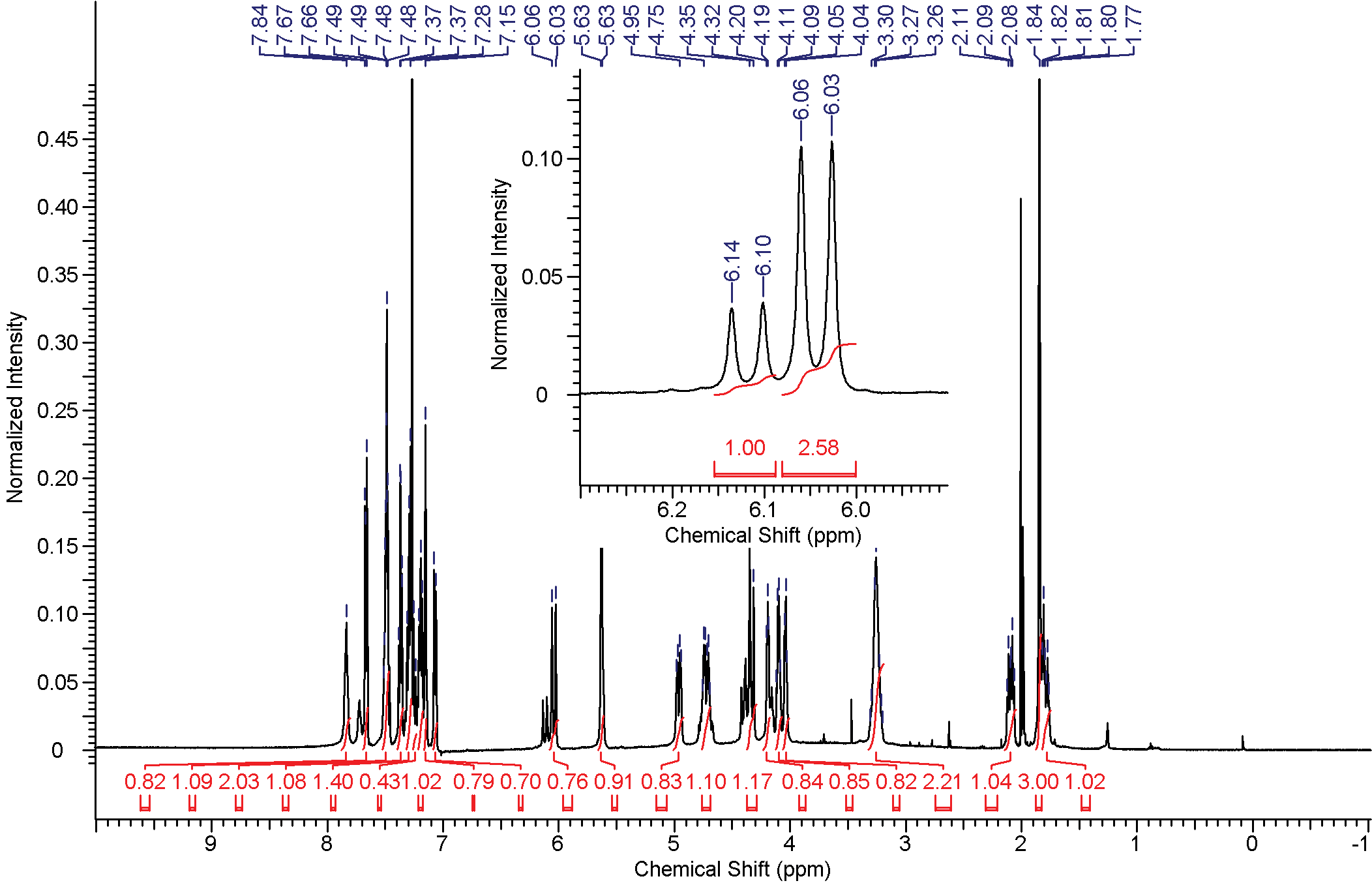

Supplement: S13 Fig — (TIF) [file pone.0144613.s013.tif]

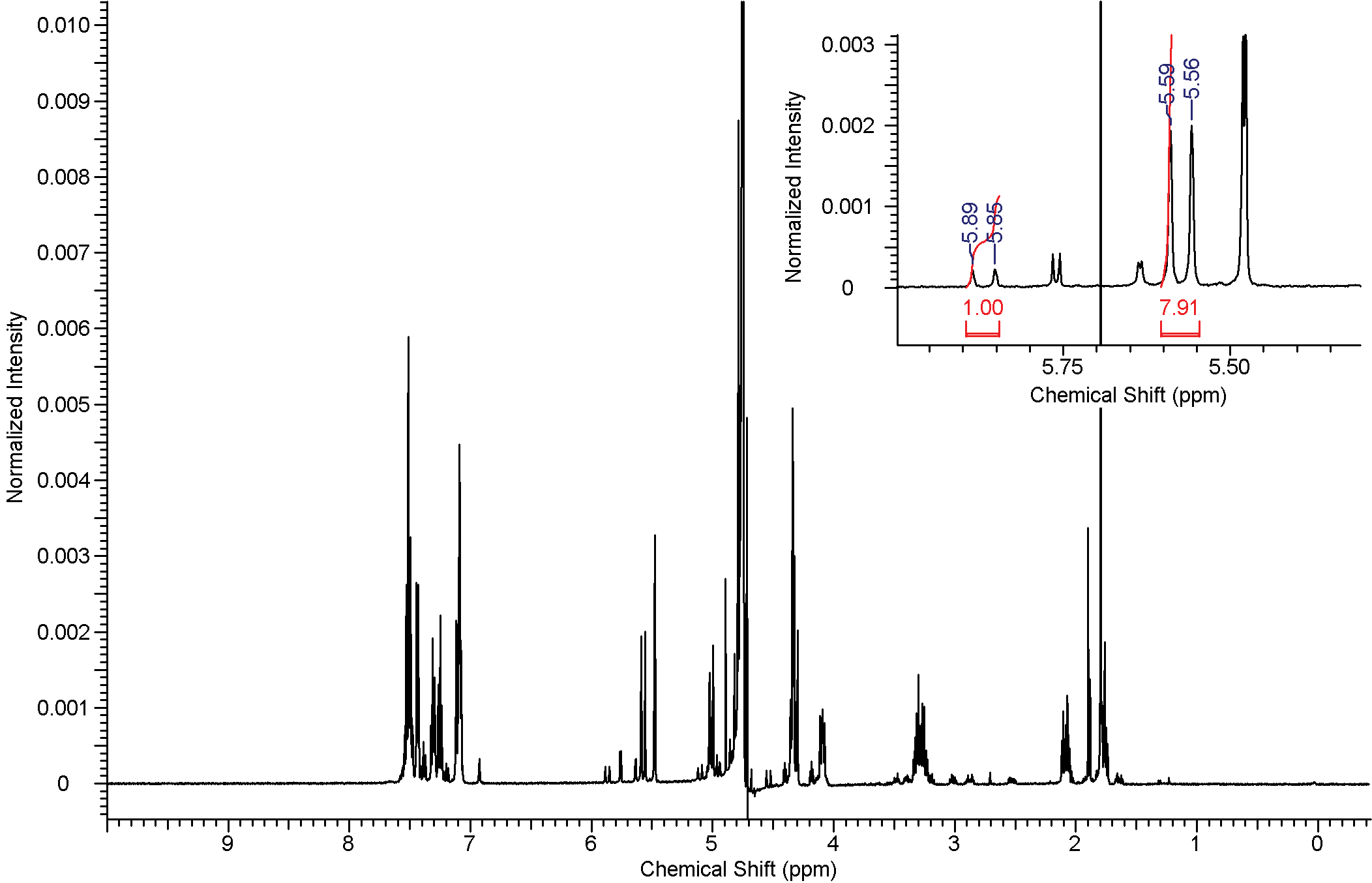

Supplement: S14 Fig — (TIF) [file pone.0144613.s014.tif]

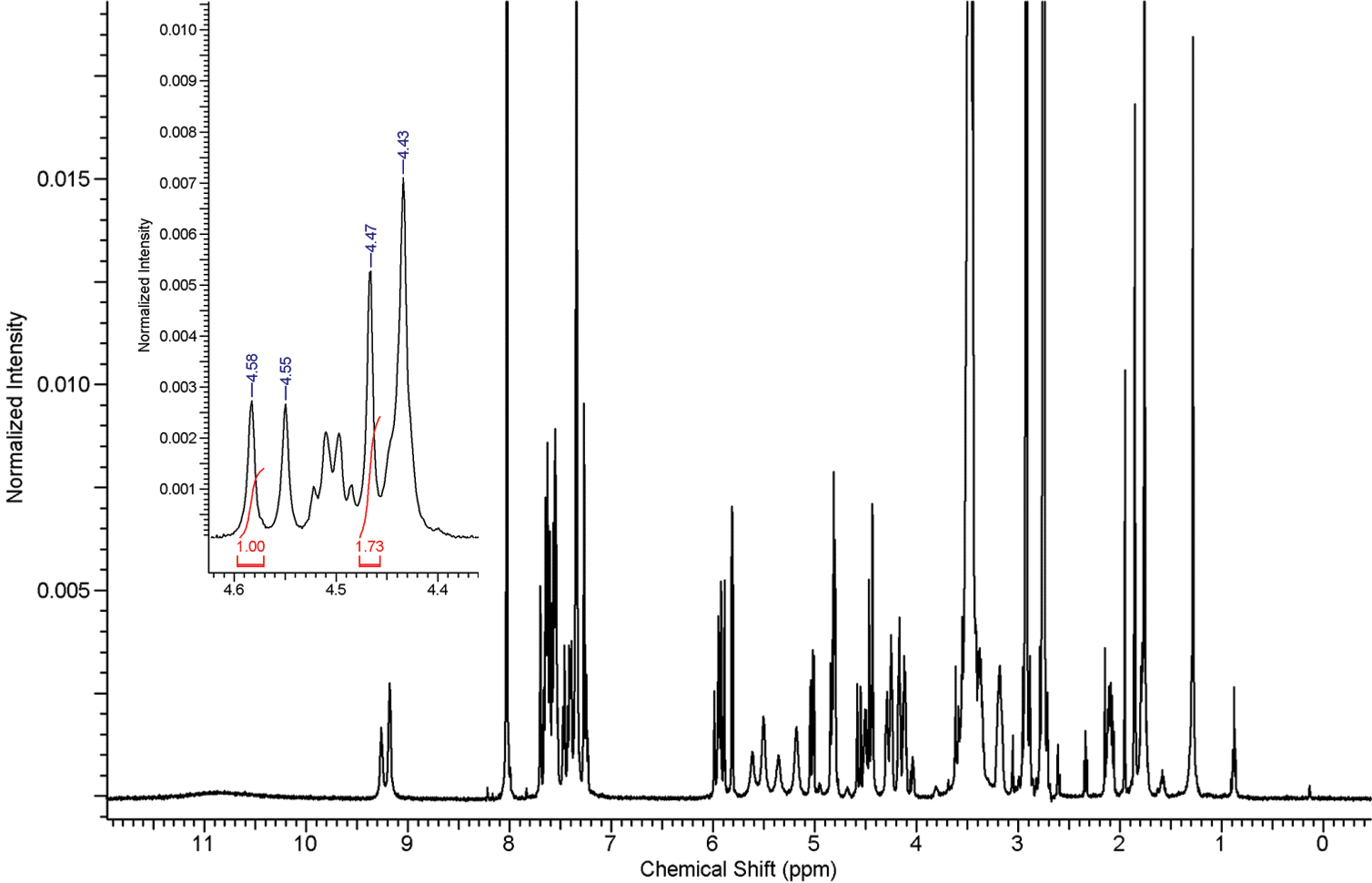

Supplement: S15 Fig — (TIF) [file pone.0144613.s015.tif]

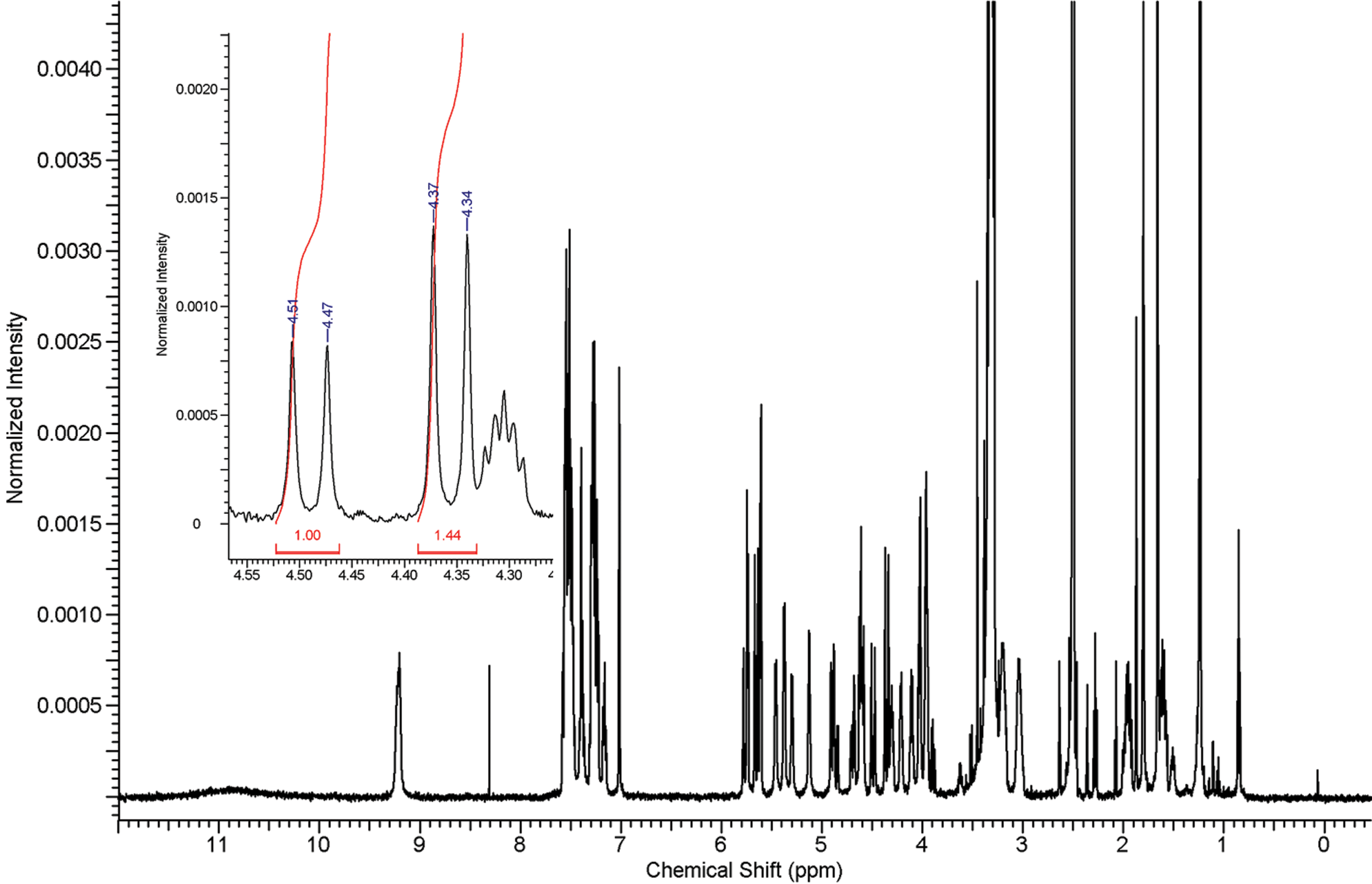

Supplement: S16 Fig — (TIF) [file pone.0144613.s016.tif]

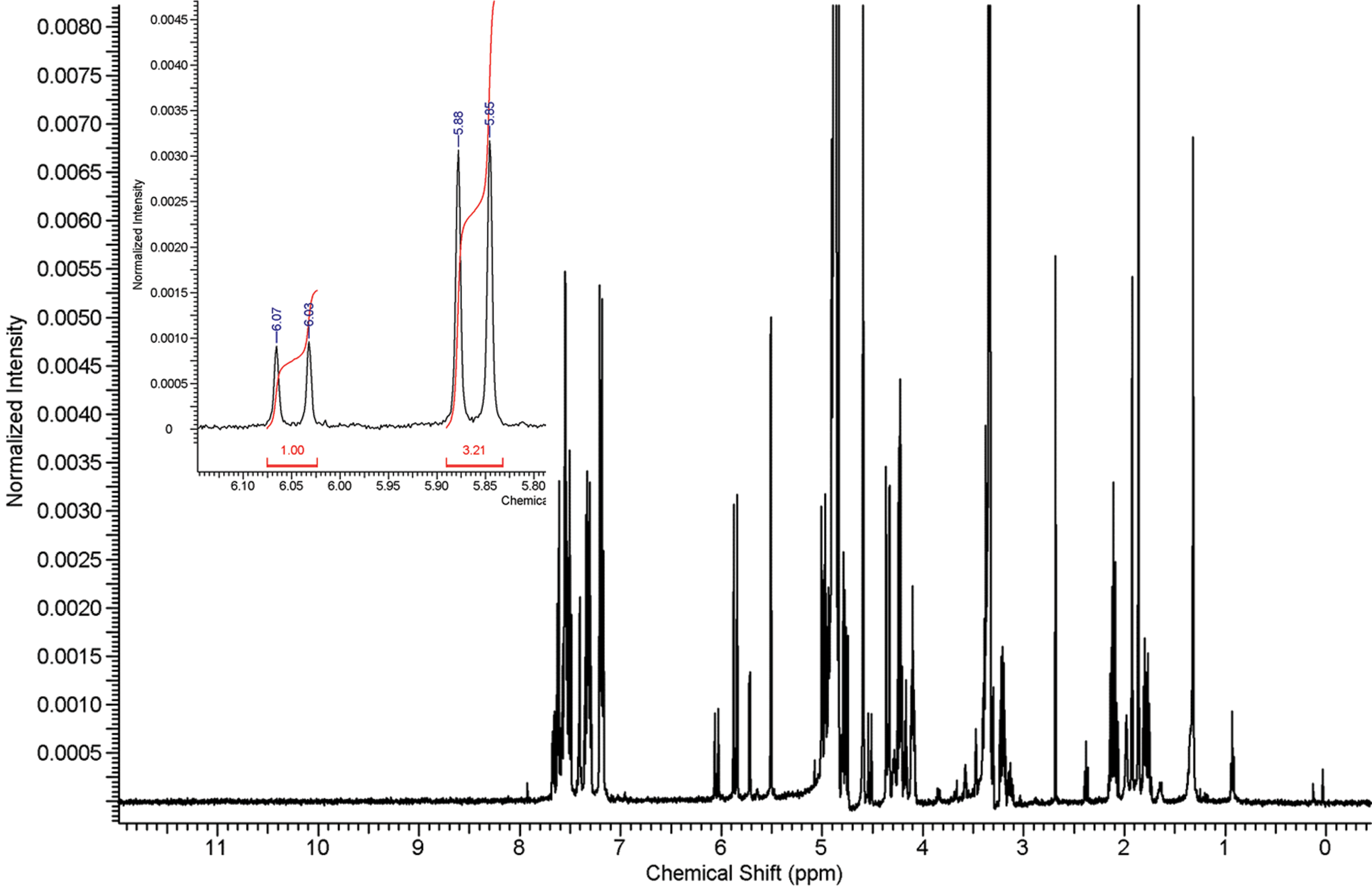

Supplement: S17 Fig — (TIF) [file pone.0144613.s017.tif]

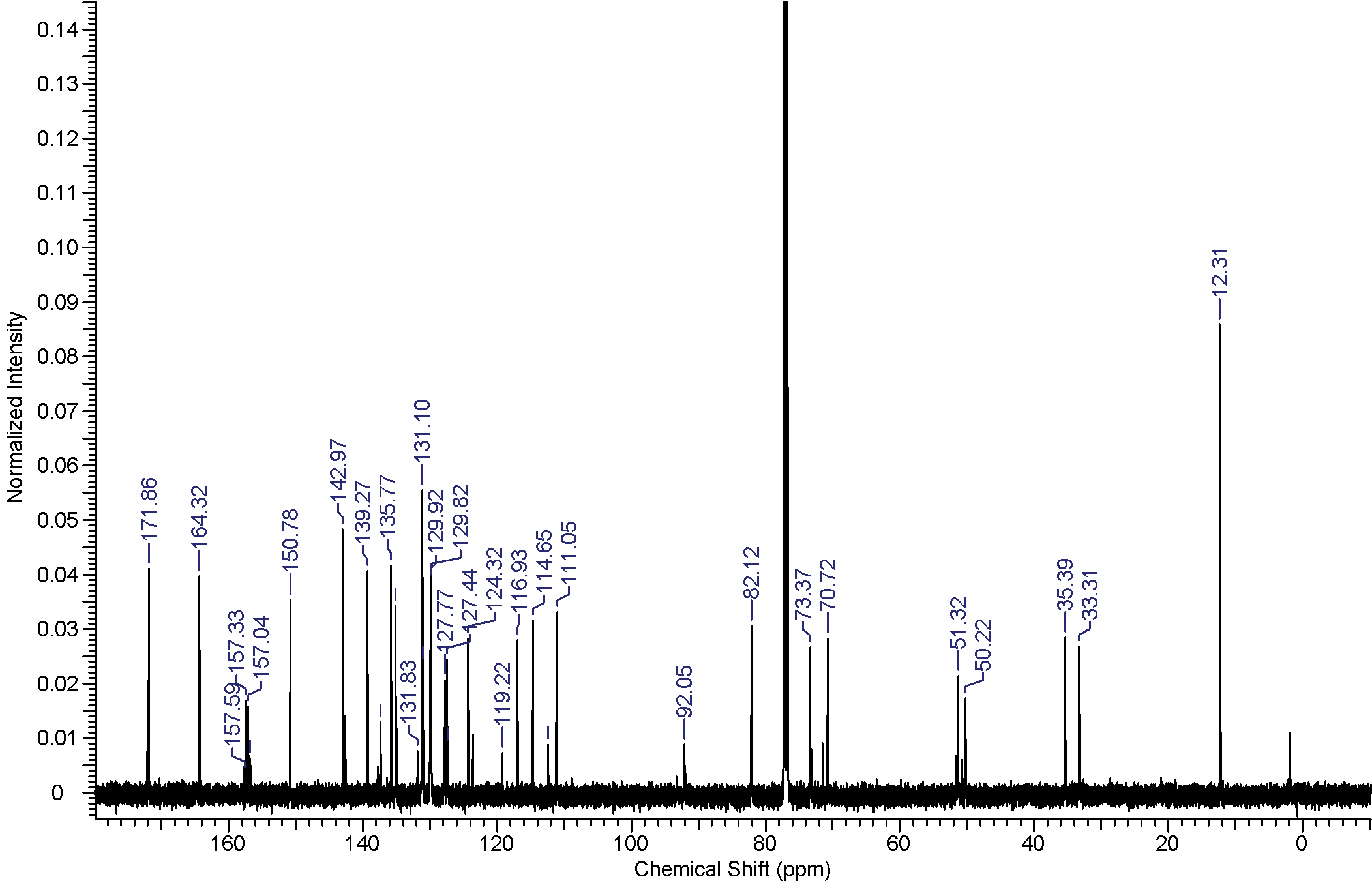

Supplement: S18 Fig — (TIF) [file pone.0144613.s018.tif]

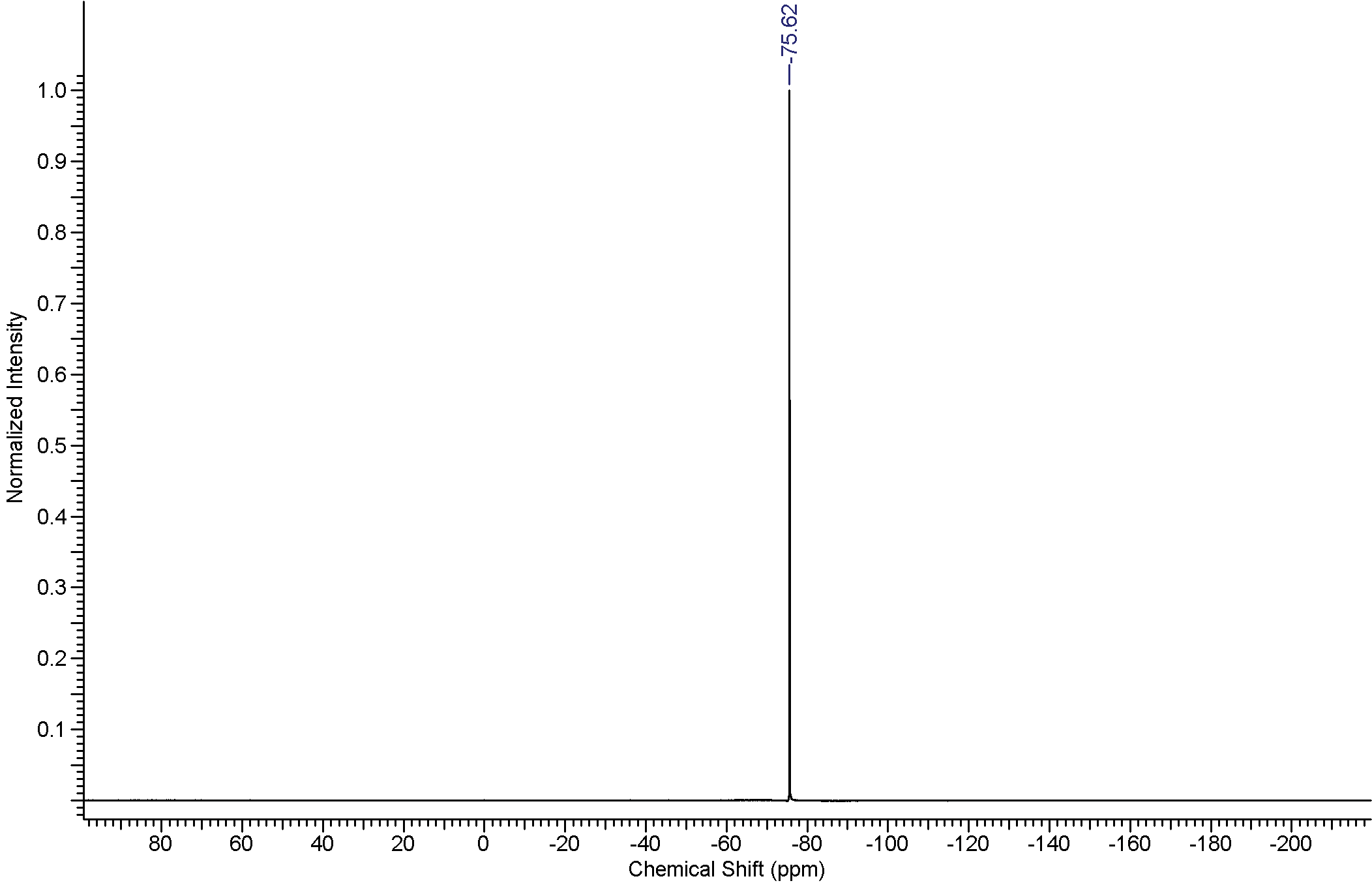

Supplement: S19 Fig — (TIF) [file pone.0144613.s019.tif]

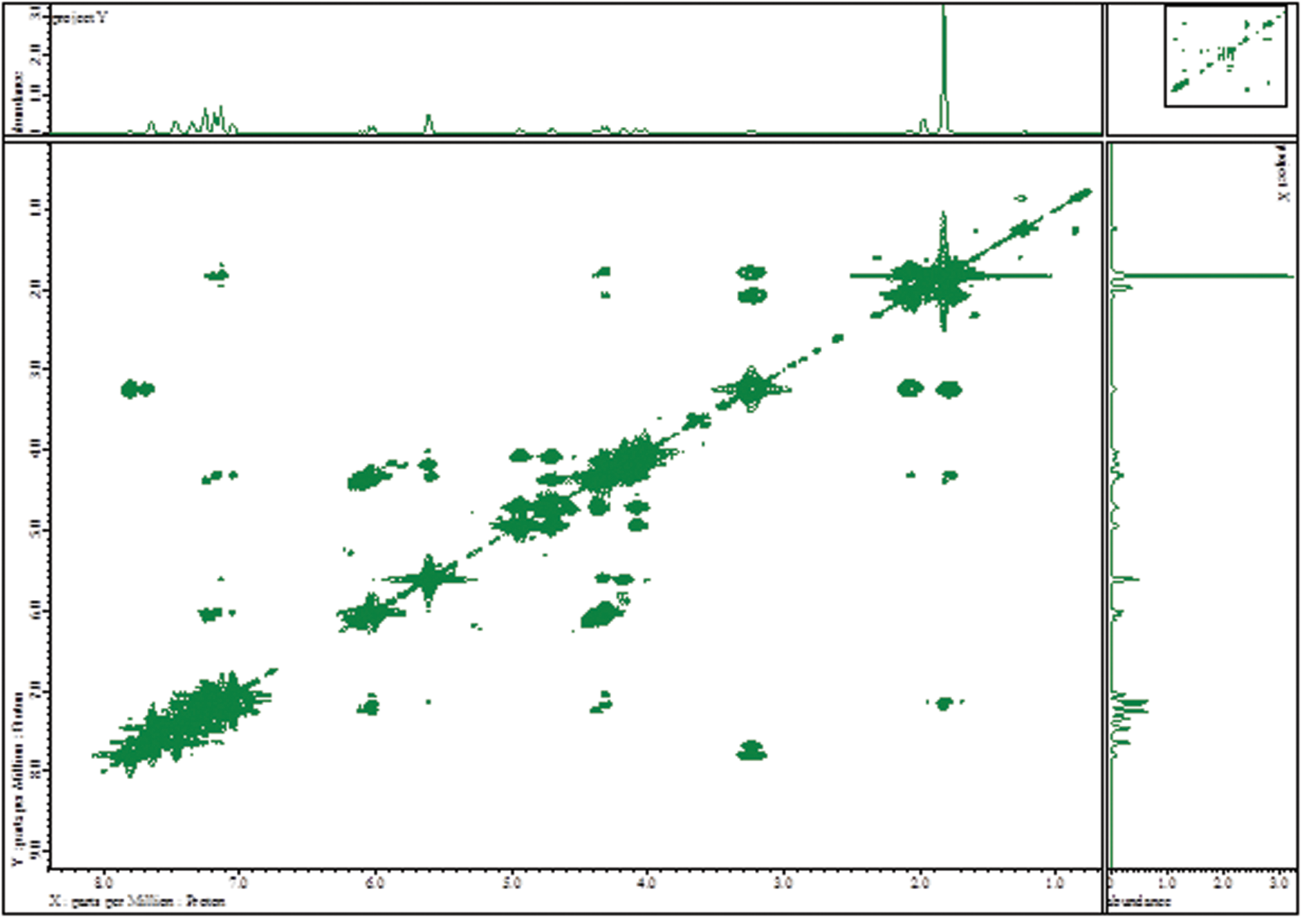

Supplement: S20 Fig — (TIF) [file pone.0144613.s020.tif]

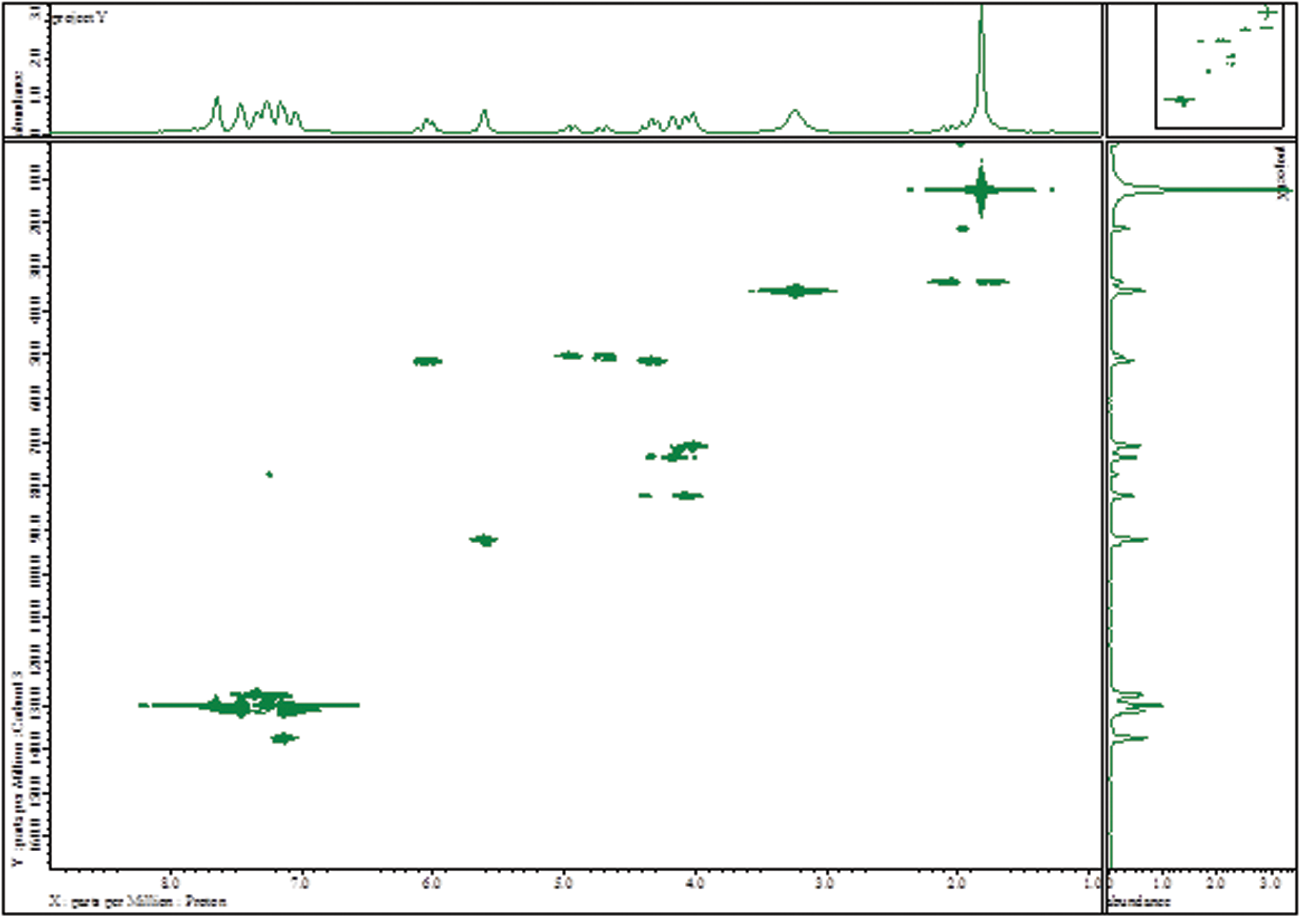

Supplement: S21 Fig — (TIF) [file pone.0144613.s021.tif]

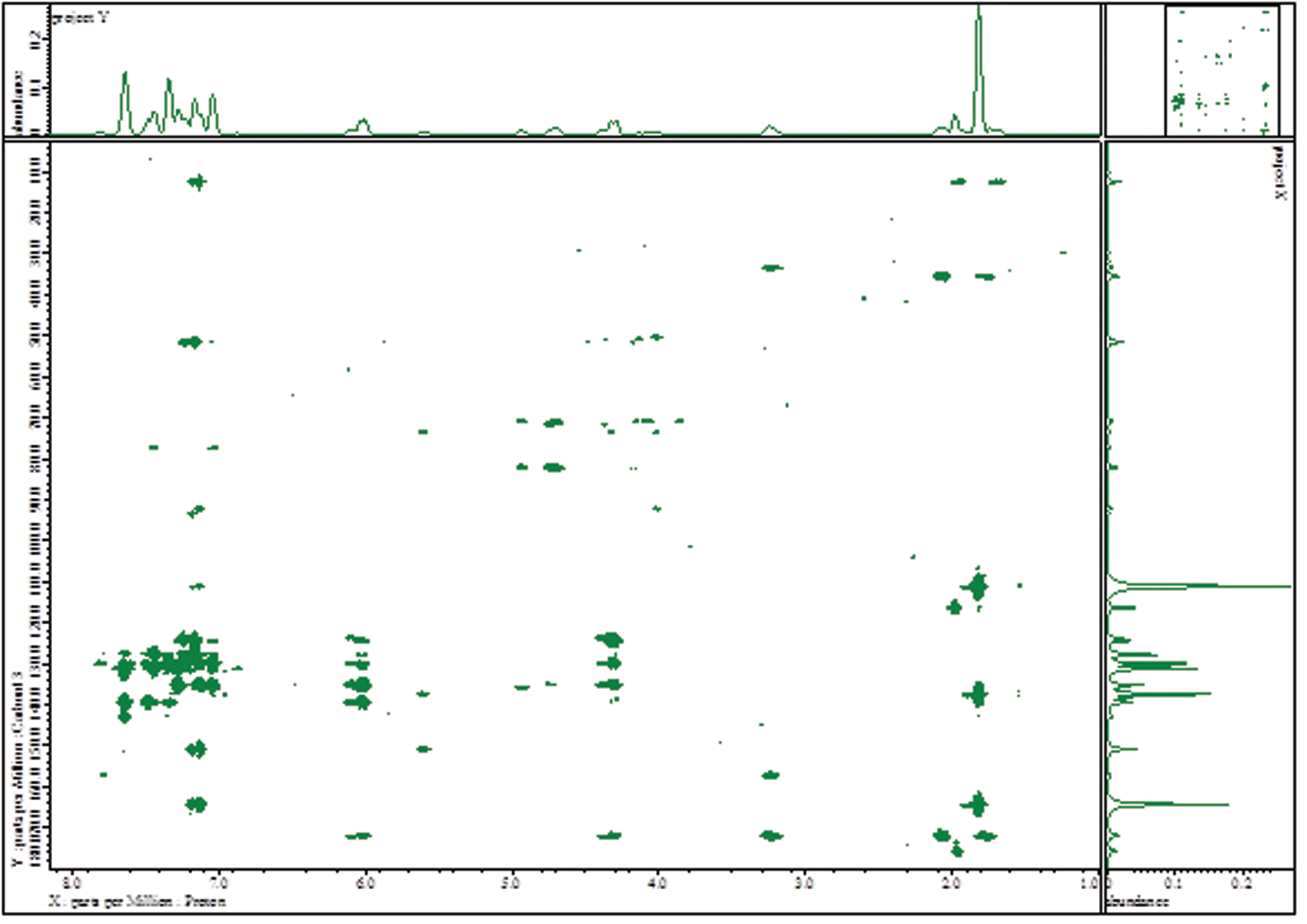

Supplement: S22 Fig — (TIF) [file pone.0144613.s022.tif]

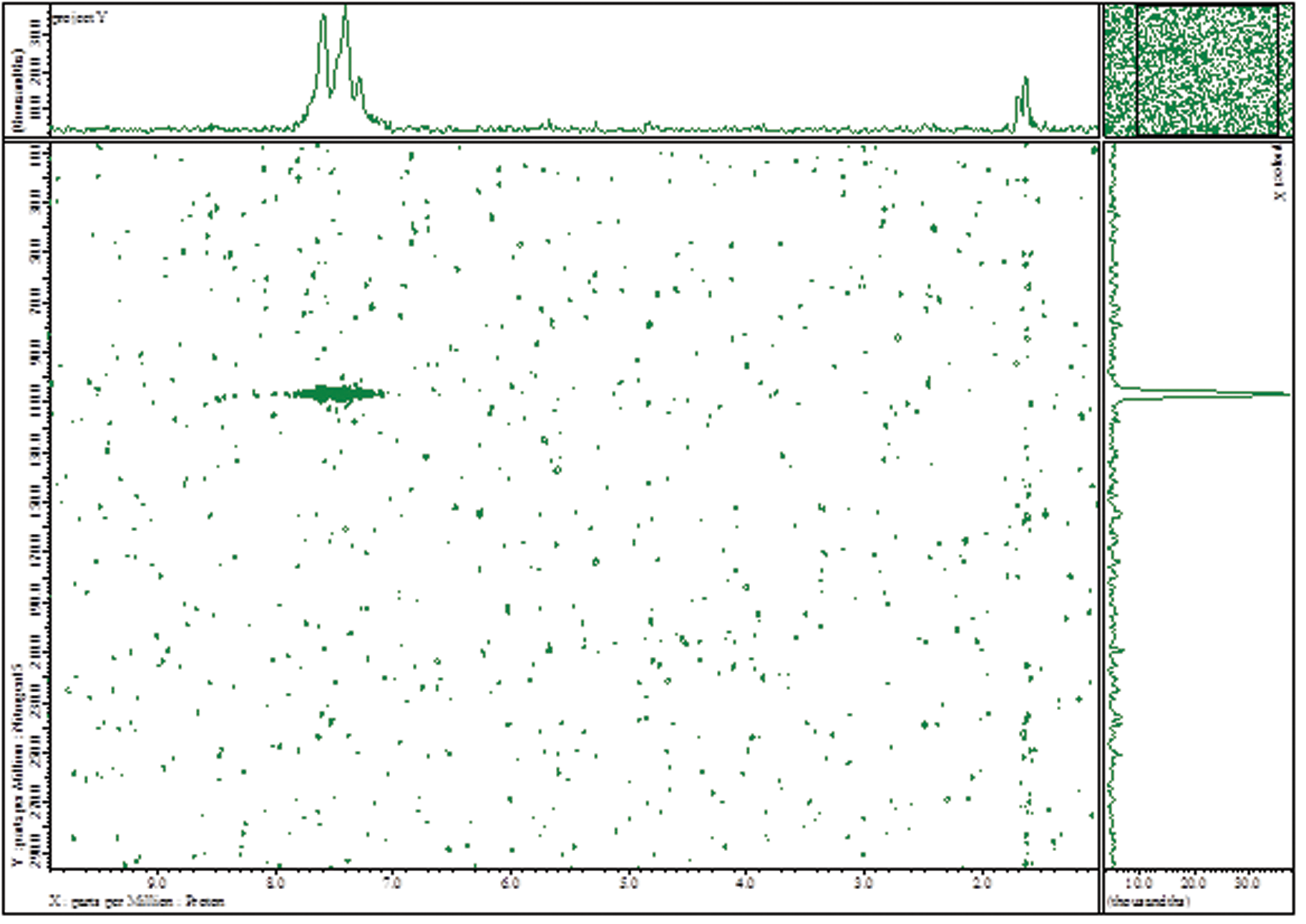

Supplement: S23 Fig — (TIF) [file pone.0144613.s023.tif]

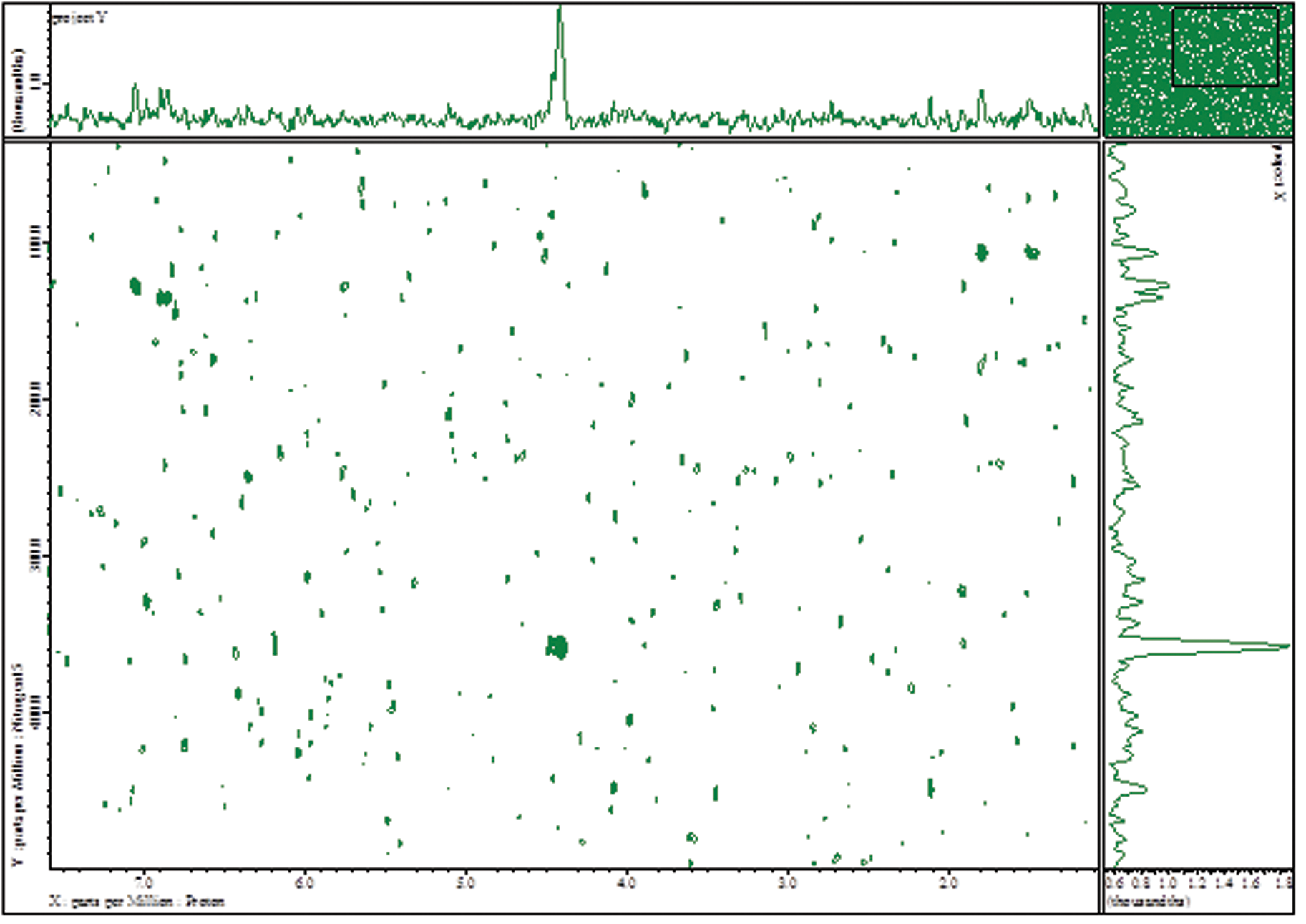

Supplement: S24 Fig — (TIF) [file pone.0144613.s024.tif]

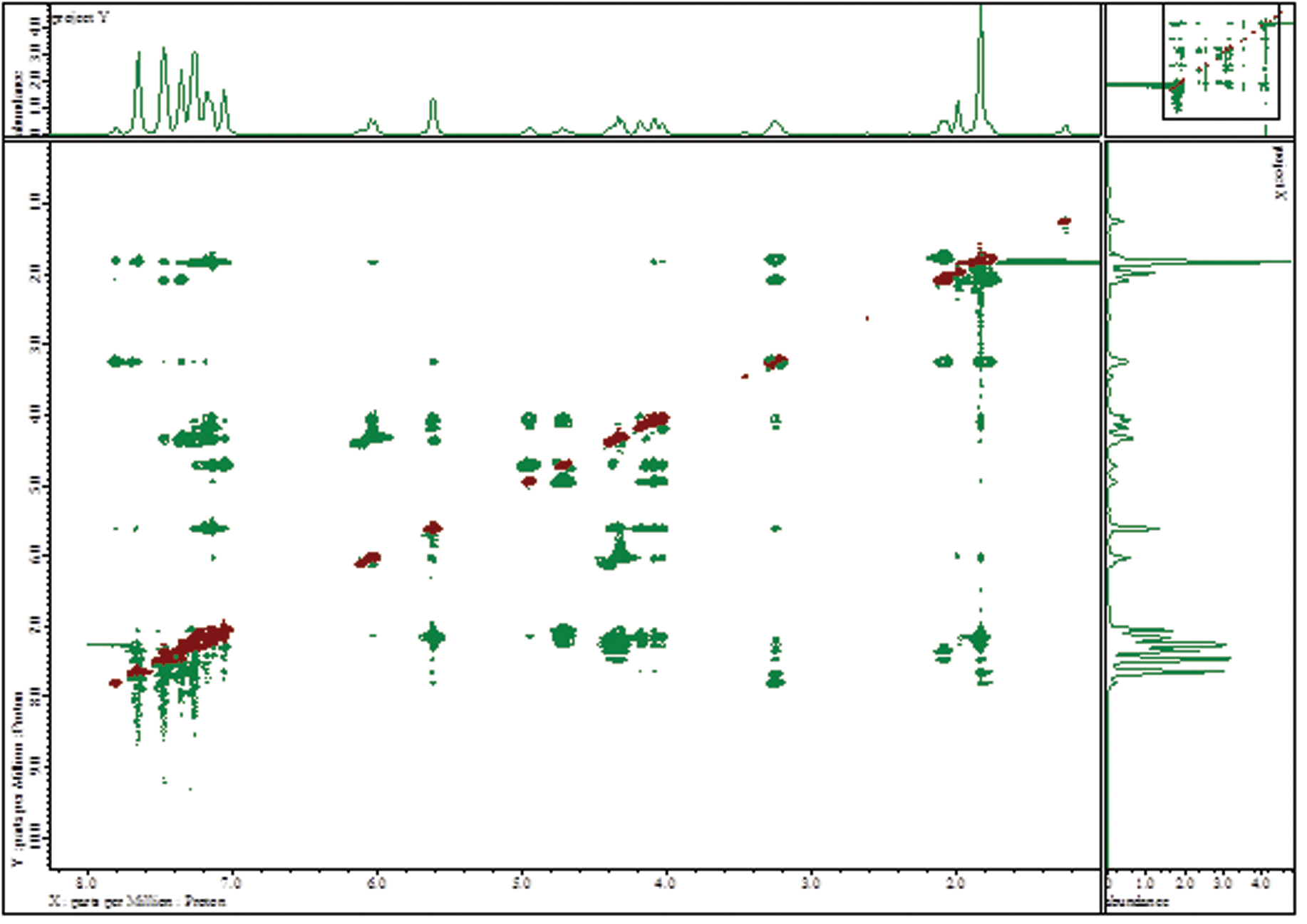

Supplement: S25 Fig — (TIF) [file pone.0144613.s025.tif]

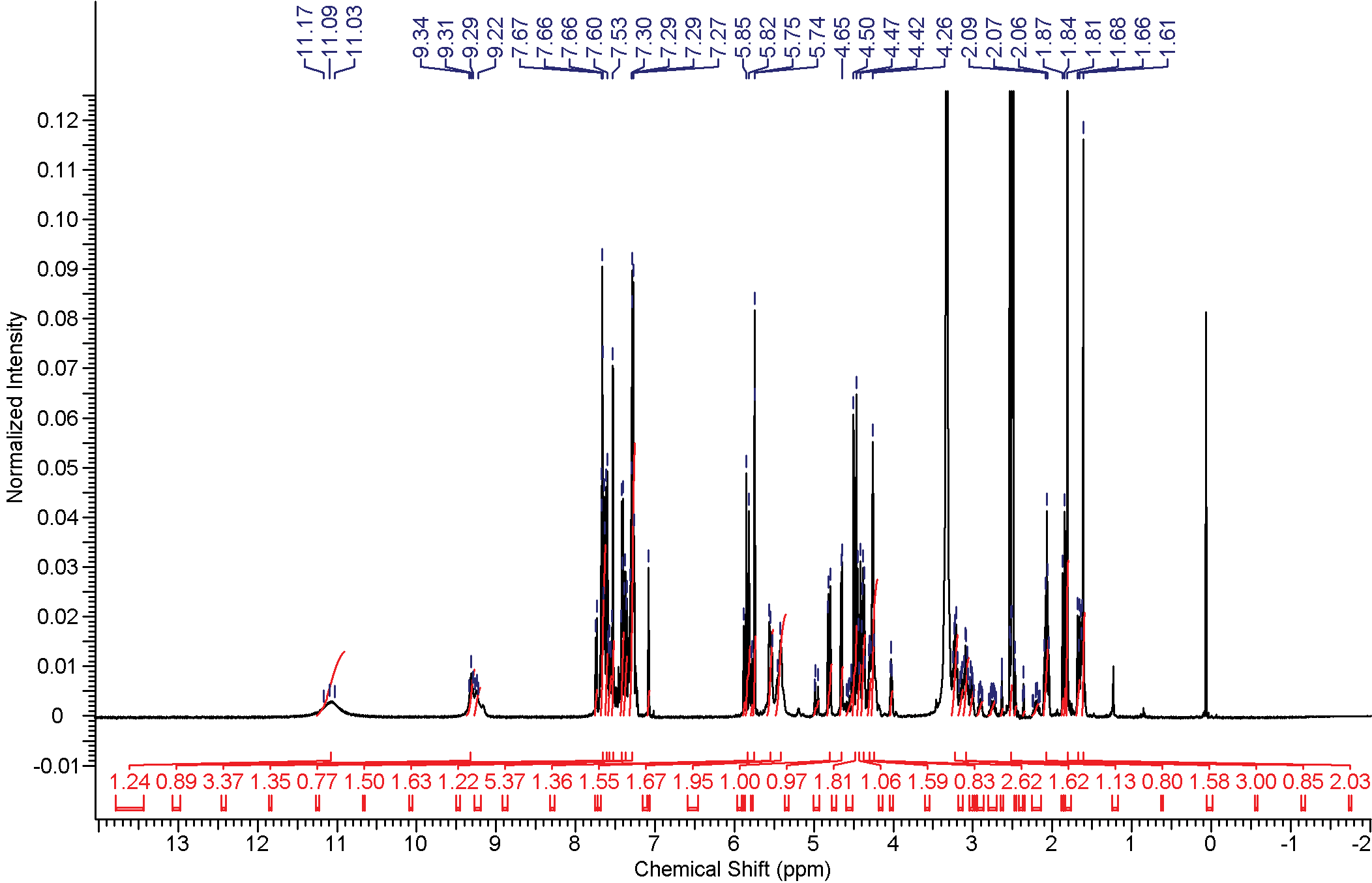

Supplement: S26 Fig — (TIF) [file pone.0144613.s026.tif]

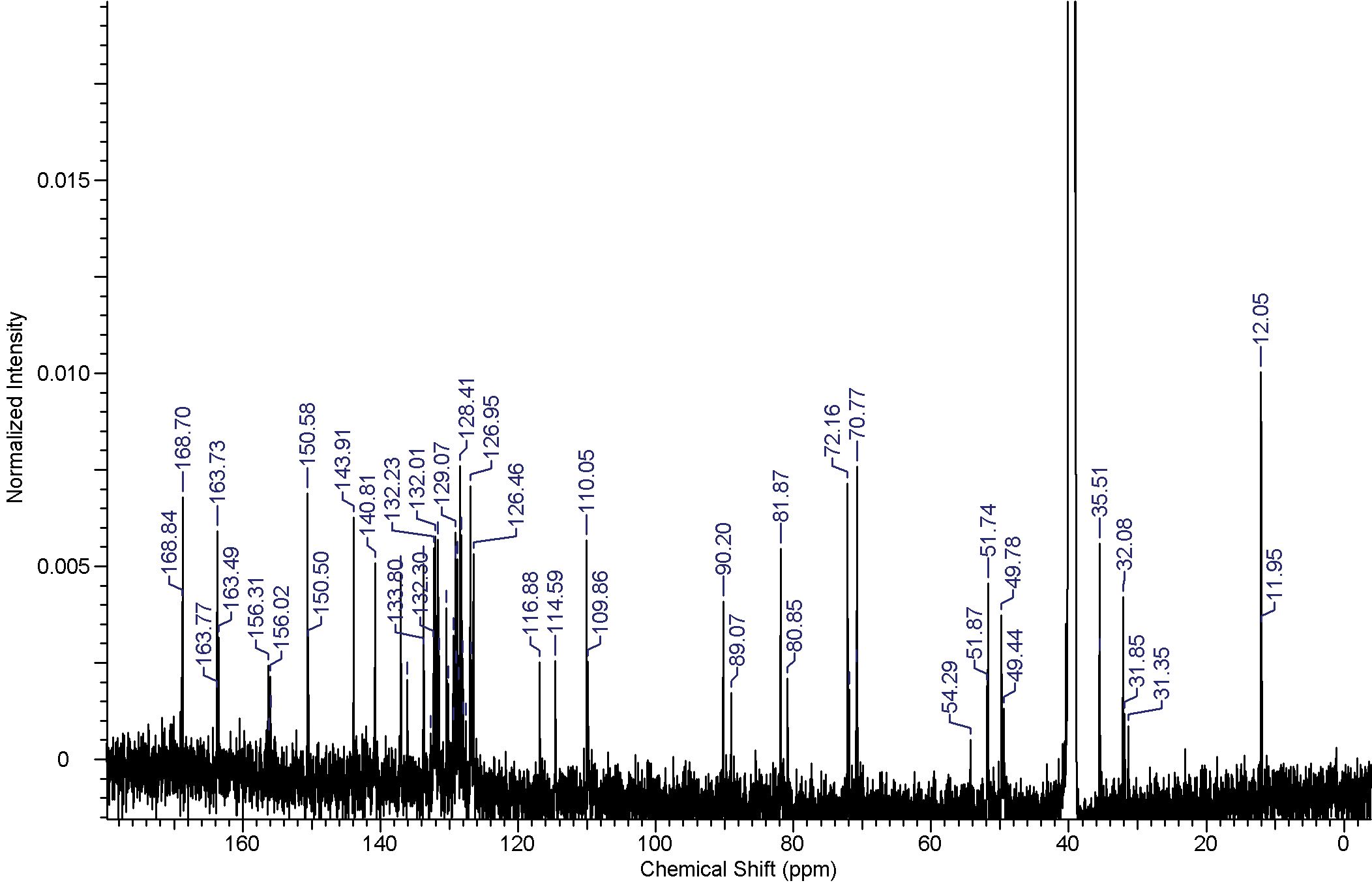

Supplement: S27 Fig — (TIF) [file pone.0144613.s027.tif]

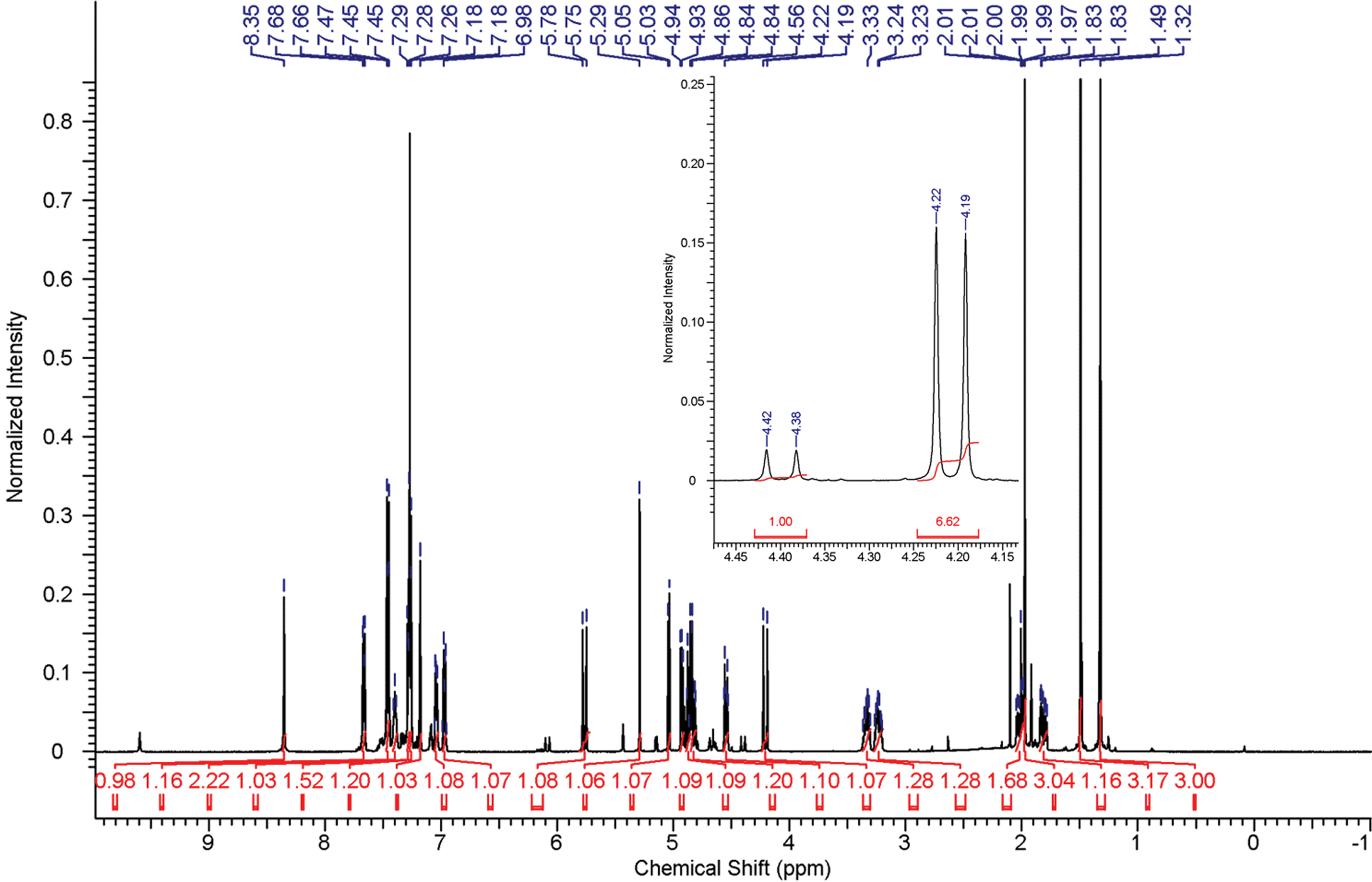

Supplement: S28 Fig — (TIF) [file pone.0144613.s028.tif]

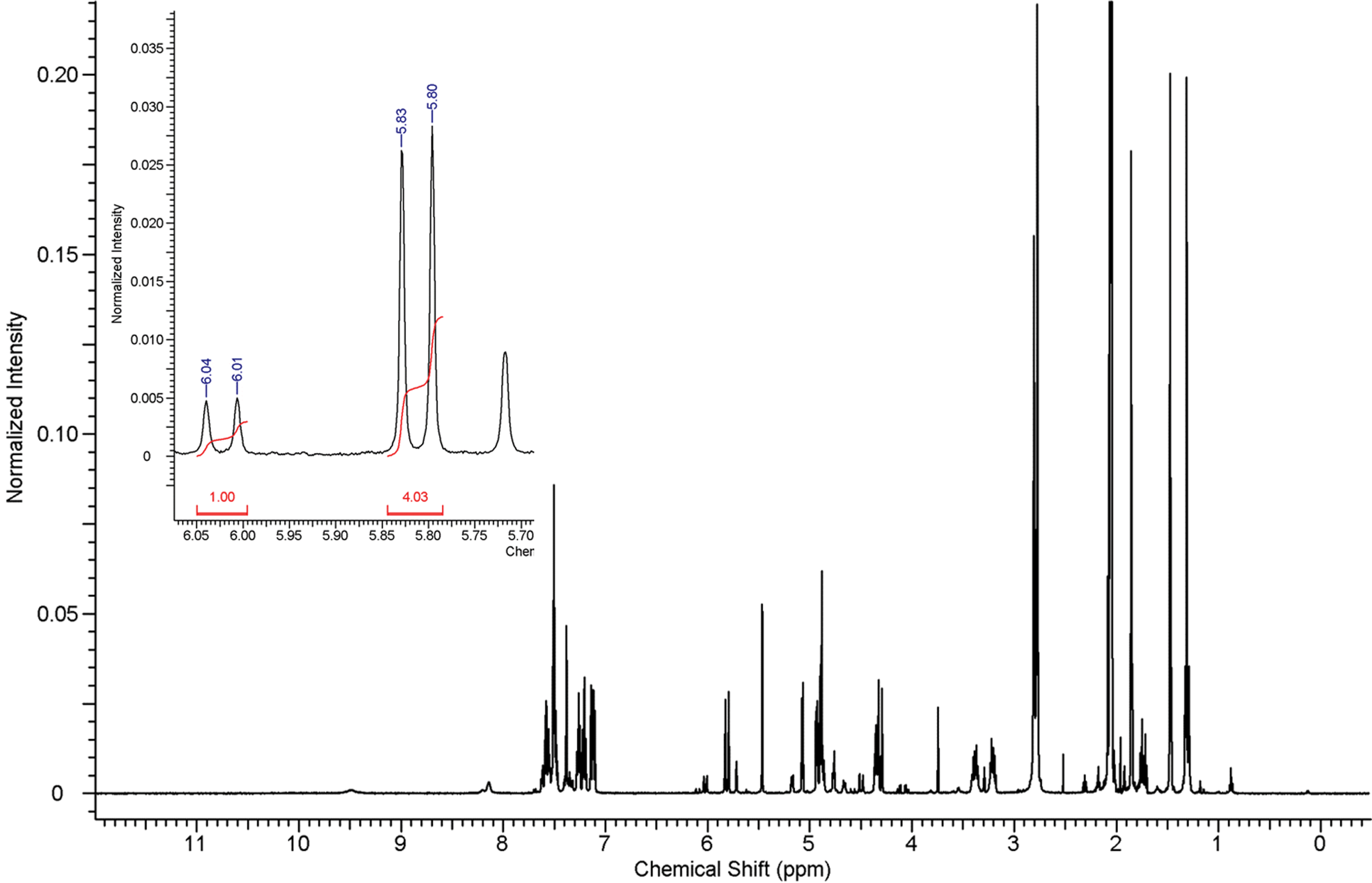

Supplement: S29 Fig — (TIF) [file pone.0144613.s029.tif]

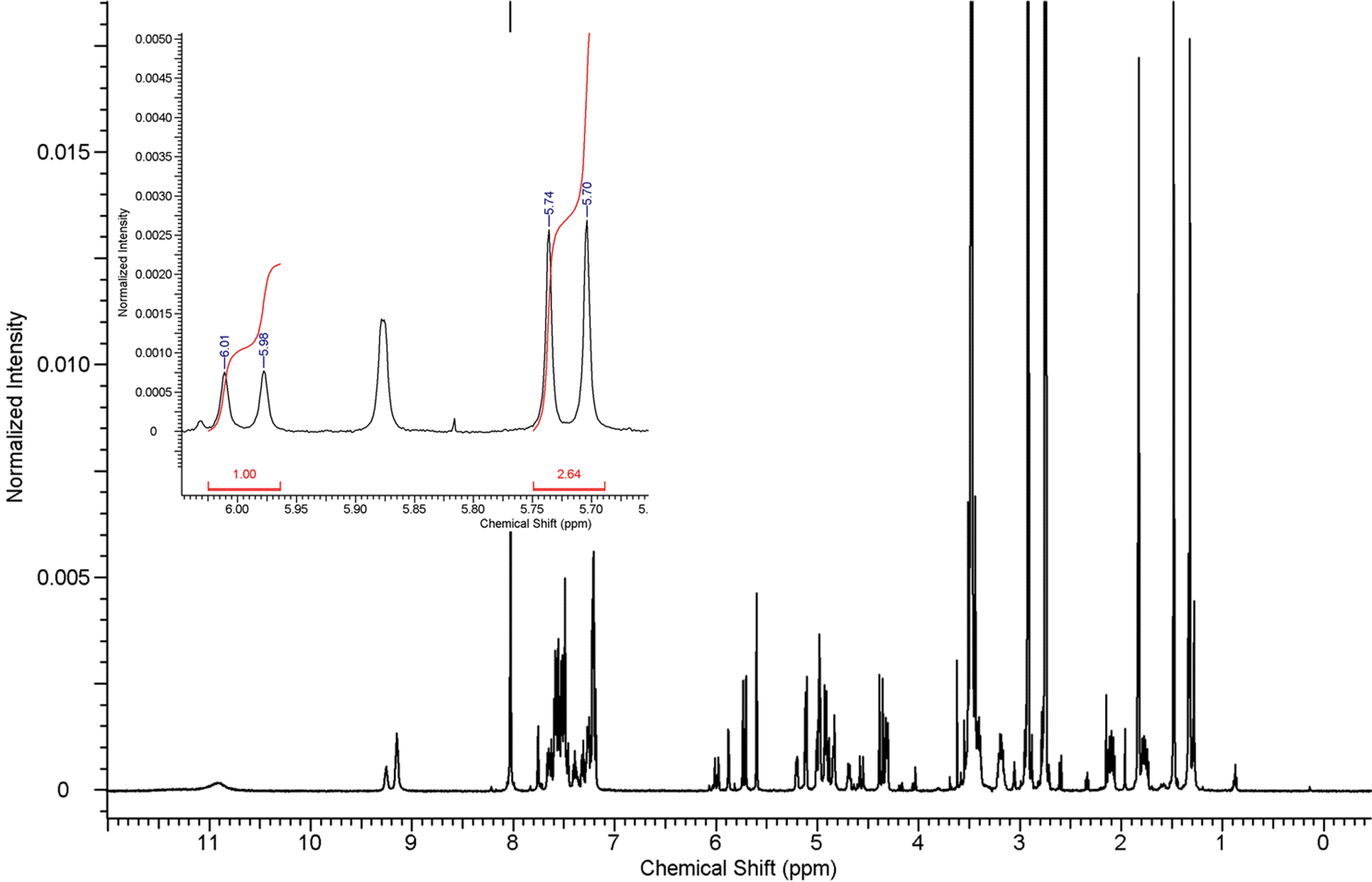

Supplement: S30 Fig — (TIF) [file pone.0144613.s030.tif]

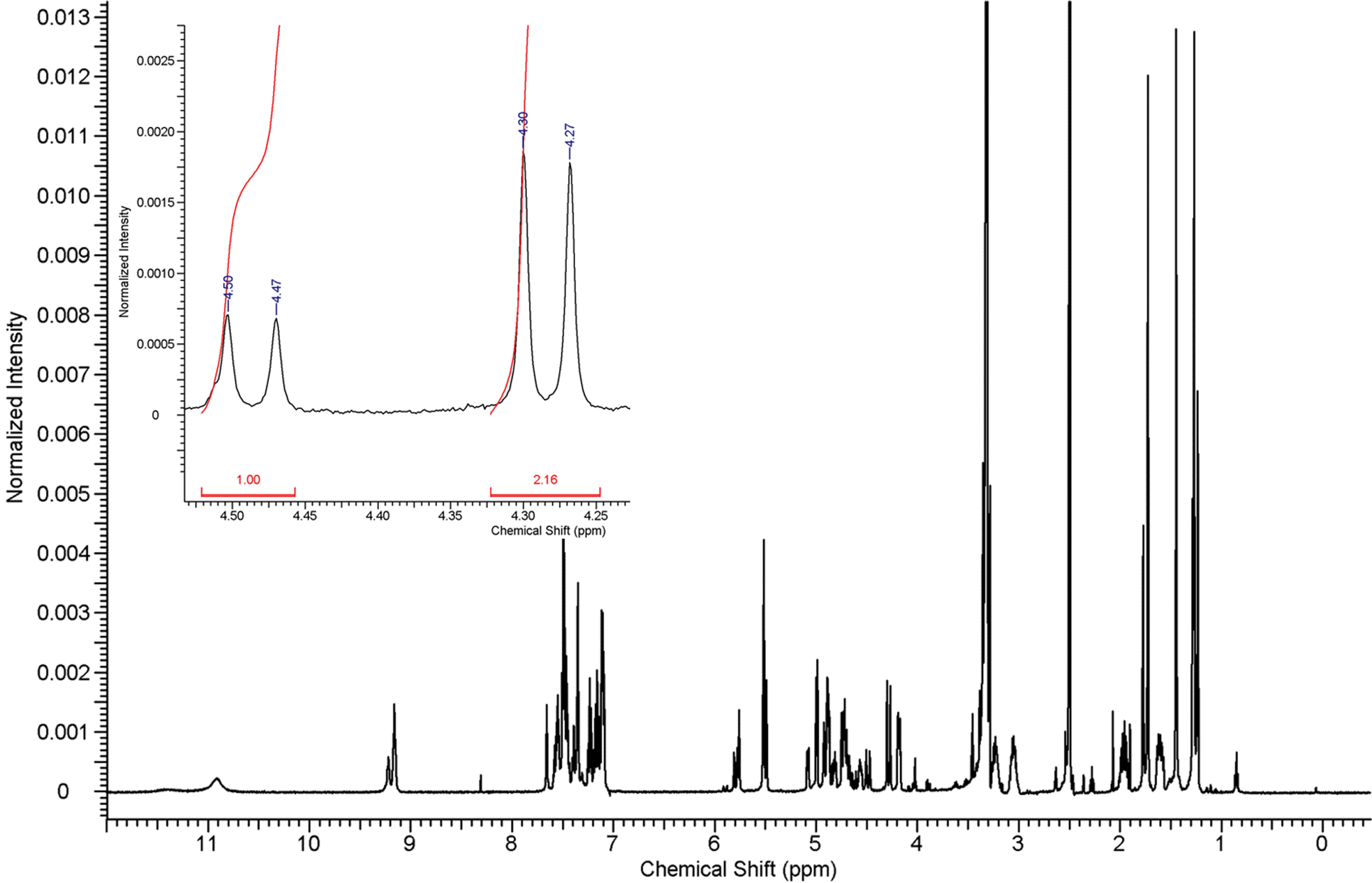

Supplement: S31 Fig — (TIF) [file pone.0144613.s031.tif]

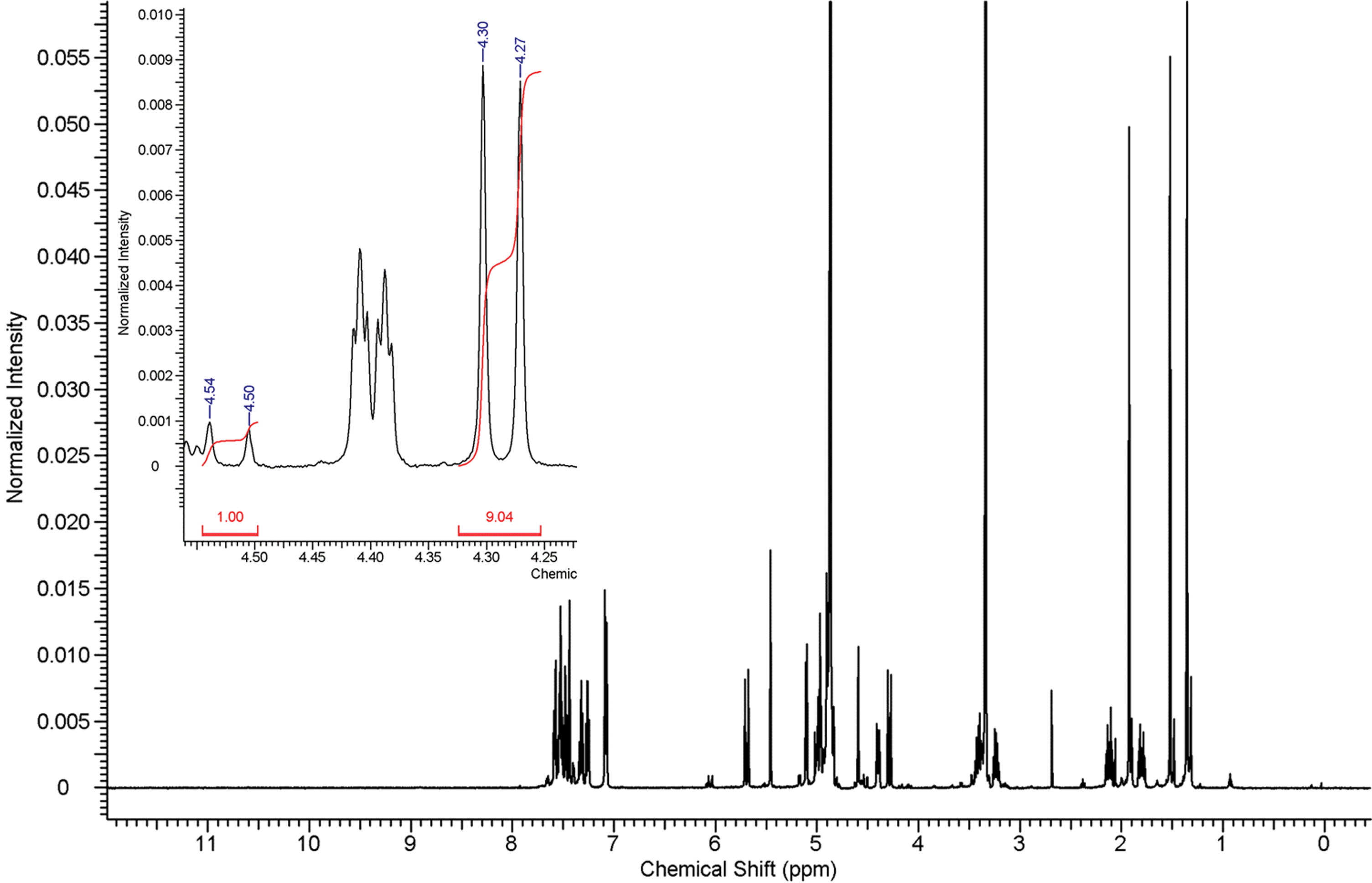

Supplement: S32 Fig — (TIF) [file pone.0144613.s032.tif]

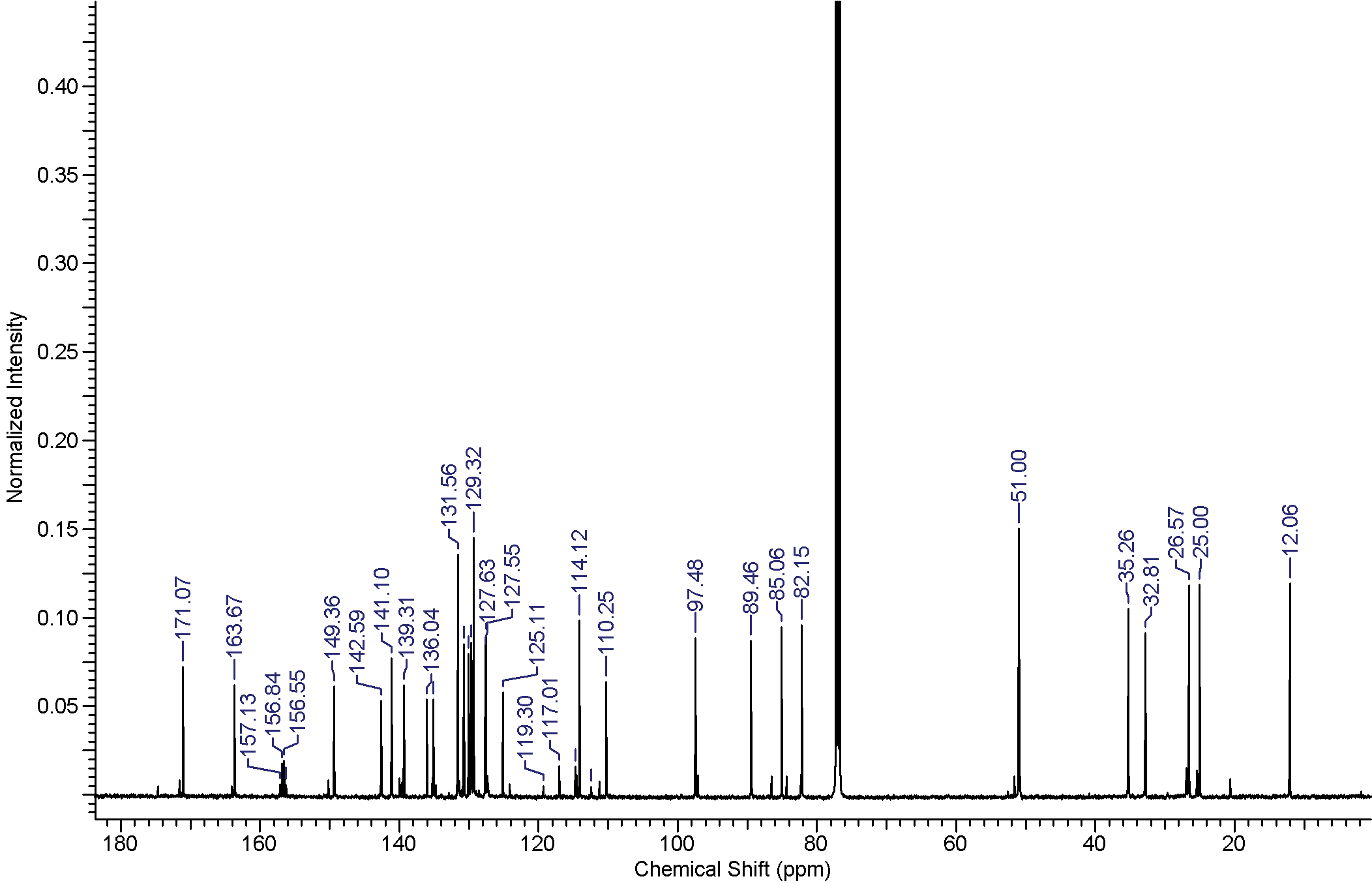

Supplement: S33 Fig — (TIF) [file pone.0144613.s033.tif]

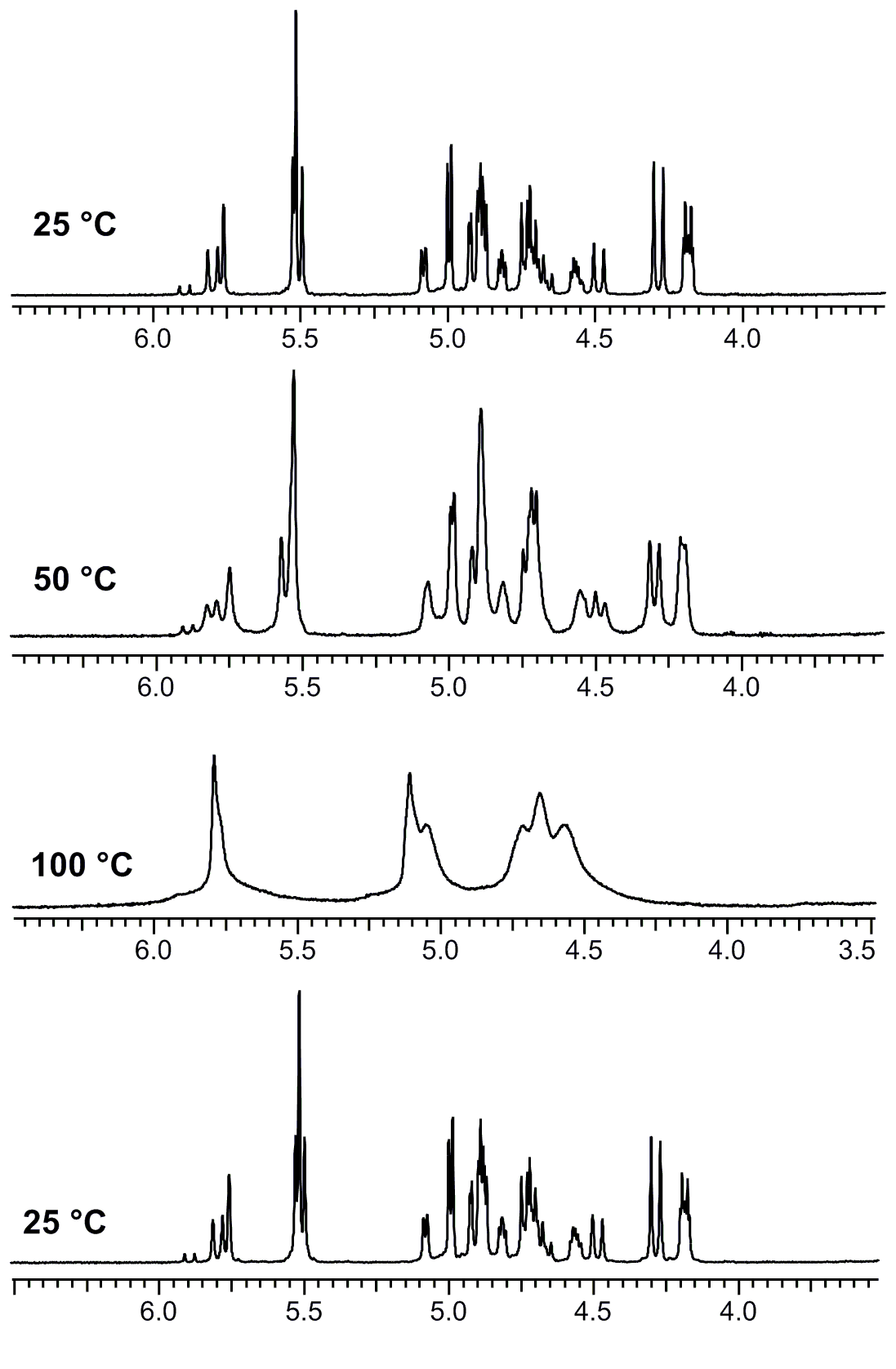

Supplement: S34 Fig — The bottom spectrum was measured after cooling the sample back. (TIF) [file pone.0144613.s034.tif]

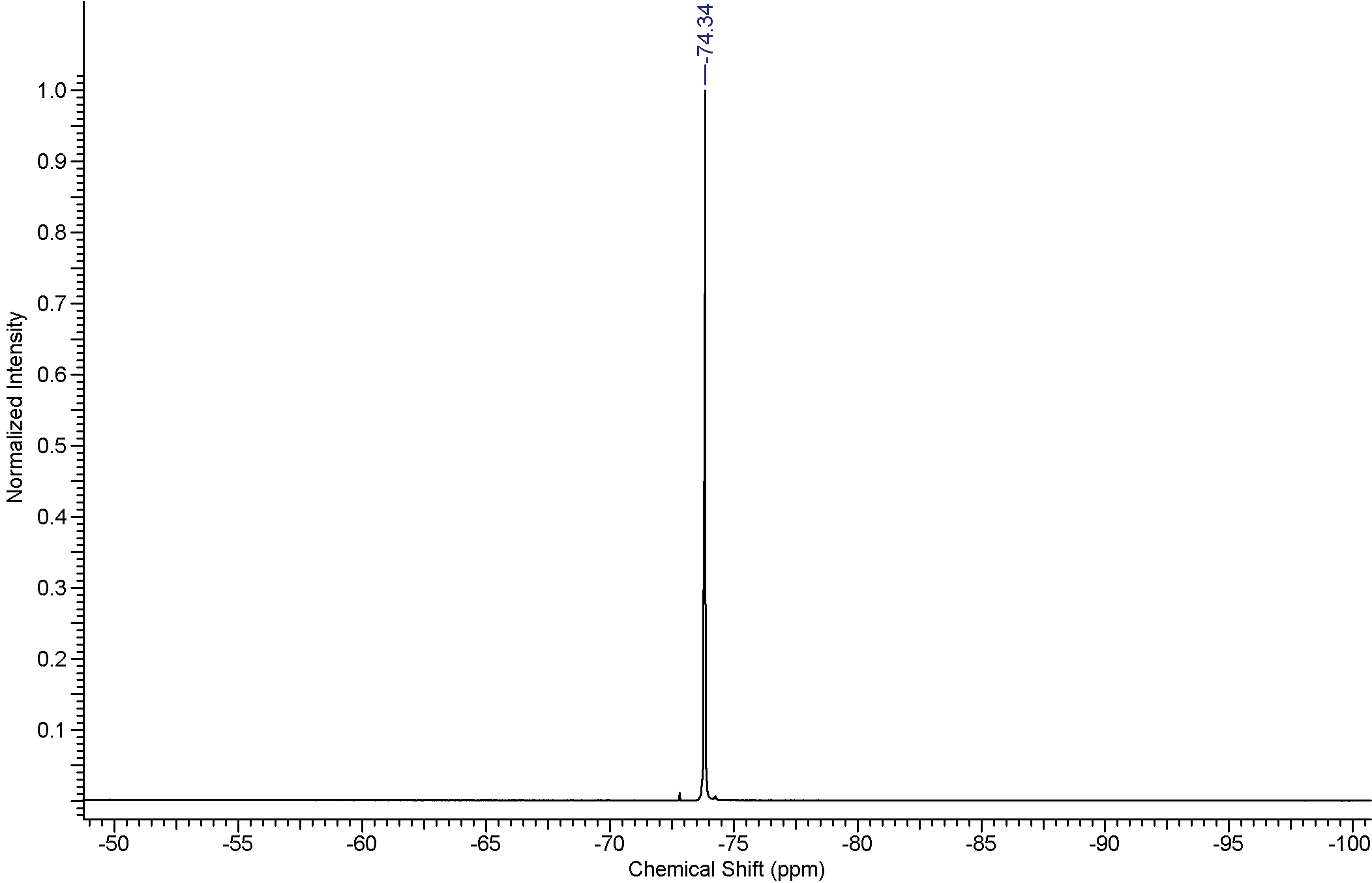

Supplement: S35 Fig — (TIF) [file pone.0144613.s035.tif]

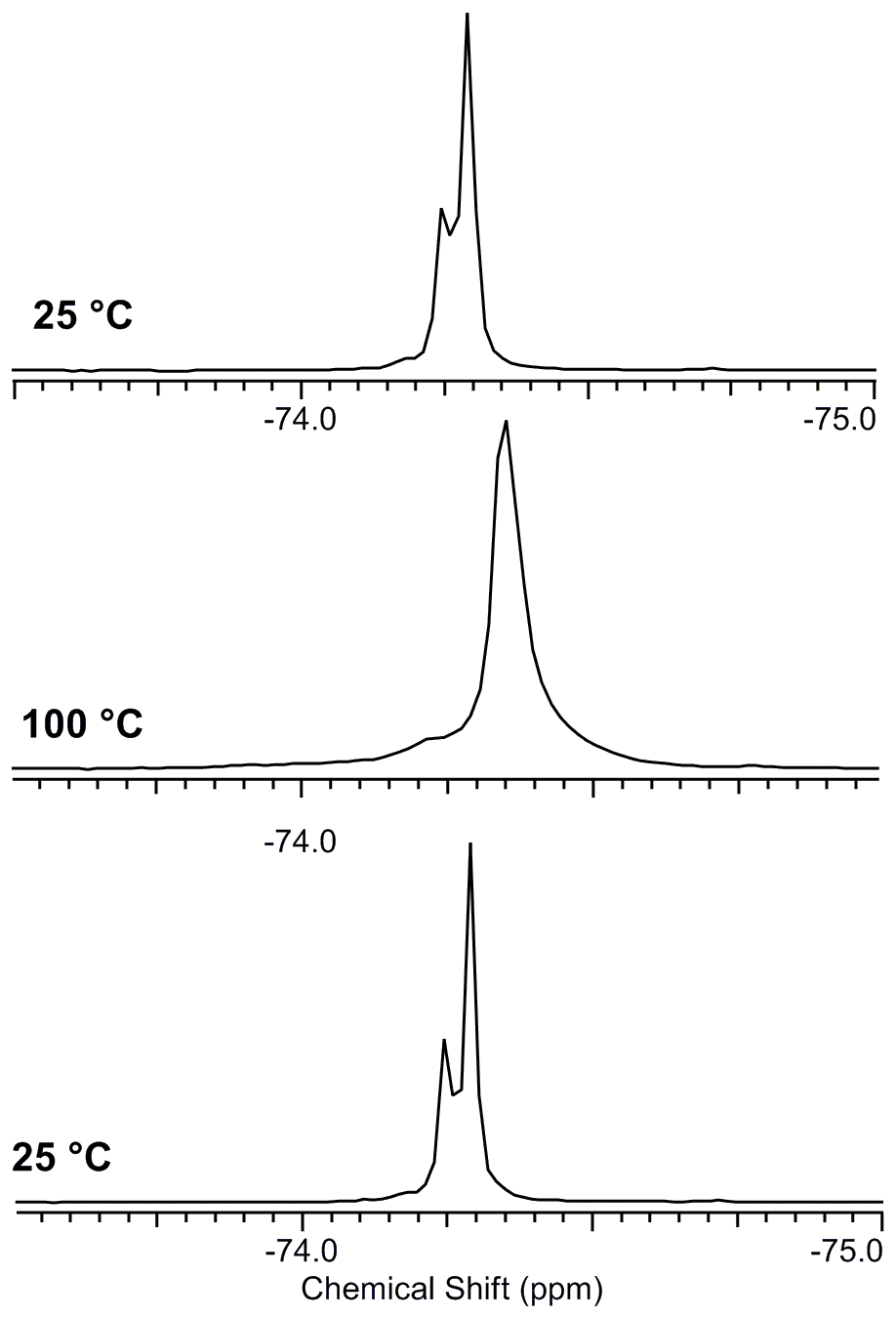

Supplement: S36 Fig — (TIF) [file pone.0144613.s036.tif]

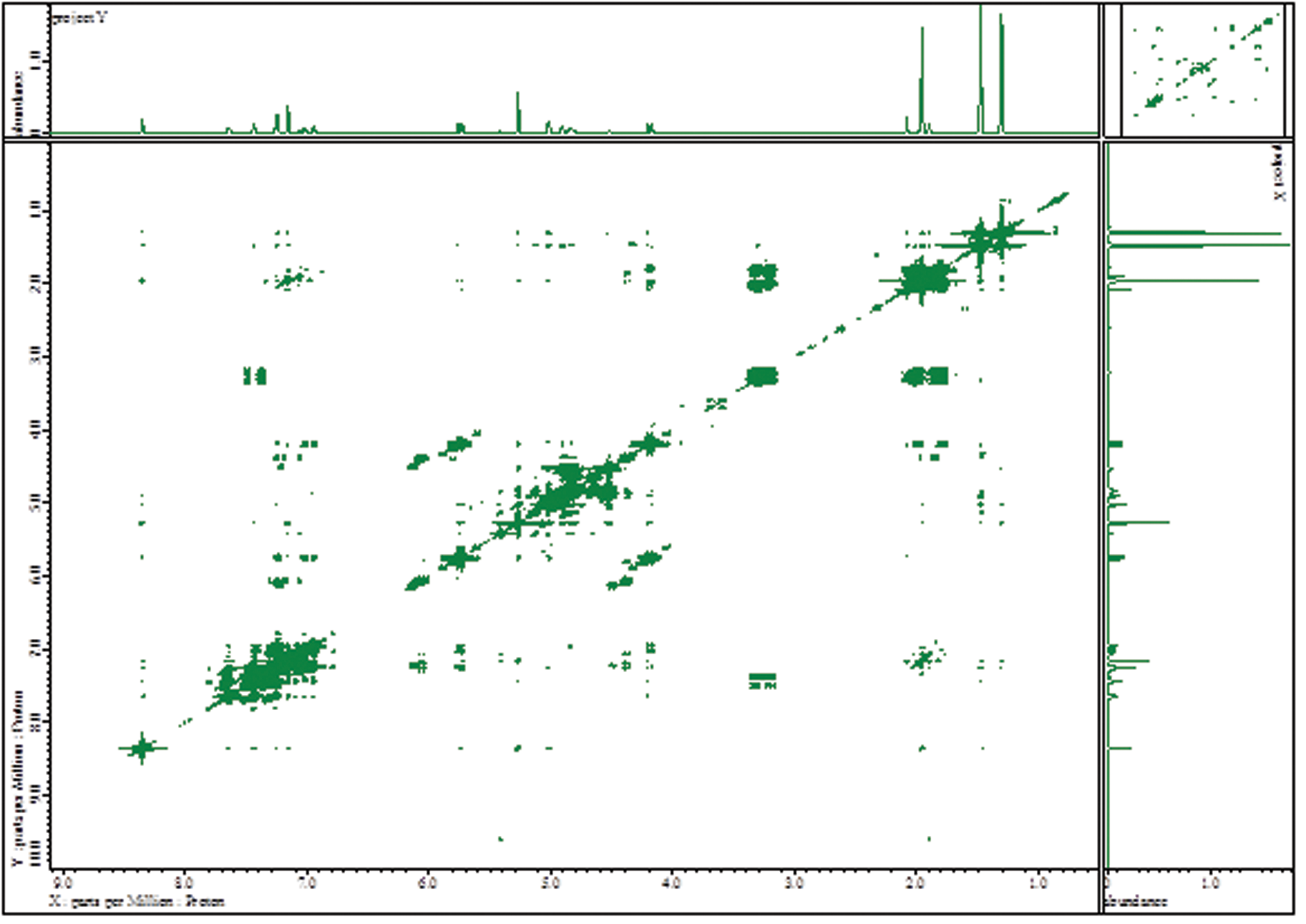

Supplement: S37 Fig — (TIF) [file pone.0144613.s037.tif]

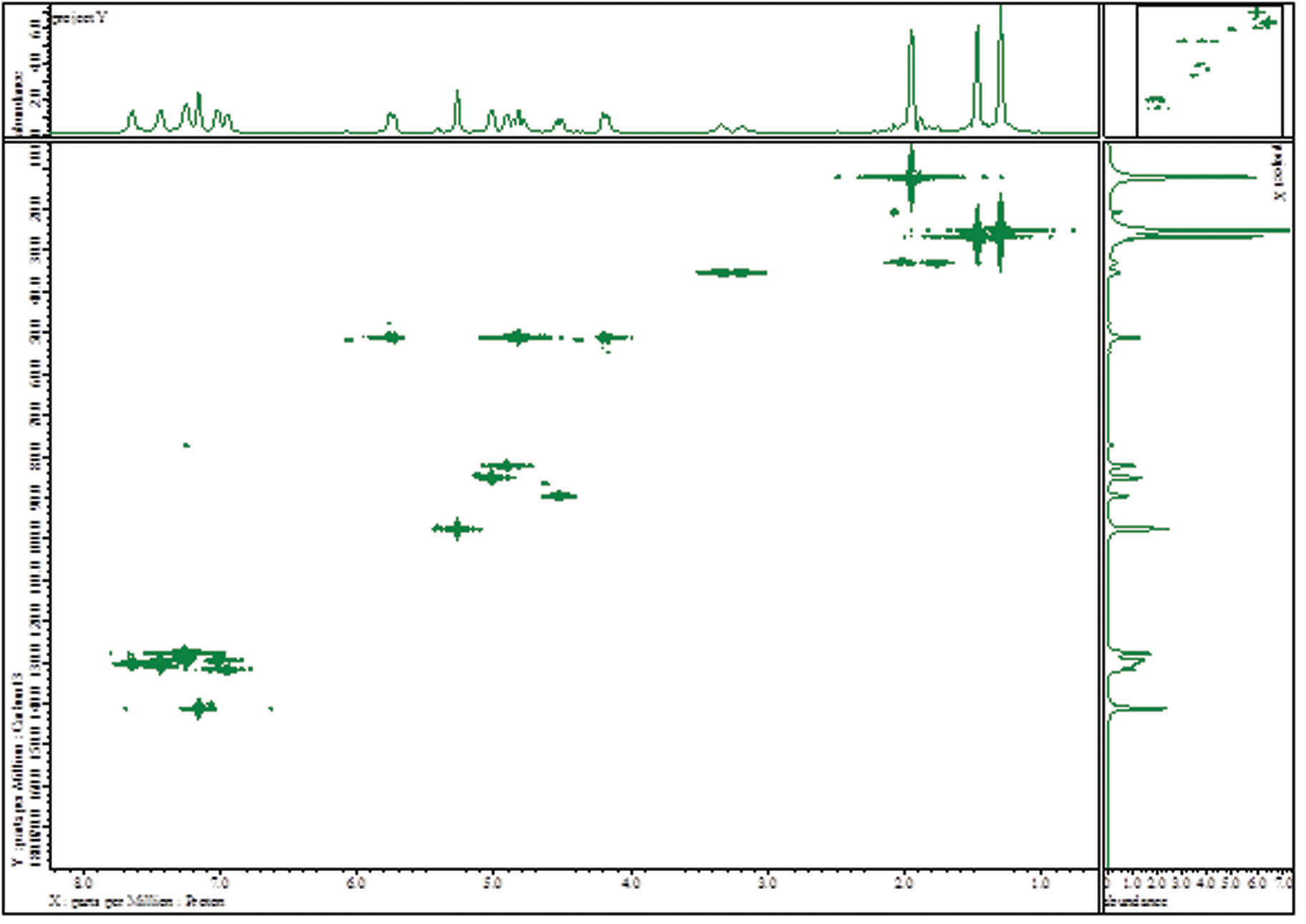

Supplement: S38 Fig — (TIF) [file pone.0144613.s038.tif]

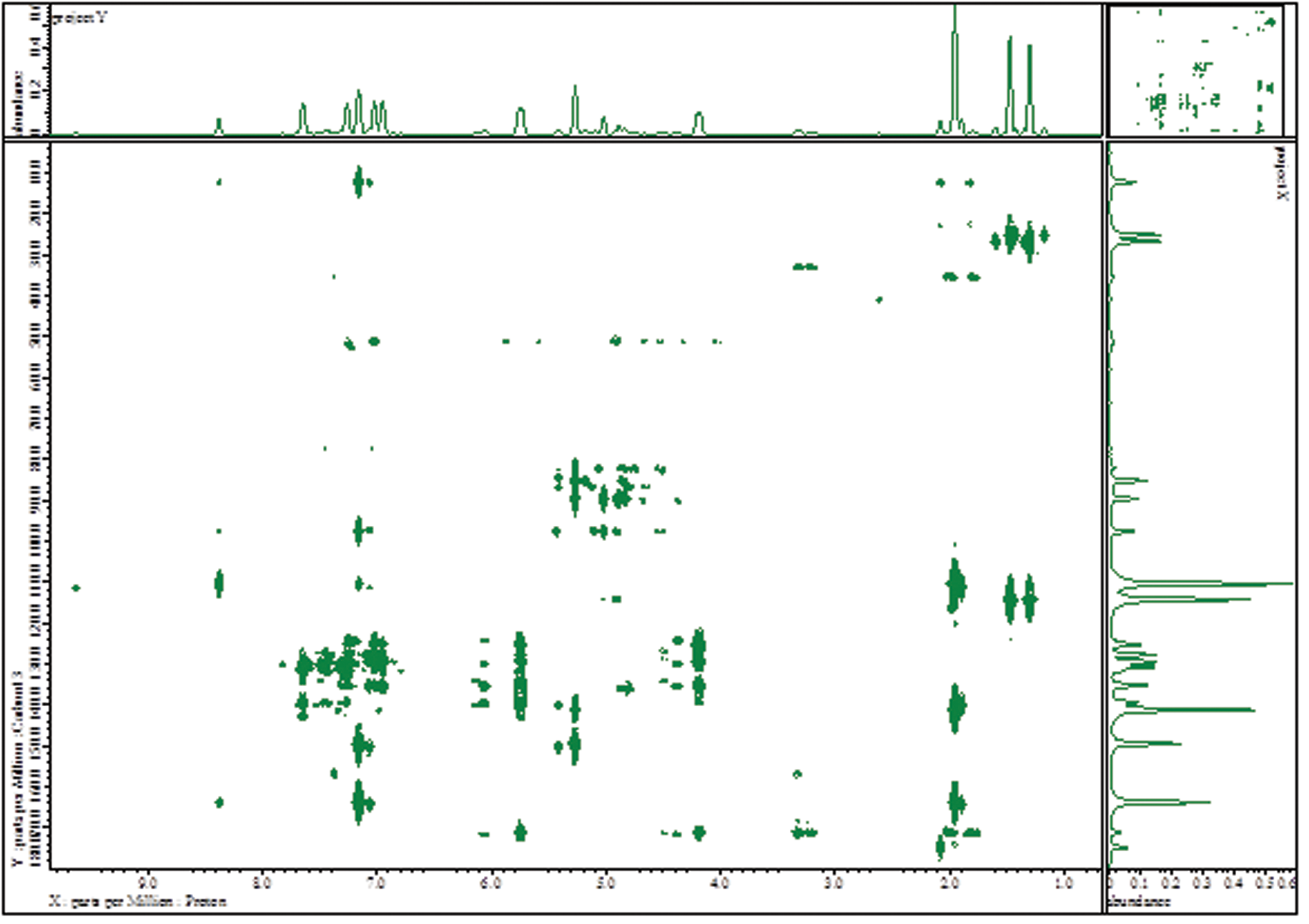

Supplement: S39 Fig — (TIF) [file pone.0144613.s039.tif]

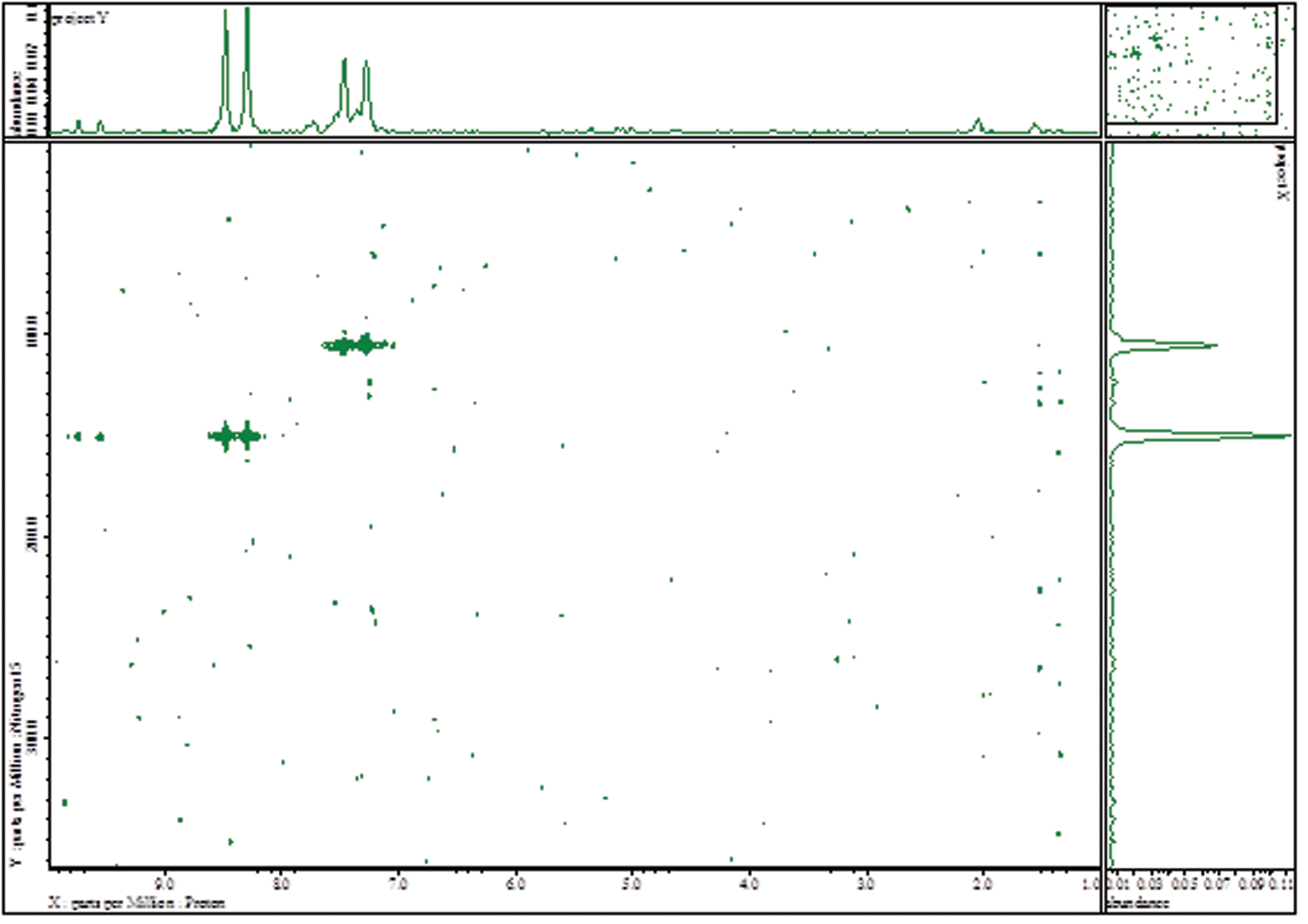

Supplement: S40 Fig — (TIF) [file pone.0144613.s040.tif]

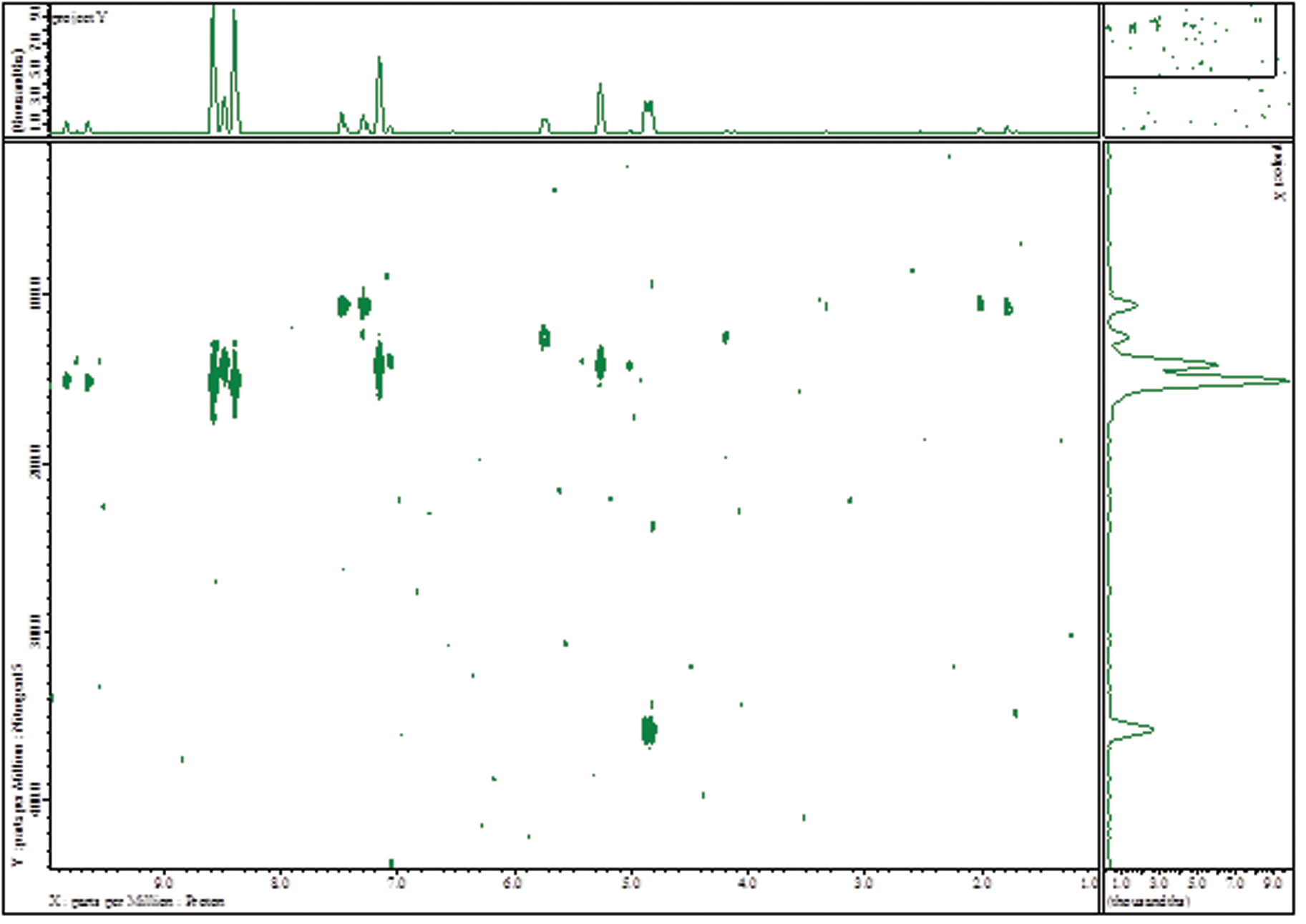

Supplement: S41 Fig — (TIF) [file pone.0144613.s041.tif]

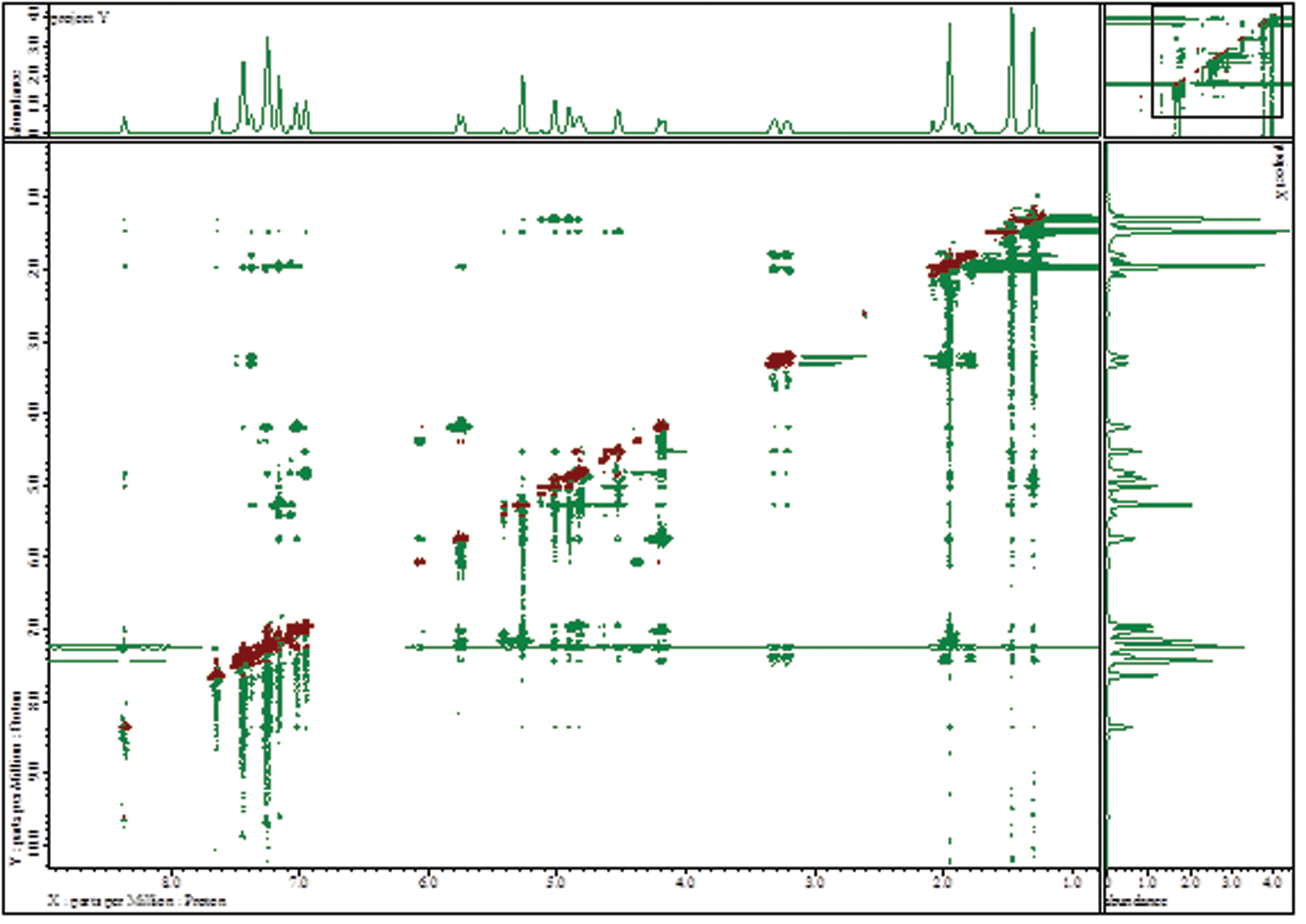

Supplement: S42 Fig — (TIF) [file pone.0144613.s042.tif]

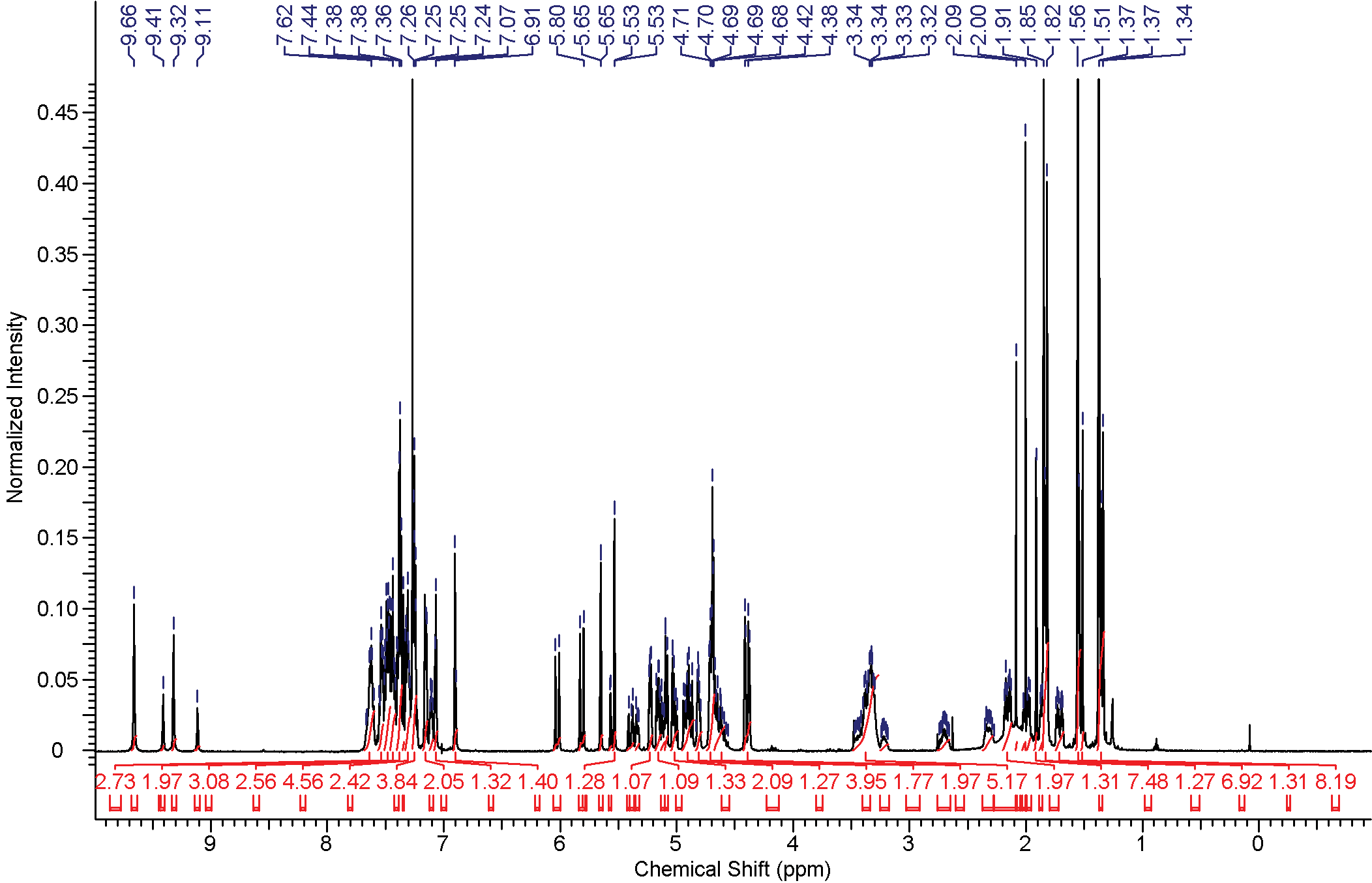

Supplement: S43 Fig — Formation of four conformers is evident especially from NH signals at 9.11–9.66. (TIF) [file pone.0144613.s043.tif]

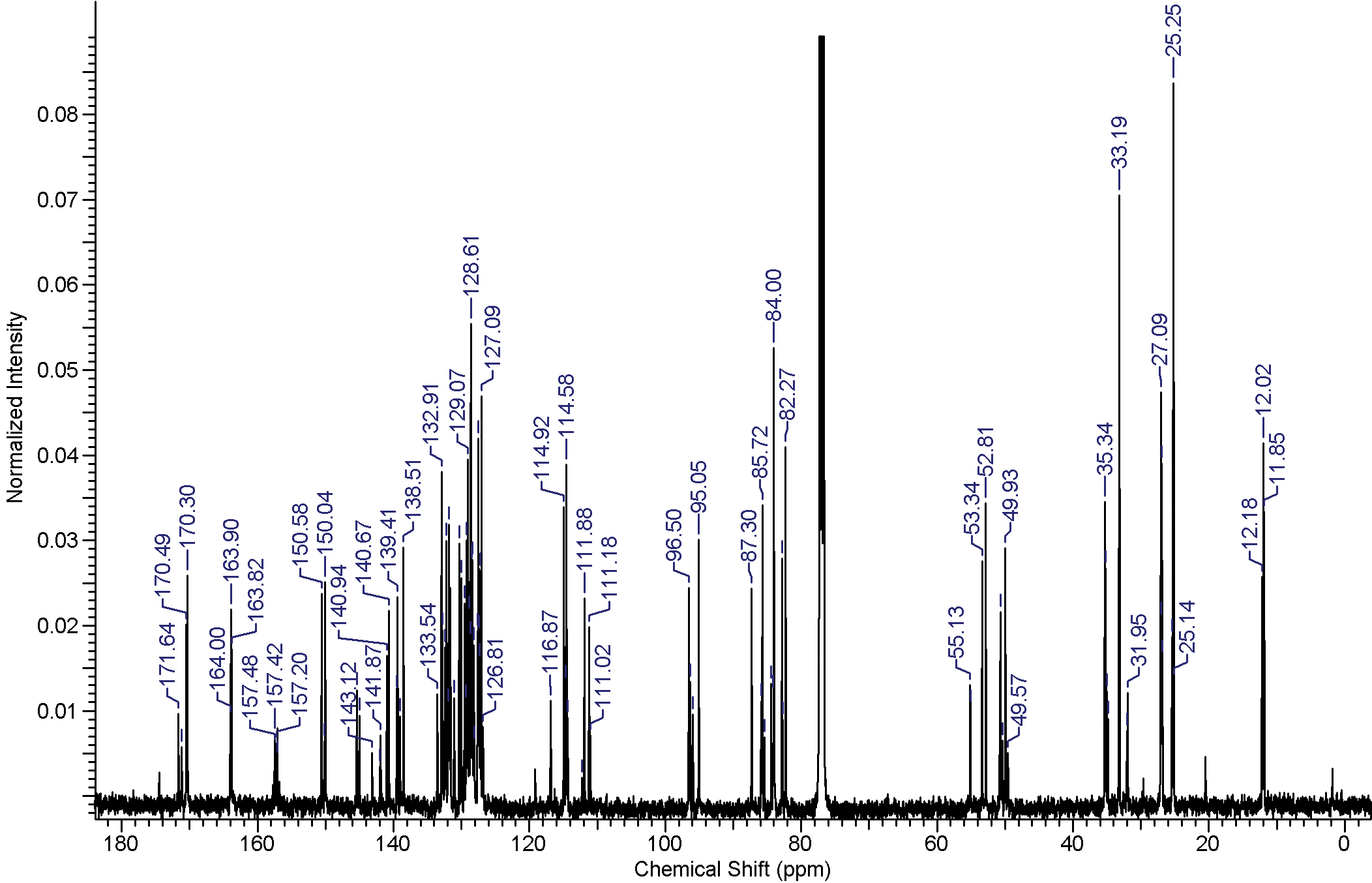

Supplement: S44 Fig — (TIF) [file pone.0144613.s044.tif]

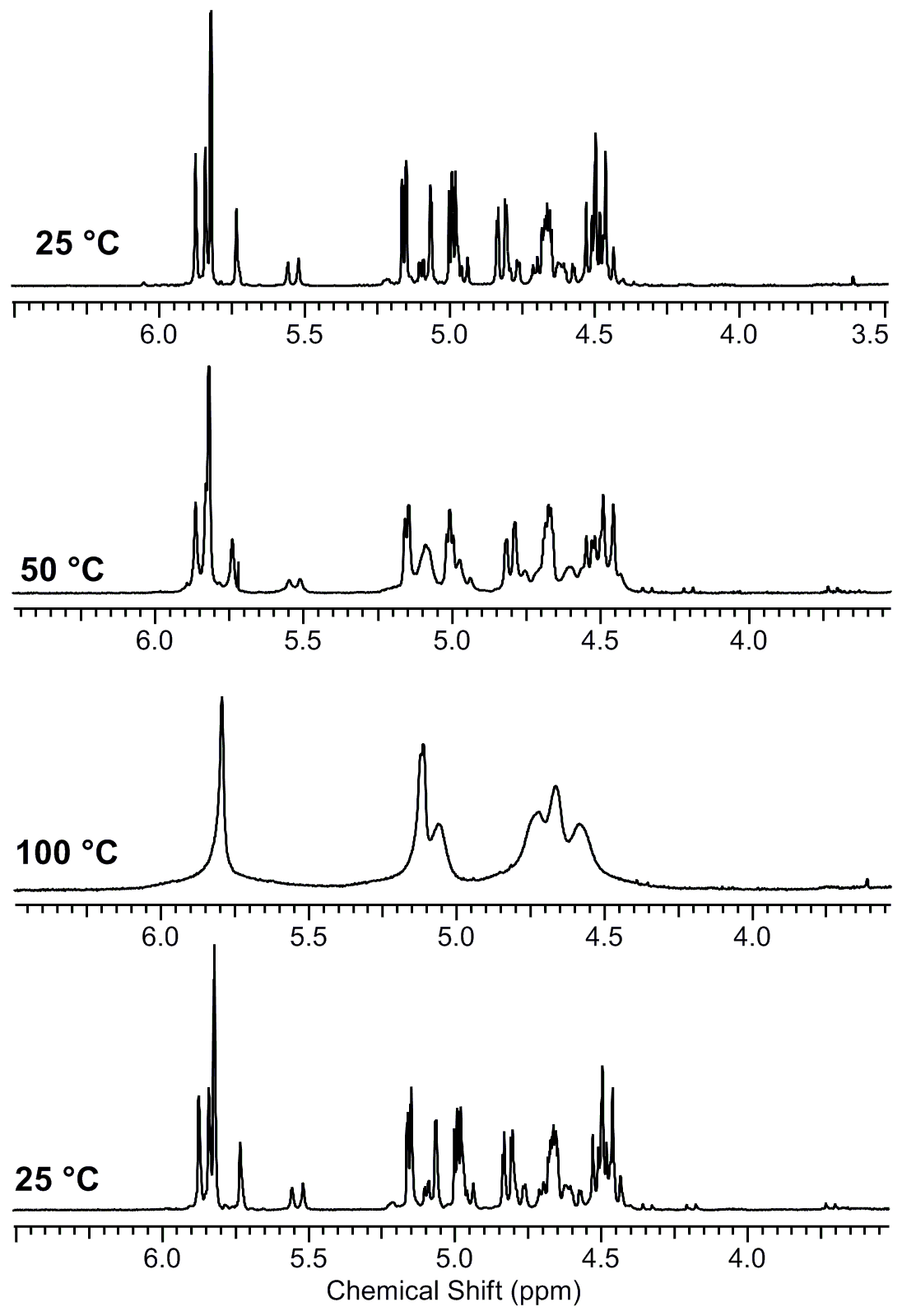

Supplement: S45 Fig — (TIF) [file pone.0144613.s045.tif]

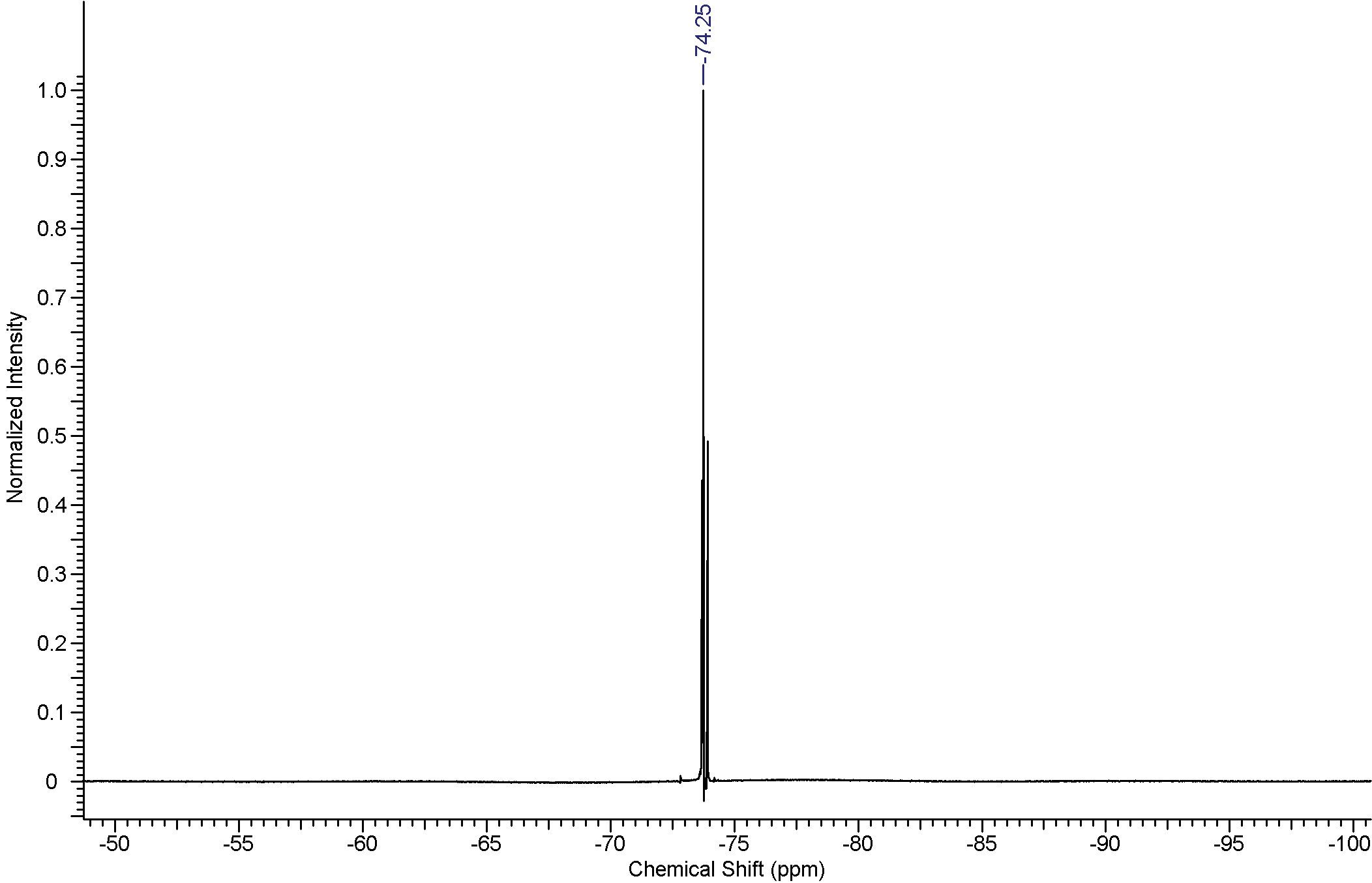

Supplement: S46 Fig — (TIF) [file pone.0144613.s046.tif]

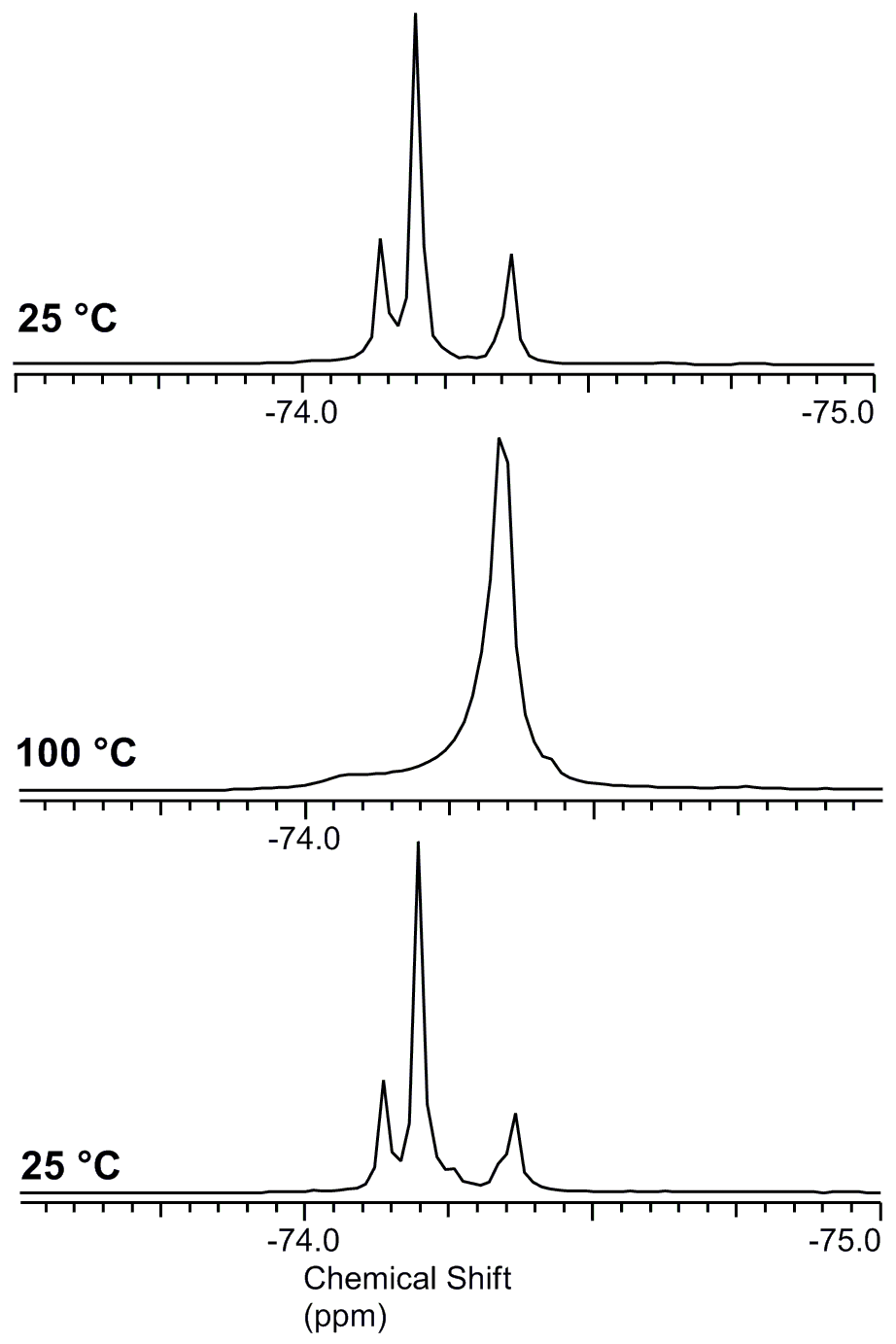

Supplement: S47 Fig — (TIF) [file pone.0144613.s047.tif]

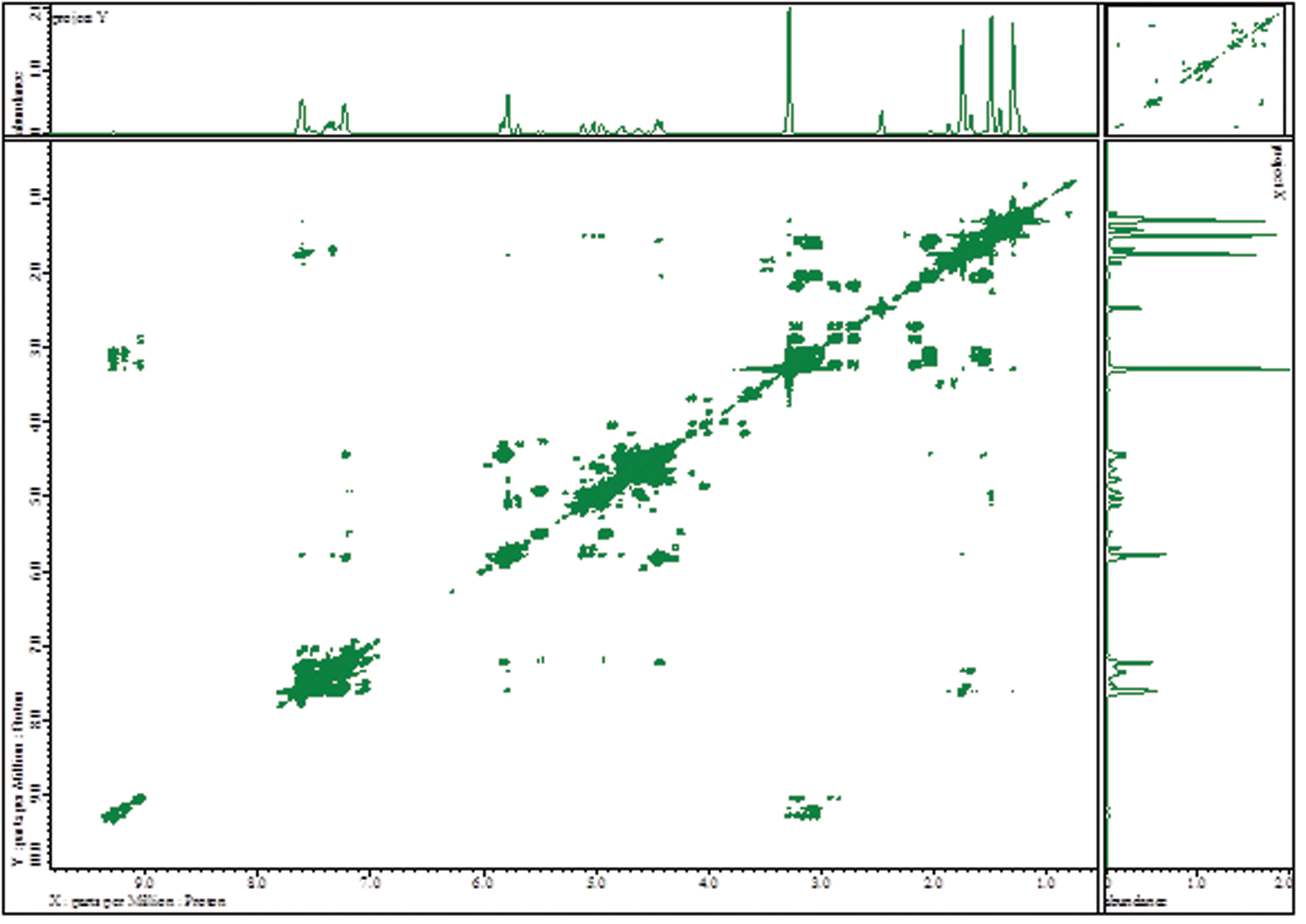

Supplement: S48 Fig — (TIF) [file pone.0144613.s048.tif]

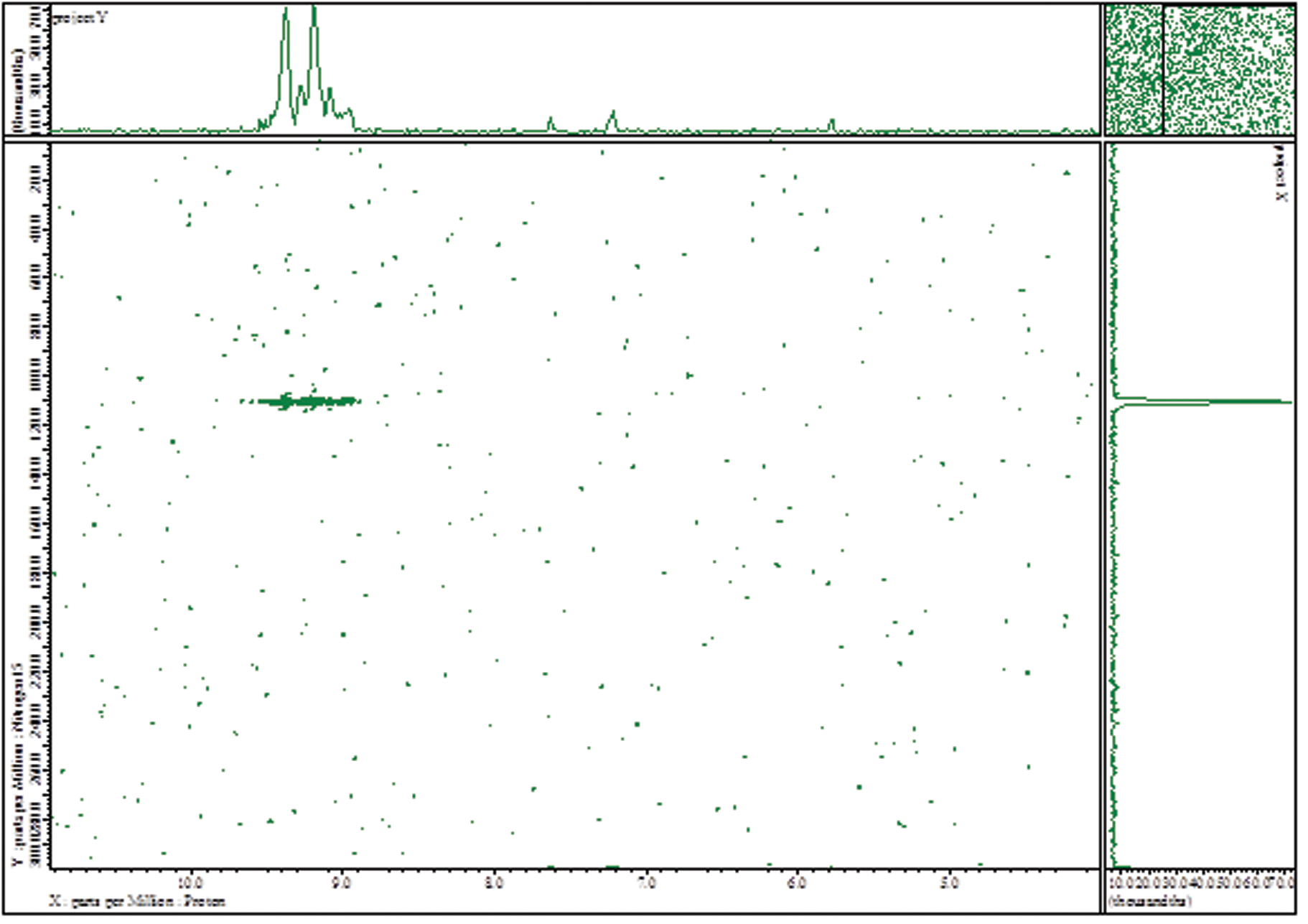

Supplement: S49 Fig — (TIF) [file pone.0144613.s049.tif]

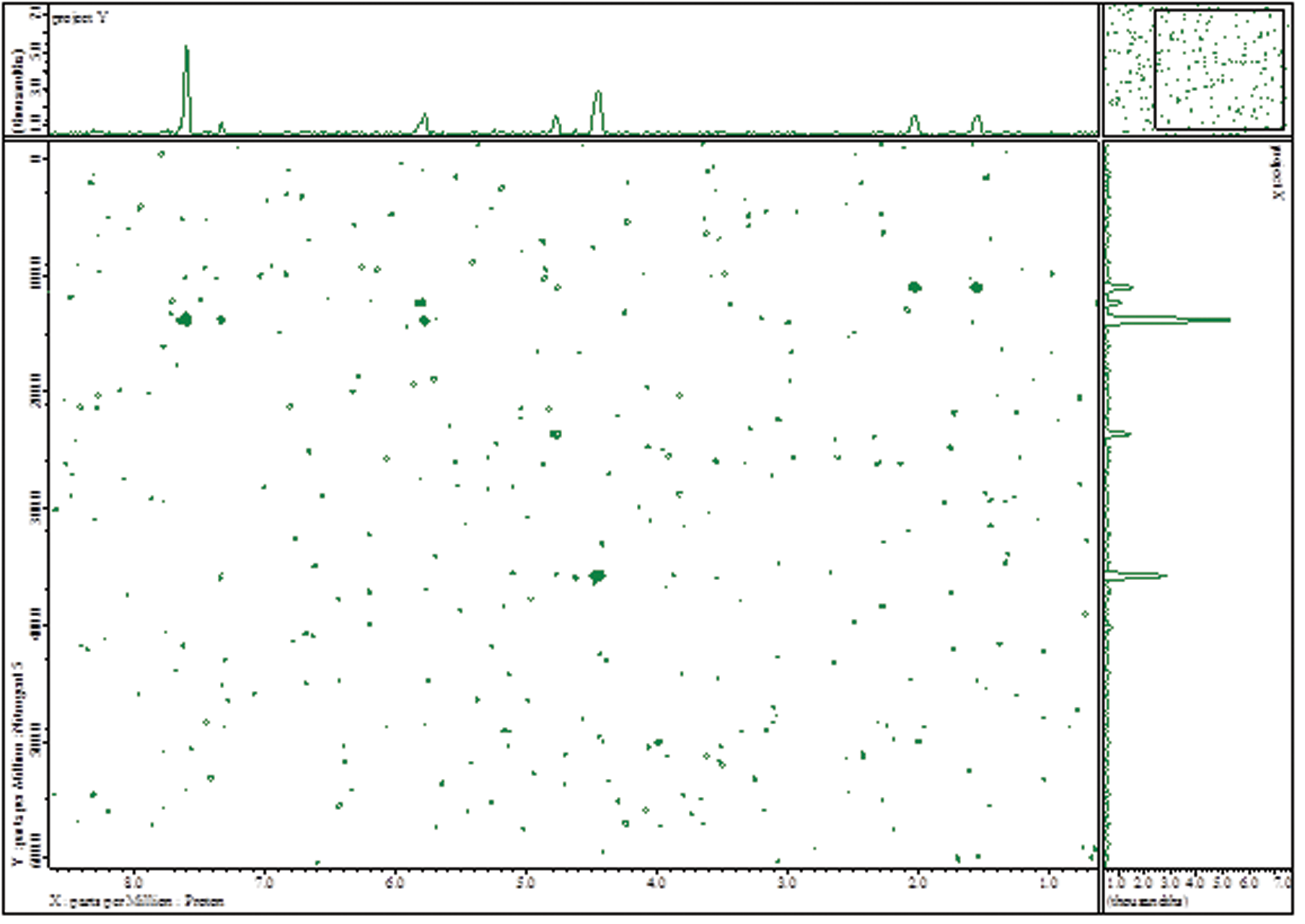

Supplement: S50 Fig — (TIF) [file pone.0144613.s050.tif]

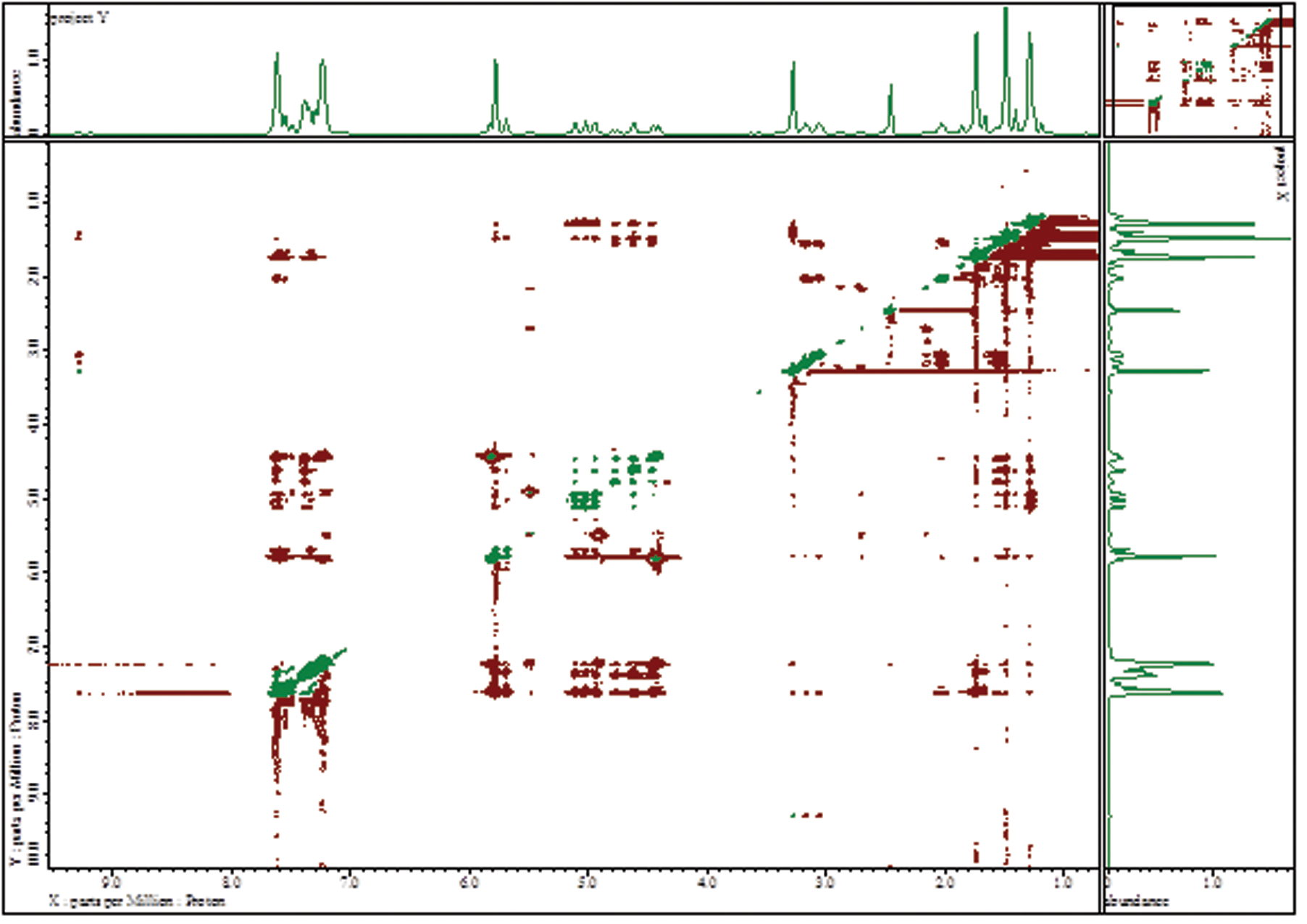

Supplement: S51 Fig — (TIF) [file pone.0144613.s051.tif]

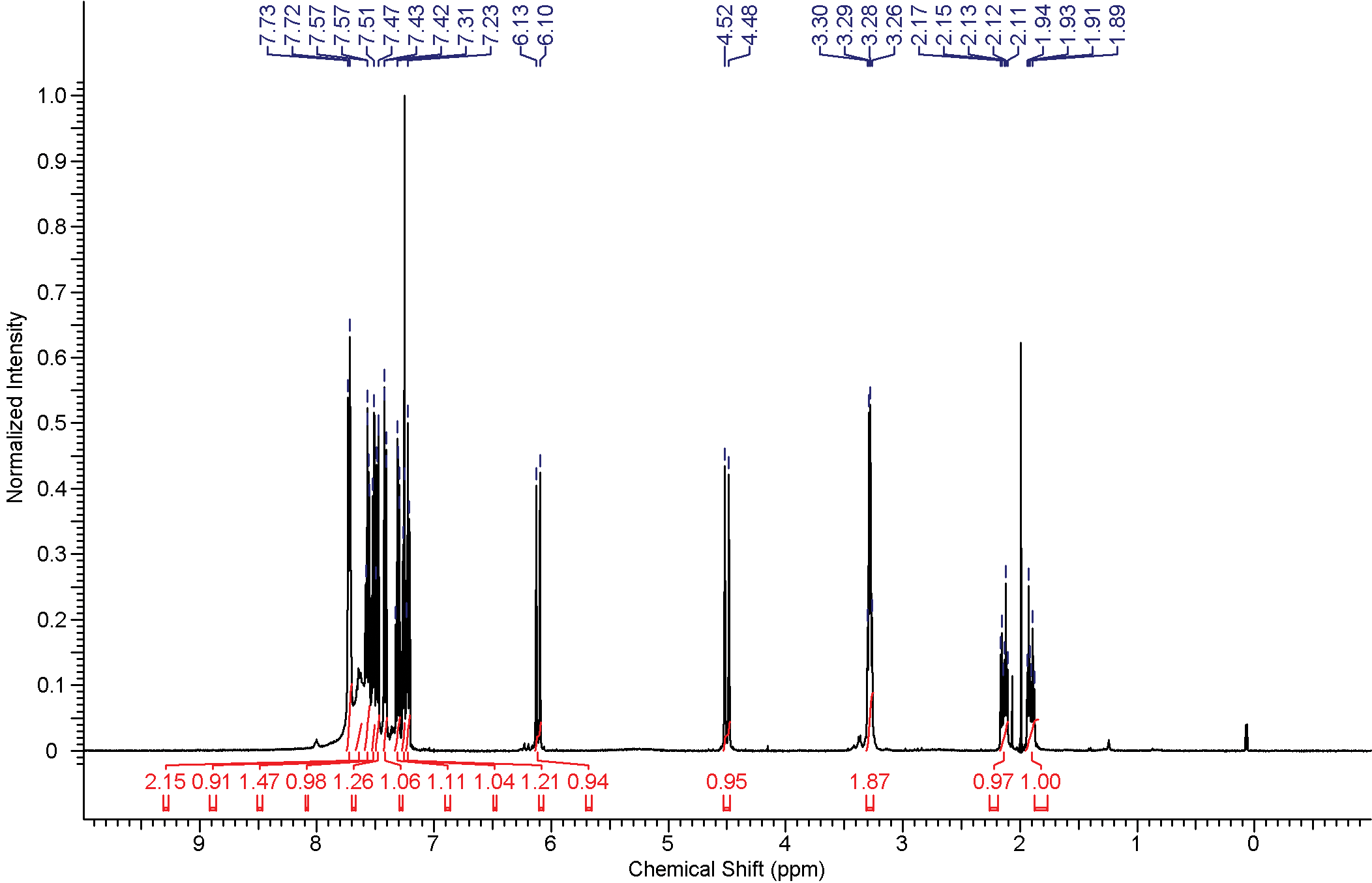

Supplement: S52 Fig — (TIF) [file pone.0144613.s052.tif]

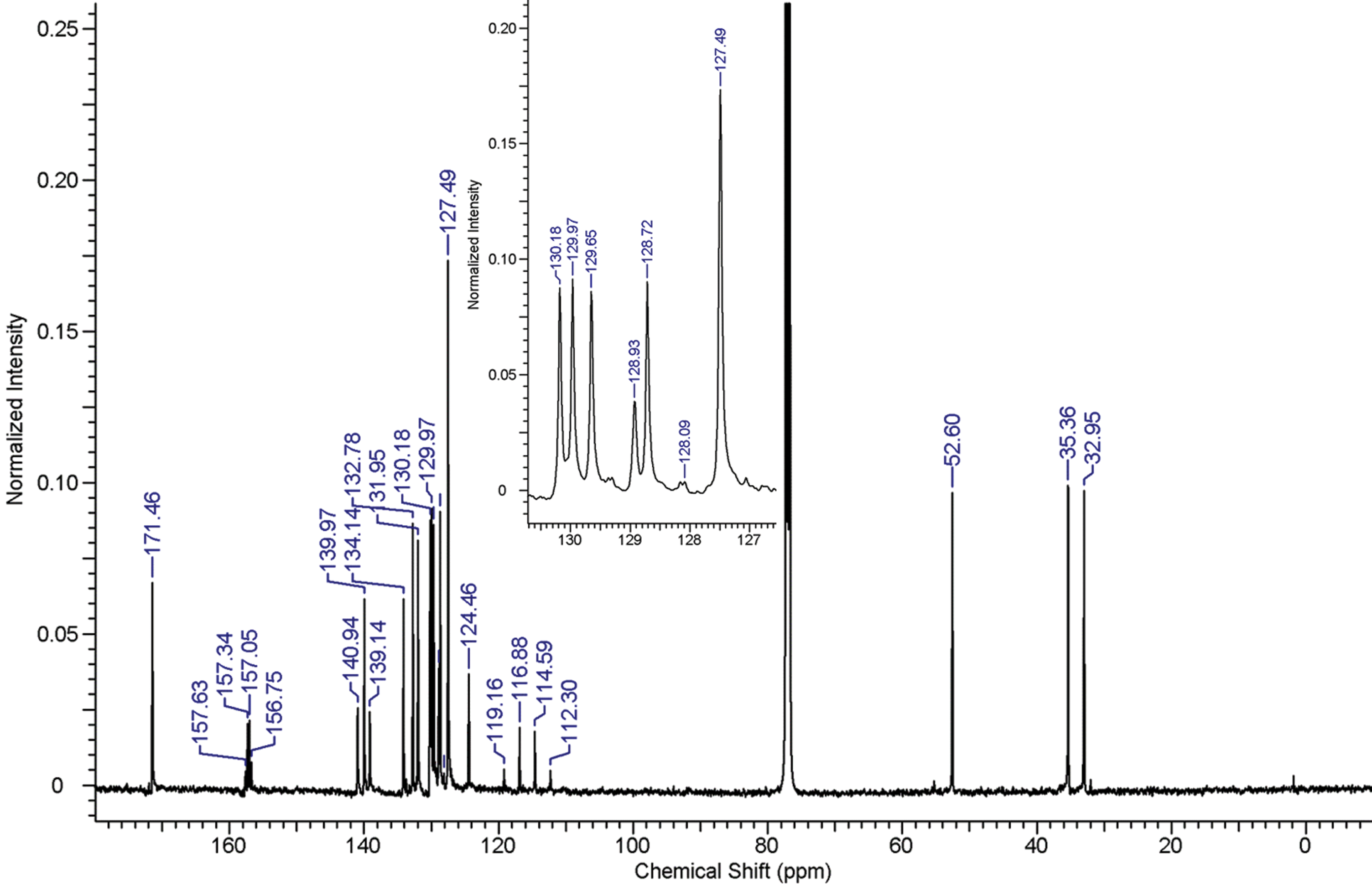

Supplement: S53 Fig — (TIF) [file pone.0144613.s053.tif]

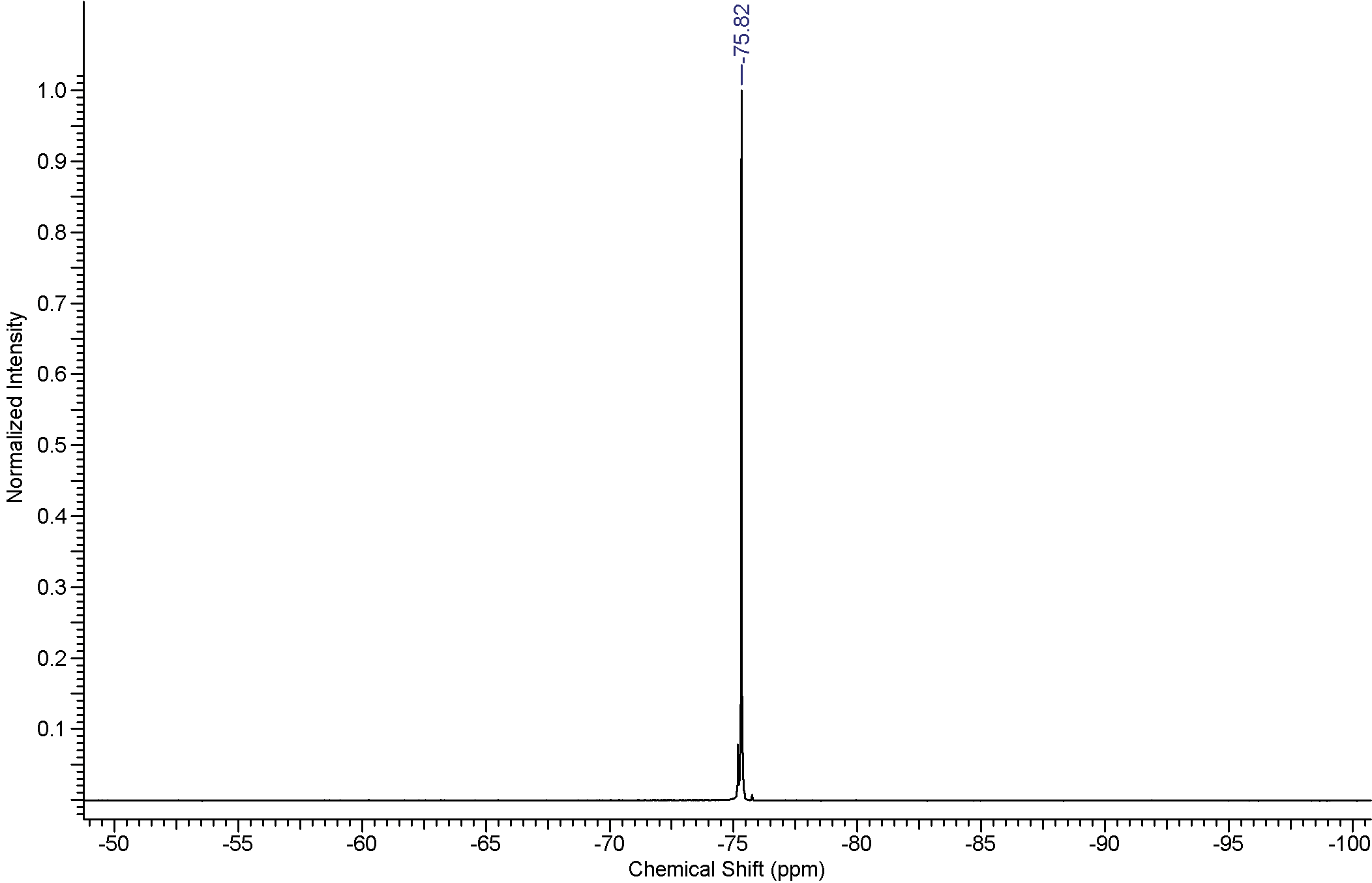

Supplement: S54 Fig — (TIF) [file pone.0144613.s054.tif]

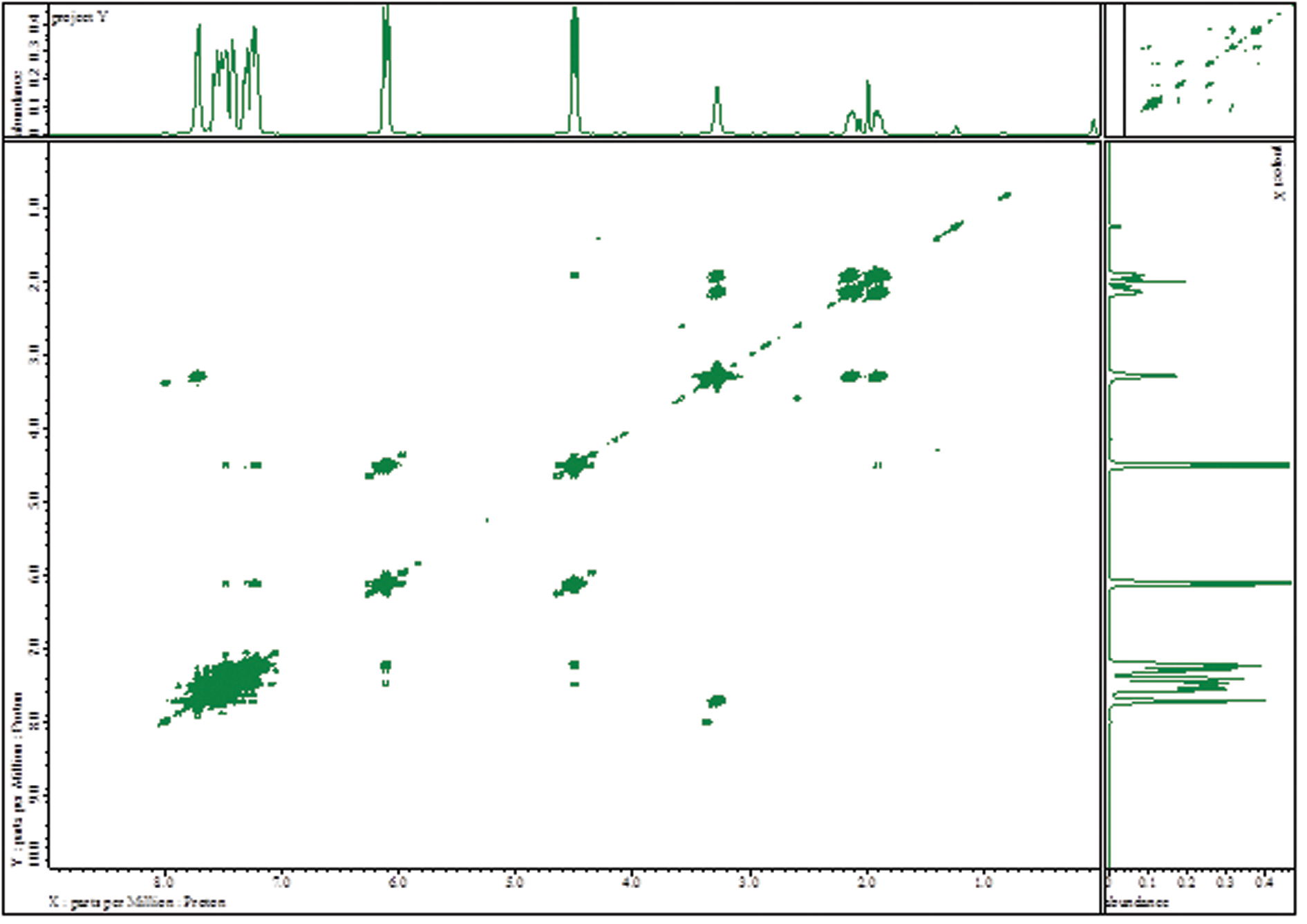

Supplement: S55 Fig — (TIF) [file pone.0144613.s055.tif]

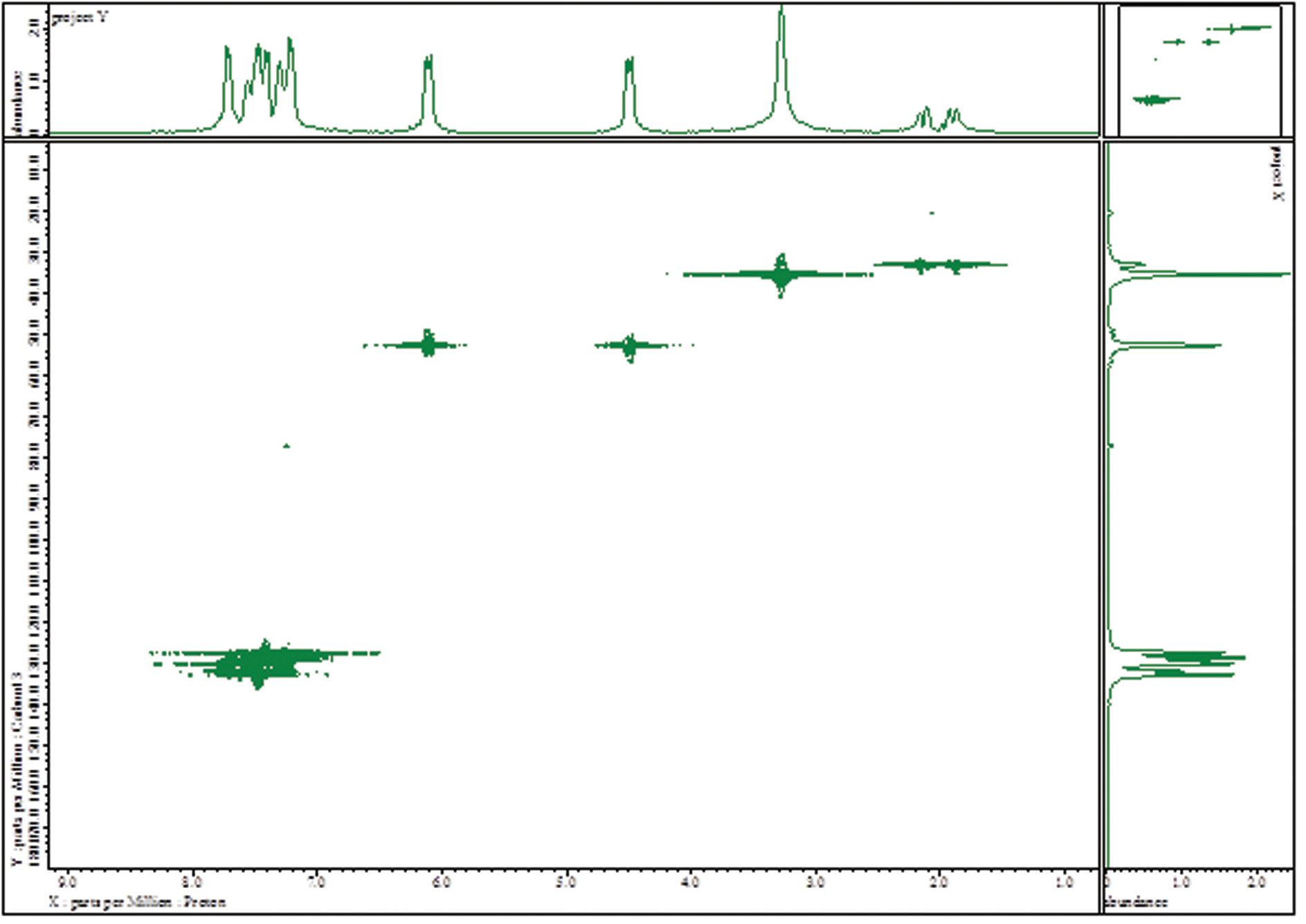

Supplement: S56 Fig — (TIF) [file pone.0144613.s056.tif]

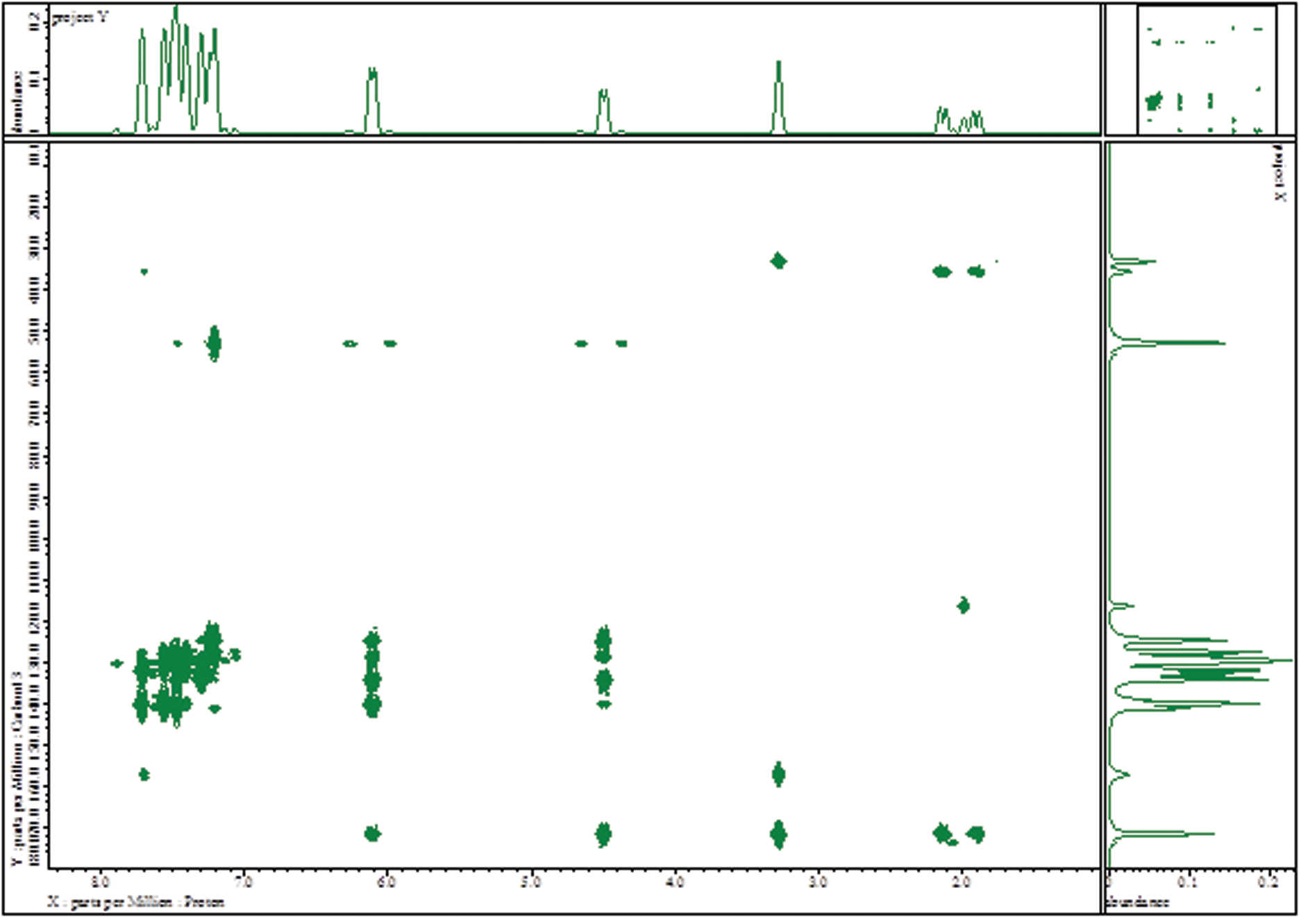

Supplement: S57 Fig — (TIF) [file pone.0144613.s057.tif]

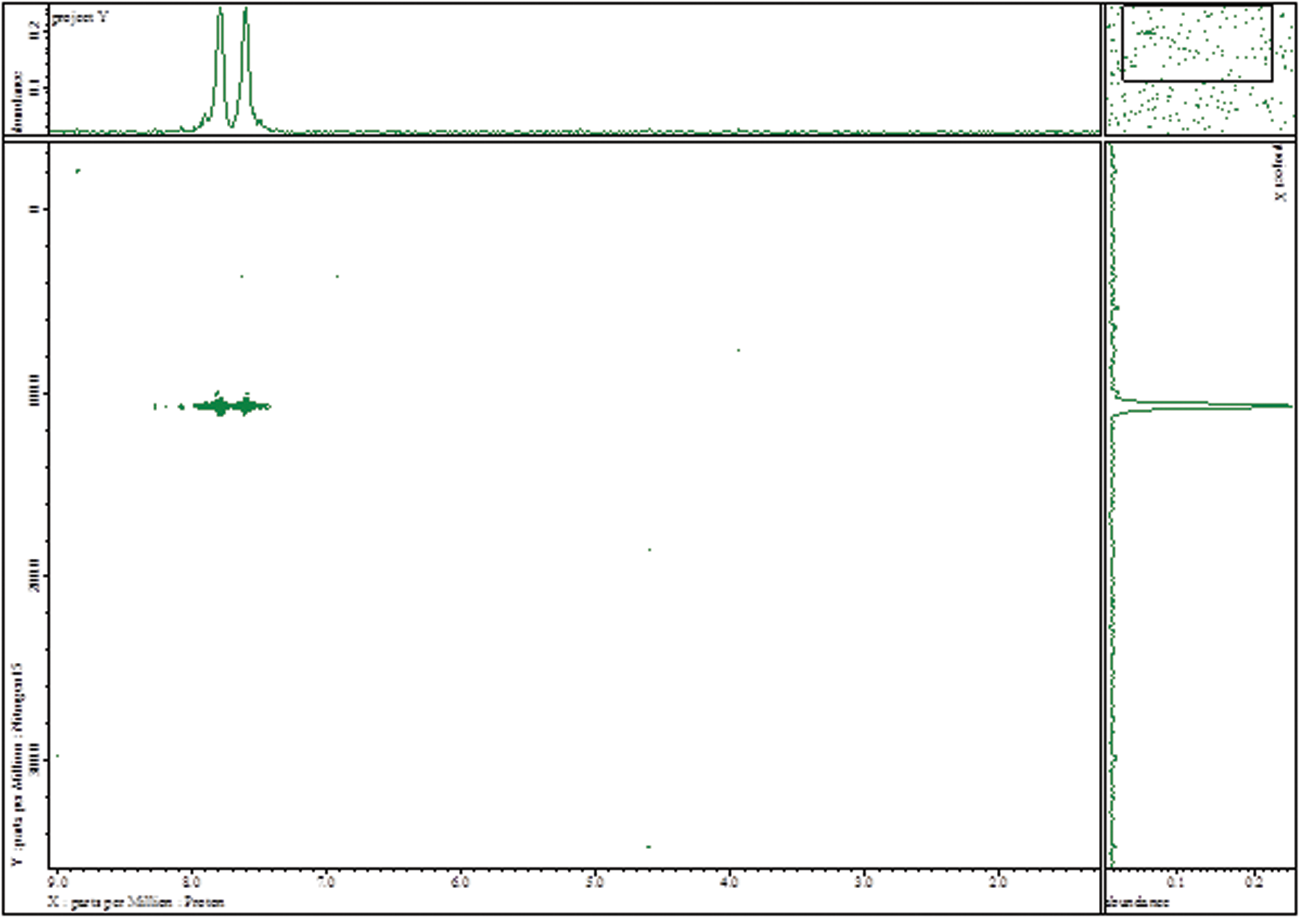

Supplement: S58 Fig — (TIF) [file pone.0144613.s058.tif]

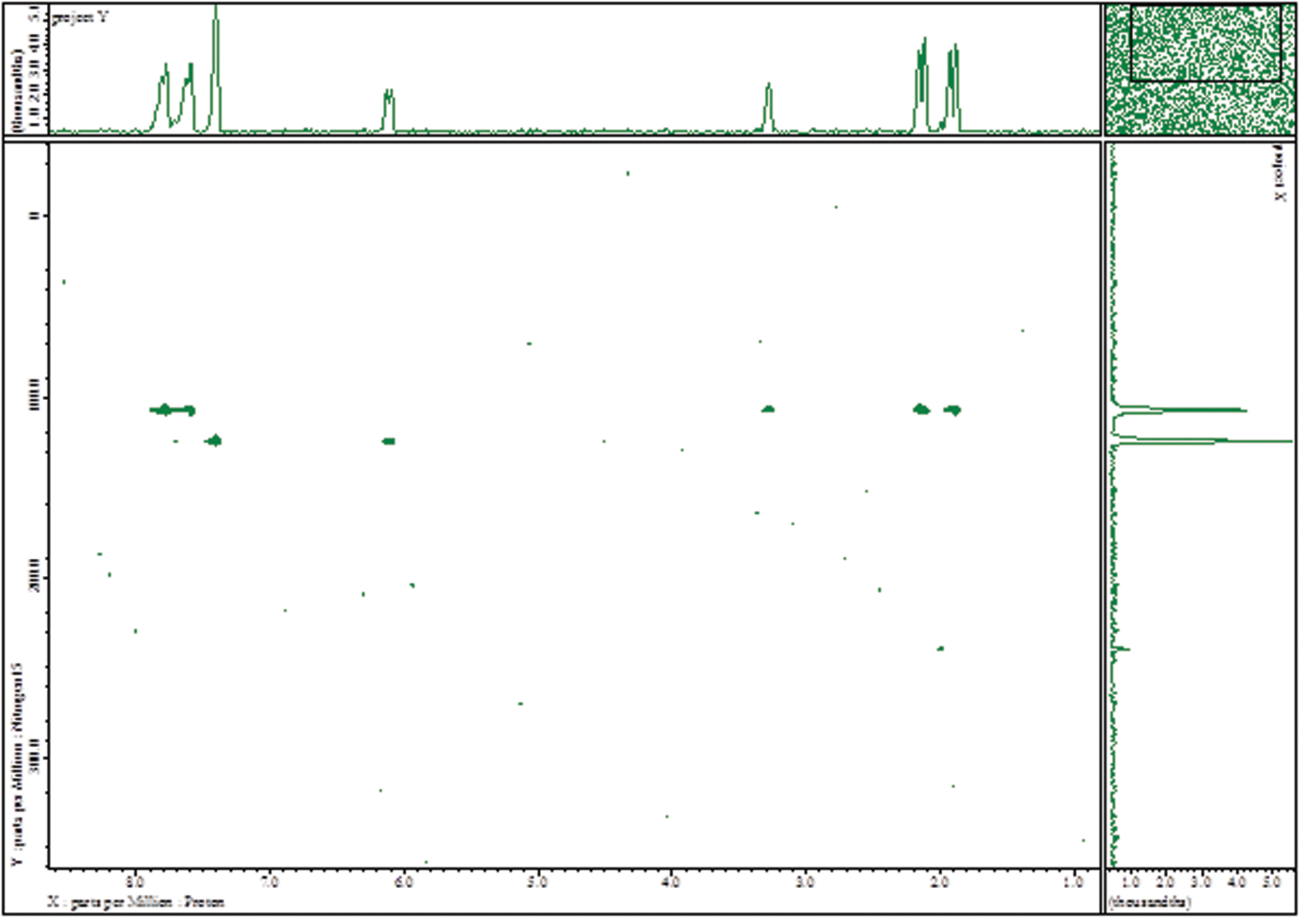

Supplement: S59 Fig — (TIF) [file pone.0144613.s059.tif]

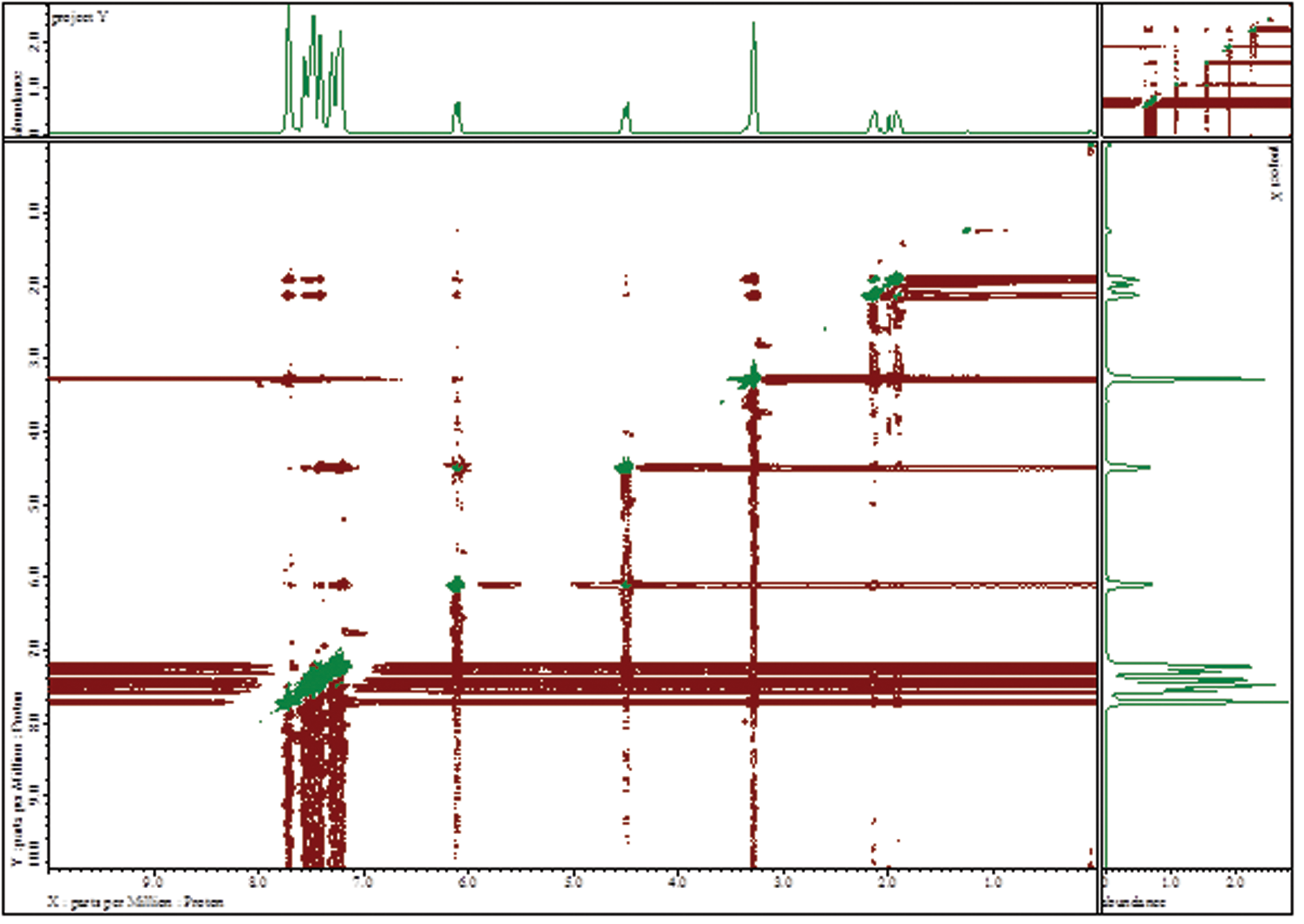

Supplement: S60 Fig — (TIF) [file pone.0144613.s060.tif]

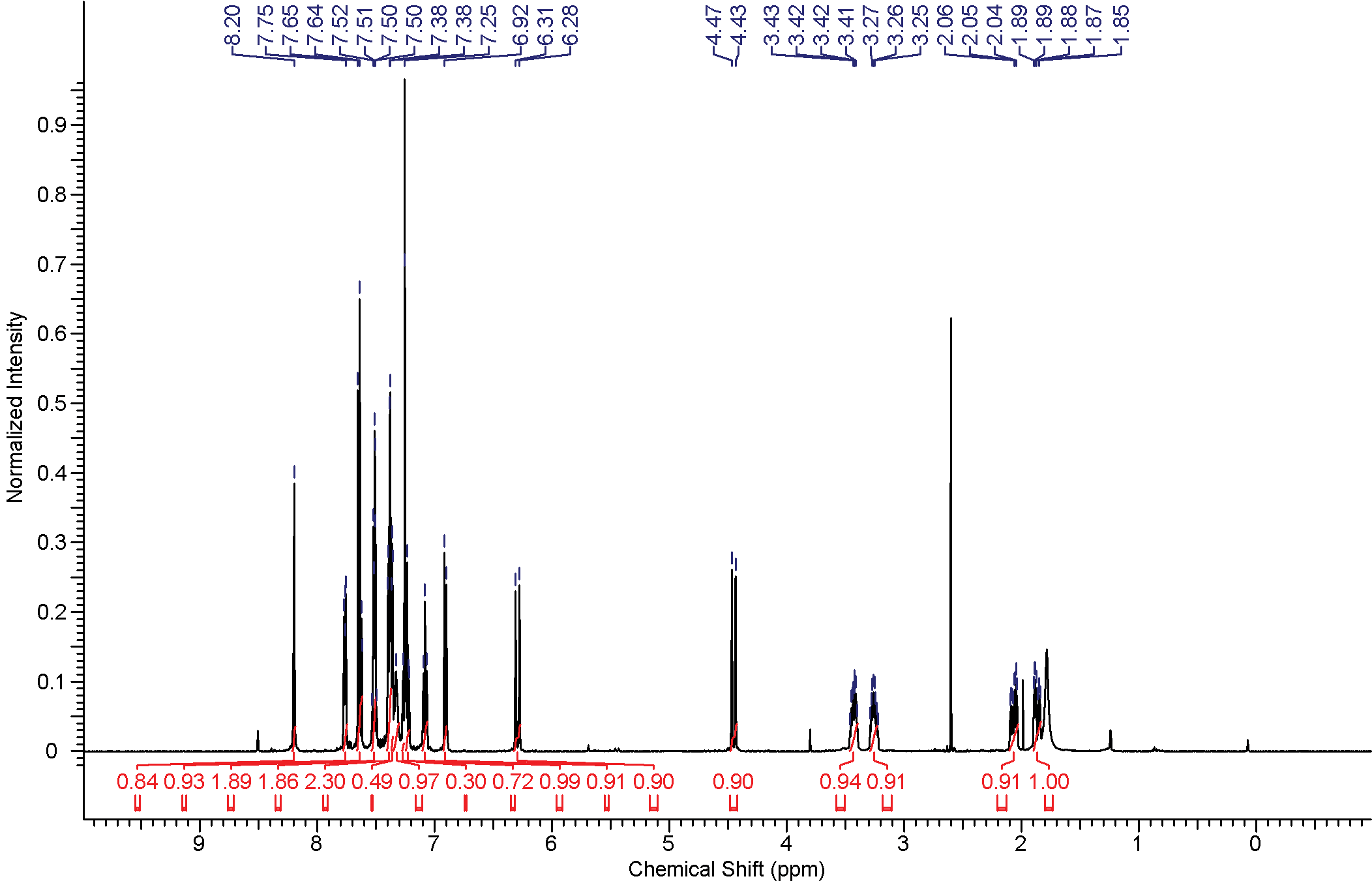

Supplement: S61 Fig — (TIF) [file pone.0144613.s061.tif]

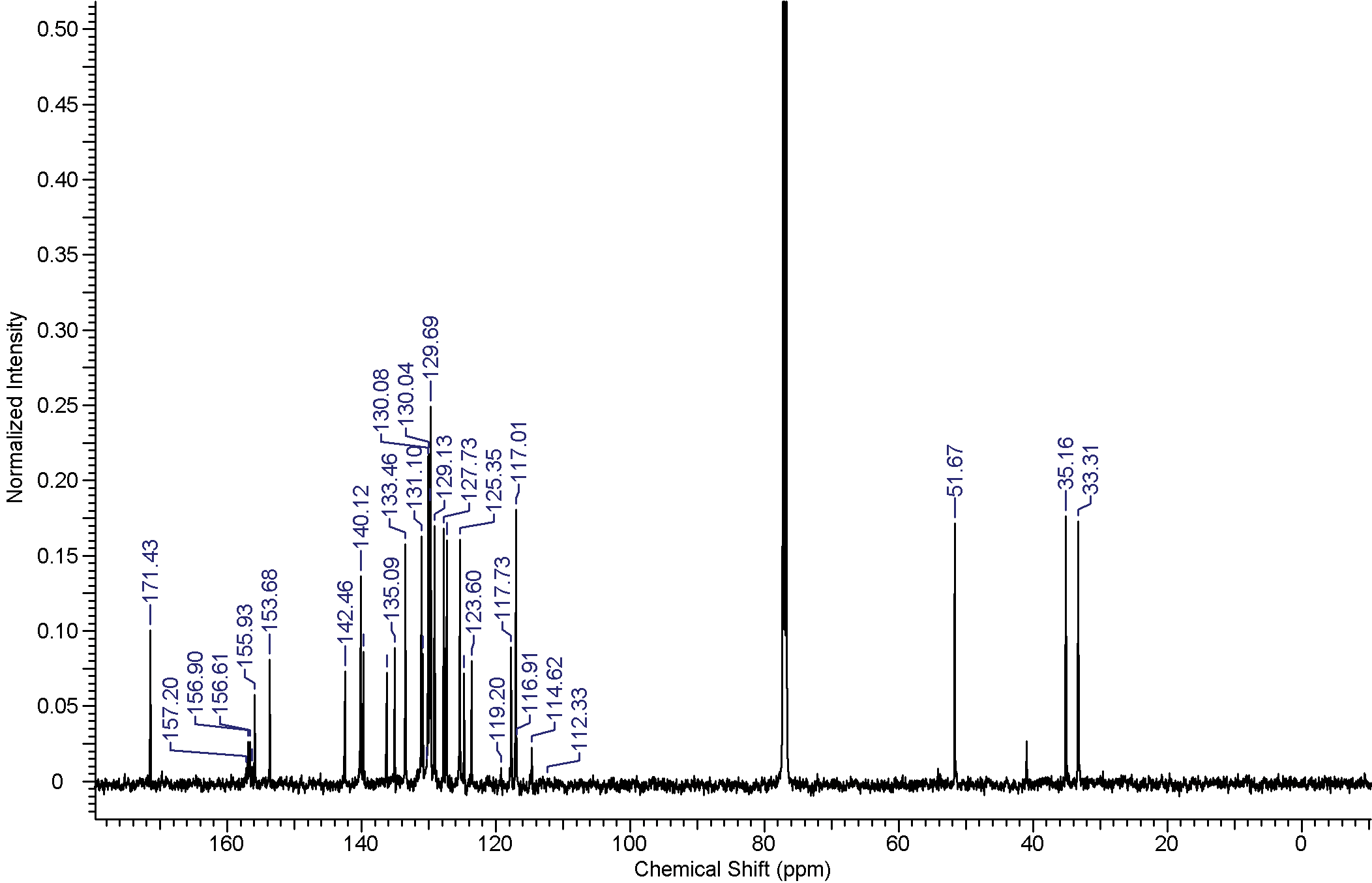

Supplement: S62 Fig — (TIF) [file pone.0144613.s062.tif]

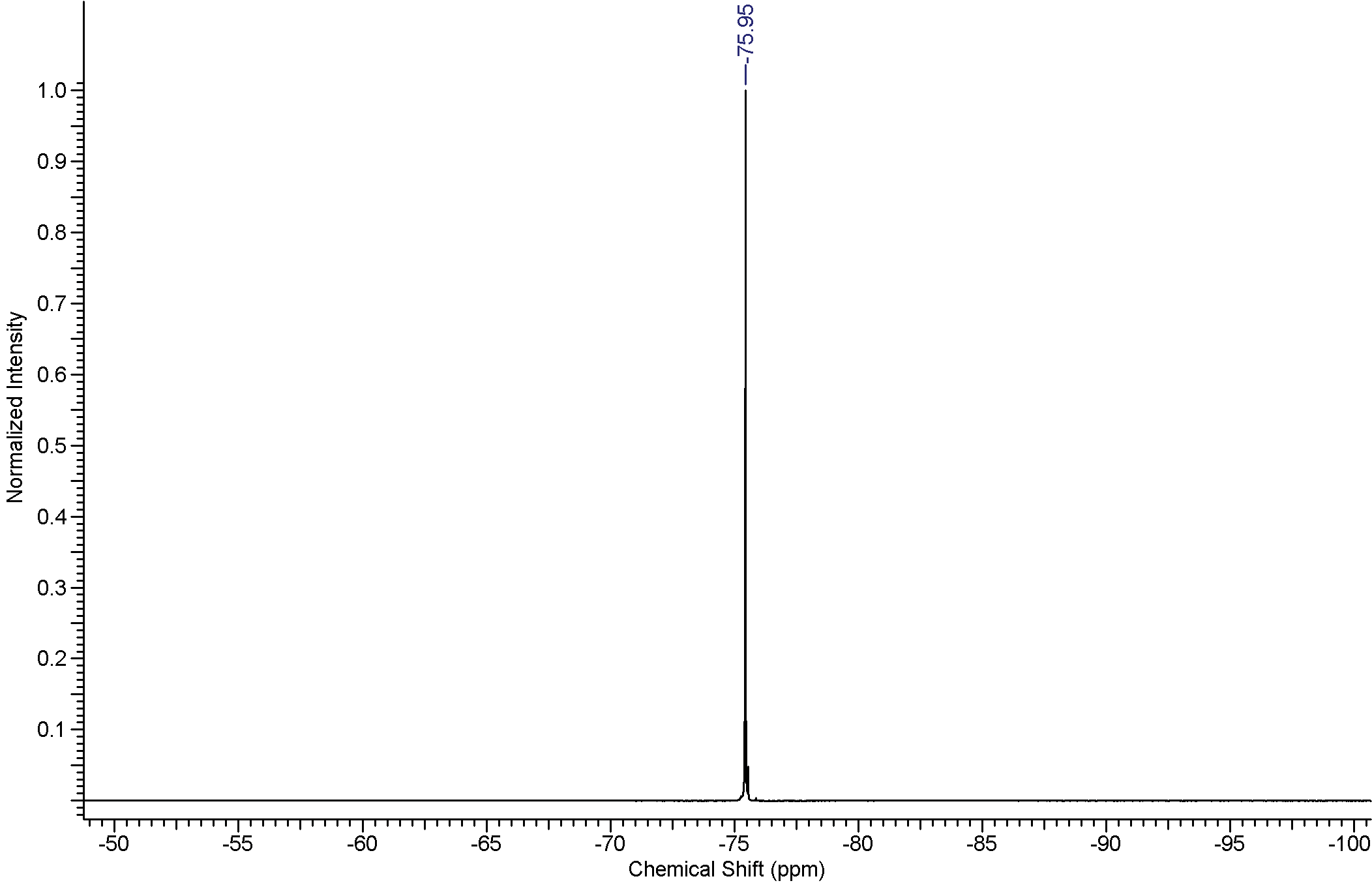

Supplement: S63 Fig — (TIF) [file pone.0144613.s063.tif]

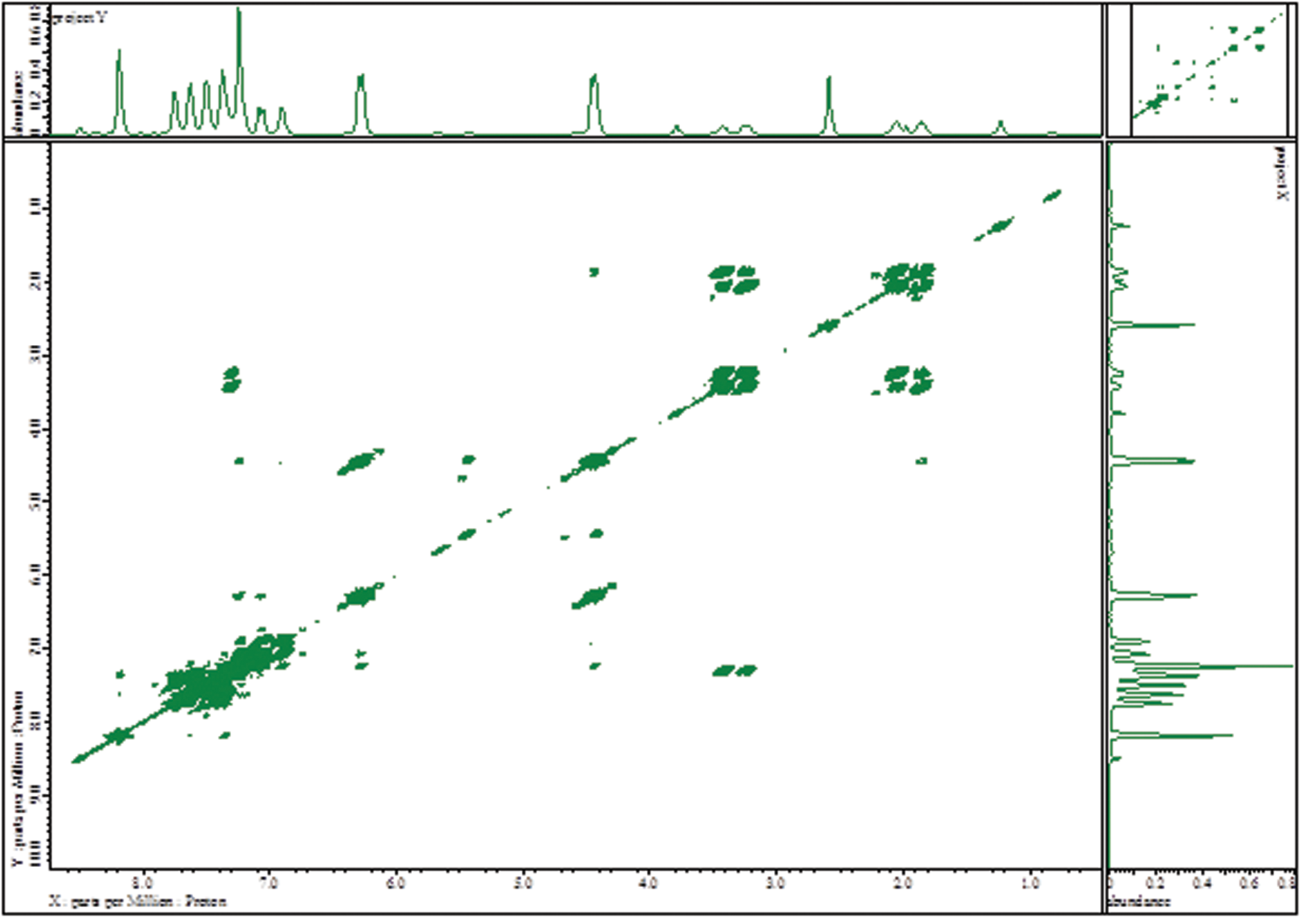

Supplement: S64 Fig — (TIF) [file pone.0144613.s064.tif]

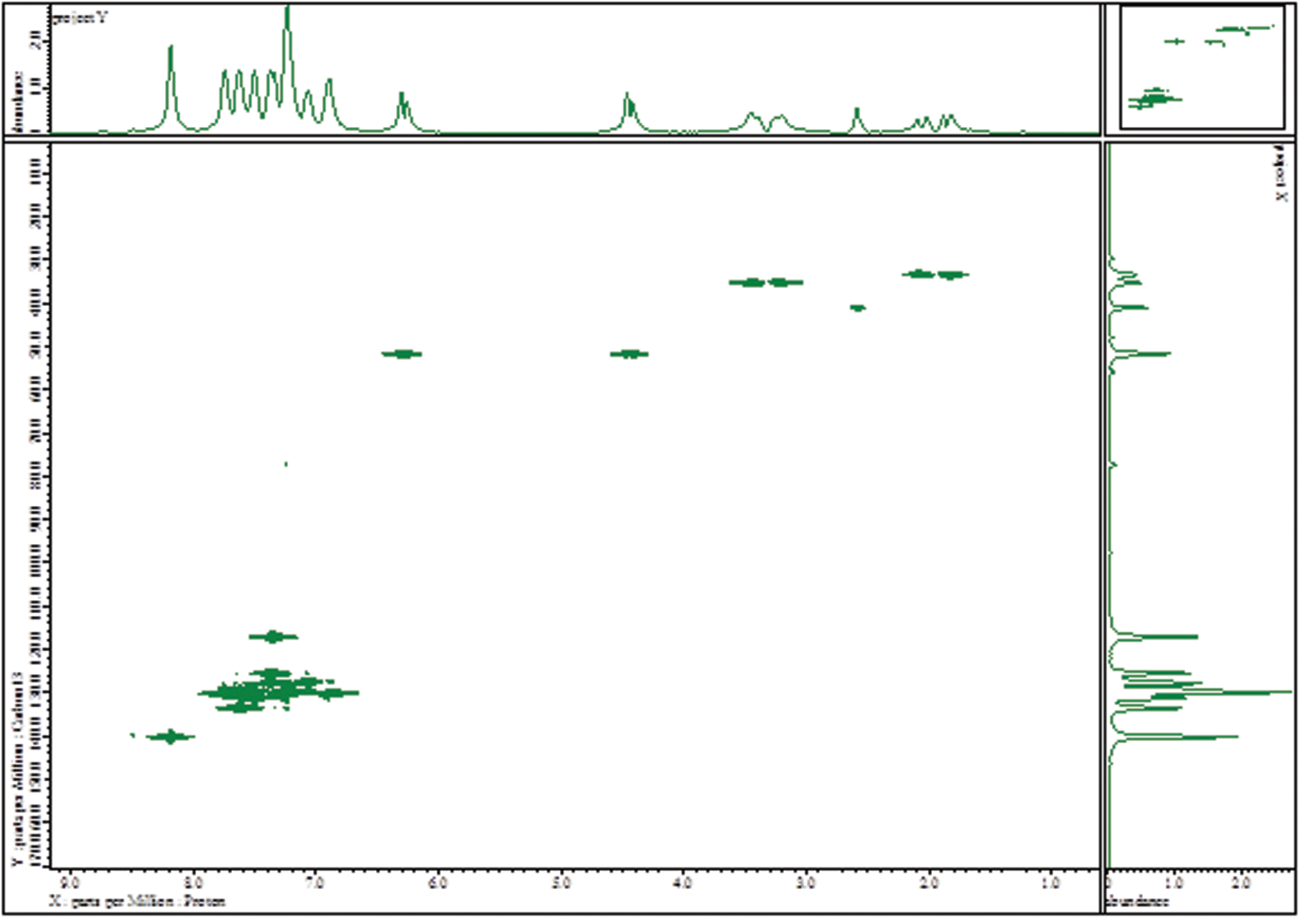

Supplement: S65 Fig — (TIF) [file pone.0144613.s065.tif]

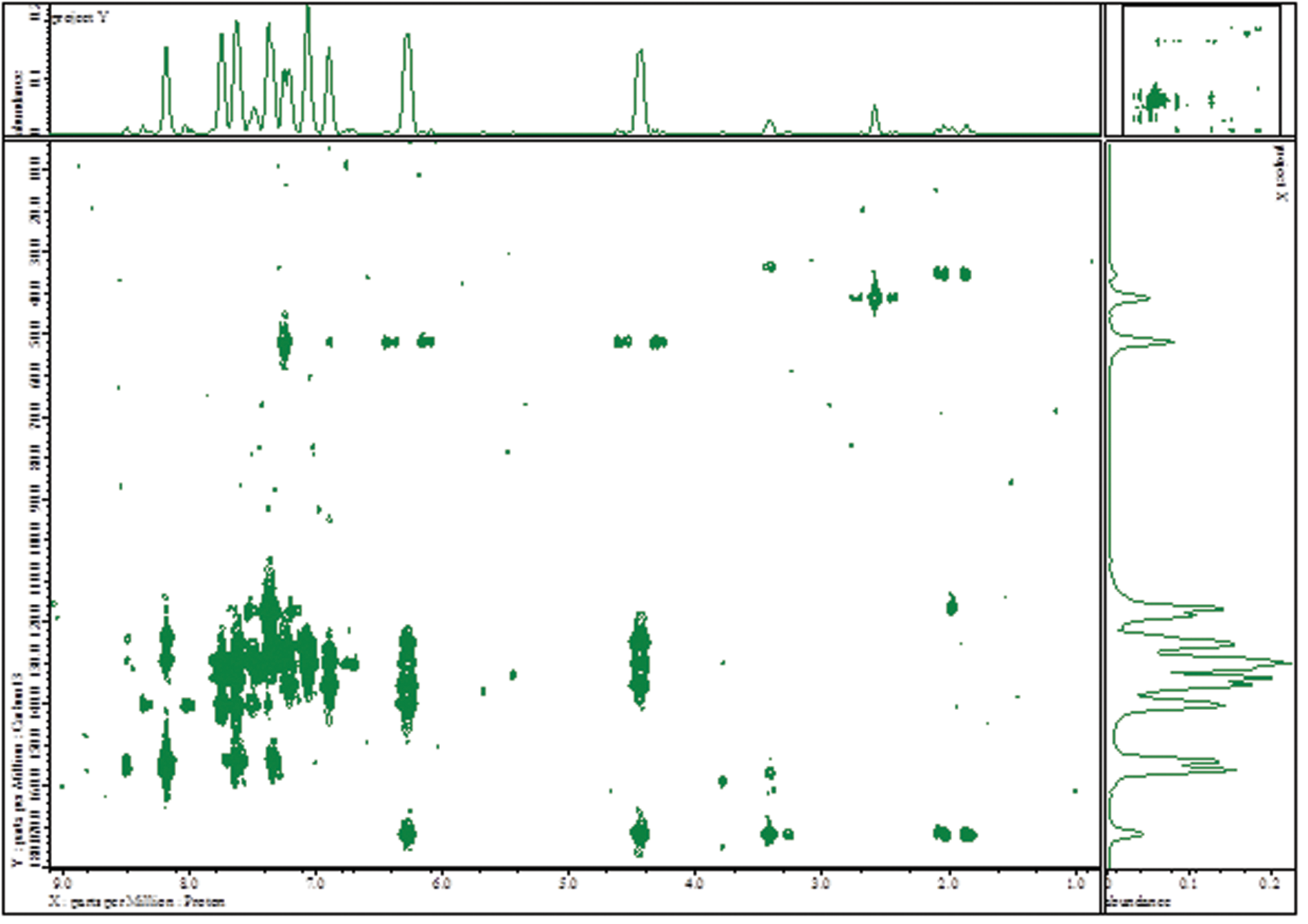

Supplement: S66 Fig — (TIF) [file pone.0144613.s066.tif]

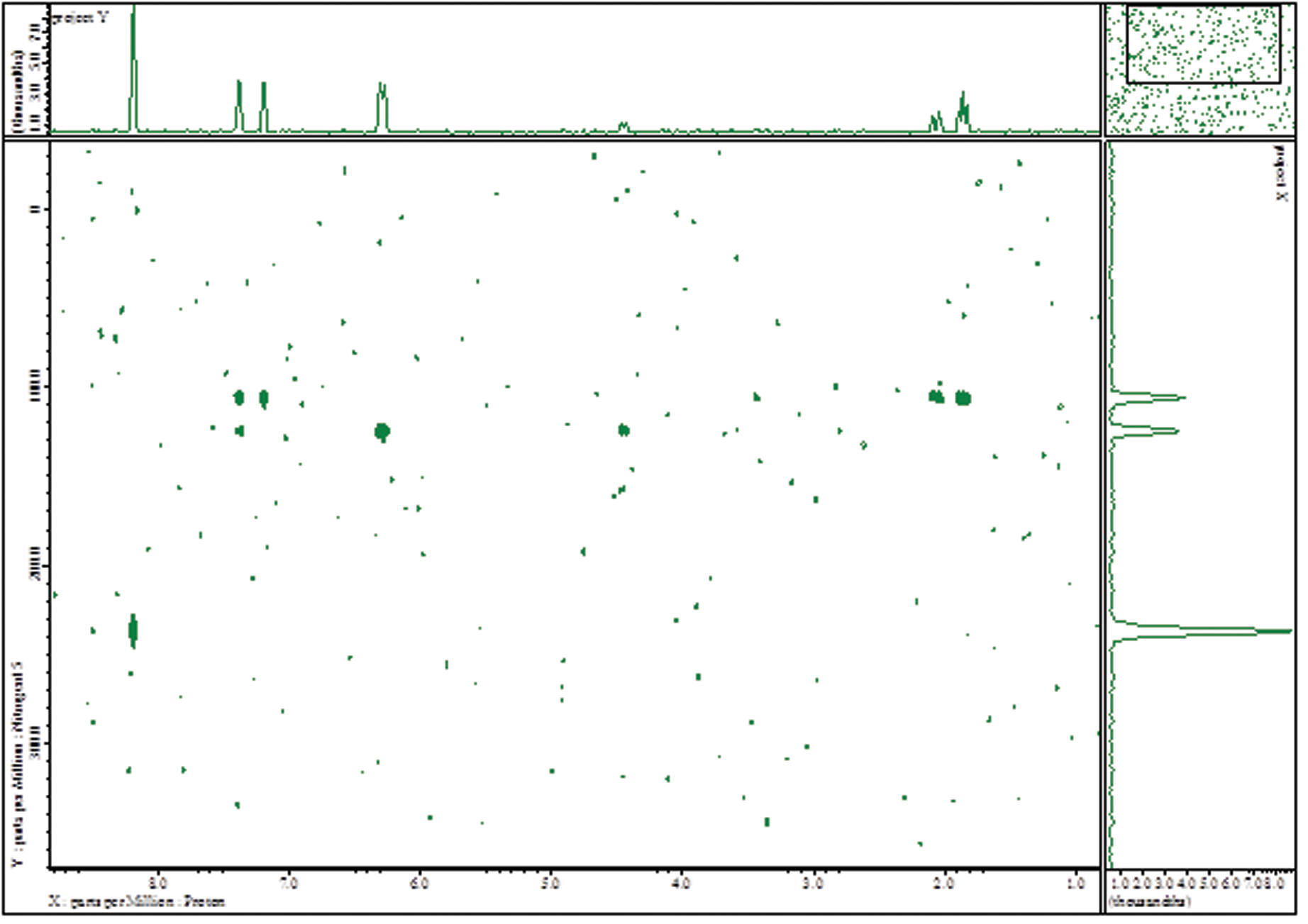

Supplement: S67 Fig — (TIF) [file pone.0144613.s067.tif]

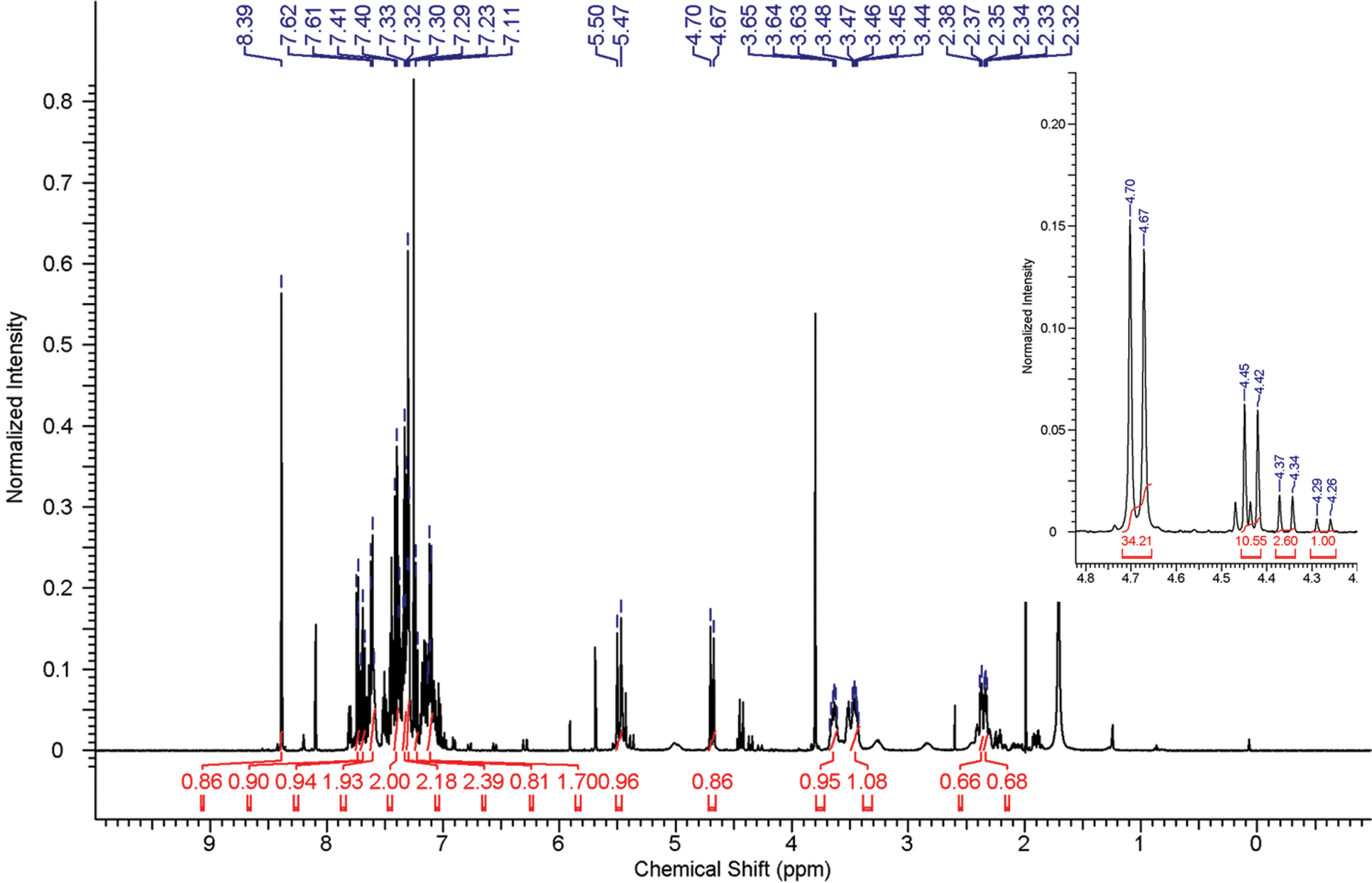

Supplement: S68 Fig — (TIF) [file pone.0144613.s068.tif]

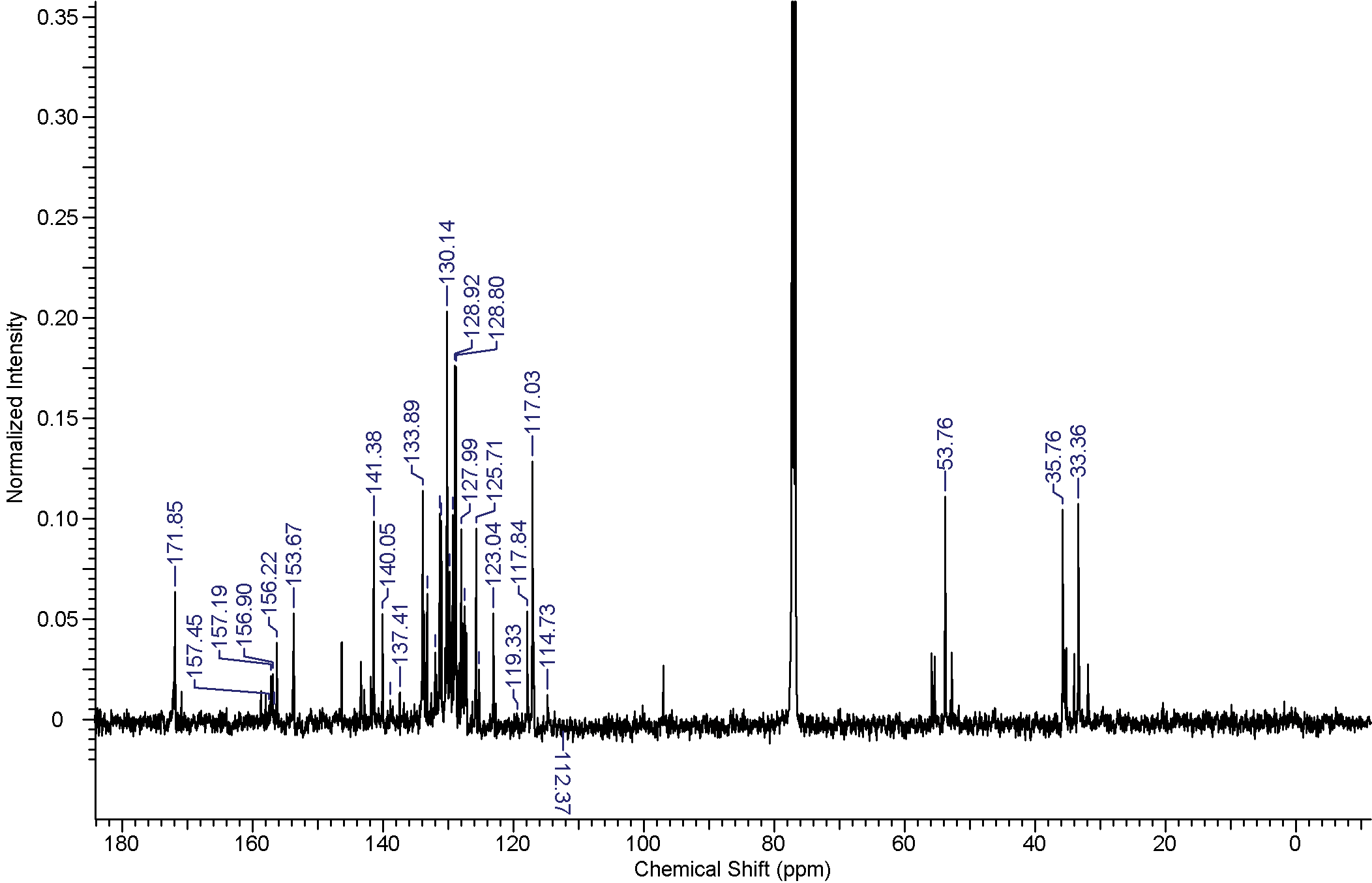

Supplement: S69 Fig — (TIF) [file pone.0144613.s069.tif]

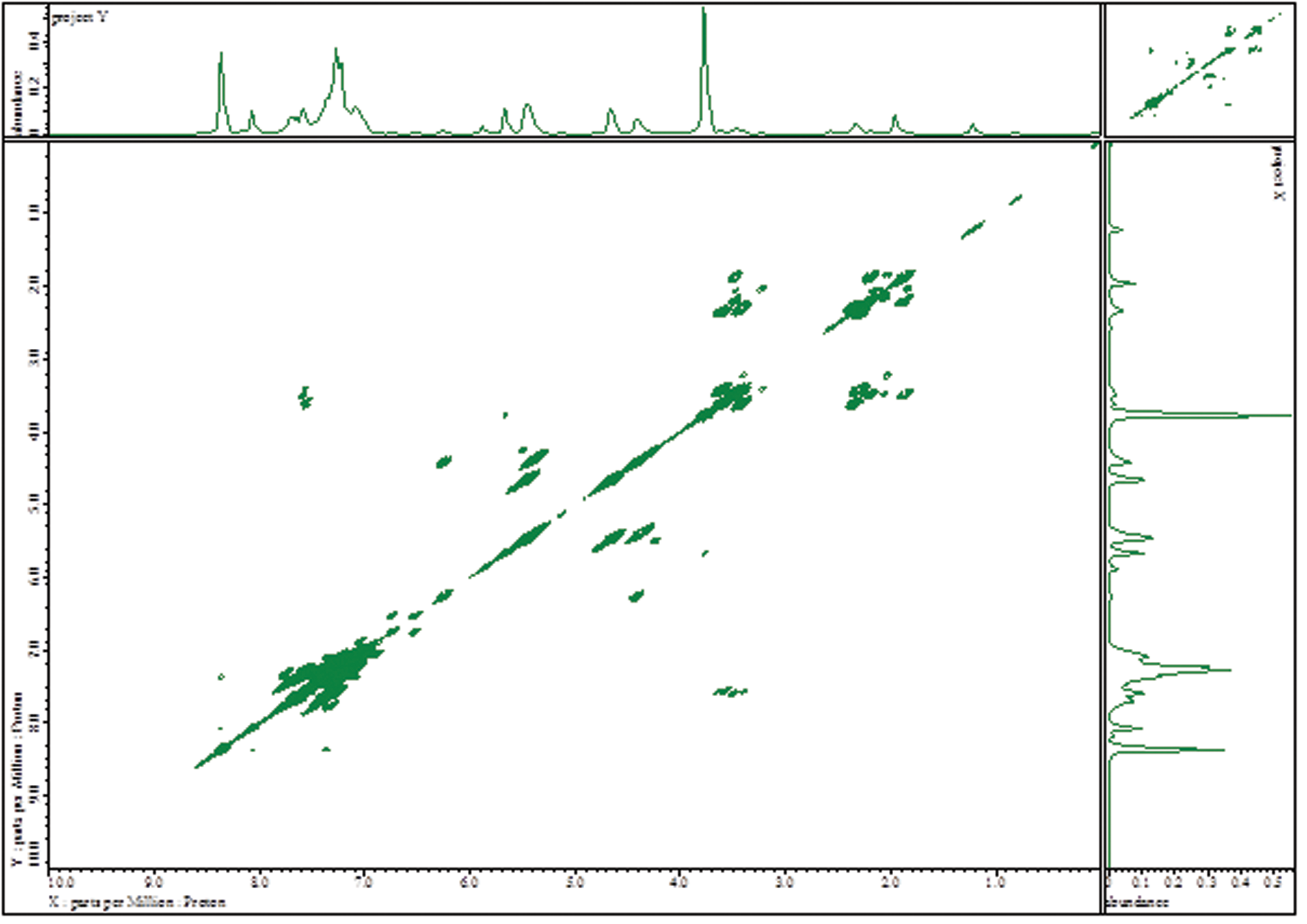

Supplement: S70 Fig — (TIF) [file pone.0144613.s070.tif]

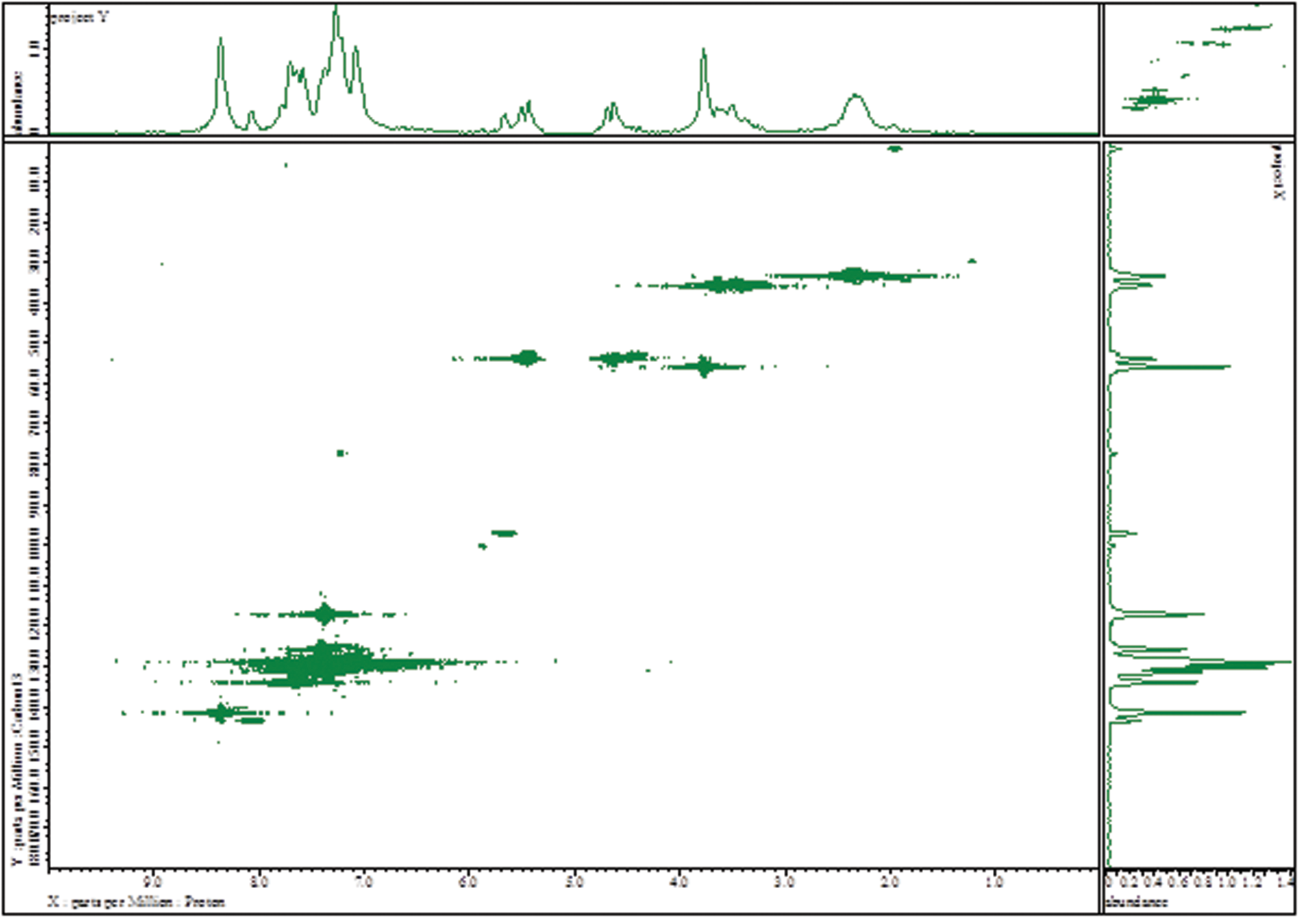

Supplement: S71 Fig — (TIF) [file pone.0144613.s071.tif]

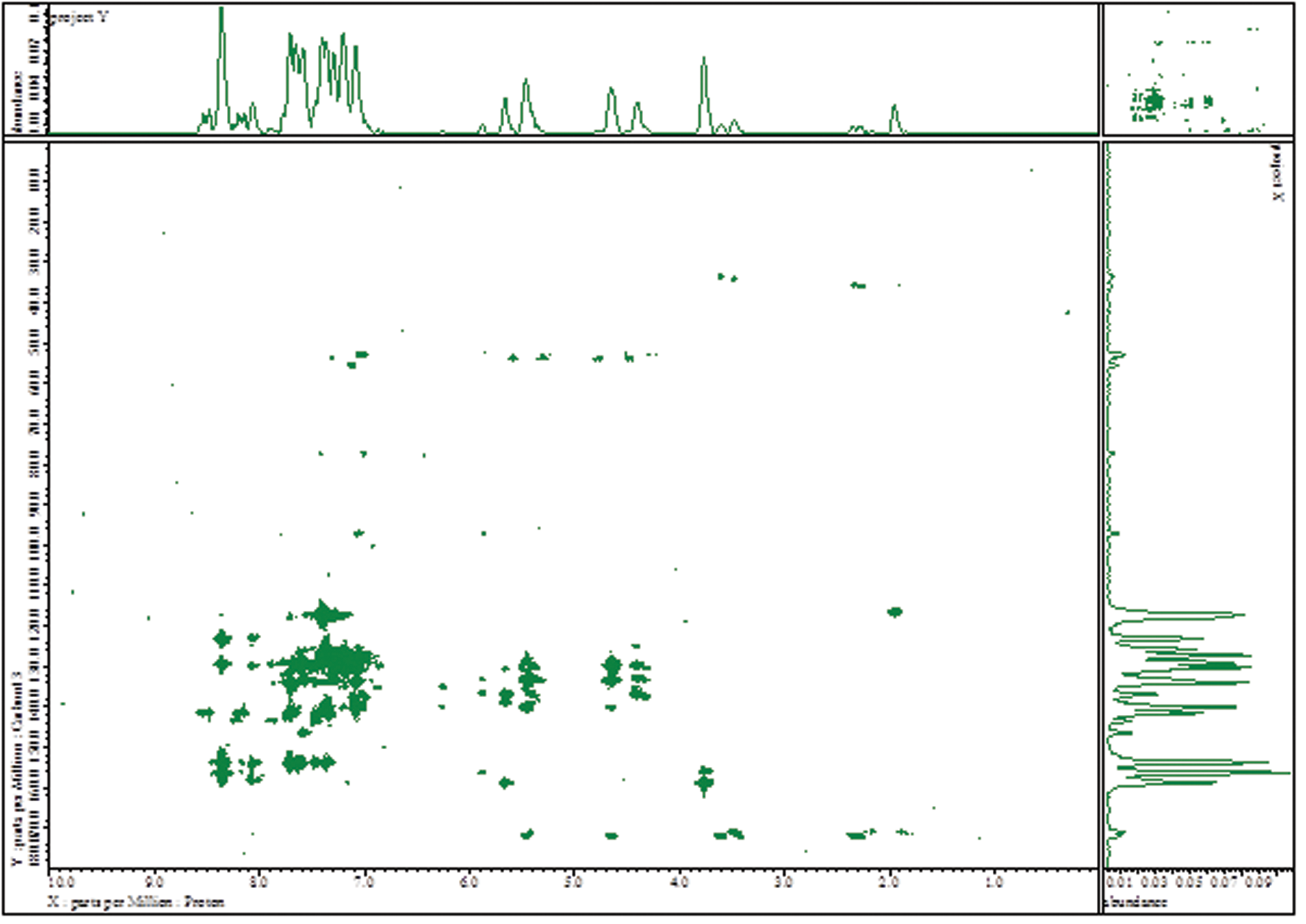

Supplement: S72 Fig — (TIF) [file pone.0144613.s072.tif]

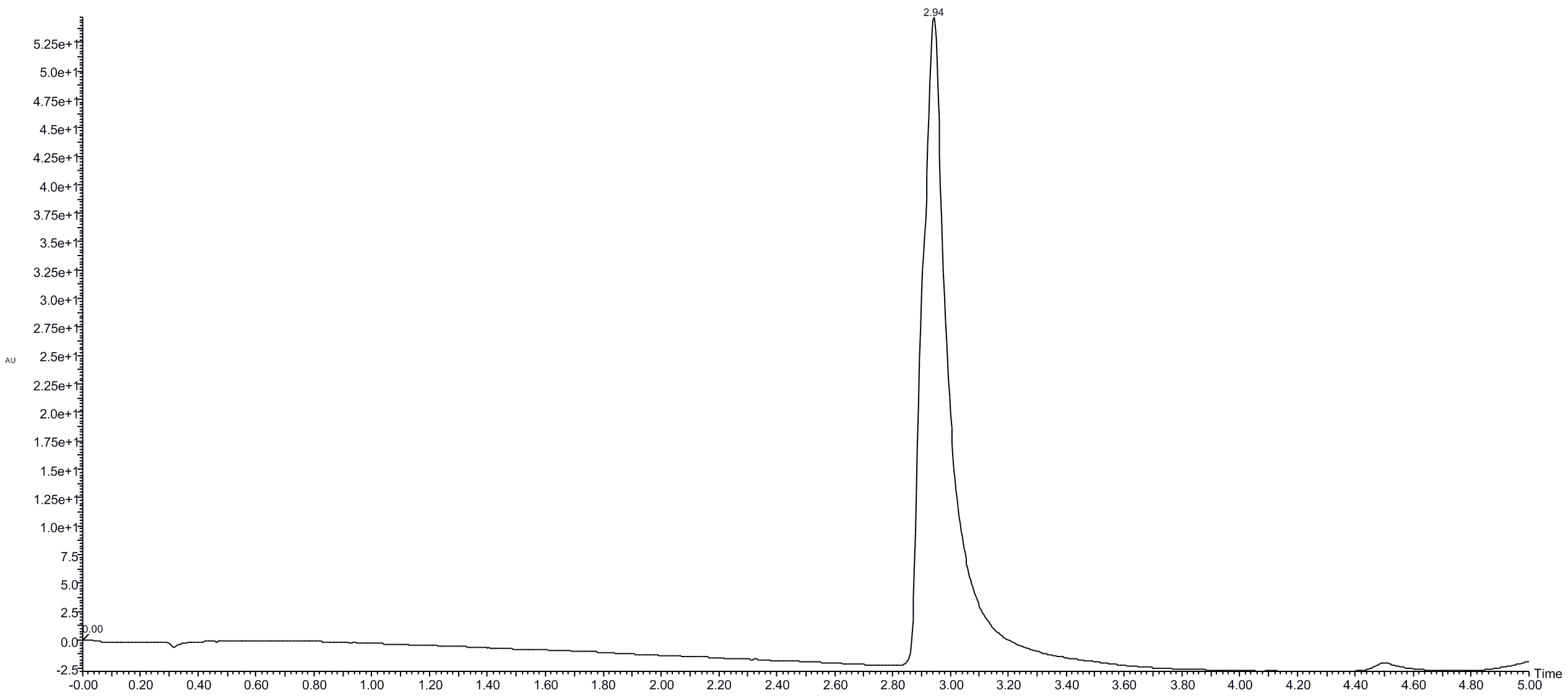

Supplement: S73 Fig — (TIF) [file pone.0144613.s073.tif]

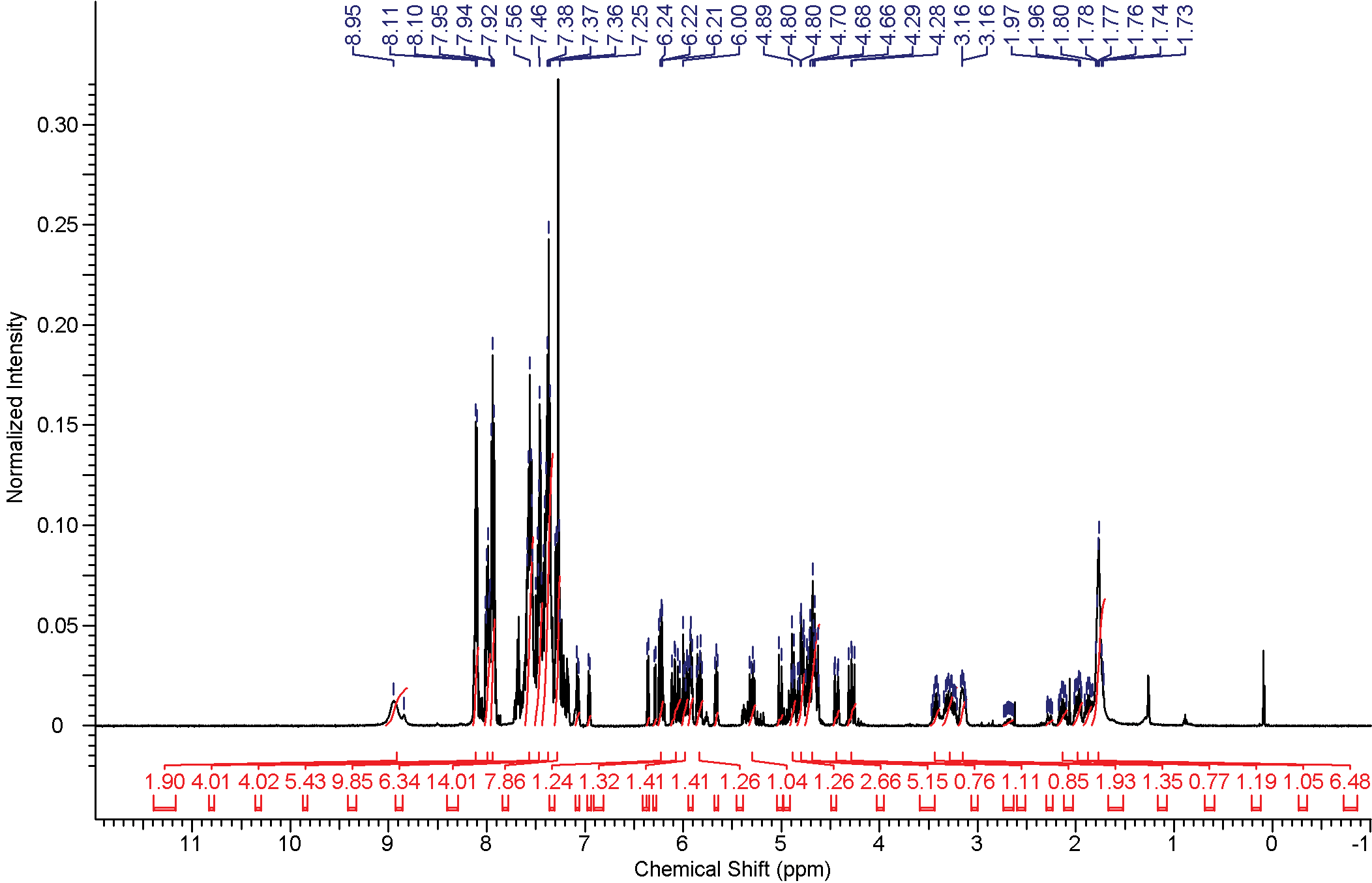

Supplement: S74 Fig — (TIF) [file pone.0144613.s074.tif]

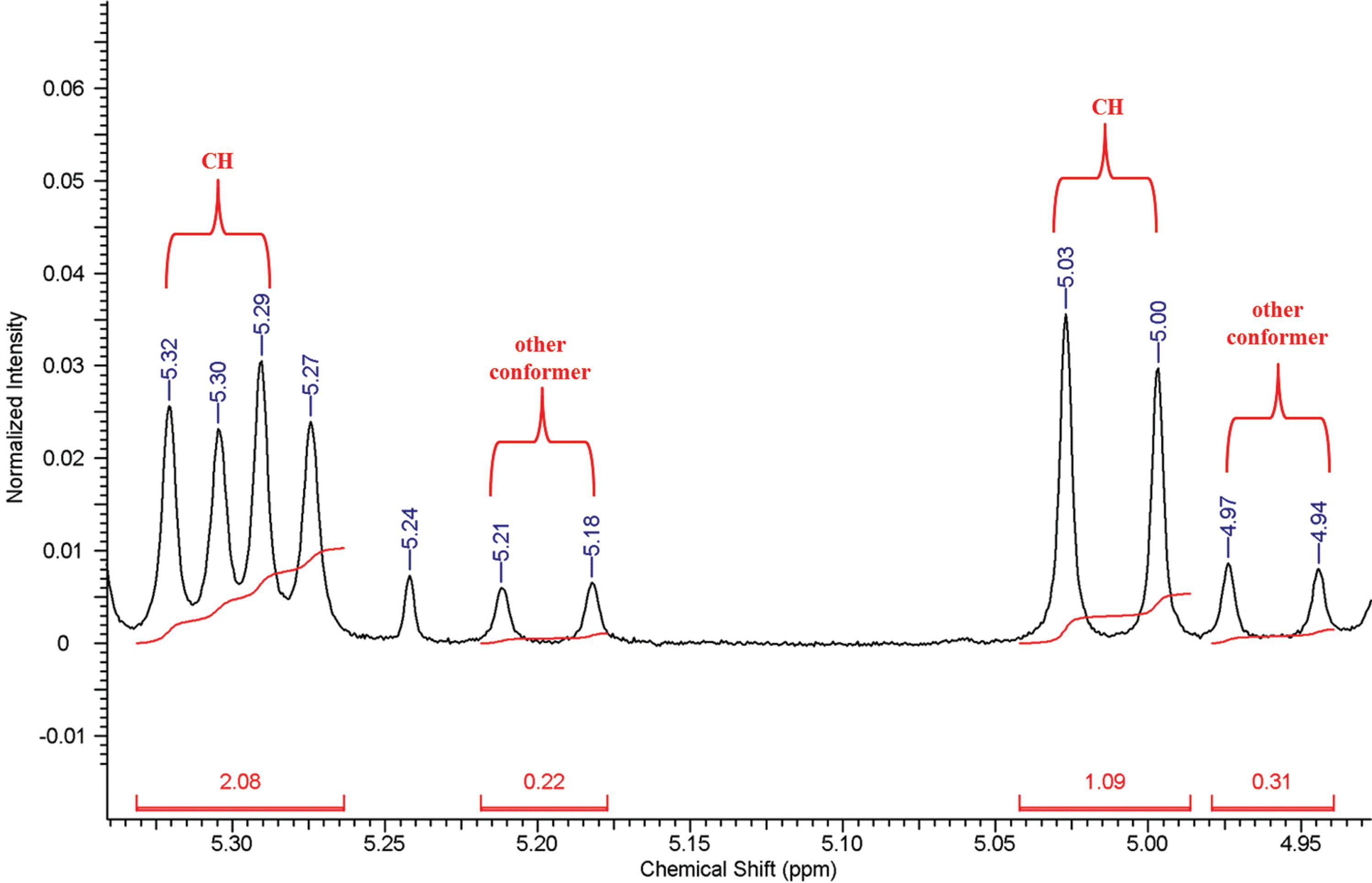

Supplement: S75 Fig — (TIF) [file pone.0144613.s075.tif]

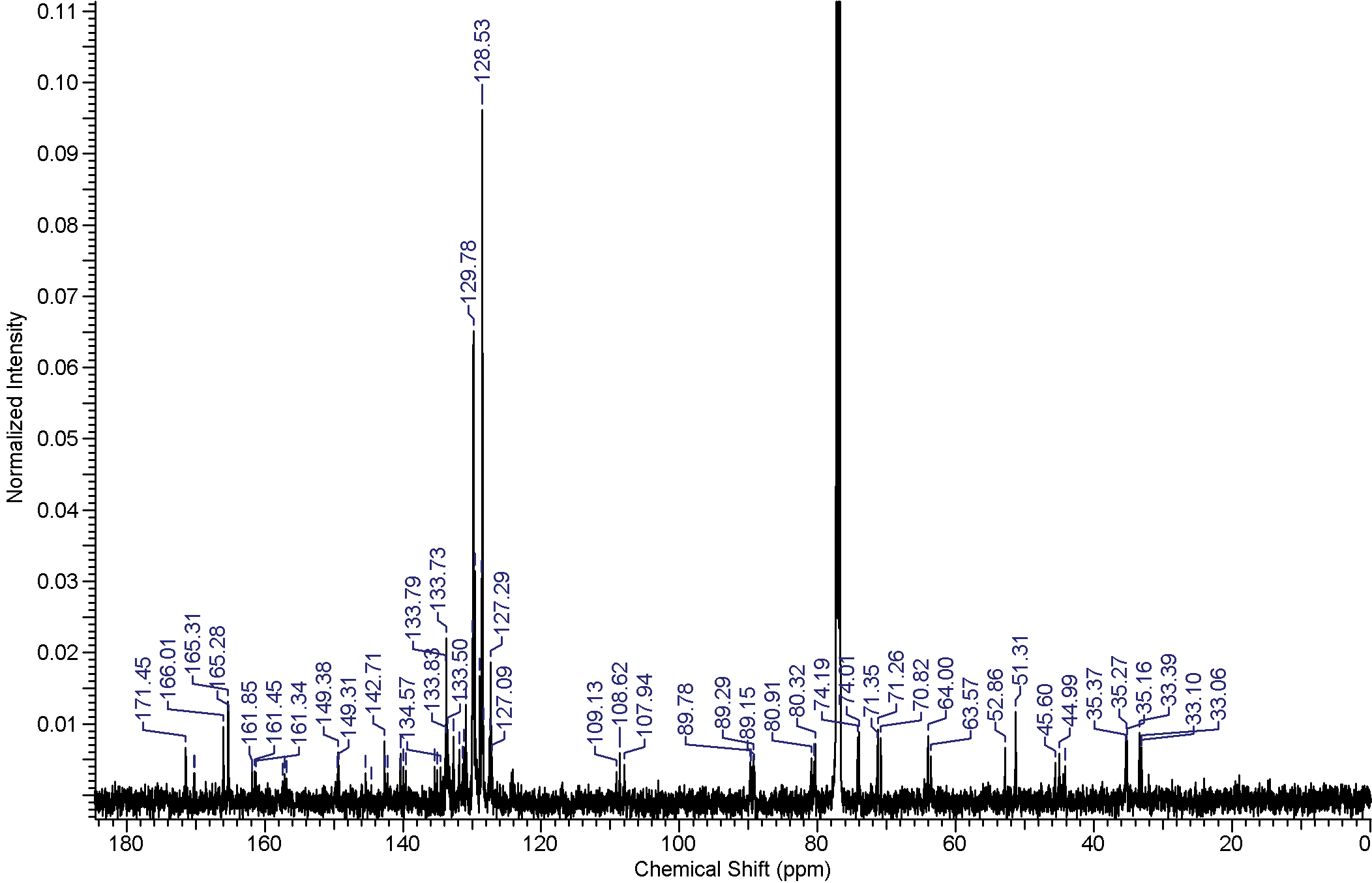

Supplement: S76 Fig — (TIF) [file pone.0144613.s076.tif]

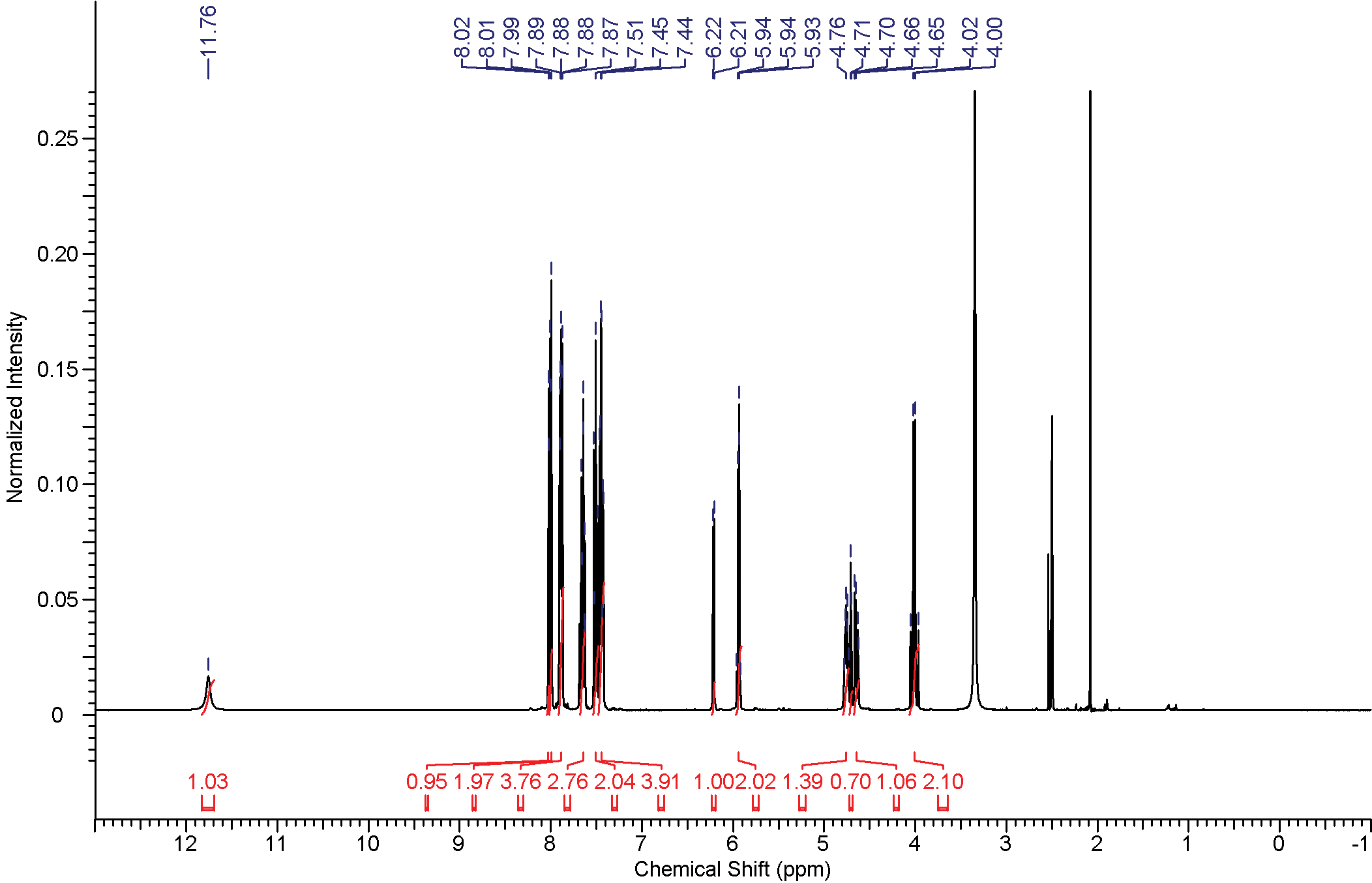

Supplement: S77 Fig — (TIF) [file pone.0144613.s077.tif]

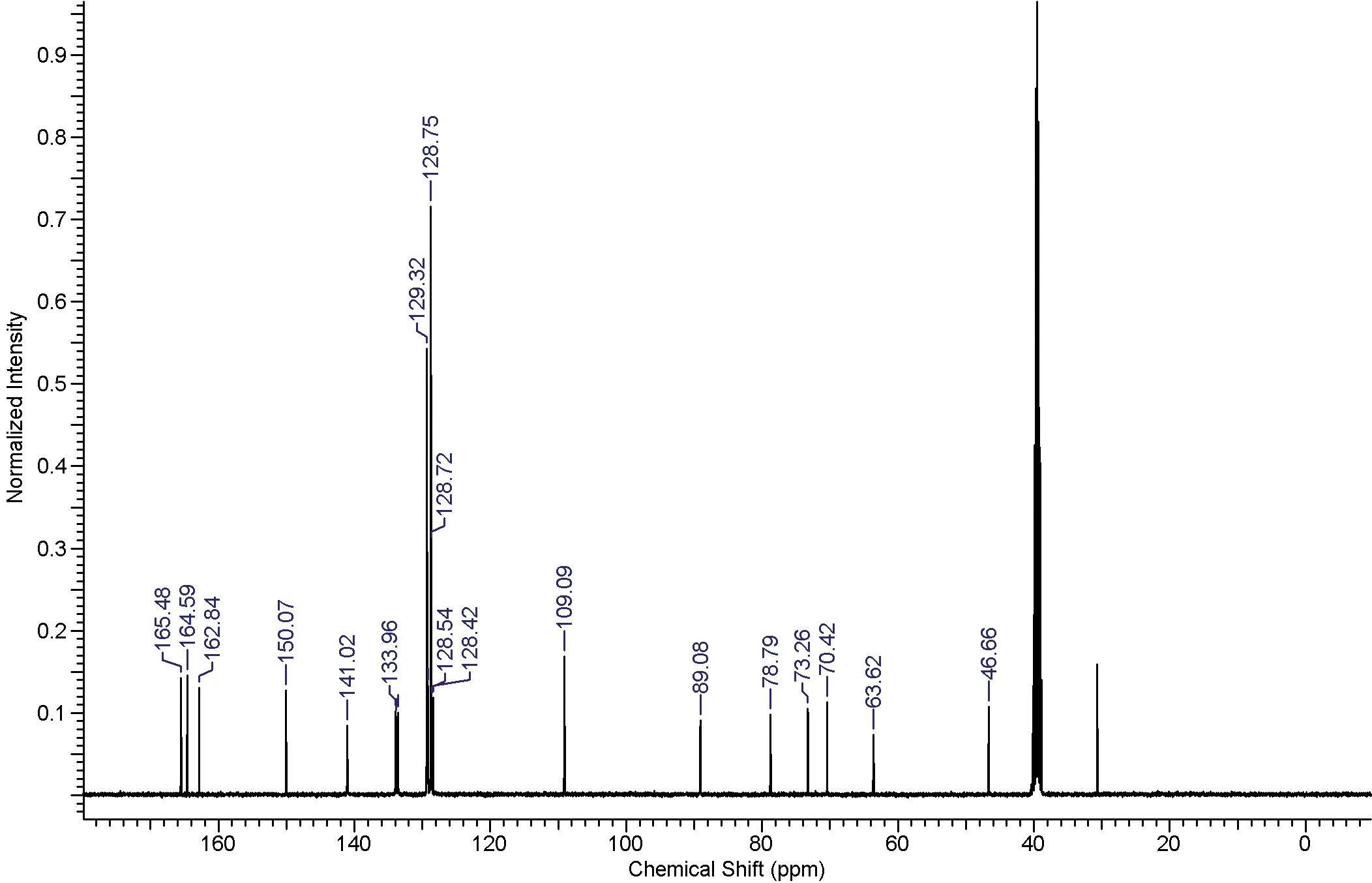

Supplement: S78 Fig — (TIF) [file pone.0144613.s078.tif]

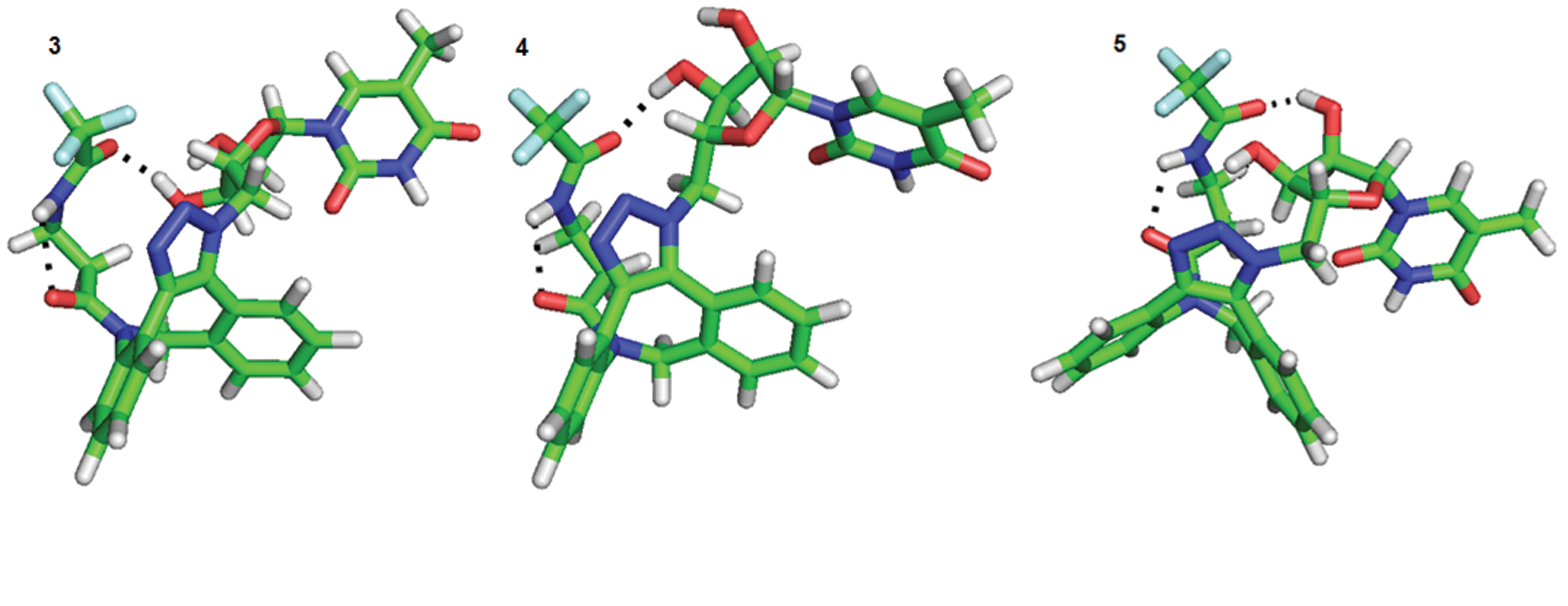

Supplement: S79 Fig — (TIF) [file pone.0144613.s079.tif]

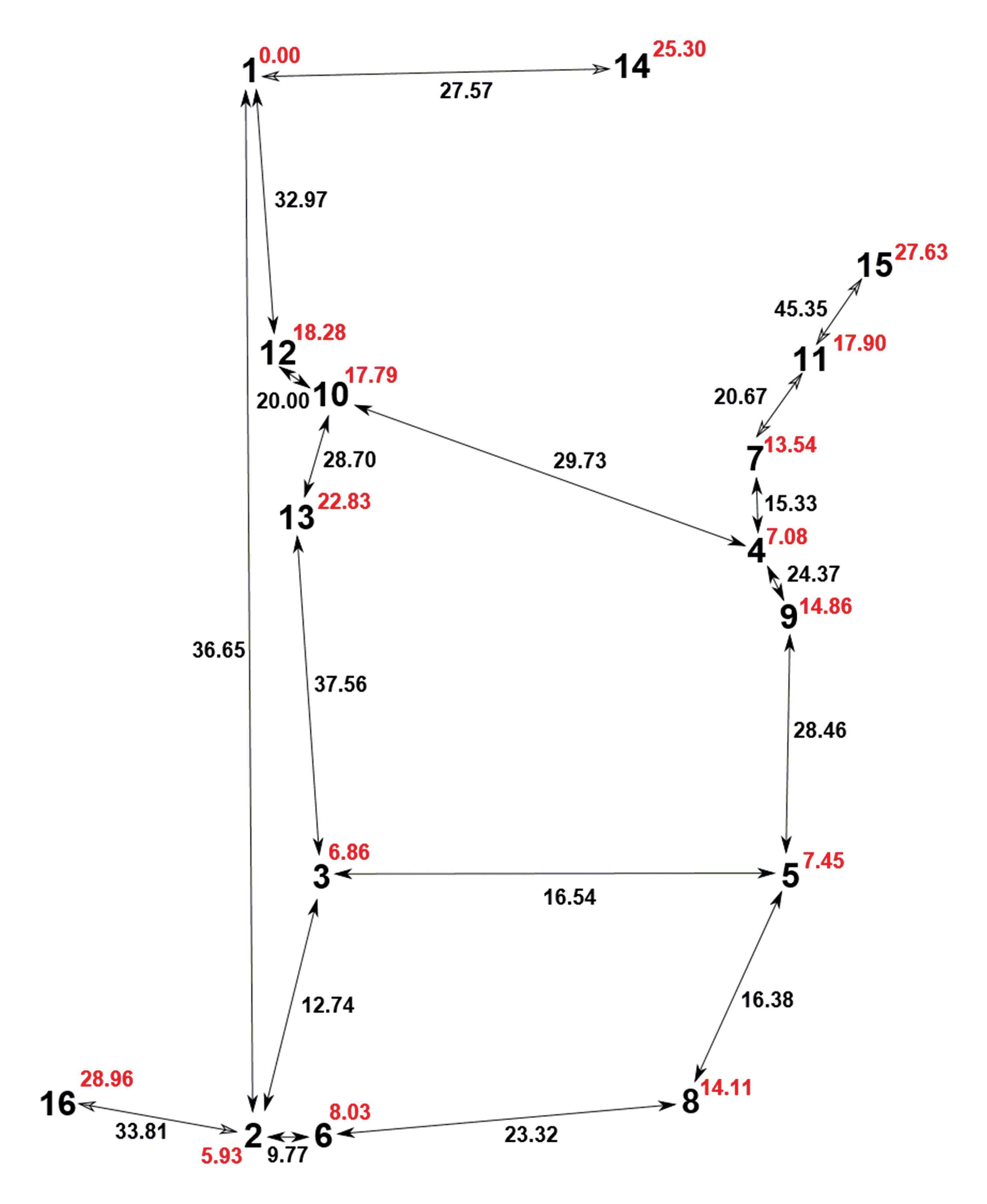

Supplement: S80 Fig — The float numbers express the energies of the conformers (red number) and the energy barriers of the conformation changes (black number). (TIF) [file pone.0144613.s080.tif]

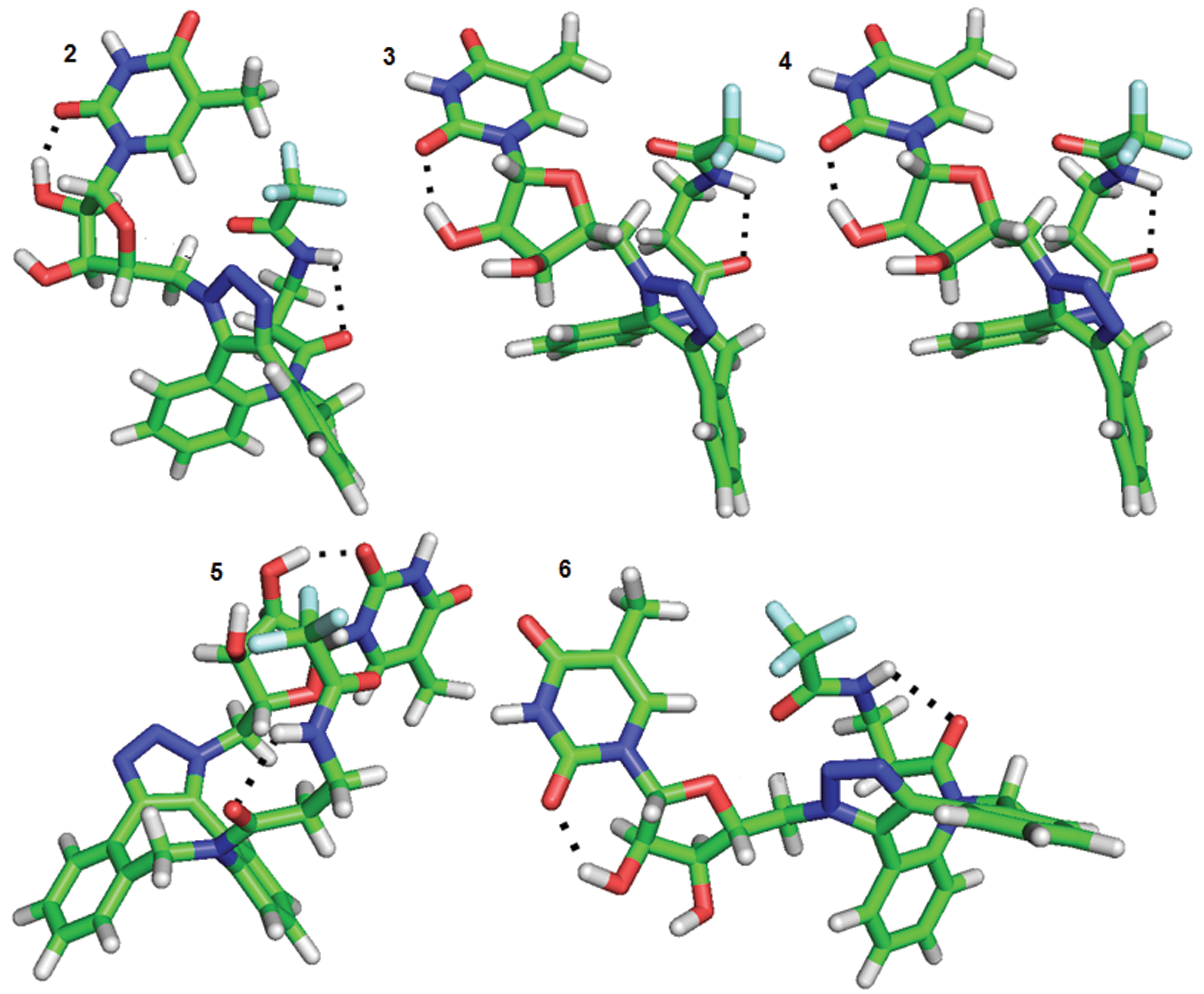

Supplement: S81 Fig — (TIF) [file pone.0144613.s081.tif]

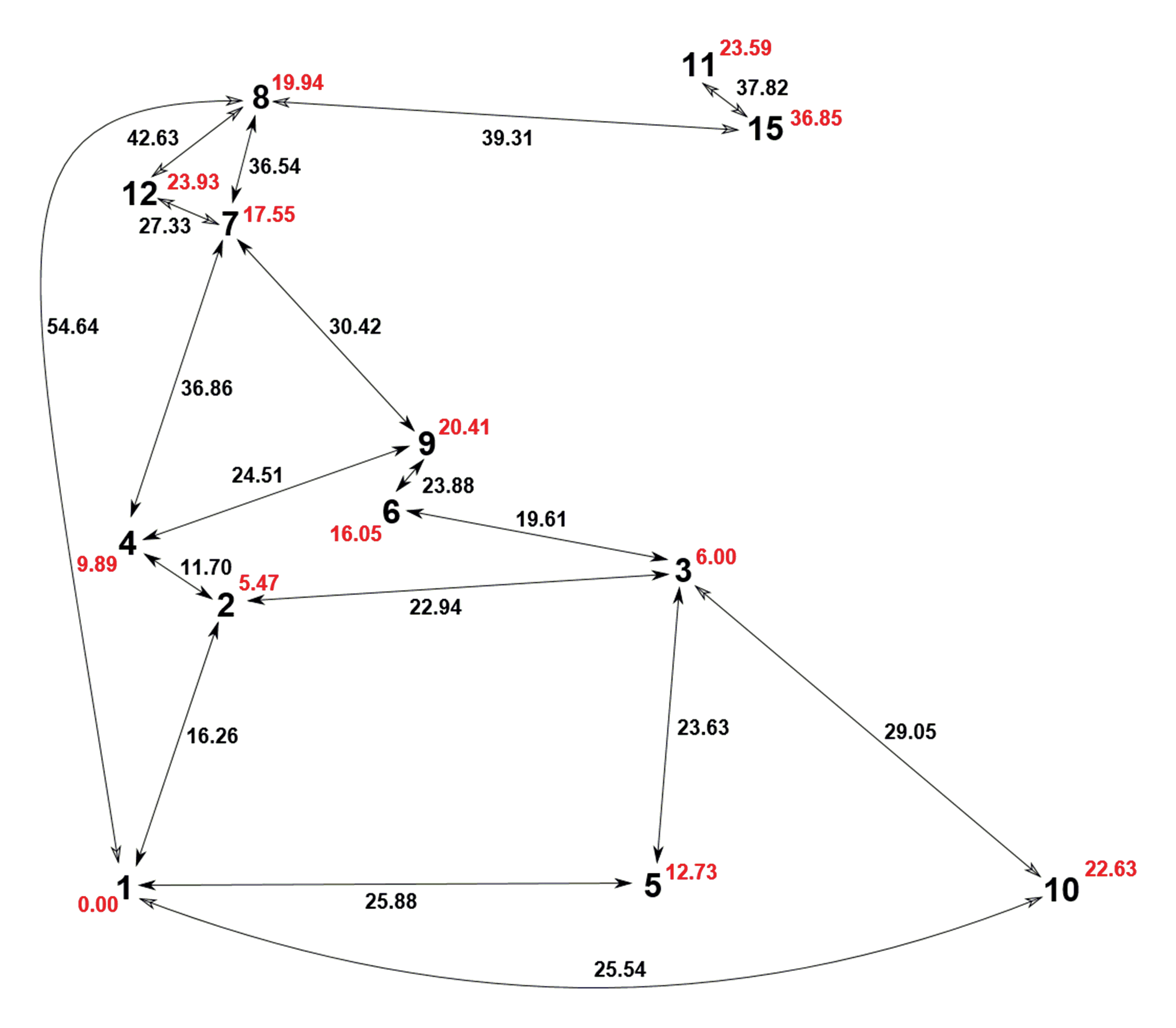

Supplement: S82 Fig — The float numbers express the energies of the conformers (red number) and the energy barriers of the conformation changes (black number). (TIF) [file pone.0144613.s082.tif]

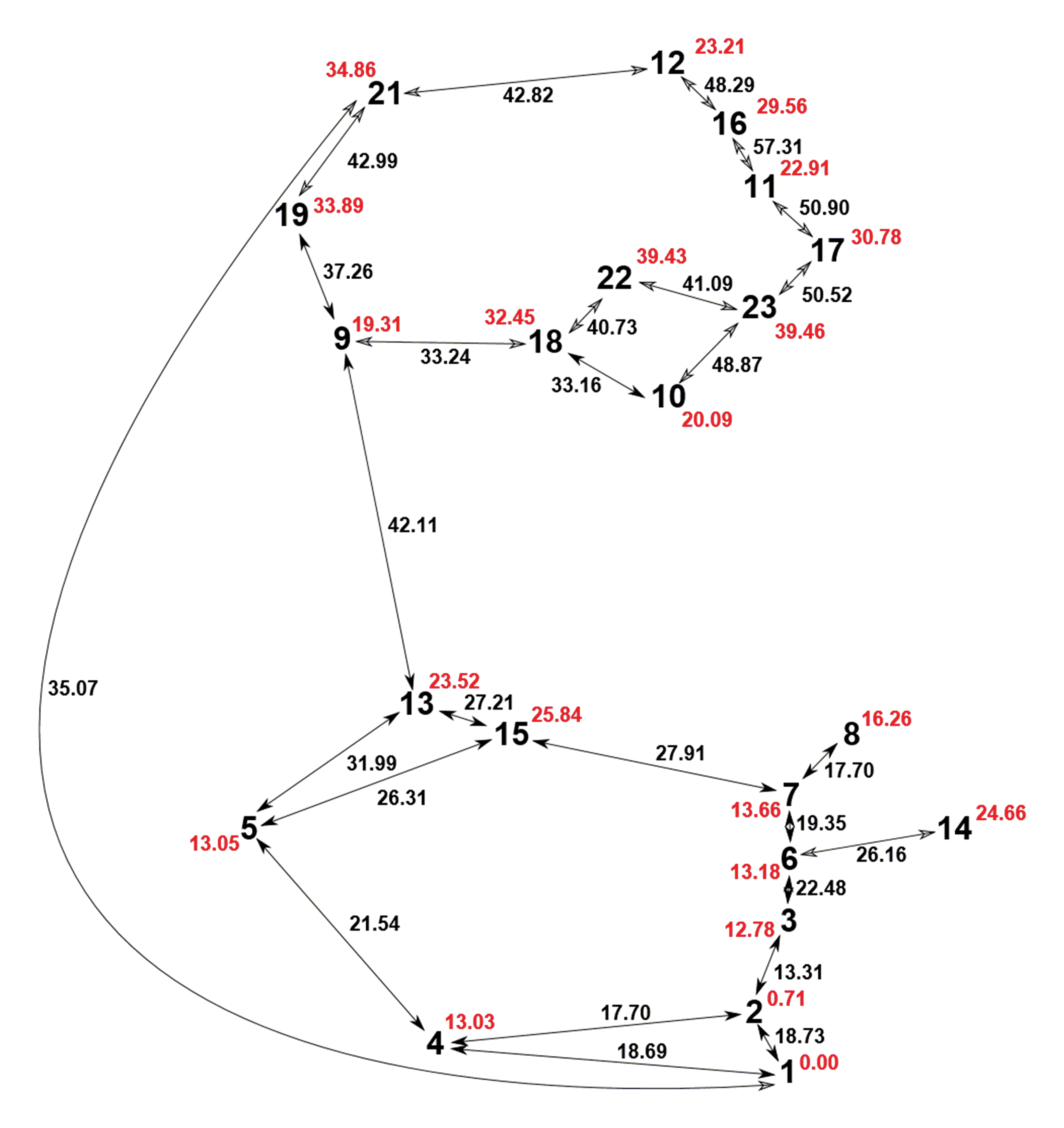

Supplement: S83 Fig — The float numbers express the energies of the conformers (red number) and the energy barriers of the conformation changes (black number). (TIF) [file pone.0144613.s083.tif]

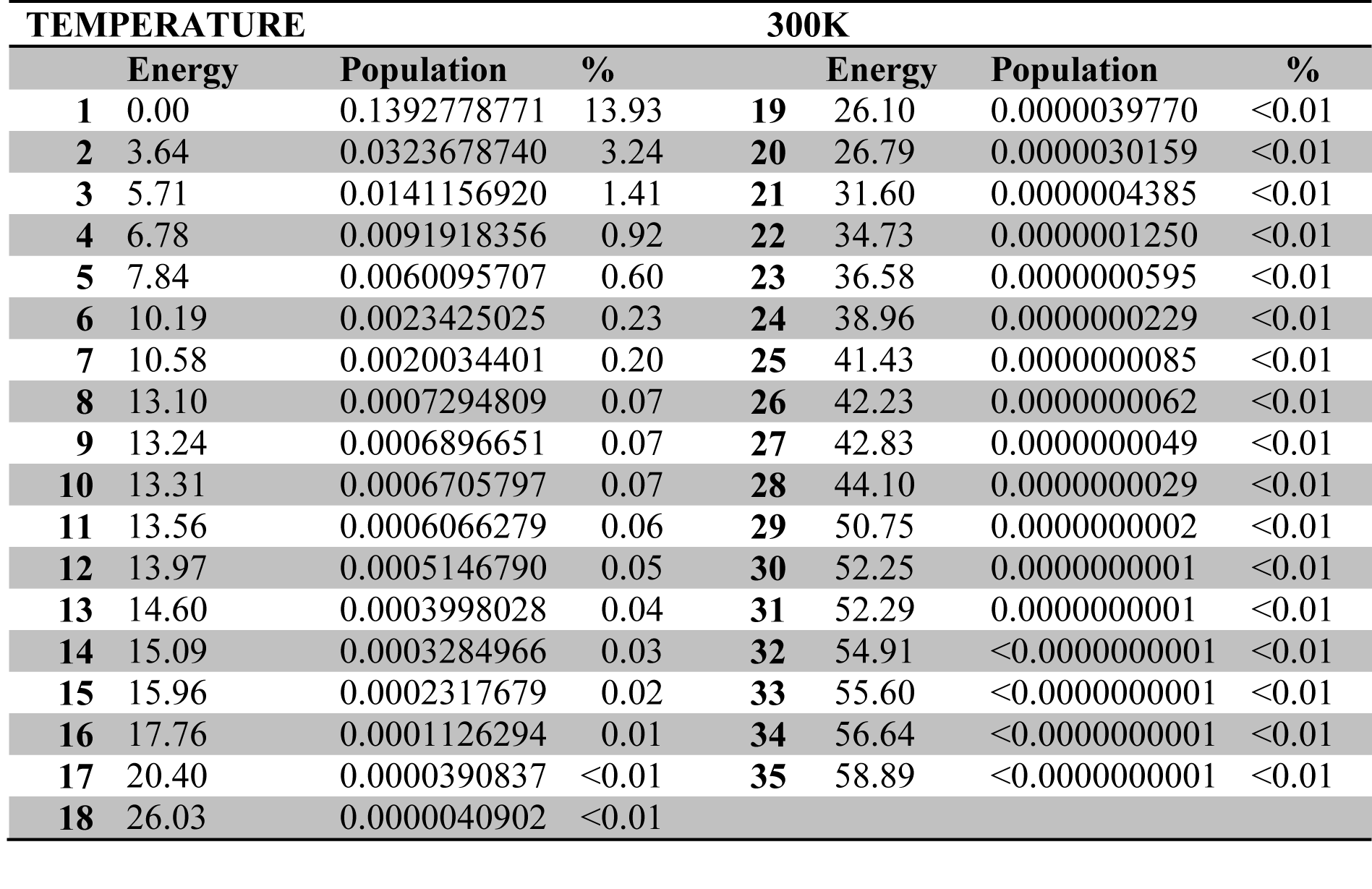

Supplement: S1 Table — (TIF) [file pone.0144613.s084.tif]

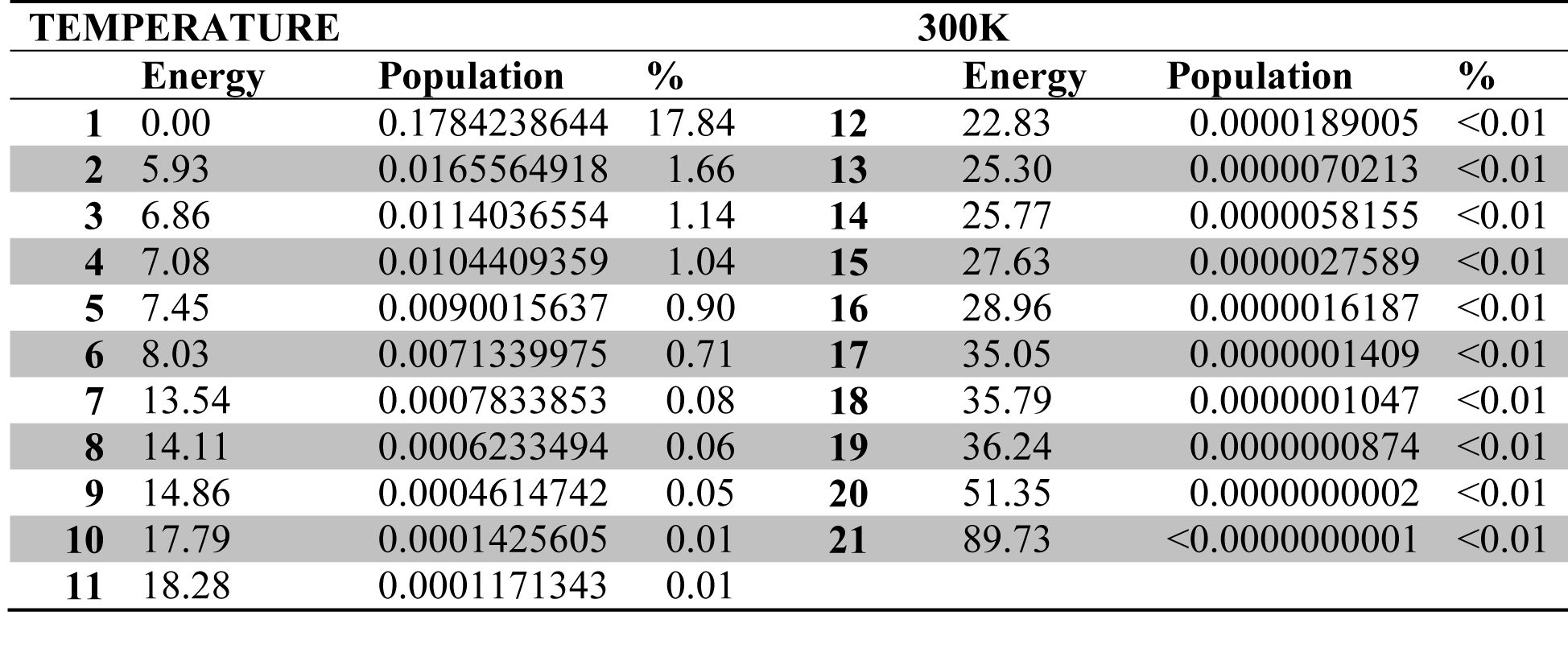

Supplement: S2 Table — (TIF) [file pone.0144613.s085.tif]

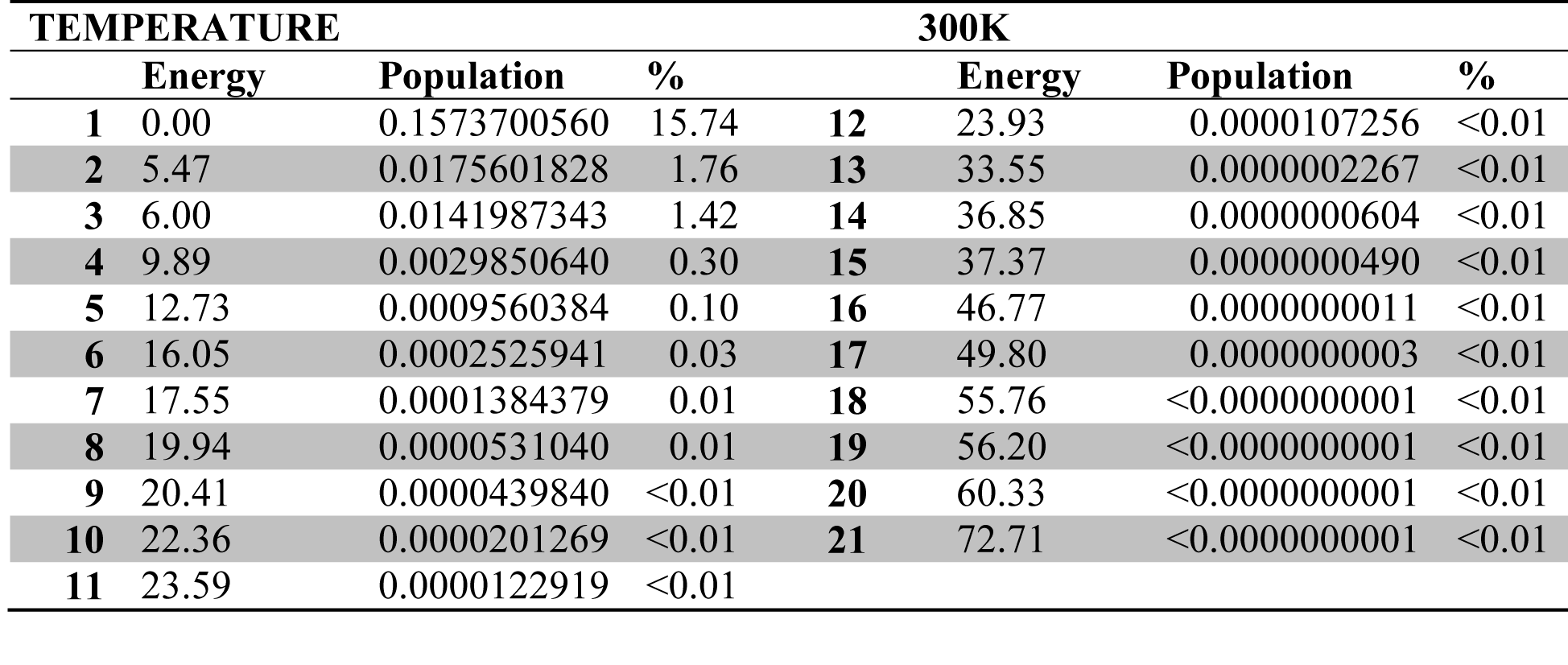

Supplement: S3 Table — (TIF) [file pone.0144613.s086.tif]

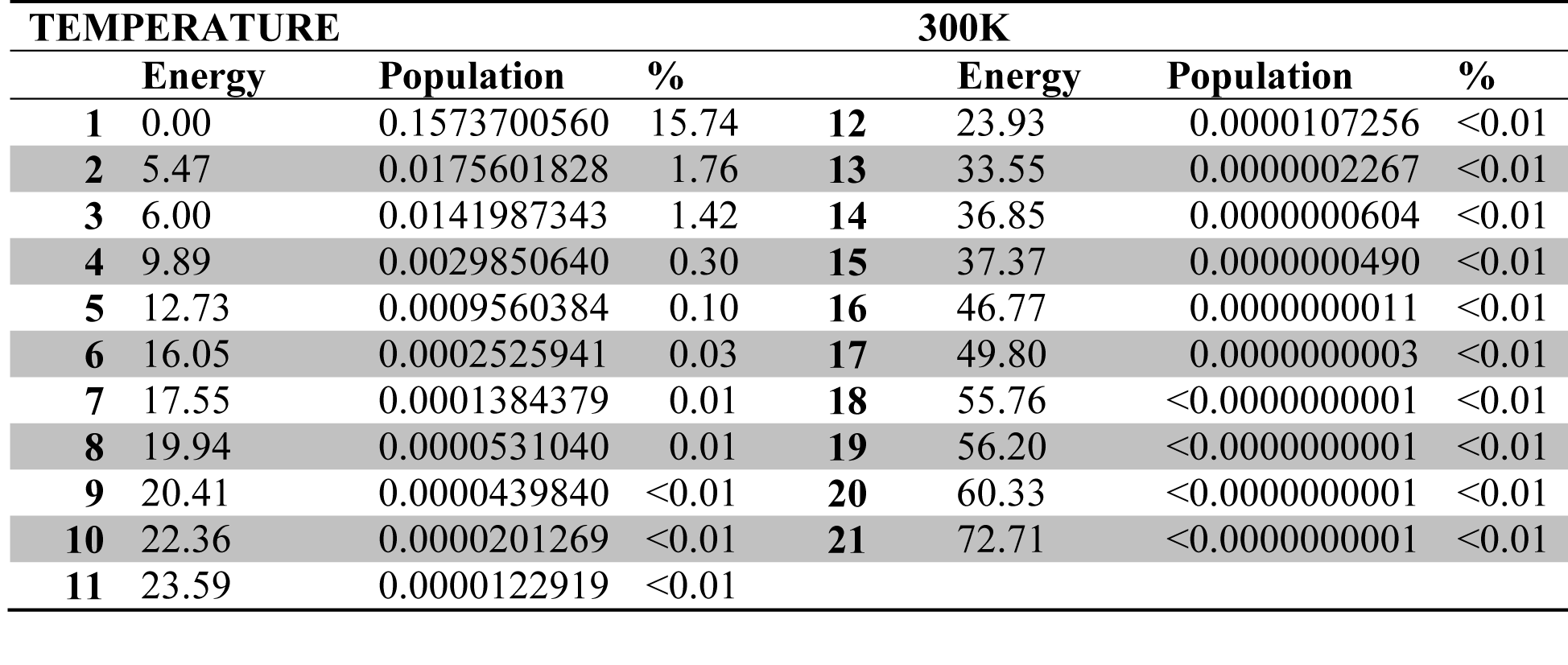

Supplement: S4 Table — (TIF) [file pone.0144613.s087.tif]
